# Genome-wide association study of yield-related traits in common wheat (*Triticum aestivum* L.) under normal and drought treatment conditions

**DOI:** 10.3389/fpls.2022.1098560

**Published:** 2023-01-04

**Authors:** Jie Zhao, Lijing Sun, Huimin Gao, Mengyun Hu, Liming Mu, Xiaohu Cheng, Jianbing Wang, Yun Zhao, Qianying Li, Peinan Wang, Hui Li, Yingjun Zhang

**Affiliations:** ^1^Institute of Cereal and Oil Crops, Laboratory of Crop Genetics and Breeding of Hebei, Hebei Academy of Agriculture and Forestry Sciences, Shijiazhuang, China; ^2^Institute of Cash Crops, Hebei Academy of Agriculture and Forestry Sciences, Shijiazhuang, China; ^3^Institute of Cereal Crops, Dingxi Academy of Agricultural Sciences, Dingxi, China

**Keywords:** common wheat, yield, GWAS, drought, KASP markers

## Abstract

The primary goal of modern wheat breeding is to develop new high-yielding and widely adaptable varieties. We analyzed four yield-related agronomic traits in 502 wheat accessions under normal conditions (NC) and drought treatment (DT) conditions over three years. The genome-wide association analysis identified 51 yield-related and nine drought-resistance-related QTL, including 13 for the thousand-grain weight (TGW), 30 for grain length (GL), three for grain width (GW), five for spike length (SL) and nine for stress tolerance index (STI) QTL in wheat. These QTL, containing 72 single nucleotide polymorphisms (SNPs), explained 2.23 – 7.35% of the phenotypic variation across multiple environments. Eight stable SNPs on chromosomes 2A, 2D, 3B, 4A, 5B, 5D, and 7D were associated with phenotypic stability under NC and DT conditions. Two of these stable SNPs had association with TGW and STI. Several novel QTL for TGW, GL and SL were identified on different chromosomes. Three linked SNPs were transformed into kompetitive allele-specific PCR (KASP) markers. These results will facilitate the discovery of promising SNPs for yield-related traits and/or drought stress tolerance and will accelerate the development of new wheat varieties with desirable alleles.

## Introduction

Wheat (*Triticum aestivum* L.), one of the most widely cultivated and most important cereal crops, provides approximately 20% of the dietary calories and proteins for human nutrition (Wang et al., 2021; Li et al., 2022a). However, climate stress and depleting fresh water for agricultural irrigation have severely affected wheat production, and drought stress is a major threat to wheat yield (Ahmed et al., 2022). Wheat is particularly susceptible to drought-induced stress throughout the growth period, and therefore, mining drought-stable QTL is vital for increasing wheat yield.

Wheat yield is a complex trait affected by grain and spike agronomic characteristics, including thousand-grain weight (TGW), grain length (GL), grain width (GW), and spike length (SL) (Li et al., 2019). In the present years, numerous QTL for wheat yield were mapped on 21 wheat chromosomes (Tshikunde et al., 2019; Cao et al., 2020). Several genes associated with wheat yield have been cloned in rice or *Arabidopsis*. These homologous genes include *TaCwi-A1* (Ma et al., 2012), *TaCKX6-D1* (Zhang et al., 2012), *TaSAP1* (Chang et al., 2013), *TaGASR7-A1* (Dong et al., 2014), *TaGW2A* (Hong et al., 2014), *TaGS-D1* (Zhang et al., 2014b), *TaCWI-4A* (Jiang et al., 2015), *TaCWI-5D* (Jiang et al., 2015), *TaCYP78A3* (Ma et al., 2015), *TaTGW6-A1* (Hanif et al., 2016), *TaTGW-7A* (Hu et al., 2016), *TaGS5-3A* (Ma et al., 2016), *TaGS5-A1* (Wang et al., 2016), *TaFlo2-A1* (Sajjad et al., 2017), *TaBT1* (Wang et al., 2019), *TaDA1* (Liu et al., 2020), *TaPRR1* (Sun et al., 2020), *TaSPL14* (Cao et al., 2021), *TaPGS1* (Guo et al., 2022), *TaIAA21* (Jia et al., 2021), and *TaPIN1s* (Yao et al., 2021). Many yield-related QTL/genes have been detected, but limited research has investigated their consistency in stressful and optimal environments. Thus, elucidating the genetic basis of grain and spike morphometric traits under diverse conditions is crucial for improving wheat yield and yield stability.

The rapid development of sequencing and high-density marker genotyping technologies has made GWAS (genome-wide association study) an effective method of identifying the quantitative variation of complex crop traits (Nordborg and Weigel, 2008; Hayes, 2013). The GWAS technique has high resolution and accuracy and has been widely used to study agronomic traits (Pang et al., 2020) and biotic and abiotic resistance (Kang et al., 2020; Shan et al., 2022) in wheat. In addition, GWAS performs genome-scale sequencing of numerous cultivars with diverse genetic backgrounds, thus, accelerating the genetic dissection of complex traits in crops (Juliana et al., 2022; Shan et al., 2022).

In this study, 502 Chinese wheat accessions were genotyped using the wheat 15K SNP array. The TGW, GL, GW, SL and STI (stress tolerance index) phenotypes were analyzed under normal (NC) and drought treatment (DT) conditions. Next, GWAS was performed using multi-year experimental data to identify high confidence loci associated with yield-related traits in a hexaploidy wheat genome under the two conditions. The findings will improve the understanding of the genetic mechanism of wheat yield-related and drought resistance traits and provide new genetic loci for breeding new high-yielding and stress-resistant wheat varieties.

## Materials and methods

### Materials and field experiment

Five hundred and two hexaploidy wheat collections in China were used in this experiment (**Supplementary Table 1**). The wheat collections were mainly from the Winter Wheat Region of the Yellow and Huai Valleys, which accounted for approximately 75% of the wheat produced in China. This study was established in the Dishang Experimental Station of the Institute of Cereal and Oil Crops, Hebei Academy of Agriculture and Forestry Sciences, Shijiazhuang, Hebei Province, China, located at 38°N and 114°83′E. The field experiments were conducted over three consecutive years (2018 – 2019, 2019 – 2020, 2020 – 2021). The average rainfalls in the October – May season of 2018 – 2019, 2019 – 2020, and 2020 – 2021 were 101, 131, and 75 mm, respectively (**Supplementary Figure 1**). Plants under normal conditions (NC) were irrigated twice at the jointing and booting stages in 2018 – 2019 (NC19), 2019 – 2020 (NC20), and 2020 – 2021 (NC21). In contrast, drought-treated plants were not irrigated throughout the growth period in 2018 – 2019 (DT19), 2019 – 2020 (DT20), and 2020 – 2021 (DT21). All plants were grown in rows 3 by 0.22 m in length and width at a sowing rate of 84 seeds per row in randomized complete blocks. Trials were fertilized and maintained free from weeds, insects, and diseases.

### Phenotyping and statistical analysis

Five randomly selected spikes were sampled for manual measurement, and then to generate one average value. The grain traits (TGW, GL, and GW) were recorded using a scaled-camera-assisted phenotyping system (Wanshen Detection Technology Co., Ltd., Hangzhou, China). The stress tolerance index of each accession was calculated using the formula: STI = (TGW.DT × TGW.NC)/(X.NC^2^), where TGW.NC and TGW.DT were the TGW for each accession under normal and drought treatment conditions, respectively; and X.NC was the average TGW of all accessions under normal conditions (Fernandez, 1992).

All statistical analysis was conducted in R v4.1.0 (Yu et al., 2019) and SPSS v16.0 software (SPSS Inc, Chicago). Pearson’s correlation coefficient was determined for all traits in the different environments. The formula *h*^2^ = *σ*_g_^2^/(*σ*_g_^2^ + *σ*_gy_^2^/*y* + *σ*_er_^2^/*yr*) was used to calculate broad sense heritability (*h*^2^), where *σ*_g_^2^, *σ*_gy_^2^, and *σ*_er_^2^ represent the genotypic variance, genotype by year effect, and the residual error, respectively. *y* and *r* are the number of years and replications, respectively.

### SNP genotyping

The wheat iSelect 15K SNP array from China Golden Maker Biotechnology Co., Ltd., Beijing (http://www.cgmb.com.cn/) was used for genotyping the 502 accessions. The array was identified with specific genetic positions based on the consensus map (Li et al., 2021). The 13,947 SNPs were filtered using PLINK v1.9 (http://pngu.mgh.harvard.edu/purcell/plink/) (Purcell et al., 2007) to delete loci with minor allele frequency (MAF) (< 5%) and low-quality SNPs (with > 20% missing data). After filtering, 13,705 SNPs remained and were used for GWAS. The physical maps of all polymorphic SNPs were obtained by searching the reference sequence IWGSC RefSeq v1.0 2018 using URGI BLAST (http://wheat-urgi.versailles.inra.fr/Tools/BLAST ).

### Population structure, kinship, and linkage disequilibrium

The phylogenetic tree was generated as previously described (Zhao et al., 2020). The principal component analysis (PCA) was constructed using PLINK v1.9, and the percentage of variance explained by the top 10 PCs was plotted to determine the optimal number of PCs. Furthermore, the relative kinship matrix (K-matrix) between accessions was calculated using TASSEL v5.2.73 (Bradbury et al., 2007). Linkage disequilibrium (LD) was estimated as the squared allele frequency correlation (*r*^2^) using PLINK v1.9. The LD decay distance was obtained by constructing a scatterplot of *r*^2^ values against SNP pairs distance and fitting the points to a smooth curve using the R package “ggplot2”.

### Genome-wide association mapping

The mixed linear model (MLM) (PCA+K) was used for GWAS using the GAPIT package (Wang and Zhang, 2021) in R software. The first five principal components were included in the GWAS model. The mixed linear model accounts for false positives caused by the population structure and relative kinship. For this reason, the *P* values with cutoff set at 0.05 was highly restrictive. Considering the potential risk of type II error, and combining the GWAS results of the three years, the -log_10_ (*P* value) ≥ 3.0 were regarded as significant marker-trait associations (MTAs), as shown in Manhattan plots (Wang et al., 2021). Important *P* value distributions (observed *P* values plotted against expected *P* values) are shown in Q-Q plots.

### KASP maker development

The SNP makers highly associated with grain and spike traits were selected and converted to KASP (kompetitive allele-specific PCR) markers. The KASP markers were used for PCR amplification of wheat DNA in 3 μL final volumes containing 40 –50 ng of genomic DNA, 0.75 μL of 2 × KASP Master Mix (LGC Genomics, product code: KBS-1050-112), and 0.0417 μL of assay primer mix (12 mM of each allele-specific primer and 30 mM of the common primer). The cycling conditions followed: 94°C for 15 min, ten cycles of 94°C for 20 s, touchdown starting at 61°C for 20 s (decreasing 0.6°C per cycle), 26 cycles of 94°C for 20 s, and 55°C for 1 min. The end-point fluorescence data were visualized with a microplate reader (Omega, BMG LABTECH, Germany) and analyzed by the Kluster-Caller software (LGC Genomics). The Student’s *t*-tests were used to compare allelic effects of the phenotypic data and the effectiveness of the associated SNPs using KASP markers. The primers used in this research were listed in **Supplementary Table 2**.

## Results

### Phenotype assessments

**Figure 1:**
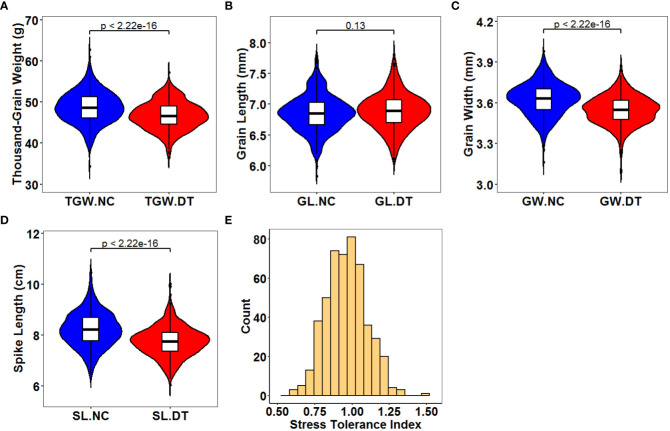
Distribution of the average phenotype for the 502 wheat accessions across all environments. **(A)** Thousand-grain weight under normal (TGW.NC) and drought treatment conditions (TGW.DT); **(B)** Grain length under normal (GL.NC) and drought treatment conditions (GL.DT); **(C)** Grain width under normal (GW.NC) and drought treatment conditions (GW.DT); **(D)** Spike length under normal (SL.NC) and drought treatment conditions (SL.DT); **(E)** Stress tolerance index. The center and edges of the boxplot represent the median and the 25th or 75th percentile respectively, while the whiskers represent the median ± 1.5 × IQR (interquartile interval).

The traits of 502 wheat accessions were characterized during three crop seasons (2018 – 2019, 2019 – 2020, and 2020 – 2021) under normal and drought treatment conditions (**Figure 1**, **Supplementary Figure 2**, and **Table 1**). The correlations were analyzed between the three crop seasons for TGW, GL, GW, and SL (**Supplementary Figure 3**). For all the detected phenotypes, positive correlations were found among different crop seasons under both normal and drought treatment conditions. Therefore, we compared the phenotypes under the different conditions based on the average values. All genotypes exhibited significant differences for each trait, with the coefficients of variation (CV) ranging from 3.83% to 16.39%. Except for GL, the normal and drought treatment conditions were significantly different (*P* < 0.001) for TGW, GW, and SL. The average TGW under the normal conditions was 48.62 g (ranging from 31.31 to 65.76 g), whereas the average TGW under the drought treatment conditions was 46.68 g (ranging from 32.40 to 63.12 g). Correspondingly, the average STI was 0.97 (ranging from 0.54 to 1.45). Traits GW and SL decreased from 3.62 to 3.55 mm and 8.22 to 7.75 cm under the DT conditions compared to the normal conditions. In contrast, the average GL differed slightly (0.03 cm) between the two conditions (**Table 1**). Therefore, drought treatment environmentally stressed the plants, reducing their spike and grain yield. All detected traits showed high broad sense heritability (*h*^2^, 76.16 – 95.66%) under the two conditions.

**Table 1:** Statistics for thousand-grain weight (TGW), grain length (GL), grain width (GW), spike length (SL) and stress tolerance index (STI) in the association population under normal (NC) and drought treatment (DT) conditions.

Traits	Mean ± SD^a^	Range	CV (%)^b^	*h^2^* (%)^c^
TGW.NC	48.62 ± 5.25	31.31 – 65.76	10.79	84.01
GL.NC	6.86 ± 0.34	5.72 – 7.97	4.9	94.55
GW.NC	3.62 ± 0.16	3.06 – 4.18	4.34	87.02
SL.NC	8.22 ± 0.92	5.20 – 11.60	11.19	88.08
TGW.DT	46.68 ± 4.34	32.40 – 63.12	9.29	88.23
GL.DT	6.89 ± 0.33	5.79 – 8.05	4.73	95.66
GW.DT	3.55 ± 0.14	2.86 – 4.02	3.83	89.42
SL.DT	7.75 ± 1.00	5.30 – 11.70	12.84	76.16
STI	0.97 ± 0.16	0.54 – 1.45	16.39	91.84

^a^SD, standard deviations; ^b^CV, coefficient of variation; ^c^*h^2^*, broad sense heritability.

**Figure 2:**
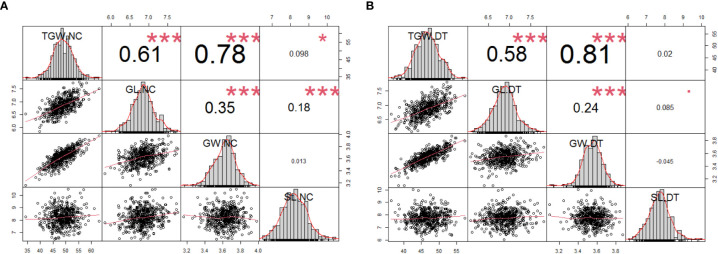
Phenotypic performances, distribution, and correlation coefficients for the 502 wheat accessions using the average phenotypic data under normal **(A)** and drought treatment conditions **(B)**. The frequency distribution of the phenotypic data for each trait is shown in the histograms. The *X*-*Y* scatter plot with the adjusted Pearson’s coefficients. *P < 0.05, ***P < 0.001. TGW.NC, thousand-grain weight under normal conditions; GL.NC, grain length under normal conditions; GW.NC, grain width under normal conditions; SL.NC, spike length under normal conditions; TGW.DT, thousand-grain weight under drought treatment conditions; GL.DT, grain length under drought treatment conditions; GW.DT, grain width under drought treatment conditions; SL.DT, spike length under drought treatment conditions.

We analyzed the correlations between the four traits (**Figure 2**). Under the normal conditions, any two of TGW, GL, and GW were positively correlated (*r* = 0.61*** between TGW and GL, *r* = 0.78*** between TGW and GW, and *r* = 0.35*** between GL and GW). Moreover, SL was positively correlated with TGW and GL (0.098* and 0.18***) respectively and uncorrelated with GW (**Figure 2A**). The correlations between TGW, GL, and GW were similar under NC and DT conditions. Additionally, SL was weakly correlated with GL (*r* = 0.085*) but uncorrelated with TGW and GW under DT (**Figure 2B**).

### Statistical analysis of genotypes

All 502 accessions were genotyped using the wheat iSelect 15K array. After filtering (missing rate > 20% and minor allele frequency < 0.05), 13,705 high-quality SNPs were retained and used for genetic analysis. These SNPs were distributed on all 21 wheat chromosomes, with 4916, 5326, and 3463 SNPs in the A, B, and D sub-genomes. Chromosome 3B had the most SNPs (1024) but the shortest average distance between SNPs (833 kb). However, 4D had the least (264 SNPs and 2056 kb) (**Supplementary Figure 4**).

### Population structure and LD analysis

**Figure 3:**
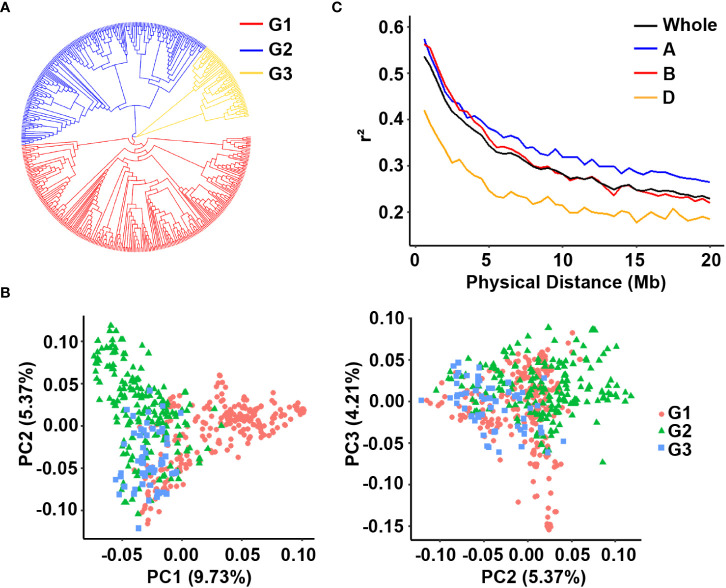
Analysis of the population structure of the 502 wheat accessions. **(A)** Neighbor-joining tree of all wheat accessions; **(B)** Principal component analysis showing the population structure in the diversity panel; **(C)** LD decay value in the whole genome, A sub-genome, B sub-genome and D sub-genome of wheat.

We used different approaches to cluster the accessions into different groups and assessed the similarity of the results. The neighbor-joining tree divided the panel into three major groups (**Figure 3A**). Similarly, PCA classified the panel into three groups despite the numerous admixtures. These results revealed a weak population structure since the first three principal components collectively explained only approximately 19.31% of the total variance, i.e., 9.73, 5.37, and 4.21% for PC1, PC2, and PC3, respectively (**Figure 3B**). Overall, the above methods classify the populations into three subgroups.

The filtered SNP data were used to calculate LD. The average *r*^2^ values for the A, B, and D sub-genomes and the whole genome gradually decreased with increasing pairwise distance (**Figure 3C**). When the cutoff threshold for *r*^2^ was half of its maximum value, the A sub-genome had the longest LD decay distance (approximately 12 Mb) compared to the B and D sub-genomes (> 10 Mb). The LD decay distance for the entire genome was approximately 12 Mb.

### GWAS of traits under two conditions

**Figure 4:**
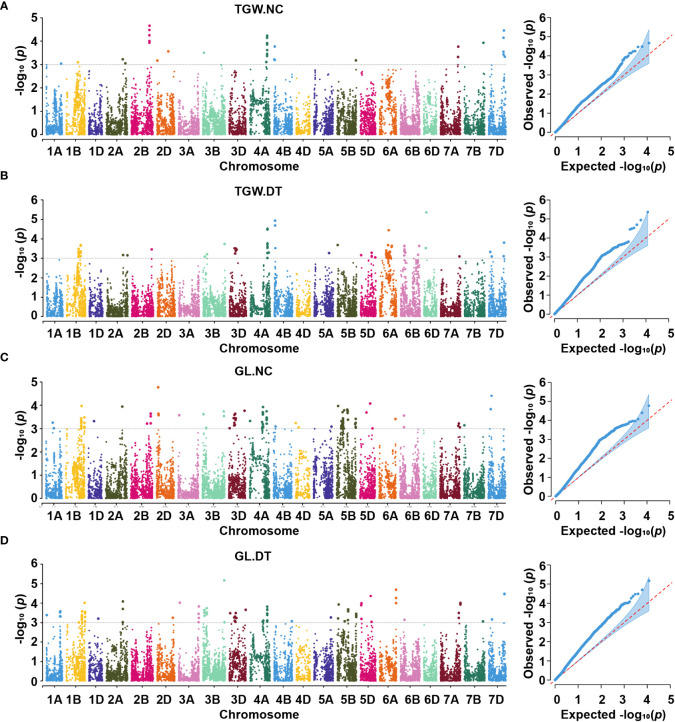
Manhattan and Q-Q plots for thousand-grain weight **(A, B)** and grain length **(C, D)** under two conditions. TGW.NC, thousand-grain weight under normal conditions; TGW.DT, thousand-grain weight under drought treatment conditions; GL.NC, grain length under normal conditions; GL.DT, grain length under drought treatment conditions.

**Figure 5:**
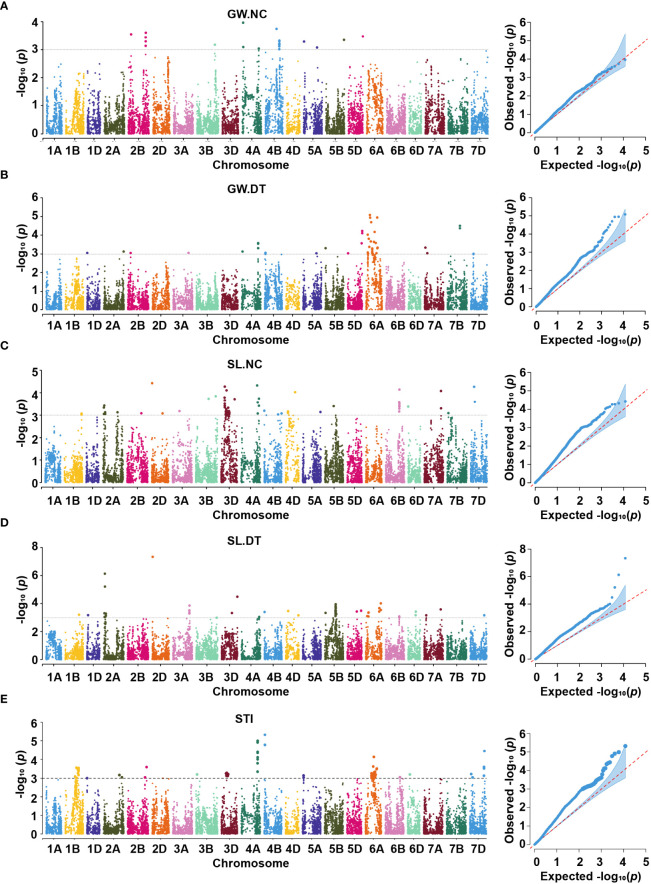
Manhattan and Q-Q plots for grain width **(A, B)**, spike length **(C, D)** and stress tolerance index **(E)** under two conditions. GW.NC, grain width under normal conditions; GW.DT, grain width under drought treatment conditions; SL.NC, spike length under normal conditions; SL.DT, spike length under drought treatment conditions; STI, stress tolerance index.

We performed GWAS for all the traits with the MLM in different environments under both conditions. We defined the significant and repetitive SNPs in at least two environments as true and reliable association loci. The purpose was to eliminate the influence of the environmental background and explain the true components of genetic variation. The Q-Q and Manhattan plots based on the average values of the individual traits are shown in **Figures 4**, **5**. The GWAS identified 666 and 821 SNPs in different environments under NC and DT conditions, respectively, including 67 significant SNPs in 51 QTL from the multiple environments (**Tables 2**, **3** and **Supplementary Table 3**). In addition, 311 SNPs were detected for STI in different environments. Among them, 18 high confidence SNPs in nine QTL for STI were detected under more than one environment (**Table 4** and **Supplementary Table 4**).

**Table 2:** The QTL and significant SNPs identified by GWAS for the yield-related traits under normal (NC) conditions.

Trait	QTL	SNP	Chr	Position (bp)^a^	Environment	P value	PVE (%)^b^	Reference^c^
TGW.NC	*qNTGW-1B*	*AX-111169510*	1B	430,155,688	NC20, NC21, NAVE	2.90E-04	7.35	
	*qNTGW-2A*	*AX-109290429*	2A	608,865,127	NC19, NC21, NAVE	1.27E-04	2.91	Mccartney et al, 2005
	*qNTGW-2B*	*AX-109505207*	2B	666,474,502	NC19, NC21, NAVE	3.29E-05	3.25	Groos et al., 2003
		*AX-111661669*	2B	666,653,511	NC19, NC21, NAVE	5.68E-05	3.05	
		*AX-111728700*	2B	667,415,257	NC19, NC21, NAVE	9.98E-05	2.84	
		*AX-94979731*	2B	667,805,353	NC19, NC21, NAVE	5.87E-05	3.20	
		*AX-95219673*	2B	671,741,110	NC19, NC21, NAVE	2.16E-05	3.40	
	*qNTGW-4A.1*	*AX-110171894*	4A	605,753,144	NC19, NC21	2.04E-04	3.44	Zheng et al., 2010;
		*AX-109626103*	4A	605,764,861	NC19, NC21	3.00E-04	3.26	Jiang et al., 2015
		*AX-108777965*	4A	605,767,873	NC19, NC21	3.38E-04	2.54	
		*AX-110961937*	4A	605,785,021	NC19, NC21, NAVE	2.08E-04	2.72	
	*qNTGW-4A.2*	*AX-109375057*	4A	633,308,367	NC20, NC21, NAVE	7.27E-05	2.96	Cui et al., 2016;
		*AX-110468999*	4A	634,108,610	NC20, NC21, NAVE	1.39E-04	2.72	Gao et al., 2015;
		*AX-111453918*	4A	634,833,572	NC20, NC21, NAVE	1.22E-04	2.77	Guan et al., 2018
		*AX-108950546*	4A	635,764,214	NC20, NC21, NAVE	5.93E-05	3.03	
	*qNTGW-7B*	*AX-89432708*	7B	701,338,884	NC20, NC21, NAVE	6.98E-06	4.44	Zhang et al., 2014a; Gao et al., 2015; Cao et al., 2020
	*qNTGW-7D.1*	*AX-108758043*	7D	547,277,450	NC20, NC21, NAVE	7.26E-05	2.96	
	*qNTGW-7D.2*	*AX-108770812*	7D	561,926,335	NC20, NC21, NAVE	3.44E-05	3.23	Li et al., 2007
GL.NC	*qNGL-1B.1*	*AX-111130381*	1B	565,517,030	NC20, NC21, NAVE	4.60E-04	2.30	
	*qNGL-1B.2*	*AX-110402488*	1B	580,075,805	NC20, NC21, NAVE	1.08E-04	2.82	
	*qNGL-1B.3*	*AX-109353011*	1B	685,283,930	NC20, NC21, NAVE	3.31E-04	2.42	
	*qNGL-2A*	*AX-109290429*	2A	608,865,127	NC19, NC21, NAVE	7.90E-05	3.98	
	*qNGL-2D.1*	*AX-109013500*	2D	63,568,198	NC20, NC21, NAVE	7.59E-06	3.98	
	*qNGL-2D.2*	*AX-111559873*	2D	79,095,877	NC20, NC21, NAVE	5.09E-05	3.25	
	*qNGL-3B.1*	*AX-111534973*	3B	41,633,676	NC19, NC20, NAVE	2.43E-04	2.53	
	*qNGL-3B.2*	*AX-110418888*	3B	785,432,286	NC19, NC20, NC21, NAVE	1.85E-04	2.63	Kumar et al., 2016
		*AX-109881148*	3B	793,075,912	NC19, NC20, NAVE	1.95E-04	2.76	
	*qNGL-3D*	*AX-109392999*	3D	236,169,118	NC19, NC20, NAVE	2.34E-04	2.54	
	*qNGL-4B*	*AX-110436979*	4B	73,152,359	NC19, NC21	1.60E-04	2.82	
		*AX-110361956*	4B	75,741,547	NC19, NC21, NAVE	1.89E-04	2.75	
	*qNGL-5B.1*	*AX-110398218*	5B	57,493,343	NC19, NC21, NAVE	1.09E-04	2.82	Yang et al., 2020
	*qNGL-5B.2*	*AX-110516200*	5B	277,898,057	NC20, NC21, NAVE	1.65E-04	2.67	Li et al., 2018
	*qNGL-5B.3*	*AX-108733256*	5B	702,228,057	NC19, NC20, NAVE	1.03E-04	3.85	Li et al., 2015
	*qNGL-5D.1*	*AX-111739361*	5D	244,617,835	NC20, NC21, NAVE	2.05E-04	2.59	
	*qNGL-5D.2*	*AX-110503408*	5D	379,836,128	NC20, NC21, NAVE	8.53E-05	2.91	
GW.NC	*qNGW-4A*	*AX-95629274*	4A	38,367,204	NC19, NC21, NAVE	1.08E-04	2.92	Li et al., 2018
SL.NC	*qNSL-6B*	*AX-109337721*	6B	528,705,208	NC20, NC21, NAVE	7.17E-05	3.15	

^a^Physical position of the SNP in the reference genome (IWGSC RefSeq v1.0); ^b^Phenotypic variation explained by the identified SNP; ^c^The reference for the near loci previously reported; NC19, NC20, NC21 and NAVE represented 2018 – 2019, 2019 – 2020, 2020 – 2021 and the mean value of three crop seasons for the yield-related traits under NC conditions, respectively.

**Table 3:** The QTL and significant SNPs identified by GWAS for the yield-related traits under drought treatment (DT) conditions.

Trait	QTL	SNP	Chr	Position (bp)^a^	Environment	P value	PVE (%)^b^	Reference^c^
TGW.DT	*qDTGW-4A*	*AX-109375057*	4A	633,308,367	DT20, DT21, DAVE	3.23E-05	3.26	Gao et al., 2015; Cui et al., 2016; Guan et al., 2018
	*qDTGW-5A*	*AX-109369427*	5A	546,521,932	DT19, DT20	9.24E-05	3.72	Cao et al., 2020;
		*AX-110016633*	5A	547,333,614	DT19, DT20, DAVE	5.17E-04	2.33	Li et al., 2018
	*qDTGW-5D*	*AX-110758473*	5D	292,945,585	DT19, DT21	4.27E-04	2.44	
	*qDTGW-6A*	*AX-109334618*	6A	328,130,345	DT19, DT20, DAVE	4.94E-05	3.08	Wang et al., 2011
	*qDTGW-7D*	*AX-108770812*	7D	561,926,335	DT19. DT21, DAVE	1.55E-04	2.69	Li et al., 2007
GL.DT	*qDGL-1A*	*AX-110046566*	1A	503,449,799	DT19, DT20, DT21, DAVE	2.95E-04	2.43	
		*AX-111802924*	1A	504,003,811	DT19, DT20, DT21, DAVE	4.70E-04	2.26	
	*qDGL-1B*	*AX-108737720*	1B	686,751,506	DT19, DT20, DAVE	2.85E-04	2.44	
		*AX-111190944*	1B	686,755,725	DT19, DT20, DAVE	5.12E-04	2.23	
		*AX-109847715*	1B	686,786,607	DT19, DT20, DAVE	3.36E-04	2.38	
	*qDGL-2A.1*	*AX-110451187*	2A	607,768,697	DT19, DT21, DAVE	2.52E-05	3.42	
		*AX-109290429*	2A	608,865,127	DT19, DT21, DAVE	1.29E-05	3.67	
	*qDGL-2A.2*	*AX-109425314*	2A	693,336,312	DT19, DT21	2.91E-04	3.31	
	*qDGL-2D*	*AX-109013500*	2D	63,568,198	DT20, DT21	4.85E-04	2.33	
	*qDGL-3A*	*AX-110707258*	3A	733,432,801	DT20, DT21, DAVE	1.47E-04	2.73	
		*AX-109281734*	3A	744,244,068	DT19, DT20, DAVE	5.23E-05	4.14	
	*qDGL-3B*	*AX-110418888*	3B	785,432,286	DT19, DT20, DT21, DAVE	6.71E-06	3.78	Kumar et al., 2016
	*qDGL-3D*	*AX-109500294*	3D	599,688,383	DT19, DT21, DAVE	1.25E-04	2.83	
	*qDGL-5B.1*	*AX-110398218*	5B	57,493,343	DT19, DT21, DAVE	1.19E-04	2.75	Yang et al., 2020
	*qDGL-5B.2*	*AX-109879806*	5B	699,999,185	DT20, DT21, DAVE	2.08E-04	2.64	Li et al., 2015;
		*AX-108757760*	5B	701,544,672	DT19, DT20, DAVE	2.56E-04	2.53	Kumar et al., 2016
		*AX-108733256*	5B	702,228,057	DT19, DT20, DAVE	3.86E-04	3.17	
	*qDGL-5D.1*	*AX-110521341*	5D	30,854,436	DT19, DT21, DAVE	7.43E-05	3.97	
	*qDGL-5D.2*	*AX-110503408*	5D	379,836,128	DT19, DT21, DAVE	4.30E-05	3.11	
	*qDGL-6A*	*AX-111034352*	6A	603,563,082	DT19, DT21, DAVE	2.01E-05	3.38	
		*AX-94788907*	6A	604,107,719	DT19, DT21, DAVE	5.52E-05	3.02	
	*qDGL-7A*	*AX-108748484*	7A	730,508,297	DT20, DT21, DAVE	9.05E-05	2.91	Gill et al., 2022
		*AX-110007752*	7A	730,855,963	DT20, DT21, DAVE	1.18E-04	2.75	
		*AX-109272527*	7A	731,240,127	DT20, DT21, DAVE	9.80E-05	2.82	
	*qDGL-7D*	*AX-108815963*	7D	577,348,898	DT19, DT21, DAVE	3.37E-06	5.50	
		*AX-109867095*	7D	577,937,328	DT19, DT21, DAVE	3.36E-06	5.50	
GW.DT	*qDGW-6A.1*	*AX-111041695*	6A	148,524,505	DT19, DT21, NAVE	8.24E-06	3.82	Li et al., 2022b
		*AX-110936625*	6A	158,068,188	DT19, DT21, NAVE	1.14E-05	3.70	
	*qDGW-6A.2*	*AX-110942969*	6A	428,454,560	DT19, DT21, NAVE	1.12E-05	3.71	Li et al., 2022b
SL.DT	*qDSL-2A*	*AX-108747720*	2A	58,539,285	DT19, DT20, DAVE	6.08E-06	4.01	
	*qDSL-2D*	*AX-110647062*	2D	23,025,488	DT19, DT20, DAVE	1.45E-08	6.31	Ma et al., 2007; Heidari et al., 2011; Wang et al., 2011; Wu et al., 2014; Deng et al., 2017; Chai et al., 2019; Li et al, 2022a;
	*qDSL-3D*	*AX-111027124*	3D	400,781,396	DT19, DT20, DAVE	1.79E-04	2.70	Yu et al., 2014
	*qDSL-5B*	*AX-111124661*	5B	437,564,786	DT19, DT20	2.44E-04	2.63	Gill et al., 2022;

^a^Physical position of the SNP in the reference genome (IWGSC RefSeq v1.0); ^b^Phenotypic variation explained by the identified SNP; ^c^The reference for the near loci previously reported; DT19, DT20, DT21 and DAVE represented 2018 – 2019, 2019 – 2020, 2020 – 2021 and the mean value of three crop seasons for the yield-related traits under DT conditions, respectively.

**Table 4:** The QTL and significant SNPs identified by GWAS for stress tolerance index (STI).

QTL	SNP	Chr	Position (bp)^a^	Environment	*P* value	PVE (%)^b^
*qSTI-1B.1*	*AX-111503092*	1B	380,647,070	STI20, STI21, STIA	5.72E-05	3.48
	*AX-110041061*	1B	390,870,930	STI20, STI21	2.42E-04	2.88
*qSTI-1B.2*	*AX-111169510*	1B	430,155,688	STI20, STI21, STIA	1.50E-04	2.85
*qSTI-2A*	*AX-109290429*	2A	608,865,127	STI19, STI21, STIA	5.11E-04	2.39
*qSTI-4A.1*	*AX-110171894*	4A	605,753,144	STI19, STI21	2.20E-04	3.37
	*AX-108777965*	4A	605,767,873	STI19, STI21	2.56E-04	3.30
	*AX-110961937*	4A	605,785,021	STI19, STI21	2.13E-04	3.38
*qSTI-4A.2*	*AX-109375057*	4A	633,308,367	STI20, STI21, STIA	9.76E-06	4.22
	*AX-110468999*	4A	634,108,610	STI20, STI21, STIA	2.77E-05	3.78
	*AX-111140650*	4A	634,368,256	STI20, STI21, STIA	4.82E-05	3.55
	*AX-111453918*	4A	634,833,572	STI20, STI21, STIA	2.19E-05	3.88
	*AX-110440161*	4A	635,292,292	STI20, STI21, STIA	2.20E-05	3.88
	*AX-108950546*	4A	635,764,214	STI20, STI21, STIA	6.19E-06	4.41
*qSTI-4B*	*AX-111150060*	4B	660,589,159	STI19, STI21	5.86E-04	2.91
*qSTI-6A*	*AX-109334618*	6A	328,130,345	STI19, STI21, STIA	7.48E-05	2.92
*qSTI-7D.1*	*AX-108758043*	7D	547,277,450	STI20, STI21, STIA	1.52E-04	3.08
	*AX-110949705*	7D	548,055,156	STI19, STI20, STI21, STIA	1.62E-04	3.05
*qSTI-7D.2*	*AX-108770812*	7D	561,926,335	STI19, STI21, STIA	3.57E-05	3.19

^a^Physical position of the SNP in the reference genome (IWGSC RefSeq v1.0); ^b^Phenotypic variation explained by the identified SNP; STI19, STI20, STI21 and STIA represented 2018 – 2019, 2019 – 2020, 2020 – 2021 and the mean value of three crop seasons for STI, respectively.

### Thousand-grain weight

Eight QTL, represented by 18 SNPs, were detected on chromosomes 1B, 2A, 2B, 4A, 7B, and 7D for TGW under normal conditions (**Figure 4A**, **Supplementary Figures 5A–C**, **Table 2**, and **Supplementary Table 3**). One important QTL, *qNTGW-2B*, had five significant SNPs in multiple environments. *qNTGW-2B* was mapped to the physical region of 666.47 – 671.74 Mb on chromosome 2B, accounting for 2.84 – 3.40% of the phenotypic variation. Two QTL associated with TGW were detected on chromosome 4A. The first, *qNTGW-4A.1*, had four SNPs mapped to the physical position of 605.75 – 605.78 Mb and explained 2.54 – 3.44% of the phenotypic variation. The second *qNTGW-4A.2*, located at position of 633.30 – 635.76 Mb, was also represented by four SNPs and explained approximately 2.72 – 3.03% of the phenotypic variation. Besides, chromosome 7D also had two QTL (*qNTGW-7D.1* and *qNTGW-7D.2*), marked by SNPs *AX-108758043* and *AX-108770812* and explaining 2.96 and 3.23% of the phenotypic variation, respectively. In addition, three QTL, *qNTGW-1B* (430.15 Mb), *qNTGW-2A* (608.86 Mb), and *qNTGW-7B* (701.33 Mb), were represented by SNPs *AX-111169510*, *AX-109290429*, and *AX-89432708*, respectively, and collectively explained 14.70% of the phenotypic variation. Under drought treatment conditions, five QTL were associated with TGW (**Figure 4B**, **Supplementary Figures 5D–F**, **Table 3**, and **Supplementary Table 3**). Among these, *qDTGW-5A* physically was mapped to the physical region of 546.52 – 547.33 Mb on chromosome 5A. This QTL was represented by two SNPs and explained 2.33 – 3.72% of the phenotypic variation under drought treatment conditions. Another QTL, *qDTGW-5D*, at 292.94 Mb on chromosome 5D and *qDTGW-6A* at 328.13 Mb on chromosome 6A explained 2.44 and 3.08% of the phenotypic variation, respectively. Furthermore, two QTL represented by SNPs *AX-109375057* and *AX-108770812* were located on chromosomes 4A and 7D under both conditions. *qDTGW-4A* on chromosome 4A explained 3.26% of the phenotypic variation under the DT conditions, whereas *qDTGW-7D* on chromosome 7D explained 2.69% of the phenotypic variation.

#### Grain length

Under the normal conditions, 15 QTL for GL, represented by 17 SNPs, were detected on chromosomes 1B, 2A, 2D, 3B, 3D, 4B, 5B, and 5D (**Figure 4C**, **Supplementary Figures 6A–C**, **Table 2**, and **Supplementary Table 3**). *qNGL-1B.1* (565.51 Mb), *qNGL-1B.2* (580.07 Mb), and *qNGL-1B.3* (685.28 Mb) were detected on chromosome 1B, accounting for 2.30 – 2.82% of the phenotypic variation. Three QTL (*qNGL-2A*, *qNGL-3B.1*, and *qNGL-3D*) marked by *AX-109290429*, *AX-111534973*, and *AX-109392999*, were detected on chromosomes 2A, 3B, and 3D, respectively. Two QTL (*qNGL-2D.1*, *qNGL-2D.2*) were mapped to 63.56 Mb and 79.09 Mb on chromosome 2D. Besides, *qNGL-3B.2* and *qNGL-4B*, explaining 2.63 – 2.82% of the phenotypic variation in GL, were represented by two SNPs, respectively. Finally, five QTL (*qNGL-5B.1*, *qNGL-5B.2*, *qNGL-5B.3*, *qNGL-5D.1*, and *qNGL-5D.2*) located on chromosomes 5B and 5D were represented by five SNPs and accounted for 2.59 – 3.85% of the phenotypic variation.

Under drought treatment conditions, fifteen QTL associated with GL were represented by 26 significant SNPs on chromosomes 1A, 1B, 2A, 2D, 3A, 3B, 3D, 5B, 5D, 6A, 7A, and 7D (**Figure 4D**, **Supplementary Figures 6D–F**, **Table 3**, and **Supplementary Table 3**). One of the 15, *qDGL-1A*, was detected at the physical position of 503.44 – 504.00 Mb on chromosome 1A. Two SNPs represented this QTL, collectively explaining 4.69% of the phenotypic variation. Seven of these QTL, including *qDGL-2A.2* (693.33 Mb), *qDGL-2D* (63.56 Mb), *qDGL-3B* (785.43 Mb), *qDGL-3D* (599.68 Mb), *qDGL-5B.1* (57.49 Mb), *qDGL-5D.1* (30.85 Mb) and *qDGL-5D.2* (379.83 Mb) were represented by various SNPs, which accounted for 2.33 – 3.97% of the phenotypic variation. *qDGL-7A* and *qDGL-7D* are located on chromosomes 7A and 7D, represented by five SNPs, accounting for 2.75 – 5.50% of the phenotypic variation. *qDGL-1B* was located at the physical position of 686.75 – 686.78 Mb on chromosome 1B, and *qDGL-5B.2* was at the physical position of 699.99 – 702.22 Mb on chromosome 5B. These two QTL, which explained 2.23 – 3.17% of the phenotypic variation, were represented by three SNPs. Three QTL (*qDGL-2A.1*, *qDGL-3A*, and *qDGL-6A*) represented by two SNPs were mapped on chromosomes 2A, 3A, and 6A, respectively, explaining 2.73 – 4.14% of the phenotypic variation. Additionally, six SNPs, including *AX-109290429* (on chromosome 2A), *AX-109013500* (on chromosome 2D), *AX-110418888* (on chromosome 3B), *AX-110398218* (on chromosome 5B), *AX-108733256* (on chromosome 5B), and *AX-110503408* (on chromosome 5D) were detected under NC and DT conditions. Most notably, *AX-109290429* was associated with TGW.NC, GL.NC, and GL.DT.

### Grain width

Under the normal conditions, one QTL, *qNGW-4A*, was detected at 38.36 Mb on chromosome 4A and accounted for 2.92% of the phenotypic variation (**Figure 5A**, **Supplementary Figures 7A–C**, **Table 2**, and **Supplementary Table 3**). By contrast, the DT conditions revealed two QTL represented by three SNPs on chromosome 6A (**Figure 5B**, **Supplementary Figures 7D–F**, **Table 3**, and **Supplementary Table 3**). The first QTL *qDGW-6A.1*, located at 148.52 – 158.06 Mb, was represented by two SNPs and explained 3.70 – 3.82% of the total phenotypic variation. The second QTL, *qDGW-6A.2*, represented by SNP *AX-110942969*, was mapped to the physical position of 428.45 Mb and explained 3.71% of the phenotypic variation.

### Spike length

For SL, one QTL, *qNSL-6B*, represented by *AX-109337721*, was mapped to 528.70 Mb on chromosome 6B and explained 3.15% of the total phenotypic variation under the normal conditions (**Figure 5C**, **Supplementary Figures 8A–C**,**Table 2**, and **Supplementary Table 3**). In contrast, we detected four QTL represented by four SNPs on chromosomes 2A, 2D, 3D, and 5B under the DT conditions (**Figure 5D**, **Supplementary Figures 8D–F**, **Table 3**, and **Supplementary Table 3**). The first QTL, *qDSL-2A*, represented by *AX-108747720*, was marked to chromosome 2A and explained 4.01% of the phenotypic variation. The second QTL, *qDSL-2D*, detected on chromosome 2D (at 23.02 Mb), was represented by *AX-110647062* and explained 6.31% of phenotypic variation in SL. The last two QTL (*qDSL-3D* and *qDSL-5B*) explained 2.70 and 2.63% of the phenotypic variation, respectively.

### Stress tolerance index

For STI, nine high confidence QTL were detected under more than one environment. These QTL involved 18 SNPs distributed on six chromosomes (including 1B, 2A, 4A, 4B, 6A and 7D) (**Figure 5E**, **Supplementary Figure 9**, **Table 4**, and **Supplementary Table 4**). Five of these QTL, including *qSTI-1B.2* (430.15 Mb), *qSTI-2A* (608.86 Mb), *qSTI-4B* (660.58 Mb), *qSTI-6A* (328.13 Mb) and *qSTI-7D.2* (561.92 Mb) were represented by various SNPs, which accounted for 2.39 – 3.19% of the phenotypic variation. In addition, *qSTI-1B.1* was located at 380.64 – 390.87 Mb on chromosome 1B, and *qSTI-7D.1* was detected at 547.27 – 548.05 Mb on chromosome 7D. These two QTL, which explained 2.88 – 3.48% of the phenotypic variation, were represented by two SNPs, respectively. Another two QTL (*qSTI-4A.1* and *qSTI-4A.2*) represented by nine SNPs on chromosome 4A totally explained 33.76% of the phenotypic variation. Notably, twelve SNPs (including *AX-111169510*, *AX-109290429*, *AX-110171894*, *AX-108777965*, *AX-110961937*,*AX-109375057*, *AX-110468999*, *AX-111453918*, *AX-108950546*, *AX-109334618*, *AX-108758043* and *AX-108770812*) had association with STI as well as TGW.

#### Additive effects of the superior alleles on phenotypes

**Figure 6:**
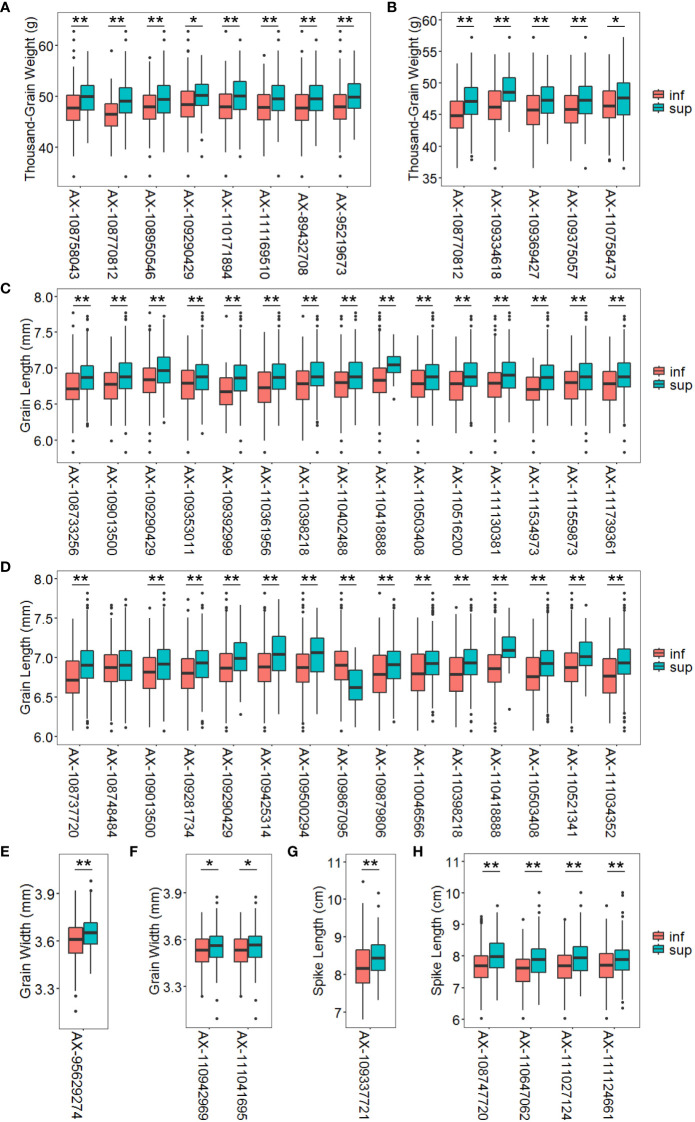
Average phenotypic values of accessions with different alleles in multi-environment significant loci under normal and drought treatment conditions for thousand-grain weight **(A, B)**, grain length **(C, D)**, grain width **(E, F)** and spike length **(G, H)**. The *P* value was determined by the two tailed Student’s *t*-test. *P < 0.05, **P < 0.01. Inf represents the inferior allele and sup represents the superior allele.

**Figure 7:**
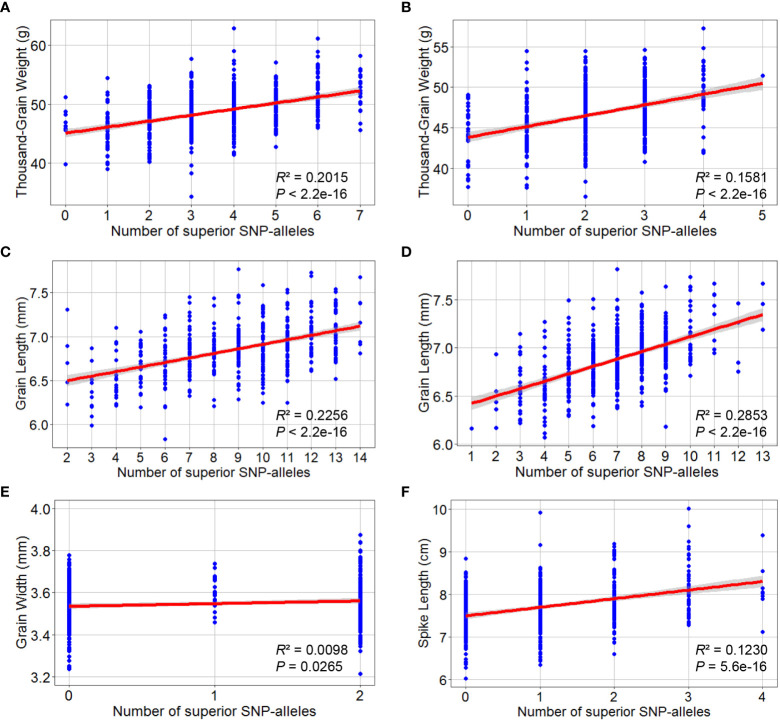
Superior allelic effects of the highest -log_10_ (*P*) SNPs by GWAS for thousand-grain weight, grain length, grain width and spike length. Thousand-grain weight under normal **(A)** and drought treatment **(B)** conditions; Grain length under normal **(C)** and drought treatment **(D)** conditions; **(E)** Grain width under drought treatment condition; **(F)** Spike length under drought treatment condition. At the bottom of the box plot corresponding to each SNP allele.

The marker alleles associated with high TGW, GL, GW, and SL were considered “superior alleles”, and the marker alleles with the opposite effects as “inferior alleles” to ease the description of allelic effects. The allelic effect was verified using the highest -log_10_ (*P*) SNPs within the detected QTL in the association population by examining whether the differences in phenotypic values (grouped by polymorphism) reached a significant level (**Figure 6**). The number of superior alleles in each wheat line was calculated based on representative SNPs for the loci detected using the average values for grain and spike traits across all environments. The patterns of relationship were similar for the detected traits, where superior alleles additively increased TGW.NC (*R*^2^ = 0.2015), TGW.DT (*R*^2^ = 0.1581), GL.NC (*R*^2^ = 0.2256), GL.DT (*R*^2^ = 0.2853), GW.DT (*R*^2^ = 0.0098) and SL.DT (*R*^2^ = 0.1230) (**Figure 7**).

#### KASP development

Three significant SNP markers (*AX-109290429*, *AX-110418888*, and *AX-109369427*) on chromosomes 2A, 3B, and 5A were identified in multiple environments were converted into KASP markers. The KASP markers could accurately classify different alleles. The results showed that different alleles carrying FAM and HEX fluorescence were distributed around the X and Y axes, respectively (**Supplementary Figure 10**). These KASP marker-based allelic typing results were consistent with those on the 15K SNP array.

## Discussion

### The drought stress reduces wheat yields

Wheat is particularly vulnerable to drought stress during growth. This study investigated the effects of drought stress on yield-related traits of wheat. Under the drought treatment conditions, TGW, GW, and SL were reduced by 4, 2, and 6% compared to the normal conditions (**Figure 1** and **Table 1**). However, the broad sense heritability of TGW, GL, GW, and SL was relatively high (76.16 – 95.66%) under both conditions, consistent with previous studies (Sukumaran et al., 2018; Wolde et al., 2019; Ji et al., 2021; Wang et al., 2021; Wang et al., 2022). Furthermore, the association panel showed significant phenotypic variation in TGW, GL, GW, and SL under normal and drought conditions. These results highlight the potential of these germplasms to identify the alleles associated with plant adaption to harsh environments for improving elite wheat yield.

### Identifying and comparing SNPs with previous findings

The association results were validated using MLM, which considers population structure and kinship to define the significant SNPs in over two environments under normal and drought conditions. Therefore, 37 and 39 significant SNPs (in 51 QTL) were considered reliable MTAs in multiple environments associated with the yield-related traits under NC and DT conditions (**Tables 2**, **3**). Eighteen MTAs (in nine QTL) associated with STI were identified in more than one environment (**Table 4**). Furthermore, we identified the QTL for the stability of grain and spike traits by comparing the MTAs detected under normal and drought treatment conditions simultaneously with those detected only under normal conditions.

### Normal conditions

We observed 18 credible SNPs associated with TGW, representing eight loci mapped onto chromosomes 1BL, 2AL, 2BL, 4AL, 7BL, and 7DL under normal conditions. One SNP cluster (consisting of five SNPs) was identified on chromosome 2BL, spanning the 5.27 Mb (2B: 666.47 – 671.74 Mb) physical position. This locus was located on the same genomic region containing previously reported QTL for TGW (Groos et al., 2003). In addition, two adjacent major QTL represented by eight SNPs were detected on chromosome 4AL, explaining 23.44% of the TGW variance. One QTL, *qNTGW-4A.1*, accounted for 11.96% of the phenotypic variation, consistent with a previous study (Zheng et al., 2010). Moreover, the QTL was adjacent to the yield-associated gene, *TaCWI-4A*, in the Jiang consensus map (Jiang et al., 2015). Another QTL, *qNTGW-4A.2*, located at the 633.30 – 635.76 Mb interval on chromosome 4A, was adjacent to other QTL for TGW, as previously indicated (Gao et al., 2015; Cui et al., 2016; Guan et al., 2018). In addition, *qNTGW-2A*, *qNTGW-7B*, and *qNTGW-7D.2* associated with TGW were detected on chromosomes 2AL, 7BL, and 7DL, respectively (Mccartney et al., 2005; Li et al., 2007; Zhang et al., 2014a; Gao et al., 2015; Cao et al., 2020). Although previous studies also identified several QTL controlling TGW on chromosome 1B (Zheng et al., 2010; Wang et al., 2012; Cui et al., 2014; Sukumaran et al., 2018; Qu et al., 2021), the QTL were inconsistent with *qNTGW-1B* detected in this study, suggesting that *qNTGW-1B*, reported for the first time in the current study was a novel QTL.

A total of 15 QTL containing 17 SNPs associated with GL were detected on chromosomes 1B, 2A, 2D, 3B, 3D, 4B, 5B, and 5D under normal conditions. Among these loci, *qNGL-3B.2*, associated with GL, was marked at 785.43 – 793.05 Mb on chromosome 3BL. Previous studies have also identified QTL that controls GL at adjacent positions (Kumar et al., 2016). We identified three QTL on chromosome 5B, which explained 9.34% of phenotypic variation in GL. The first QTL, *qNGL-5B.1*, on chromosome 5BS was mapped at a similar position with *Qgl.caas-5BS*, associated with GL (Yang et al., 2020). The second QTL was located at the neighboring chromosomal region of the grain-length-related QTL, *QKL.caas-5BS*, detected in the Chinese bread wheat recombinant inbred population (Li et al., 2018). The third QTL, *qNGL-5B.3*, on chromosome 5BL shared a similar region with *QKl5B.2-16* for GL (Li et al., 2015). Notably, the SNP *AX-109290429* on chromosome 2AL was significantly associated with GL, TGW and STI, suggesting that this locus is highly valuable for these traits. Position-based comparisons of the SNPs identified in this study with previous reports identified another ten new and preliminary loci associated with GL on chromosomes 1B, 2D, 3B, 3D, 4B, and 5D.

Several previous studies also identified QTL for GW, such as *qKW-4A* (Su et al., 2018), *QKW.ndsu.4A* (Kumar et al., 2016), *QKw-4A.1* (Cui et al., 2016), *QKW.caas-4AS* (Li et al., 2018), and *QKW.caas-4AL* (Li et al., 2018) on chromosome 4A. This study also detected *qNGW-4A* for GW on chromosome 4AS under normal conditions, similar with the position identified by Li et al. (Li et al., 2018).

Moreover, there were several QTL for SL detected on chromosome 6B in the previous studies (Liu et al., 2014; Gao et al., 2015; Deng et al., 2017; Li et al., 2018; Cao et al., 2019). We also detected a credible QTL at the physical position of 528.70 Mb on chromosome 6B. However, this QTL for SL was not previously reported, suggesting that it may be new.

### Drought treatment conditions

The SNPs detected under DT differed from those under the normal conditions (**Table 3** and **Supplementary Table 3**) owing to the effects of the drought stress. We also detected five QTL represented by six SNPs for TGW under DT. Most notably, two of these SNPs (*AX-109375057* and *AX-108770812*) were associated with TGW.NC, TGW.DT and STI, suggesting that these SNPs’ effects on TGW and STI were extremely important and unaffected by environmental changes (**Tables 2**–**4** and **Supplementary Tables 3**, **4**). Moreover, the QTL *qDTGW-5A* for TGW was located at adjacent chromosomal regions (Li et al., 2018; Cao et al., 2020). Furthermore, *qDTGW-6A* was marked at an approximately equivalent chromosomal region in a previous study (Wang et al., 2011). Comparisons between *qDTGW-5D* discovered in this research with other QTL identified in previous studies showed that *qDTGW-5D*, located on chromosome 5D, was a newly detected QTL regulating TGW.

For GL, nine QTL were detected under DT conditions, and six others under NC and DT conditions. Previously, *qDGL-7A* for GL was only detected under DT conditions in the region near SNP S7A_717859384, associated with GL (Gill et al., 2022). For GW, we detected two QTL on chromosome 6A under DT. These QTL were marked at a near location similar with a previously detected QTL for GW (Li et al., 2022b). We discovered four QTL for SL on chromosome 2AS, 2DS, 3DL, and 5BL under DT. Of these, *qDSL-2D, qDSL-3D*, and *qDSL-5B* were located at similar regions with QTL for SL from previous studies (Ma et al., 2007; Heidari et al., 2011; Wang et al., 2011; Wu et al., 2014; Yu et al., 2014; Deng et al., 2017; Chai et al., 2019; Gill et al., 2022; Li et al., 2022a). Notably, *qDSL-2A* for SL was first discovered in the current study by comparing this study with previous research.

A total of nine high confidence QTL for STI were detected, out of which, seven QTL (*qDRI-1B.2*, *qDRI-2A*, *qDRI-4A.1*, *qDRI-4A.2*, *qDRI-6A*, *qDRI-7D.1*, *qDRI-7D.2*) were co-located with QTL for TGW under NC or DT conditions. Therefore, these seven co-located QTL can be used to synergistically improve the yield and drought resistance of wheat. To the best of our knowledge, only a few QTL for STI, which was calculated from TGW, have been detected in wheat (Negisho et al., 2022). Nevertheless, they were located at different chromosomal regions with these QTL for STI detected in this study.

Many agronomic traits are complex quantitative traits that are susceptible to environmental factors. Therefore, this study detected eight stable MTAs (two for TGW and STI, six for GL) under normal and drought conditions. The MTAs detected are important for both normal and drought treatment conditions, providing insights into the genetic basis of the stability of agronomic traits. These MTAs can be used to develop useful markers for genetic improvement in wheat breeding.

### Validation of multi-environment significant SNPs in the association population

This study identified numerous SNPs closely associated with TGW, GL, GW, SL and STI. Among these, three SNP markers were developed as KASP markers, making the genotype identification process more efficient, inexpensive, fast, convenient, and greatly accelerating the process of molecular marker-assisted selection. Under normal conditions, accessions with superior alleles for TGW at eight SNP loci showed TGW values of 49.29 to 50.17 g. In contrast, accessions with inferior alleles at these loci showed TGW values of 46.45 – 48.54 g. Under DT conditions, accessions with superior alleles for TGW at five SNP loci showed TGW values of 47.36 – 49.03 g compared to 44.93 – 46.55 g for accessions with inferior alleles. Nevertheless, the frequency of superior alleles of SNP *AX-109334618* on chromosome 6A was only about 5%, indicating that this SNP has not been fully utilized. Furthermore, this SNP was associated with not only TGW but also STI, suggesting that it is worth considering as a major genetic locus for improving TGW and drought resistance (**Figures 6A, B**).

Under normal conditions, accessions with superior alleles for GL at 15 SNP loci showed significantly higher GL values (6.87 – 7.07 mm) than those with inferior alleles (6.68 – 6.84 mm). Moreover, under DT conditions, the GL values of accessions with superior alleles for this trait at 15 SNP loci ranged from 6.89 to 7.12 mm compared to 6.64 – 6.87 mm for accessions with inferior alleles at the same loci. However, superior alleles at some loci, such as *AX-109290429*, *AX-109425314*, *AX-110418888*, *AX-109500294*, and *AX-110521341*, were present in < 12% of the accessions, suggesting that these loci require more attention when studying the improvement of GL (**Figures 6C, D**). Under normal conditions, accessions with a superior allele at *AX-95629274* showed 3.65 mm GW values than the 3.60 mm for accessions with an inferior allele at this locus. Furthermore, accessions with superior alleles at two SNP loci showed higher GW values (~3.53 mm) than accessions with inferior alleles (~3.56 mm) under DT conditions (**Figures 6E, F**).

However, accessions with the superior allele of SNP *AX-109337721* showed significantly higher SL values (~8.17 cm) than those with the inferior allele (~8.52 cm) under normal conditions. Notably, the superior allele of this SNP was < 14% frequent among accessions, indicating that this SNP is underutilized and a probably significant genetic locus for improving SL. Finally, the SL values of accessions with superior alleles for this trait at the four SNP loci were 7.88 – 8.01 cm compared to 7.55 – 7.70 cm for accessions with inferior alleles under DT conditions. Nevertheless, the frequencies of superior alleles for *AX-108747720*, *AX-111027124*, and *AX-111124661* were < 26%, indicating these loci require more attention when improving SL (**Figures 6G, H**).

The TGW of the detected varieties from different decades increased with increasing frequency of superior alleles under both conditions. Furthermore, increased TGW was strongly selected for breeding before the 2010s. However, after 2020, the average TGW of the accessions in this study decreased slightly, as did the corresponding number of superior alleles in both conditions (**Supplementary Figure 11**). Altogether, the elite alleles at these loci detected under two conditions can be integrated into wheat varieties using molecular breeding to improve wheat yield.

## Conclusion

In summary, this study identified high confidence loci for yield-related and drought-resistance-related traits in normal and drought-stressed common wheat by GWAS and developed three KASP markers associated with these traits, which is a major step to accelerate the breeding of high-yielding and drought-resistant varieties.

## Data availability statement

The original contributions presented in the study are included in the article/**Supplementary Material.** Further inquiries can be directed to the corresponding author.

## Author contributions

HL and YJZ conceived and designed the experiments. JZ, LS and HG performed most of the experiments and wrote the manuscript. MH, LM, XC and JW provided technical assistance and conducted the collection and maintenance of wheat germplasm. YZ, QL and PW involved in discussion and participated in field trials. All authors contributed to the article and approved the submitted version.

## References

[B1] Ahmed H. G. M. Zeng Y. Shah A. N. Yar M. M. Ullah A. Ali M. (2022). Conferring of drought tolerance in wheat (*Triticum aestivum* L.) genotypes using seedling indices. Front. Plant Sci. 13, 961049. doi: 10.3389/fpls.2022.961049 35937360 PMC9355593

[B2] Bradbury P. J. Zhang Z. Kroon D. E. Casstevens T. M. Ramdoss Y. Buckler E. S. (2007). TASSEL: software for association mapping of complex traits in diverse samples. Bioinformatics 23, 2633–2635. doi: 10.1093/bioinformatics/btm308 17586829

[B3] Cao P. Liang X. Zhao H. Feng B. Xu E. Wang L.. (2019). Identification of the quantitative trait loci controlling spike-related traits in hexaploid wheat (*Triticum aestivum* L.). Planta 250, 1967–1981. doi: 10.1007/s00425-019-03278-0 31529397

[B4] Cao J. Liu K. Song W. Zhang J. Yao Y. Xin M.. (2021). Pleiotropic function of the *SQUAMOSA PROMOTER-BINDING PROTEIN-LIKE* gene *TaSPL14* in wheat plant architecture. Planta 253, 44. doi: 10.1007/s00425-020-03531-x 33481116 PMC7822796

[B5] Cao S. Xu D. Hanif M. Xia X. He Z. (2020). Genetic architecture underpinning yield component traits in wheat. Theor. Appl. Genet. 133, 1811–1823. doi: 10.1007/s00122-020-03562-8 32062676

[B6] Chai L. Chen Z. Bian R. Zhai H. Cheng X. Peng H.. (2019). Dissection of two quantitative trait loci with pleiotropic effects on plant height and spike length linked in coupling phase on the short arm of chromosome 2D of common wheat (*Triticum aestivum* L.). Theor. Appl. Genet. 132, 1815–1831. doi: 10.1007/s00122-019-03318-z 30915484 PMC6531420

[B7] Chang J. Zhang J. Mao X. Li A. Jia J. Jing R. (2013). Polymorphism of *TaSAP1-A1* and its association with agronomic traits in wheat. Planta 237, 1495–1508. doi: 10.1007/s00425-013-1860-x 23462884

[B8] Cui F. Fan X. Chen M. Zhang N. Zhao C. Zhang W.. (2016). QTL detection for wheat kernel size and quality and the responses of these traits to low nitrogen stress. Theor. Appl. Genet. 129, 469–484. doi: 10.1007/s00122-015-2641-7 26660466

[B9] Cui F. Zhao C. Ding A. Li J. Wang L. Li X.. (2014). Construction of an integrative linkage map and QTL mapping of grain yield-related traits using three related wheat RIL populations. Theor. Appl. Genet. 127, 659–675. doi: 10.1007/s00122-013-2249-8 24326459

[B10] Deng Z. Cui Y. Han Q. Fang W. Li J. Tian J. (2017). Discovery of consistent QTLs of wheat spike-related traits under nitrogen treatment at different development stages. Front. Plant Sci. 8, 2120. doi: 10.3389/fpls.2017.02120 29326735 PMC5737097

[B11] Dong L. Wang F. Liu T. Dong Z. Li A. Jing R.. (2014). Natural variation of *TaGASR7-A1* affects grain length in common wheat under multiple cultivation conditions. Mol. Breed. 34, 937–947. doi: 10.1007/s11032-014-0087-2

[B12] Fernandez G. C. J. (1992). “Effective selection criteria for assessing plant stress tolerance,” in Proceedings of the International Symposium on Adaptation of Vegetables and Other Food Crops in Temperature and Water Stress. Ed. Kuo C. G. (Tainan, Taiwan).

[B13] Gao F. Wen W. Liu J. Rasheed A. Yin G. Xia X.. (2015). Genome-wide linkage mapping of QTL for yield components, plant height and yield-related physiological traits in the Chinese wheat cross zhou 8425B/Chinese spring. Front. Plant Sci. 6, 1099. doi: 10.3389/fpls.2015.01099 26734019 PMC4683206

[B14] Gill H. S. Halder J. Zhang J. Rana A. Kleinjan J. Amand P. S.. (2022). Whole-genome analysis of hard winter wheat germplasm identifies genomic regions associated with spike and kernel traits. Theor. Appl. Genet. 135, 2953–2967. doi: 10.1007/s00122-022-04160-6 35939073

[B15] Groos C. Robert N. Bervas E. Charmet G. (2003). Genetic analysis of grain protein-content, grain yield and thousand-kernel weight in bread wheat. Theor. Appl. Genet. 106, 1032–1040. doi: 10.1007/s00122-002-1111-1 12671751

[B16] Guan P. Lu L. Jia L. Kabir M. R. Zhang J. Zhao Y.. (2018). Global QTL analysis identifies genomic regions on chromosomes 4A and 4B harboring stable loci for yield-related traits across different environments in wheat (*Triticum aestivum* L.). Front. Plant Sci. 9, 529. doi: 10.3389/fpls.2018.00529 29922302 PMC5996883

[B17] Guo X. Fu Y. Lee Y. J. Chern M. Li M. Cheng M.. (2022). The PGS1 basic helix-loop-helix (bHLH) protein regulates *Fl3* to impact seed growth and grain yield in cereals. Plant Biotechnol. J. 20, 1311–1326. doi: 10.1111/pbi.13809 35315196 PMC9241376

[B18] Hanif M. Gao F. Liu J. Wen W. Zhang Y. Rasheed A.. (2016). *TaTGW6-A1*, an ortholog of rice *TGW6*, is associated with grain weight and yield in bread wheat. Mol. Breed. 36, 1. doi: 10.1007/s11032-015-0425-z

[B19] Hayes B. (2013). Overview of statistical methods for genome-wide association studies (GWAS). Methods Mol. Biol. 1019, 149–169. doi: 10.1007/978-1-62703-447-0_6 23756890

[B20] Heidari B. Sayed-Tabatabaei B. E. Saeidi G. Kearsey M. Suenaga K. (2011). Mapping QTL for grain yield, yield components, and spike features in a doubled haploid population of bread wheat. Genome 54, 517–527. doi: 10.1139/g11-017 21635161

[B21] Hong Y. Chen L. Du L. P. Su Z. Wang J. Ye X.. (2014). Transcript suppression of *TaGW2* increased grain width and weight in bread wheat. Funct. Integr. Genomics 14, 341–349. doi: 10.1007/s10142-014-0380-5 24890396

[B22] Hu M. J. Zhang H. P. Liu K. Cao J. J. Wang S. X. Jiang H.. (2016). Cloning and characterization of *TaTGW-7A* gene associated with grain weight in wheat *via* SLAF-seq-BSA. Front. Plant Sci. 7, 1902. doi: 10.3389/fpls.2016.01902 28066462 PMC5167734

[B23] Jia M. Li Y. Wang Z. Tao S. Sun G. Kong X.. (2021). TaIAA21 represses TaARF25-mediated expression of *TaERFs* required for grain size and weight development in wheat. Plant J. 108, 1754–1767. doi: 10.1111/tpj.15541 34643010

[B24] Jiang Y. Jiang Q. Hao C. Hou J. Wang L. Zhang H.. (2015). A yield-associated gene *TaCWI*, in wheat: Its function, selection and evolution in global breeding revealed by haplotype analysis. Theor. Appl. Genet. 128, 131–143. doi: 10.1007/s00122-014-2417-5 25367379

[B25] Ji G. Xu Z. Fan X. Zhou Q. Yu Q. Liu X.. (2021). Identification of a major and stable QTL on chromosome 5A confers spike length in wheat (*Triticum aestivum* L.). Mol. Breed. 41, 56. doi: 10.1007/s11032-021-01249-6 PMC10236030 37309397

[B26] Juliana P. Govindan V. Crespo-Herrera L. Mondal S. Huerta-Espino J. Shrestha S.. (2022). Genome-wide association mapping identifies key genomic regions for grain zinc and iron biofortification in bread wheat. Front. Plant Sci. 13, 903819. doi: 10.3389/fpls.2022.903819 35845653 PMC9280339

[B27] Kang Y. Barry K. Cao F. Zhou M. (2020). Genome-wide association mapping for adult resistance to powdery mildew in common wheat. Mol. Boil. Rep. 47, 1241–1256. doi: 10.1007/s11033-019-05225-4 31813131

[B28] Kumar A. Mantovani E. E. Seetan R. Soltani A. Echeverry-Solarte M. Jain S.. (2016). Dissection of genetic factors underlying wheat kernel shape and size in an elite × nonadapted cross using a high density SNP linkage map. Plant Genome 9, 1–22. doi: 10.3835/plantgenome2015.09.0081 27898771

[B29] Li T. Deng G. Su Y. Yang Z. Tang Y. Wang J.. (2022b). Genetic dissection of quantitative trait loci for grain size and weight by high-resolution genetic mapping in bread wheat (*Triticum aestivum* L.). Theor. Appl. Genet. 135, 257–271. doi: 10.1007/s00122-021-03964-2 34647130

[B30] Li A. Hao C. Wang Z. Geng S. Jia M. Wang F.. (2022a). Wheat breeding history reveals synergistic selection of pleiotropic genomic sites for plant architecture and grain yield. Mol. Plant 15, 504–519. doi: 10.1016/j.molp.2022.01.004 35026438

[B31] Li S. Jia J. Wei X. Zhang X. Li L. Chen H.. (2007). A intervarietal genetic map and QTL analysis for yield traits in wheat. Mol. Breed. 20, 167–178. doi: 10.1007/s11032-007-9080-3

[B32] Liu G. Jia L. Lu L. Qin D. Zhang J. Guan P.. (2014). Mapping QTLs of yield-related traits using RIL population derived from common wheat and Tibetan semi-wild wheat. Theor. Appl. Genet. 127, 2415–2432. doi: 10.1007/s00122-014-2387-7 25208643

[B33] Liu H. Li H. Hao C. Wang K. Wang Y. Qin L.. (2020). *TaDA1*, a conserved negative regulator of kernel size, has an additive effect with *TaGW2* in common wheat (*Triticum aestivum* L.). Plant Biotechnol. J. 18, 1330–1342. doi: 10.1111/pbi.13298 31733093 PMC7152612

[B34] Li F. Wen W. He Z. Liu J. Jin H. Cao S.. (2018). Genome-wide linkage mapping of yield-related traits in three Chinese bread wheat populations using high-density SNP markers. Theor. Appl. Genet. 131, 1903–1924. doi: 10.1007/s00122-018-3122-6 29858949

[B35] Li F. Wen W. Liu J. Zhang Y. Cao S. He Z.. (2019). Genetic architecture of grain yield in bread wheat based on genome-wide association studies. BMC Plant Biol. 19, 168. doi: 10.1186/s12870-019-1781-3 31035920 PMC6489268

[B36] Li Q. Zhang Y. Liu T. Wang F. Liu K. Chen J.. (2015). Genetic analysis of kernel weight and kernel size in wheat (*Triticum aestivum* L.) using unconditional and conditional QTL mapping. Mol. Breed. 35, 194. doi: 10.1007/s11032-015-0384-4

[B37] Li L. Zhang Y. Zhang Y. Li M. Xu D. Tian X.. (2021). Genome-wide linkage mapping for preharvest sprouting resistance in wheat using 15K single-nucleotide polymorphism arrays. Front. Plant Sci. 12, 749206. doi: 10.3389/fpls.2021.749206 34721477 PMC8551680

[B38] Ma L. Li T. Hao C. Wang Y. Chen X. Zhang X. (2016). *TaGS5-3A*, a grain size gene selected during wheat improvement for larger kernel and yield. Plant Biotechnol. J. 14, 1269–1280. doi: 10.1111/pbi.12492 26480952 PMC11389196

[B39] Ma M. Wang Q. Li Z. Cheng H. Li Z. Liu X.. (2015). Expression of *TaCYP78A3*, a gene encoding cytochrome P450 CYP78A3 protein in wheat (*Triticum aestivum* L.), affects seed size. Plant J. 83, 312–325. doi: 10.1111/tpj.12896 26043144

[B40] Ma D. Yan J. He Z. Wu L. Xia X. (2012). Characterization of a cell wall invertase gene *TaCwi-A1* on common wheat chromosome 2A and development of functional markers. Mol. Breed. 29, 43–52. doi: 10.1007/s11032-010-9524-z

[B41] Ma Z. Zhao D. Zhang C. Zhang Z. Xue S. Lin F.. (2007). Molecular genetic analysis of five spike-related traits in wheat using RIL and immortalized F_2_ populations. Mol. Genet. Genomics 277, 31–42. doi: 10.1007/s00438-006-0166-0 17033810

[B42] Mccartney C. A. Somers D. J. Humphreys D. G. Lukow O. Ames N. Noll J.. (2005). Mapping quantitative trait loci controlling agronomic traits in the spring wheat cross RL4452 × 'AC domain'. Genome 48, 870–883. doi: 10.1139/g05-055 16391693

[B43] Negisho K. Shibru S. Matros A. Pillen K. Ordon F. Wehner G. (2022). Association mapping of drought tolerance indices in Ethiopian durum wheat (*Triticum turgidum* ssp. durum). Front. Plant Sci. 13, 838088. doi: 10.3389/fpls.2022.838088 35693182 PMC9178276

[B44] Nordborg M. Weigel D. (2008). Next-generation genetics in plants. Nature 456, 720–723. doi: 10.1038/nature07629 19079047

[B45] Pang Y. Liu C. Wang D. St Amand P. Bernardo A. Li W.. (2020). High-resolution genome-wide association study identifies genomic regions and candidate genes for important agronomic traits in wheat. Mol. Plant 13, 1311–1327. doi: 10.1016/j.molp.2020.07.008 32702458

[B46] Purcell S. Neale B. Todd-Brown K. Thomas L. Ferreira M. A. Bender D.. (2007). PLINK: A tool set for whole-genome association and population-based linkage analyses. Am. J. Hum. Genet. 81, 559–575. doi: 10.1086/519795 17701901 PMC1950838

[B47] Qu P. Wang J. Wen W. Gao F. Liu J. Xia X.. (2021). Construction of consensus genetic map with applications in gene mapping of wheat (*Triticum aestivum* L.) using 90K SNP array. Front. Plant Sci. 12, 727077. doi: 10.3389/fpls.2021.727077 34512703 PMC8424075

[B48] Sajjad M. Ma X. Habibullah Khan S. Shoaib M. Song Y. Yang W.. (2017). *TaFlo2-A1*, an ortholog of rice *Flo2*, is associated with thousand grain weight in bread wheat (*Triticum aestivum* L.). BMC Plant Biol. 17, 164. doi: 10.1186/s12870-017-1114-3 29037166 PMC5644068

[B49] Shan D. Ali M. Shahid M. Arif A. Waheed M. Q. Xia X.. (2022). Genetic networks underlying salinity tolerance in wheat uncovered with genome-wide analyses and selective sweeps. Theor. Appl. Genet. 135, 2925–2941. doi: 10.1007/s00122-022-04153-5 35915266

[B50] Sukumaran S. Lopes M. Dreisigacker S. Reynolds M. (2018). Genetic analysis of multi-environmental spring wheat trials identifies genomic regions for locus-specific trade-offs for grain weight and grain number. Theor. Appl. Genet. 131, 985–998. doi: 10.1007/s00122-017-3037-7 29218375

[B51] Sun H. Zhang W. Wu Y. Gao L. Cui F. Zhao C.. (2020). The circadian clock gene, *TaPRR1*, is associated with yield-related traits in wheat (*Triticum aestivum* L.). Front. Plant Sci. 11, 285. doi: 10.3389/fpls.2020.00285 32226438 PMC7080851

[B52] Su Q. Zhang X. Zhang W. Zhang N. Song L. Liu L.. (2018). QTL detection for kernel size and weight in bread wheat (*Triticum aestivum* L.) using a high-density SNP and SSR-based linkage map. Front. Plant Sci. 9, 1484. doi: 10.3389/fpls.2018.01484 30364249 PMC6193082

[B53] Tshikunde N. M. Mashilo J. Shimelis H. Odindo A. (2019). Agronomic and physiological traits, and associated quantitative trait loci (QTL) affecting yield response in wheat (*Triticum aestivum* L.): A review. Front. Plant Sci. 10, 1428. doi: 10.3389/fpls.2019.01428 31749826 PMC6848381

[B54] Wang L. Ge H. Hao C. Dong Y. Zhang X. (2012). Identifying loci influencing 1,000-kernel weight in wheat by microsatellite screening for evidence of selection during breeding. PloS One 7, e29432. doi: 10.1371/journal.pone.0029432 22328917 PMC3273457

[B55] Wang X. Guan P. Xin M. Wang Y. Chen X. Zhao A.. (2021). Genome-wide association study identifies QTL for thousand grain weight in winter wheat under normal- and late-sown stressed environments. Theor. Appl. Genet. 134, 143–157. doi: 10.1007/s00122-020-03687-w 33030571

[B56] Wang Y. Hou J. Liu H. Li T. Wang K. Hao C.. (2019). *TaBT1*, affecting starch synthesis and thousand kernel weight, underwent strong selection during wheat improvement. J. Exp. Bot. 70, 1497–1511. doi: 10.1093/jxb/erz032 30753656 PMC6411380

[B57] Wang J. Liu W. Wang H. Li L. Wu J. Yang X.. (2011). QTL mapping of yield-related traits in the wheat germplasm 3228. Euphytica 177, 277–292. doi: 10.1007/s10681-010-0267-z

[B58] Wang P. Tian T. Ma J. Liu Y. Zhang P. Chen T.. (2022). Genome-wide association study of kernel traits using a 35K SNP array in bread wheat (*Triticum aestivum* L.). Front. Plant Sci. 13, 905660. doi: 10.3389/fpls.2022.905660 35734257 PMC9207461

[B59] Wang S. Yan X. Wang Y. Liu H. Cui D. Chen F. (2016). Haplotypes of the *TaGS5-A1* gene are associated with thousand-kernel weight in Chinese bread wheat. Front. Plant Sci. 7, 783. doi: 10.3389/fpls.2016.00783 27375643 PMC4891348

[B60] Wang J. Zhang Z. (2021). GAPIT version 3: boosting power and accuracy for genomic association and prediction. Genomics Proteomics Bioinf. 19, 629–640. doi: 10.1016/j.gpb.2021.08.005 PMC9121400 34492338

[B61] Wolde G. M. Trautewig C. Mascher M. Schnurbusch T. (2019). Genetic insights into morphometric inflorescence traits of wheat. Theor. Appl. Genet. 132, 1661–1676. doi: 10.1007/s00122-019-03305-4 30762083 PMC6531419

[B62] Wu X. Cheng R. Xue S. Kong Z. Wan H. Li G.. (2014). Precise mapping of a quantitative trait locus interval for spike length and grain weight in bread wheat (*Triticum aestivum* L.). Mol. Breed. 33, 129–138. doi: 10.1007/s11032-013-9939-4

[B63] Yang L. Zhao D. Meng Z. Xu K. Yan J. Xia X.. (2020). QTL mapping for grain yield-related traits in bread wheat *via* SNP-based selective genotyping. Theor. Appl. Genet. 133, 857–872. doi: 10.1007/s00122-019-03511-0 31844965

[B64] Yao F. Q. Li X. H. Wang H. Song Y. N. Li Z. Q. Li X. G.. (2021). Down-expression of *TaPIN1s* increases the tiller number and grain yield in wheat. BMC Plant Biol. 21, 443. doi: 10.1186/s12870-021-03217-w 34592922 PMC8482684

[B65] Yu K. Liu D. Chen Y. Wang D. Yang W. Yang W.. (2019). Unraveling the genetic architecture of grain size in einkorn wheat through linkage and homology mapping and transcriptomic profiling. J. Exp. Bot. 70, 4671–4688. doi: 10.1093/jxb/erz247 31226200 PMC6760303

[B66] Yu M. Mao S. L. Chen G. Y. Pu Z. E. Wei Y. M. Zheng Y. L. (2014). QTLs for uppermost internode and spike length in two wheat RIL populations and their affect upon plant height at an individual QTL level. Euphytica 200, 95–108. doi: 10.1007/s10681-014-1156-7

[B67] Zhang X. Deng Z. Wang Y. Li J. Tian J. (2014a). Unconditional and conditional QTL analysis of kernel weight related traits in wheat (*Triticum aestivum* L.) in multiple genetic backgrounds. Genetica 142, 371–379. doi: 10.1007/s10709-014-9781-6 25060952

[B68] Zhang Y. Liu J. Xia X. He Z. (2014b). *TaGS-D1*, an ortholog of rice *OsGS3*, is associated with grain weight and grain length in common wheat. Mol. Breed. 34, 1097–1107. doi: 10.1007/s11032-014-0102-7

[B69] Zhang L. Zhao Y. L. Gao L. F. Zhao G. Y. Zhou R. H. Zhang B. S.. (2012). *TaCKX6-D1*, the ortholog of rice *OsCKX2*, is associated with grain weight in hexaploid wheat. New Phytol. 195, 574–584. doi: 10.1111/j.1469-8137.2012.04194.x 22670578

[B70] Zhao J. Wang S. Qin J. Sun C. Liu F. (2020). The lipid transfer protein OsLTPL159 is involved in cold tolerance at the early seedling stage in rice. Plant Biotechnol. J. 18, 756–769. doi: 10.1111/pbi.13243 31469486 PMC7004919

[B71] Zheng B. S. Le Gouis J. Leflon M. Rong W. Y. Laperche A. Brancourt-Hulmel M. (2010). Using probe genotypes to dissect QTL × environment interactions for grain yield components in winter wheat. Theor. Appl. Genet. 121, 1501–1517. doi: 10.1007/s00122-010-1406-6 20697687

