# Supplementary material for: Genome-wide association study of yield-related traits in common wheat (Triticum aestivum L.) under normal and drought treatment conditions
Source: Front Plant Sci. 2023 Jan 4;13:1098560. doi: 10.3389/fpls.2022.1098560 (PMC9846334; doi:10.3389/fpls.2022.1098560)
Supplement: Supplementary file 1 [file DataSheet_1.docx]

Supplementary Material

# Supplementary Figures and Tables

## Supplementary Figures
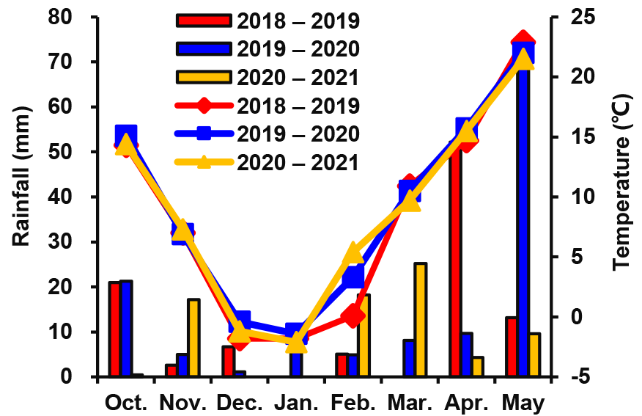


**Supplementary Figure 1.** Monthly rainfall distribution and air temperature during the wheat growing seasons in experimental fields at Shijiazhuang, Hebei Province, China in 2018 – 2019, 2019 – 2020 and 2020 – 2021.


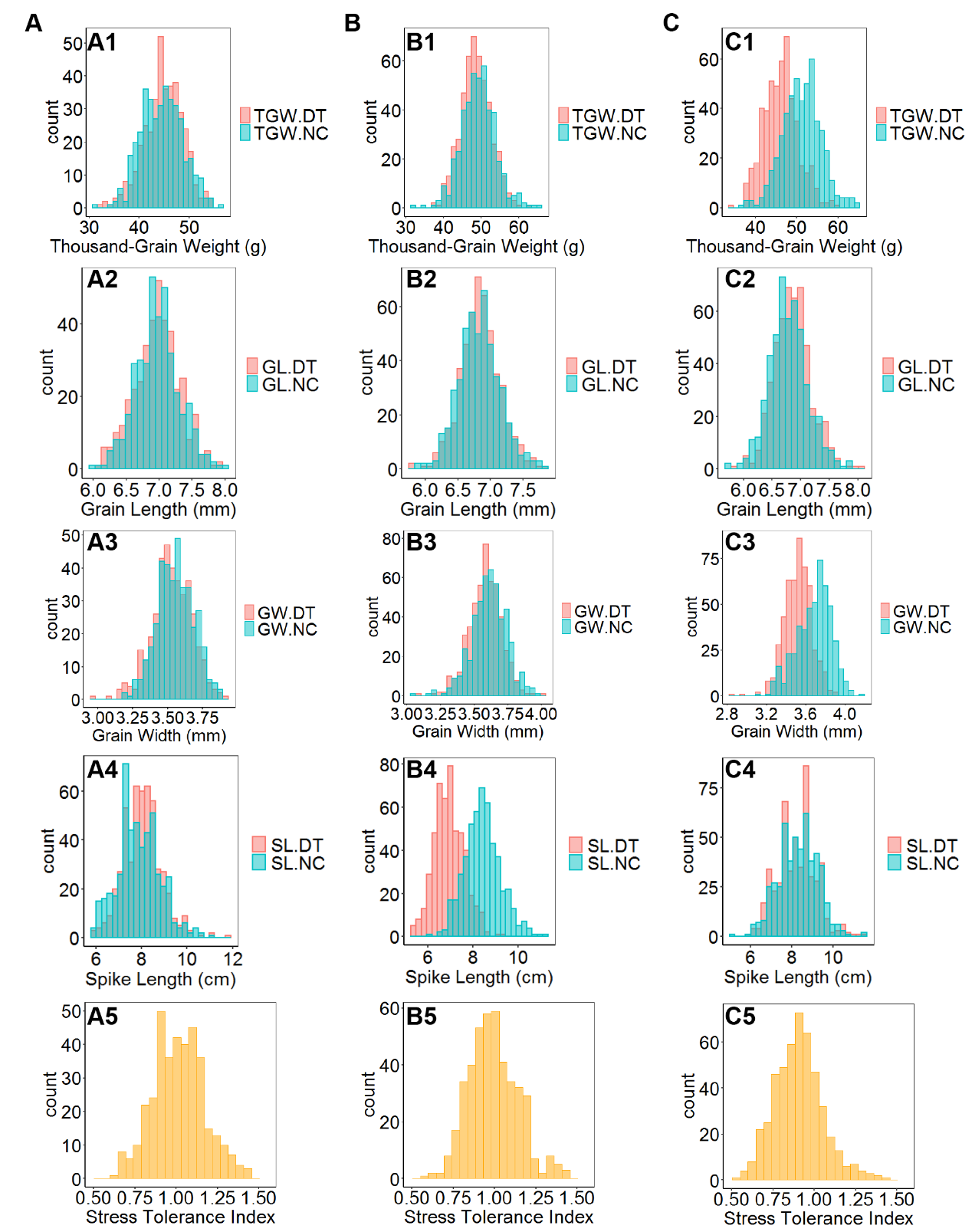


**Supplementary Figure 2.** Phenotype frequency distribution in multiple environments. **(A)** 2018 – 2019, **(B)** 2019 – 2020, **(C)** 2020 – 2021. TGW.NC, thousand-grain weight under normal conditions; TGW.DT, thousand-grain weight under drought treatment conditions; GL.NC, grain length under normal conditions; GL.DT, grain length under drought treatment conditions; GW.NC, grain width under normal conditions; GW.DT, grain width under drought treatment conditions; SL.NC, spike length under normal conditions; SL.DT, spike length under drought treatment conditions.


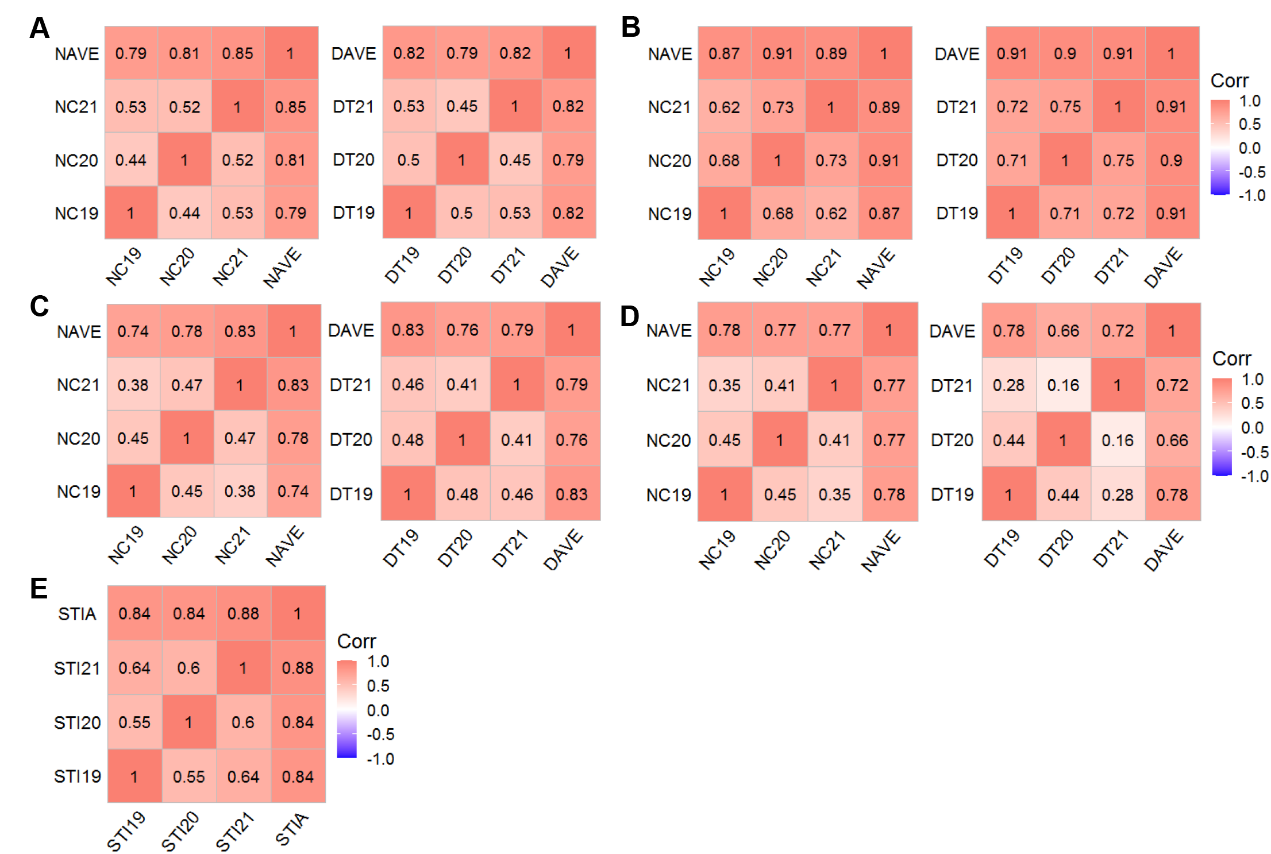


**Supplementary Figure 3.** Correlation coefficients for the detected phenotypes during three crop seasons. **(A)** Thousand-grain weight, **(B)** Grain length, **(C)** Grain width, **(D)** Spike length and **(E)** Stress tolerance index. NC19, NC20, NC21 and NAVE represented the detected phenotypes in 2018 – 2019, 2019 – 2020, 2020 – 2021 and the average value under normal conditions; DT19, DT20, DT21 and DAVE represented the detected phenotypes in 2018 – 2019, 2019 – 2020, 2020 – 2021 and the average value under drought treatment conditions; STI19, STI20, STI21 and STIA represented stress tolerance index in 2018 – 2019, 2019 – 2020, 2020 – 2021 and the average STI, respectively.


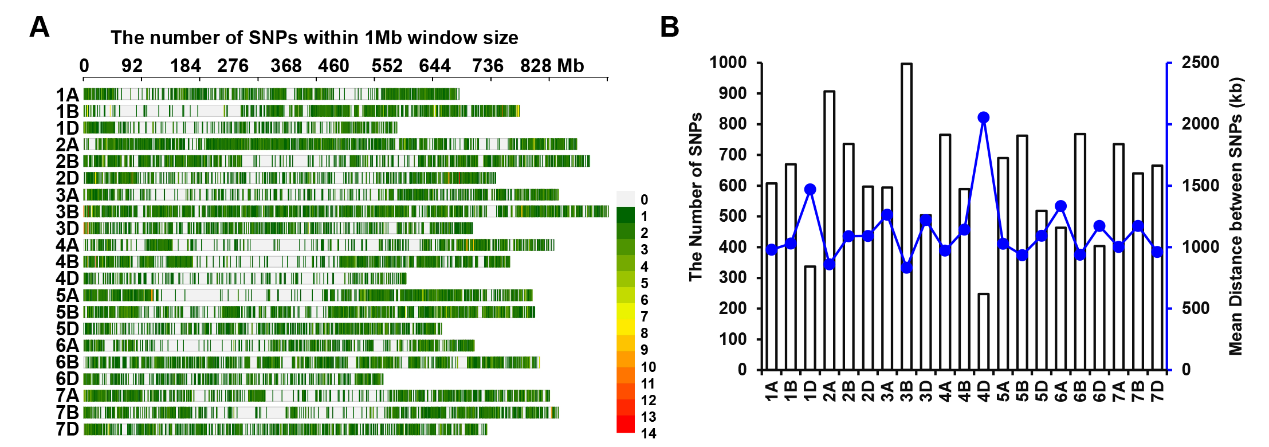


**Supplementary Figure 4.** The distribution of SNP (single nucleotide polymorphism) on 21 wheat chromosomes. **(A)** SNP density on 21 wheat chromosomes, **(B)** Statistical status of SNPs on 21 wheat chromosomes.


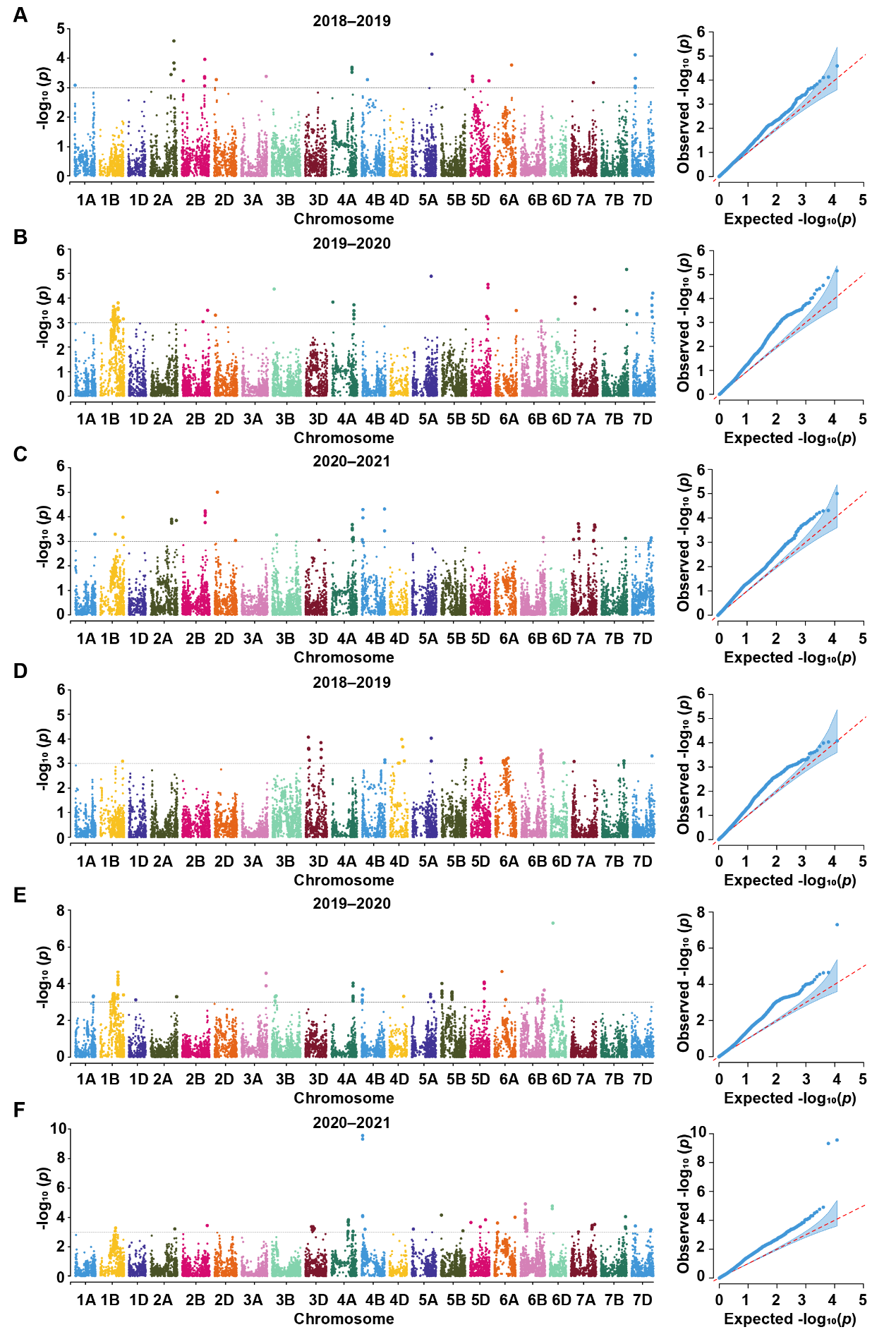


**Supplementary Figure 5.** Manhattan and Q-Q plots for thousand-grain weight in 2018 – 2019, 2019 – 2020 and 2020 – 2021 under normal **(A, B, C)** and drought treatment **(D, E, F)** conditions.


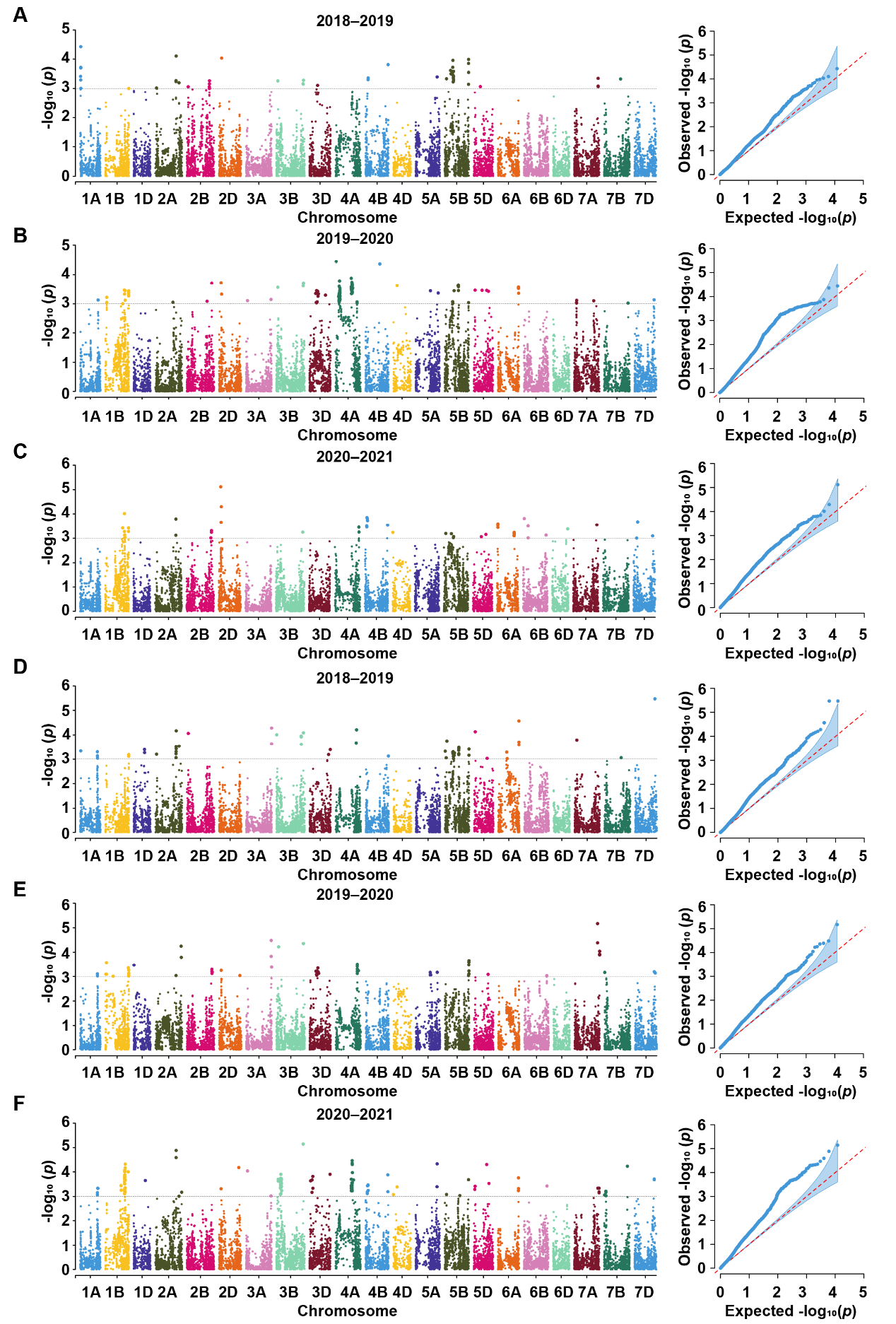


**Supplementary Figure 6.** Manhattan and Q-Q plots for grain length in 2018 – 2019, 2019 – 2020 and 2020 – 2021 under normal **(A, B, C)** and drought treatment **(D, E, F)** conditions.


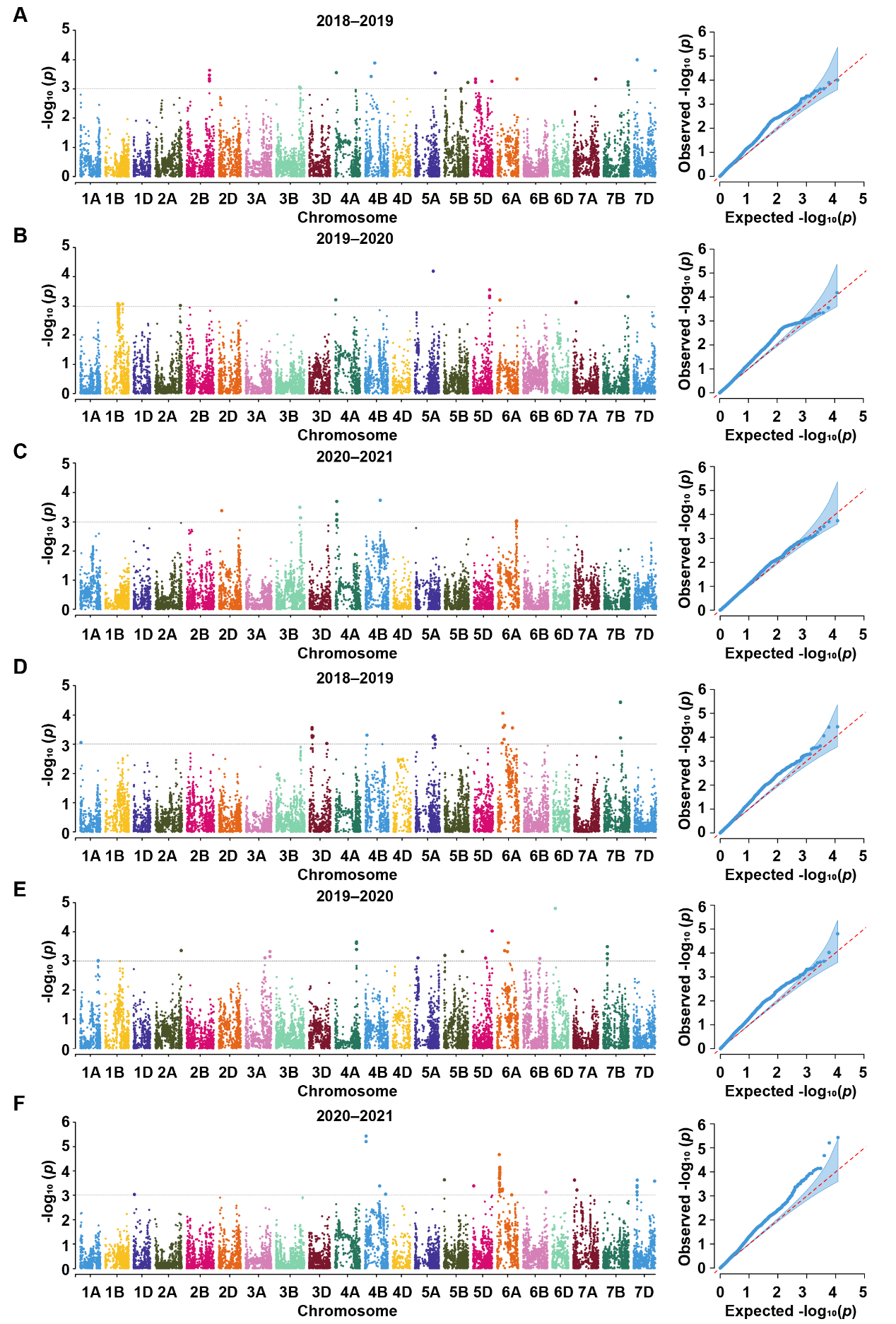


**Supplementary Figure 7.** Manhattan and Q-Q plots for grain width in 2018 – 2019, 2019 – 2020 and 2020 – 2021 under normal **(A, B, C)** and drought treatment **(D, E, F)** conditions.


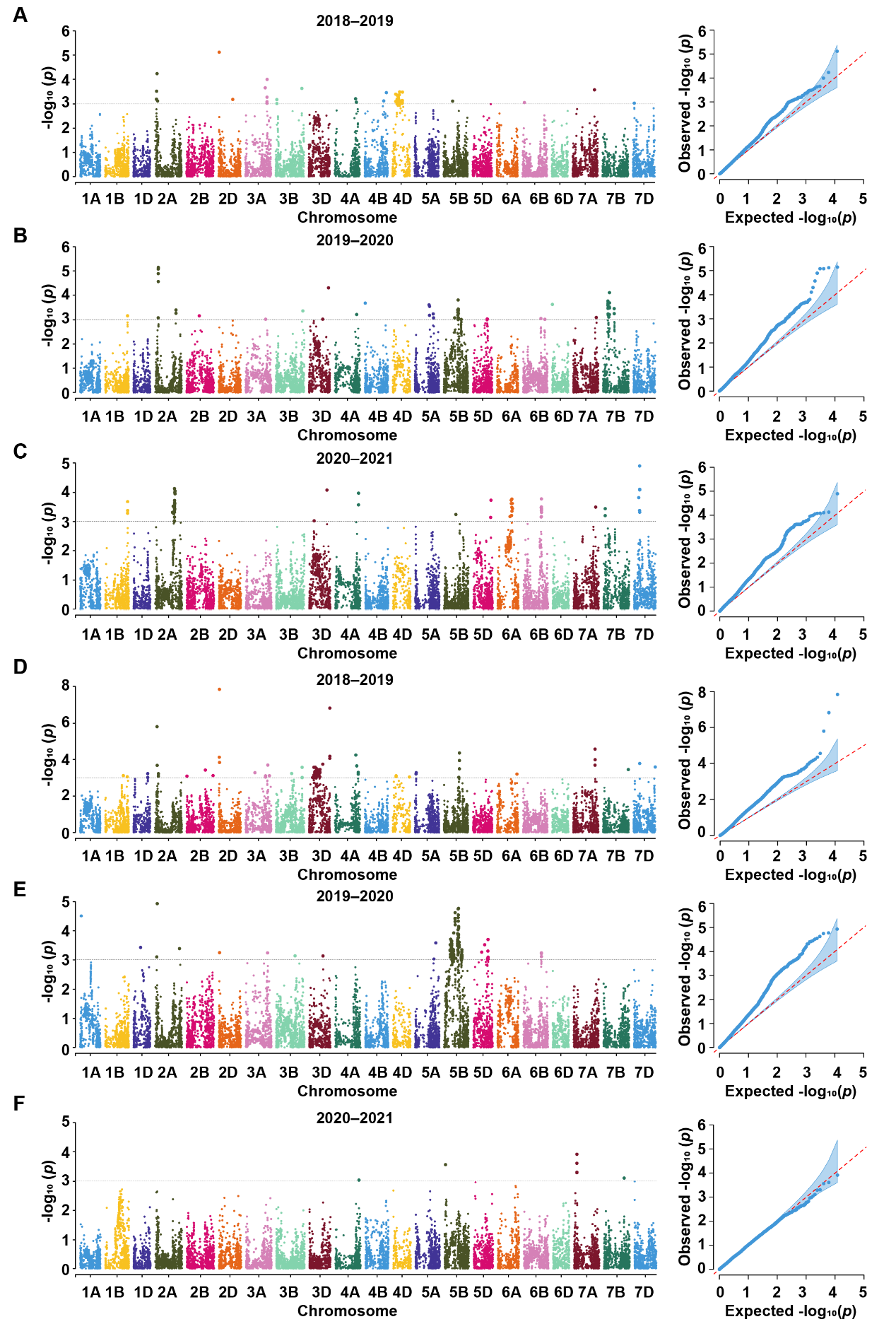


**Supplementary Figure 8.** Manhattan and Q-Q plots for spike length in 2018 – 2019, 2019 – 2020 and 2020 – 2021 under normal **(A, B, C)** and drought treatment **(D, E, F)** conditions.


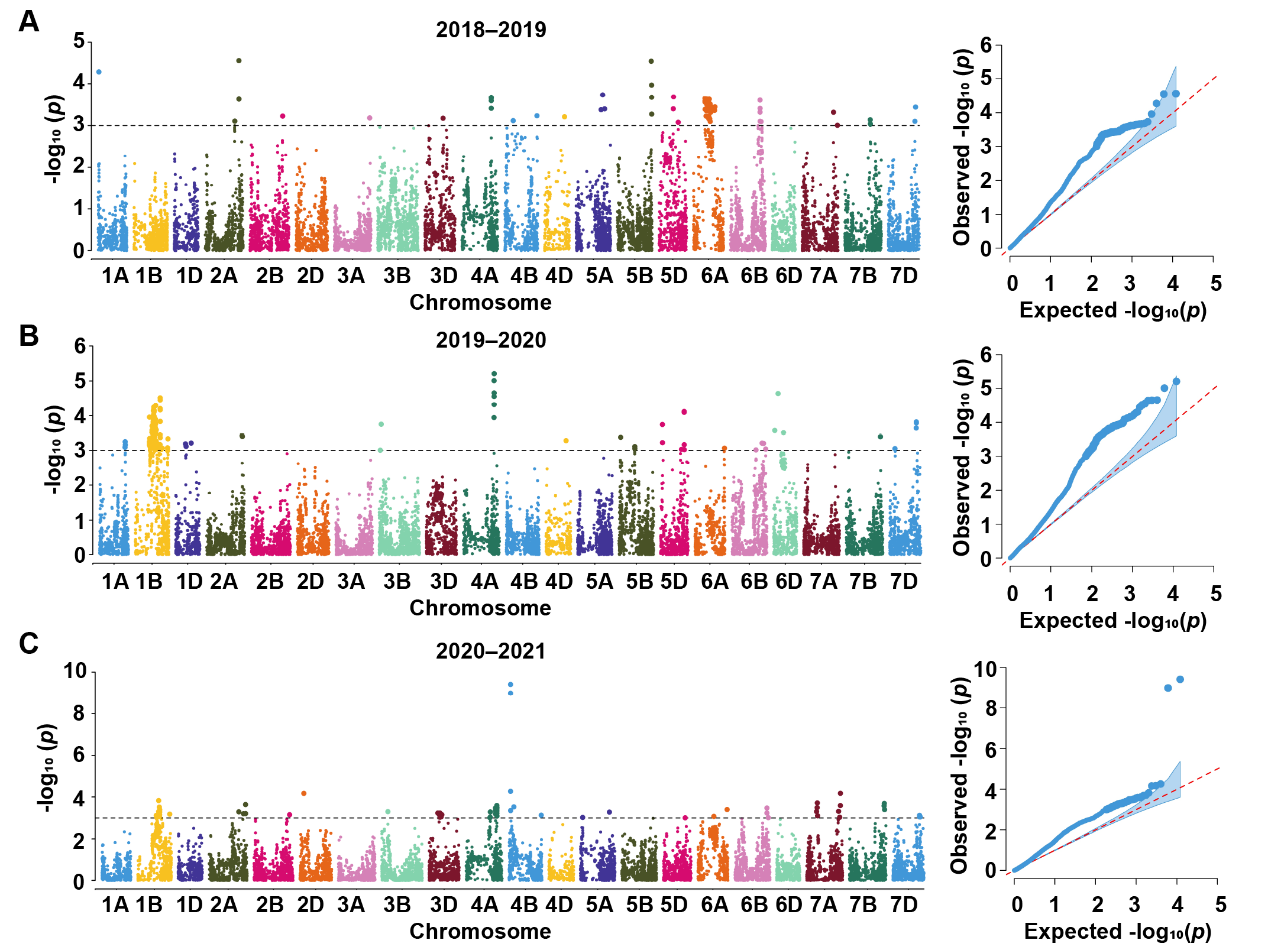


**Supplementary Figure 9.** Manhattan and Q-Q plots for stress tolerance index in 2018 – 2019 **(A)**, 2019 – 2020 **(B)** and 2020 – 2021 **(C)**.


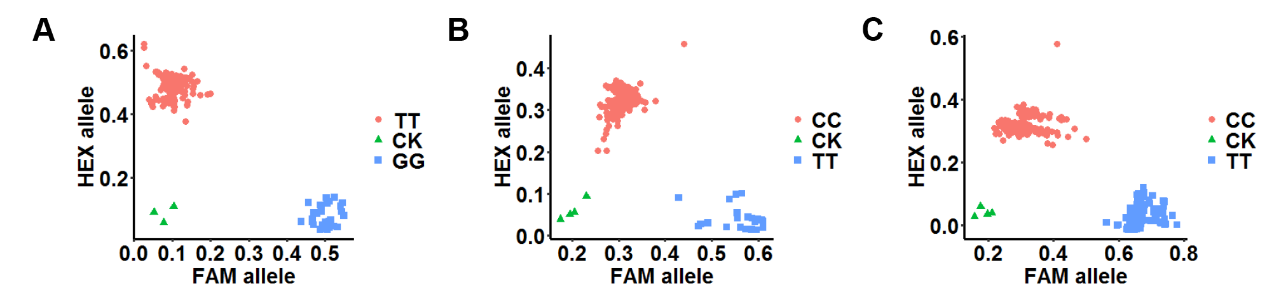


**Supplementary Figure 10.** Clustering of different accessions in scatter plots for KASP assays. **(A)** *AX-109290429*, **(B)** *AX-110418888*, **(C)** *AX-109369427*.


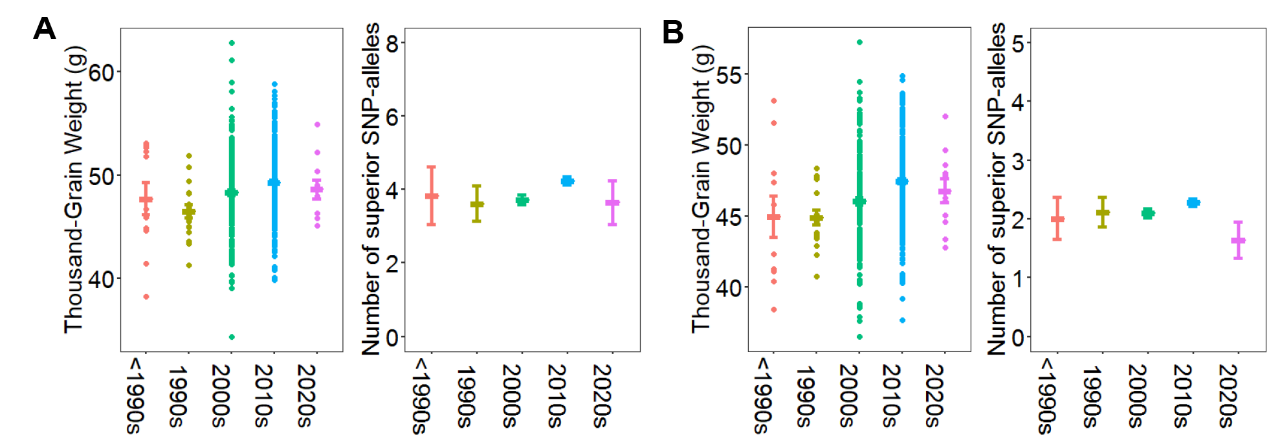


**Supplementary Figure 11.** The average thousand-grain weight and number of superior alleles in cultivars released before the 1990s, 1990s, 2000s, 2010s and 2020s. The distributions of thousand-grain weight and number of superior alleles for thousand-grain weight under normal **(A)** and drought treatment conditions **(B)**. Values shown are means ± SEM.

## Supplementary Tables

**Supplementary Table 1.** Details information of the 502 wheat accessions for genome-wide association analysis.

| **Number** | **Accessions** | **Year** | **Origin** |
| --- | --- | --- | --- |
| 1 | CG15-009 |  |  |
| 2 | DH155 | 2009 | Beijing |
| 3 | THW09-1 | 2017 | Henan |
| 4 | WF2 | 2017 | Hebei |
| 5 | YB66180 | 2009 | Hebei |
| 6 | Aikang 58 | 2005 | Henan |
| 7 | Baiqimai | 1950 | Gansu |
| 8 | Bainong 160 | 2007 | Henan |
| 9 | Bainong 4199 | 2017 | Henan |
| 10 | Bangmai 207 | 2017 | Shandong |
| 11 | Bangmai 32 | 2016 | Shandong |
| 12 | Baofeng10-82 | 2006 | Anhui |
| 13 | Baomai 10 | 2004 | Hebei |
| 14 | Bomai 7 | 2018 | Hebei |
| 15 | Bomai 8 | 2017 | Hebei |
| 16 | Bomai 7612 | 2017 | Hebei |
| 17 | Cangmai 208 | 2009 | Hebei |
| 18 | Cangmai 6005 | 2010 | Hebei |
| 19 | Cangxuan 18 | 2017 | Hebei |
| 20 | Cunmai 12 | 2016 | Henan |
| 21 | Cunmai 619 | 2020 | Henan |
| 22 | Cunmai 8 | 2014 | Henan |
| 23 | Daimai 2251 | 2014 | Shandong |
| 24 | Dehongfu 6 | 2014 | Henan |
| 25 | Deyou 061 | 2010 | Shandong |
| 26 | Dezhou 08-12 | 2008 | Shandong |
| 27 | Denghai 51306 | 2015 | Shandong |
| 28 | Dongmai 15 | 2015 | Hebei |
| 29 | Dongmai 35 | 2017 | Hebei |
| 30 | Emai 170 | 2014 | Hubei |
| 31 | Fanmai 9 | 2007 | Henan |
| 32 | Feng 1001 | 2015 | Gansu |
| 33 | Fengchuan 6 | 2003 | Shandong |
| 34 | Fengde 3 | 2012 | Henan |
| 35 | Fengdecunmai 1 | 2011 | Henan |
| 36 | Fengdecunmai 4 | 2013 | Henan |
| 37 | Fengkang 13 | 1980 | Beijing |
| 38 | Fengkang 15 | 1983 | Beijing |
| 39 | Fengyuan 2017 | 2017 | Henan |
| 40 | Fusui 1 | 2018 | Henan |
| 41 | Fumai 2008 | 2006 | Henan |
| 42 | Gaoyou 503 | 2001 | Hebei |
| 43 | Gaoyou 5218 | 2014 | Hebei |
| 44 | Gaoyou 5766 | 2008 | Hebei |
| 45 | Gaoyou 8901 | 1998 | Hebei |
| 46 | Gaoyou 2018 | 2005 | Hebei |
| 47 | Gaunmai 1 | 2016 | Henan |
| 48 | Guomai 301 | 2012 | Henan |
| 49 | Han 08-6012 | 2008 | Hebei |
| 50 | Han 3475 | 1990 | Hebei |
| 51 | Han 4564 | 2006 | Hebei |
| 52 | Han 4589 | 2001 | Hebei |
| 53 | Han 6172 | 1995 | Hebei |
| 54 | Han 7086 | 2006 | Hebei |
| 55 | Hanmai 4 | 2000 | Hebei |
| 56 | Hanmai 7 | 2005 | Hebei |
| 57 | Hanmai 13 | 2009 | Hebei |
| 58 | Hannong 1412 | 2016 | Hebei |
| 59 | Hannong1812 | 2016 | Hebei |
| 60 | Hansheng 730 | 2016 | Hebei |
| 61 | Hanyou 1 | 2007 | Hebei |
| 62 | Hanzao 1 | 2010 | Hebei |
| 63 | Hanxuan 10 | 1966 | Shanxi |
| 64 | Hangmai 186 | 2018 | Beijing |
| 65 | Hangmai 301 | 2007 | Shandong |
| 66 | Hedongwumai | 1997 | Shanxi |
| 67 | Henong 130 | 2014 | Hebei |
| 68 | Henong 4198 | 2005 | Hebei |
| 69 | Henong 5156 | 2006 | Hebei |
| 70 | Henong 58-3 | 2006 | Hebei |
| 71 | Henong 6049 | 2009 | Hebei |
| 72 | Henong 6085 | 2014 | Hebei |
| 73 | Henong 7069 | 2013 | Hebei |
| 74 | Henong 7106 | 2012 | Hebei |
| 75 | Henong 825 | 2009 | Hebei |
| 76 | Henong 9311 | 2009 | Hebei |
| 77 | Hemai 0839 | 2008 | Shandong |
| 78 | Hemai 06-77 | 2006 | Shandong |
| 79 | Heima 1 | 2004 | Shandong |
| 80 | Heng 0628 | 2008 | Hebei |
| 81 | Heng 06-6632 | 2010 | Hebei |
| 82 | Heng 0816 | 2013 | Hebei |
| 83 | Heng 10s29-2 | 2012 | Hebei |
| 84 | Heng 11-6021 | 2015 | Hebei |
| 85 | Heng 136 | 2009 | Hebei |
| 86 | Heng 1589 | 2018 | Hebei |
| 87 | Heng 4399 | 2003 | Hebei |
| 88 | Heng 4444 | 2012 | Hebei |
| 89 | Heng 5229 | 2004 | Hebei |
| 90 | Heng 5299 | 2005 | Hebei |
| 91 | Heng 5366 | 2008 | Hebei |
| 92 | Heng 5835 | 2018 | Hebei |
| 93 | Heng 7228 | 2005 | Hebei |
| 94 | Heng 9966 | 2018 | Hebei |
| 95 | Heng S13-5005 | 2013 | Hebei |
| 96 | Heng S13-5022 | 2013 | Hebei |
| 97 | Hengfu1416 | 2014 | Hebei |
| 98 | Hengguan 35 | 2004 | Hebei |
| 99 | Hengke 6021 | 2016 | Hebei |
| 100 | Hengshui 714 | 1978 | Hebei |
| 101 | Hengyou 18 | 2004 | Hebei |
| 102 | Hengyuan 5 | 2000s | Hebei |
| 103 | Hongdi 95 | 2016 | Shandong |
| 104 | Hongyun 1 | 2017 |  |
| 105 | Huapei 176 | 1990 | Hebei |
| 106 | Huapei 3 | 2002 | Henan |
| 107 | Huapei 6 | 2008 | Henan |
| 108 | Huacheng 3366 | 2013 | Anhui |
| 109 | Huamai 8 | 2016 | Jiangsu |
| 110 | Huayu 116 | 2010 | Henan |
| 111 | Huaimai 18 | 1999 | Jiangsu |
| 112 | Huaimai 25 | 2007 | Jiangsu |
| 113 | Huaimai 29 | 2009 | Jiangsu |
| 114 | Huangshengmai 98 |  |  |
| 115 | Jimai 4 | 2005 | Shandong |
| 116 | Jimai 19 | 2001 | Shandong |
| 117 | Jimai 20 | 2004 | Shandong |
| 118 | Jimai 21 | 2004 | Shandong |
| 119 | Jimai 22 | 2006 | Shandong |
| 120 | Jimai 229 | 2016 | Shandong |
| 121 | Jimai 44 | 2018 | Shandong |
| 122 | Jimai 60 | 2018 | Shandong |
| 123 | Jinan 35037 | 2010 | Shandong |
| 124 | Jining 16 | 2001 | Shandong |
| 125 | Ji 5265 | 2009 | Hebei |
| 126 | Ji 7369 | 2003 | Hebei |
| 127 | Jifeng 703 | 2005 | Hebei |
| 128 | Jifeng 717 | 2015 | Hebei |
| 129 | Jimai 031 | 2017 | Hebei |
| 130 | Jimai 120 | 2016 | Hebei |
| 131 | Jimai 138 | 2009 | Hebei |
| 132 | Jimai 161 | 2015 | Hebei |
| 133 | Jimai 181 | 2016 | Hebei |
| 134 | Jimai 21 | 1985 | Hebei |
| 135 | Jimai 26 | 1988 | Hebei |
| 136 | Jimai 38 | 1994 | Hebei |
| 137 | Jimai 42 | 2010 | Hebei |
| 138 | Jimai 325 | 2016 | Hebei |
| 139 | Jimai 403 | 2006 | Hebei |
| 140 | Jimai 418 | 2016 | Hebei |
| 141 | Jimai 479 | 2018 | Hebei |
| 142 | Jimai 485 | 2016 | Hebei |
| 143 | Jimai 511 | 2017 | Hebei |
| 144 | Jimai 518 | 2013 | Hebei |
| 145 | Jimai 520 | 2017 | Hebei |
| 146 | Jimai 585 | 2011 | Hebei |
| 147 | Jimai 599 | 2017 | Hebei |
| 148 | Jimai 631 | 2016 | Hebei |
| 149 | Jimai 665 | 2017 | Hebei |
| 150 | Jimai 738 | 2016 | Hebei |
| 151 | Jimai 782 | 2017 | Hebei |
| 152 | Jimai 817 | 2018 | Hebei |
| 153 | Jimai 867 | 2015 | Hebei |
| 154 | Jimai 929 | 2020 | Hebei |
| 155 | Jimai 958 | 2020 | Hebei |
| 156 | Jimai U68 | 2019 | Hebei |
| 157 | Jimai U75 | 2020 | Hebei |
| 158 | Jimai U80 | 2017 | Hebei |
| 159 | Jinuo 200 | 2007 | Hebei |
| 160 | Jinuo 58 | 2013 | Hebei |
| 161 | Jiqumai 26 | 2003 | Hebei |
| 162 | Jishi 1107 | 2011 | Hebei |
| 163 | Jixing 868 | 2018 | Hebei |
| 164 | Jiafeng 80 | 2016 | Hebei |
| 165 | Jieyou 4 |  |  |
| 166 | Jiemai 19 | 2015 | Hebei |
| 167 | Jinfeng 7183 | 2014 | Hebei |
| 168 | Jinhe 15-310 | 2015 | Hebei |
| 169 | Jinhe 9123 | 2008 | Hebei |
| 170 | Jinhuangmai 6 |  |  |
| 171 | Jinmai 1 | 2014 | Shaanxi |
| 172 | Jinmai 27 | 2015 | Tianjin |
| 173 | Jinmai 6002 | 2017 | Hebei |
| 174 | Jinnong 6 | 2010 | Tianjin |
| 175 | Jinmai 68 | 2017 | Hebei |
| 176 | Jinmai 811 | 2017 | Hebei |
| 177 | Jinmai 47 | 1995 | Shanxi |
| 178 | Jinmai 79 | 2006 | Shanxi |
| 179 | Jinmai 85 | 2008 | Shanxi |
| 180 | Jinmai 92 | 2012 | Shanxi |
| 181 | Jinmai 95 | 2014 | Shanxi |
| 182 | Jinmai 96 | 2014 | Shanxi |
| 183 | Jintai 170 | 2003 | Shanxi |
| 184 | Jing 411 | 1992 | Beijing |
| 185 | Jingdong 17 | 2007 | Beijing |
| 186 | Jingdong 18 | 2010 | Beijing |
| 187 | Jinghua 9 | 2007 | Beijing |
| 188 | Jingmai 15 | 2019 | Beijing |
| 189 | Jingbaimai 1 | 2003 | Hebei |
| 190 | Jingyang 670 | 2016 | Shandong |
| 191 | Junda 107 | 2016 | Henan |
| 192 | Junda 129 | 2017 | Henan |
| 193 | Jun 2016 | 2011 | Henan |
| 194 | Junmai K8 | 2012 | Henan |
| 195 | Kaimai 21 | 2011 | Henan |
| 196 | Ke 1201 |  | Hebei |
| 197 | Kelin 011 | 2015 | Henan |
| 198 | Kemai 3 | 2007 | Sichuan |
| 199 | Kenong 1002 | 2021 | Hebei |
| 200 | Kenong 1006 | 2013 | Hebei |
| 201 | Kenong 199 | 2006 | Hebei |
| 202 | Kenong 2009 | 2010 | Hebei |
| 203 | Kenong 2011 | 2016 | Hebei |
| 204 | Kenong 8024 | 2015 | Hebei |
| 205 | Kenong 8162 | 2021 | Hebei |
| 206 | Kenong 9204 | 2002 | Hebei |
| 207 | Kexin 9 | 2009 | Shandong |
| 208 | Keyi 26 | 1990 | Beijing |
| 209 | Keyi 6014 | 2013 | Shanxi |
| 210 | Keyuan 026 | 2015 | Shandong |
| 211 | Lainong 0453 | 2004 | Shandong |
| 212 | Laizhou 95021 | 2001 | Shandong |
| 213 | Lande 677 | 2015 | Hebei |
| 214 | Lande C31305 | 2016 | Hebei |
| 215 | Lankao 4 | 1990 | Henan |
| 216 | Lankaoaizao 8 | 2003 | Henan |
| 217 | Lantian 081 | 2018 | Gansu |
| 218 | Langyan 3611 | 2009 | Hebei |
| 219 | Lemai 558 | 2010 | Hebei |
| 220 | Lemai Z651 | 2016 | Anhui |
| 221 | Letu 808 | 2019 | Hebei |
| 222 | Letu 909 | 2019 | Hebei |
| 223 | Liyu 18 |  |  |
| 224 | Lianmai 2 | 2005 | Jiangsu |
| 225 | Liangxing 66 | 2002 | Shandong |
| 226 | Liangxing 99 | 2004 | Shandong |
| 227 | Liangsheng 101 | 2016 | Hebei |
| 228 | Liangsheng 103 | 2015 | Hebei |
| 229 | Liaomai 18 | 2006 | Shandong |
| 230 | Liaomai 19 | 2007 | Shandong |
| 231 | Lin Y7287 | 2009 | Shanxi |
| 232 | Linhan 6 | 2006 | Shanxi |
| 233 | Linmai 4 | 2006 | Shandong |
| 234 | Linyuan 8 | 2010 | Shanxi |
| 235 | Longmai 1 | 2019 | Hebei |
| 236 | Longmai 2 | 2017 | Hebei |
| 237 | Longmai 28 | 2016 | Jiangsu |
| 238 | Longpingmai 5 | 2013 | Henan |
| 239 | Longpingmai 7 | 2017 | Anhui |
| 240 | Lumai 5 | 2006 | Shandong |
| 241 | Lumai 16 | 1990 | Shandong |
| 242 | Luxingmai 9 |  | Shandong |
| 243 | Luyan 213 | 2017 | Shandong |
| 244 | Luyan 217 | 2017 | Shandong |
| 245 | Luyuan 502 | 2011 | Shandong |
| 246 | Luyuan 890 | 2016 | Shandong |
| 247 | Lunxuan 061 | 2006 | Hebei |
| 248 | Lunxuan 103 | 2015 | Hebei |
| 249 | Lunxuan 192 | 2020 | Hebei |
| 250 | Lunxuan 518 | 2007 | Beijing |
| 251 | Lunxuan 987 | 2003 | Beijing |
| 252 | Lunxuan 988 | 2009 | Henan |
| 253 | Luohan 2 | 2001 | Henan |
| 254 | Luohan 6 | 2006 | Henan |
| 255 | Luohan 9 | 2009 | Henan |
| 256 | Luohan 11 | 2008 | Henan |
| 257 | Luohan 13 | 2009 | Henan |
| 258 | Luomai 21 | 2009 | Henan |
| 259 | Luomai 23 | 2009 | Henan |
| 260 | Luomai 24 | 2013 | Henan |
| 261 | Luomai 29 | 2016 | Henan |
| 262 | Luomai 8 | 2007 | Henan |
| 263 | Luomai 9 | 2008 | Henan |
| 264 | Minxing 375 | 2016 | Henan |
| 265 | Minxing 379 | 2017 | Henan |
| 266 | Neinongke 201 | 2007 | Henan |
| 267 | Ningfeng 518 | 2012 | Anhui |
| 268 | Ningmai 13 | 2006 | Jiangsu |
| 269 | Ningmai 16 | 2009 | Jiangsu |
| 270 | Nongda 211 | 2007 | Beijing |
| 271 | Nongda 399 | 2007 | Hebei |
| 272 | Nongda 5363 | 2013 | Beijing |
| 273 | Nongmai 1 | 2015 | Jiangsu |
| 274 | Pingan 7 | 2008 | Henan |
| 275 | Pingan 9 | 2014 | Henan |
| 276 | Pingmai 108-9 | 2013 | Henan |
| 277 | Puxing 03044 | 2014 | Henan |
| 278 | Puxing 5 | 2017 | Henan |
| 279 | Pubing 3737 | 2018 | Shaanxi |
| 280 | Qimai 2 | 2014 | Shandong |
| 281 | Qimai 5 | 2013 | Shandong |
| 282 | Qingmai 6 | 2007 | Shandong |
| 283 | Qingnong 9 | 2020 | Shandong |
| 284 | Qiule 2122 | 2014 | Henan |
| 285 | Qiushuo 001 | 2009 | Hebei |
| 286 | Quanmai 890 | 2017 | Henan |
| 287 | Shannong 14 | 2006 | Shandong |
| 288 | Shannong 20 | 2010 | Shandong |
| 289 | Shannong 24 | 2013 | Shandong |
| 290 | Shannong 25 | 2014 | Shandong |
| 291 | Shannong 27 | 2014 | Shandong |
| 292 | Shannong 28 | 2017 | Shandong |
| 293 | Shannong 29 | 2016 | Shandong |
| 294 | Shannong 055849 | 2013 | Shandong |
| 295 | Shannong 0911 | 2014 | Shandong |
| 296 | Shannong 2149 | 2009 | Shandong |
| 297 | Shannong 411603 | 2016 | Shandong |
| 298 | Shannong 8355 | 2005 | Shandong |
| 299 | Shannong K32561 | 2016 | Shandong |
| 300 | Shannong N4283 | 2016 | Shandong |
| 301 | Shan 253 | 2001 | Shaanxi |
| 302 | Shanken 224 | 2014 | Shaanxi |
| 303 | Shannong 78 | 2002 | Shaanxi |
| 304 | Shenmai 1 | 2004 | Henan |
| 305 | Shengmai 104 | 2014 | Shandong |
| 306 | Shengmai 23 | 2019 | Shandong |
| 307 | Shengmai 5 | 2013 | Shandong |
| 308 | Shengtianmai 69 | 2015 | Shandong |
| 309 | Shiluan 02-1 | 2007 | Hebei |
| 310 | Shiluan 08-2 | 2008 | Hebei |
| 311 | Shi 08-4741 | 2014 | Hebei |
| 312 | Shi 09-4276 | 2011 | Hebei |
| 313 | Shi 10-4195 | 2016 | Hebei |
| 314 | Shi 10-4393 | 2018 | Hebei |
| 315 | Shi 11-5139 | 2014 | Hebei |
| 316 | Shi 12-4025 | 2018 | Hebei |
| 317 | Shi 12-6260 | 2004 | Hebei |
| 318 | Shi 13-6650 | 2004 | Hebei |
| 319 | Shi 14-7022 | 2004 | Hebei |
| 320 | Shi 15-6327 | 2006 | Hebei |
| 321 | Shi 20-7221 | 2007 | Hebei |
| 322 | Shi 4185 | 1999 | Hebei |
| 323 | Shi 4366 | 2014 | Hebei |
| 324 | Shi B05-7388 | 2009 | Hebei |
| 325 | Shi B08-5341 | 2016 | Hebei |
| 326 | Shi B10-7074 | 2003 | Hebei |
| 327 | Shi H083-366 | 2010 | Hebei |
| 328 | Shi H09-7075 | 2013 | Hebei |
| 329 | Shi U06-4532 | 2007 | Hebei |
| 330 | Shi U10-4045 | 2013 | Hebei |
| 331 | Shi U11-349 | 2011 | Hebei |
| 332 | Shijiazhuang 10 | 2003 | Hebei |
| 333 | Shijiazhuang 8 | 2001 | Hebei |
| 334 | Shimai 12 | 2004 | Hebei |
| 335 | Shimai 14 | 2004 | Hebei |
| 336 | Shimai 15 | 2007 | Hebei |
| 337 | Shimai 16 | 2009 | Hebei |
| 338 | Shimai 18 | 2008 | Hebei |
| 339 | Shimai 19 | 2009 | Hebei |
| 340 | Shimai 22 | 2011 | Hebei |
| 341 | Shinong 086 | 2009 | Hebei |
| 342 | Shixin 549 | 2017 | Hebei |
| 343 | Shixin 616 | 2008 | Hebei |
| 344 | Shixin 633 | 2013 | Hebei |
| 345 | Shixin 828 | 2005 | Hebei |
| 346 | Shiyou 17 | 2007 | Hebei |
| 347 | Shuangfengshou |  | Shaanxi |
| 348 | Shunmai 1718 | 2007 | Shanxi |
| 349 | Tai 95-1 | 1995 | Shandong |
| 350 | Taikemai 33 | 2018 | Shandong |
| 351 | Taikemai 6309 | 2013 | Shandong |
| 352 | Tainong 18 | 2008 | Shandong |
| 353 | Tainong 9862 | 2011 | Shandong |
| 354 | Taishan 21 | 2003 | Shandong |
| 355 | Taishan 22 | 2004 | Shandong |
| 356 | Taishan 23 | 2004 | Shandong |
| 357 | Taishan 24 | 2005 | Shandong |
| 358 | Taishan 27 | 2012 | Shandong |
| 359 | Taishan 28 | 2013 | Shandong |
| 360 | Taishan 5366 | 2010 | Shandong |
| 361 | Taitianmai 118 | 2020 | Shandong |
| 362 | Taitianmai 126 | 2020 | Shandong |
| 363 | Tanmai 98 | 2009 | Shandong |
| 364 | Tianhe 3 | 2012 | Henan |
| 365 | Tianhe 4 | 2015 | Henan |
| 366 | Tianmin 298 | 2016 | Henan |
| 367 | Tiantaiyoumai 2 | 2019 | Shandong |
| 368 | Tonggeng 1 | 2014 | Hebei |
| 369 | Tunfeng 801 | 2015 | Henan |
| 370 | Tunmai 258 | 2020 | Henan |
| 371 | Tunmai 3638 | 2011 | Henan |
| 372 | Wan 981 | 2016 | Anhui |
| 373 | Wannong 306 | 2016 | Anhui |
| 374 | Wenhang 6 | 2006 | Shandong |
| 375 | Wennong 14 | 2011 | Shandong |
| 376 | Wennong 20 | 2018 | Shandong |
| 377 | Wennong 5 | 2003 | Shandong |
| 378 | Womai 4176 | 2017 | Hebei |
| 379 | Xinong 2208 | 2003 | Shaanxi |
| 380 | Xinong 538 | 2010 | Shaanxi |
| 381 | Xinong 556 | 2012 | Shaanxi |
| 382 | Xinong 558 | 2011 | Shaanxi |
| 383 | Xinong 889 | 2005 | Shaanxi |
| 384 | Xinong 928 | 2008 | Shaanxi |
| 385 | Xinong 979 | 2005 | Shaanxi |
| 386 | Xianmai 10 | 2012 | Henan |
| 387 | Xianmai 8 | 2013 | Hubei |
| 388 | Xiangmai 55 | 2009 | Hubei |
| 389 | Xiaobingmai 33 | 1995 | Heilongjiang |
| 390 | Xiaoyan 22 | 2003 | Shaanxi |
| 391 | Xiaoyan 54 | 1999 | Shaanxi |
| 392 | Xiaoyan 6 | 1981 | Shaanxi |
| 393 | Xiaoyan 60 | 2016 | Hebei |
| 394 | Xinliang 2 |  |  |
| 395 | Xinmai 11 | 2003 | Henan |
| 396 | Xinmai 18 | 2004 | Henan |
| 397 | Xinmai 19 | 2006 | Henan |
| 398 | Xinmai 26 | 2010 | Henan |
| 399 | Xinmai 28 | 2014 | Henan |
| 400 | Xinmai 296 | 2014 | Shandong |
| 401 | Xinmai 807 | 2019 | Shandong |
| 402 | Xinxing 169 | 2017 | Shandong |
| 403 | Xingmai 10 | 2015 | Hebei |
| 404 | Xingmai 6 | 2008 | Hebei |
| 405 | Xumai 0031 | 2013 | Jiangsu |
| 406 | Xumai 9169 | 2012 | Jiangsu |
| 407 | Xuke 316 | 2011 | Henan |
| 408 | Xuke 718 | 2012 | Henan |
| 409 | Yan 99102 | 2011 | Shandong |
| 410 | Yannong 0428 | 2008 | Shandong |
| 411 | Yannong 1212 | 2018 | Shandong |
| 412 | Yannong 21 | 2002 | Shandong |
| 413 | Yannong 836 | 2010 | Shandong |
| 414 | Yanyou 361 | 2001 | Shandong |
| 415 | Yanmai 8911 | 2001 | Shaanxi |
| 416 | Yanmai 98 | 2010 | Shandong |
| 417 | Yangao21 | 2017 | Henan |
| 418 | Yangmai 23 | 2013 | Jiangsu |
| 419 | Yangguang 10 | 2013 | Shandong |
| 420 | Yangguang 4 |  | Henan |
| 421 | Yangguang 503 | 2013 | Henan |
| 422 | Yaomai 18 | 2015 | Shanxi |
| 423 | Yimai 1 |  | Shandong |
| 424 | Yimai 2 | 1978 | Shandong |
| 425 | Yimai 6 | 2011 | Henan |
| 426 | Yingbo 700 | 2012 | Hebei |
| 427 | Yingman 208 | 2018 | Henan |
| 428 | Yong 4896 | 2002 | Hebei |
| 429 | Yongmai 3 | 2008 | Shanxi |
| 430 | Younongmai 887 | 2013 | Hebei |
| 431 | Yuliang 1688 | 2013 | Henan |
| 432 | Yutianmai 119 | 2018 | Shandong |
| 433 | Yuan 208 | 2009 | Henan |
| 434 | Yufeng 079 | 2015 | Henan |
| 435 | Yufeng 1679 | 2016 | Henan |
| 436 | Yujiao 5 | 2011 | Henan |
| 437 | Yumai 21 | 1992 | Henan |
| 438 | Yumai 34 | 1982 | Henan |
| 439 | Yumai 47 | 1997 | Henan |
| 440 | Yumai 66 | 2000 | Henan |
| 441 | Yunong 059 | 2019 | Henan |
| 442 | Yunong 416 | 2009 | Henan |
| 443 | Yunong 949 | 2005 | Henan |
| 444 | Yutong 101 | 2004 | Henan |
| 445 | Yuanmai 758 | 2015 | Hebei |
| 446 | Yuansheng 14-29 | 2017 | Hebei |
| 447 | Yunhan 102 | 2014 | Shanxi |
| 448 | Yunhan 22-33 | 2005 | Shanxi |
| 449 | Yunhan 618 | 2010 | Shanxi |
| 450 | Yunhan 805 | 2011 | Shanxi |
| 451 | Zaiyou 29 |  |  |
| 452 | Zaoshu 6061 |  |  |
| 453 | Zao 9919 | 2009 | Shandong |
| 454 | Zemai 2 | 2004 | Shanxi |
| 455 | Chang 4640 | 2004 | Shanxi |
| 456 | Chang 4738 | 2006 | Shanxi |
| 457 | Chang 6359 | 2005 | Shanxi |
| 458 | Chang 6878 | 2002 | Shanxi |
| 459 | Zhaonong 1147 | 2011 | Hebei |
| 460 | Zhengmai 004 | 2004 | Henan |
| 461 | Zhengmai 101 | 2013 | Henan |
| 462 | Zhengmai 110 | 2010 | Henan |
| 463 | Zhengmai 366 | 2005 | Henan |
| 464 | Zhengmai 7698 | 2011 | Henan |
| 465 | Zhengmai 9023 | 2001 | Henan |
| 466 | Zhengnong 17 | 2004 | Henan |
| 467 | Zhengnong 46 | 2017 | Henan |
| 468 | Zhengyumai 0519 | 2013 | Henan |
| 469 | Zhengyumai 9989 | 2007 | Henan |
| 470 | Chinese Spring | 1900 | Sichuan |
| 471 | Zhongjian 49 |  |  |
| 472 | Zhongkegao 8 |  |  |
| 473 | Zhonglemai 178 | 2017 | Anhui |
| 474 | Zhongmai 155 | 2003 | Hebei |
| 475 | Zhongmai 175 | 2011 | Hebei |
| 476 | Zhongmai 349 | 2009 | Shanxi |
| 477 | Zhongmai 415 | 2010 | Beijing |
| 478 | Zhongmai 816 | 2013 | Jiangsu |
| 479 | Zhongmai 875 | 2014 | Shandong |
| 480 | Zhongmai 895 | 2013 | Henan |
| 481 | Zhongshi 110 |  |  |
| 482 | Zhongwomai 1 | 2017 | Hebei |
| 483 | Zhongxinmai 98 | 2015 | Hebei |
| 484 | Zhongxinmai 99 | 2016 | Hebei |
| 485 | Zhongyou 206 | 2008 | Beijing |
| 486 | Zhongyou 9507 | 2001 | Beijing |
| 487 | Zhongyu 10 | 2007 | Henan |
| 488 | Zhongzhongmai 145 | 2015 | Beijing |
| 489 | Zhongzhongmai 20 | 2016 | Beijing |
| 490 | Zhongmai 998 | 2008 | Henan |
| 491 | Zhongxin 6178 | 2017 | Hebei |
| 492 | Zhou 9811 | 2009 | Henan |
| 493 | Zhoumai 16 | 2003 | Henan |
| 494 | Zhoumai 18 | 2005 | Henan |
| 495 | Zhoumai 24 | 2009 | Henan |
| 496 | Zhoumai 26 | 2012 | Henan |
| 497 | Zhoumai 27 | 2011 | Henan |
| 498 | Zhouyuan 9369 | 2007 | Shandong |
| 499 | Zhumai 395 | 2018 | Henan |
| 500 | Zhuoke 5 | 2017 | Henan |
| 501 | Zimai 12 | 2001 | Shandong |
| 502 | Zimai 28 | 2001 | Shandong |

**Supplementary Table 2.** Primers used in this study.

| **SNP** | **Primer name** | **Forward primer (5′−3′)** | **Reverse primer (5′−3′)** |
| --- | --- | --- | --- |
| *AX-109290429* | Kasp_2A-608 | **GAAGGTGACCAAGTTCATGCT**ATGAGTTAAGCAGTAGAAGGTTATGG | TAACCTAGAGAACCACAATGTCCAAAGAT |
|  |  | **GAAGGTCGGAGTCAACGGATT**GATGAGTTAAGCAGTAGAAGGTTATGT |  |
| *AX-110418888* | Kasp_3B-785 | **GAAGGTGACCAAGTTCATGCT**TTGTTAACACAGTGTGAAAAGT | CACTCACCCACTCATAACCAT |
|  |  | **GAAGGTCGGAGTCAACGGATT**TTGTTAACACAGTGTGAAAAGC |  |
| *AX-109369427* | Kasp_5A-546 | **GAAGGTGACCAAGTTCATGCT**CAGGGGCCATATGGCATATCCT | GTCGTCCTGCCATCATTTTTCAGAATATA |
|  |  | **GAAGGTCGGAGTCAACGGATT**AGGGGCCATATGGCATATCCC |  |

**Supplementary Table 3.** The significant SNPs identified by GWAS for the yield-related traits under two conditions.

| **Trait** | **SNP** | **Chr** | **Position (bp)^a^** | **Environment** | ***P* value** | **PVE (%)^b^** |
| --- | --- | --- | --- | --- | --- | --- |
| TGW.NC | *AX-111084696* | 1A | 16,422,919 | NC19 | 8.21E-04 | 2.78 |
| TGW.NC | *AX-108730532* | 1A | 547,884,554 | NAVE | 9.08E-04 | 2.06 |
| TGW.NC | *AX-108914803* | 1A | 574,481,294 | NC21 | 5.10E-04 | 2.38 |
| TGW.NC | *AX-95176576* | 1B | 314,569,349 | NC20 | 7.61E-04 | 2.46 |
| TGW.NC | *AX-110531641* | 1B | 365,441,304 | NC20 | 9.32E-04 | 2.38 |
| TGW.NC | *AX-111463788* | 1B | 365,926,056 | NC20 | 8.71E-04 | 2.41 |
| TGW.NC | *AX-111482877* | 1B | 366,492,631 | NC20 | 6.46E-04 | 2.53 |
| TGW.NC | *AX-109437757* | 1B | 366,917,565 | NC20 | 5.91E-04 | 2.57 |
| TGW.NC | *AX-111118968* | 1B | 367,588,737 | NC20 | 7.74E-04 | 2.46 |
| TGW.NC | *AX-111733823* | 1B | 367,838,063 | NC20 | 6.88E-04 | 2.51 |
| TGW.NC | *AX-110597141* | 1B | 368,675,675 | NC20 | 3.91E-04 | 2.74 |
| TGW.NC | *AX-110474956* | 1B | 369,345,717 | NC20 | 5.60E-04 | 2.59 |
| TGW.NC | *AX-110623229* | 1B | 369,515,449 | NC20 | 6.17E-04 | 2.55 |
| TGW.NC | *AX-109581046* | 1B | 370,197,585 | NC20 | 7.30E-04 | 2.48 |
| TGW.NC | *AX-108845851* | 1B | 371,140,782 | NC20 | 3.18E-04 | 2.82 |
| TGW.NC | *AX-110386580* | 1B | 372,157,887 | NC20 | 8.78E-04 | 2.41 |
| TGW.NC | *AX-109387657* | 1B | 372,217,456 | NC20 | 8.93E-04 | 2.40 |
| TGW.NC | *AX-110740030* | 1B | 373,856,428 | NC20 | 7.72E-04 | 2.46 |
| TGW.NC | *AX-111707424* | 1B | 374,316,467 | NC20 | 7.44E-04 | 2.47 |
| TGW.NC | *AX-109932384* | 1B | 374,913,451 | NC20 | 6.48E-04 | 2.53 |
| TGW.NC | *AX-109580210* | 1B | 375,303,546 | NC20 | 4.98E-04 | 2.64 |
| TGW.NC | *AX-110369203* | 1B | 375,821,909 | NC20 | 4.51E-04 | 2.68 |
| TGW.NC | *AX-111121879* | 1B | 376,791,311 | NC20 | 5.04E-04 | 2.63 |
| TGW.NC | *AX-109544098* | 1B | 377,963,460 | NC20 | 4.11E-04 | 2.72 |
| TGW.NC | *AX-109853345* | 1B | 378,461,864 | NC20 | 7.85E-04 | 2.45 |
| TGW.NC | *AX-111496509* | 1B | 378,971,833 | NC20 | 5.94E-04 | 2.57 |
| TGW.NC | *AX-109070579* | 1B | 379,583,836 | NC20 | 4.52E-04 | 2.68 |
| TGW.NC | *AX-109271826* | 1B | 380,240,446 | NC20 | 6.98E-04 | 2.50 |
| TGW.NC | *AX-111503092* | 1B | 380,647,070 | NC20 | 2.17E-04 | 2.98 |
| TGW.NC | *AX-108973265* | 1B | 381,032,751 | NC20 | 4.65E-04 | 2.67 |
| TGW.NC | *AX-109983759* | 1B | 383,176,594 | NC20 | 4.23E-04 | 2.71 |
| TGW.NC | *AX-109419267* | 1B | 384,249,145 | NC20 | 5.55E-04 | 2.59 |
| TGW.NC | *AX-108756721* | 1B | 384,566,103 | NC20 | 3.37E-04 | 2.80 |
| TGW.NC | *AX-111715015* | 1B | 385,300,997 | NC20 | 6.14E-04 | 2.55 |
| TGW.NC | *AX-111468437* | 1B | 386,240,704 | NC20 | 4.85E-04 | 2.65 |
| TGW.NC | *AX-109340517* | 1B | 386,689,382 | NC20 | 6.90E-04 | 2.50 |
| TGW.NC | *AX-109391593* | 1B | 387,288,895 | NC20 | 3.81E-04 | 2.75 |
| TGW.NC | *AX-111549107* | 1B | 388,341,838 | NC20 | 5.29E-04 | 2.61 |
| TGW.NC | *AX-111022842* | 1B | 389,321,645 | NC20 | 4.59E-04 | 2.67 |
| TGW.NC | *AX-109873586* | 1B | 389,879,147 | NC20 | 3.70E-04 | 2.76 |
| TGW.NC | *AX-111109396* | 1B | 390,276,179 | NC20 | 4.64E-04 | 2.67 |
| TGW.NC | *AX-110002681* | 1B | 391,917,068 | NC20 | 9.71E-04 | 2.36 |
| TGW.NC | *AX-109523456* | 1B | 403,418,894 | NC20 | 8.95E-04 | 2.40 |
| TGW.NC | *AX-109272373* | 1B | 404,523,501 | NC20 | 5.94E-04 | 2.57 |
| TGW.NC | *AX-111782039* | 1B | 404,883,727 | NC20 | 3.99E-04 | 2.73 |
| TGW.NC | *AX-111744000* | 1B | 405,524,154 | NC20 | 6.24E-04 | 2.55 |
| TGW.NC | *AX-110057926* | 1B | 407,869,097 | NC20 | 5.15E-04 | 2.62 |
| TGW.NC | *AX-109356137* | 1B | 409,617,898 | NC20 | 3.72E-04 | 2.76 |
| TGW.NC | *AX-111007632* | 1B | 410,299,329 | NC20 | 4.16E-04 | 2.71 |
| TGW.NC | *AX-111569795* | 1B | 410,787,194 | NC20 | 4.35E-04 | 2.69 |
| TGW.NC | *AX-111077672* | 1B | 415,335,518 | NC20 | 5.00E-04 | 2.64 |
| TGW.NC | *AX-111726835* | 1B | 417,400,703 | NC20 | 4.72E-04 | 2.66 |
| TGW.NC | *AX-110419189* | 1B | 418,160,680 | NC20 | 3.60E-04 | 2.77 |
| TGW.NC | *AX-111083420* | 1B | 418,826,302 | NC20 | 6.69E-04 | 2.52 |
| TGW.NC | *AX-108808608* | 1B | 419,407,395 | NC20 | 2.94E-04 | 2.86 |
| TGW.NC | *AX-110368439* | 1B | 419,950,198 | NC20 | 4.96E-04 | 2.64 |
| TGW.NC | *AX-111169510* | 1B | 430,155,688 | NC20 | 2.90E-04 | 2.86 |
| TGW.NC | *AX-111169510* | 1B | 430,155,688 | NC21 | 5.10E-04 | 2.39 |
| TGW.NC | *AX-111169510* | 1B | 430,155,688 | NAVE | 7.94E-04 | 2.11 |
| TGW.NC | *AX-109421320* | 1B | 431,469,851 | NC20 | 9.00E-04 | 2.40 |
| TGW.NC | *AX-110916356* | 1B | 489,129,788 | NC20 | 9.67E-04 | 2.37 |
| TGW.NC | *AX-110670988* | 1B | 489,973,360 | NC20 | 7.13E-04 | 2.49 |
| TGW.NC | *AX-94442756* | 1B | 509,901,004 | NC20 | 8.97E-04 | 2.40 |
| TGW.NC | *AX-111780371* | 1B | 510,356,945 | NC20 | 2.98E-04 | 2.85 |
| TGW.NC | *AX-108920917* | 1B | 511,050,019 | NC20 | 1.57E-04 | 3.12 |
| TGW.NC | *AX-109457059* | 1B | 511,409,502 | NC20 | 1.59E-04 | 3.11 |
| TGW.NC | *AX-109966524* | 1B | 512,107,159 | NC20 | 2.55E-04 | 2.91 |
| TGW.NC | *AX-94532894* | 1B | 512,458,040 | NC20 | 6.03E-04 | 2.56 |
| TGW.NC | *AX-109516797* | 1B | 556,006,557 | NC20 | 8.44E-04 | 2.42 |
| TGW.NC | *AX-110585482* | 1B | 656,500,596 | NC21 | 6.82E-04 | 2.28 |
| TGW.NC | *AX-108892781* | 1B | 657,274,040 | NC21 | 1.05E-04 | 2.98 |
| TGW.NC | *AX-110489420* | 1B | 663,230,030 | NC20 | 7.08E-04 | 2.49 |
| TGW.NC | *AX-109290429* | 2A | 608,865,127 | NC19 | 3.60E-04 | 3.17 |
| TGW.NC | *AX-109290429* | 2A | 608,865,127 | NC21 | 1.27E-04 | 2.91 |
| TGW.NC | *AX-109290429* | 2A | 608,865,127 | NAVE | 5.92E-04 | 2.21 |
| TGW.NC | *AX-110510308* | 2A | 611,567,265 | NC21 | 1.82E-04 | 2.77 |
| TGW.NC | *AX-86179766* | 2A | 611,946,860 | NC21 | 1.58E-04 | 2.83 |
| TGW.NC | *AX-109425314* | 2A | 693,336,312 | NC19 | 2.57E-05 | 4.44 |
| TGW.NC | *AX-94547450* | 2A | 694,333,888 | NC19 | 1.46E-04 | 3.60 |
| TGW.NC | *AX-110425132* | 2A | 705,807,076 | NC19 | 2.36E-04 | 3.37 |
| TGW.NC | *AX-110425132* | 2A | 705,807,076 | NAVE | 8.78E-04 | 2.07 |
| TGW.NC | *AX-94495090* | 2A | 747,147,132 | NC21 | 1.41E-04 | 2.87 |
| TGW.NC | *AX-109018898* | 2B | 46,116,725 | NC19 | 5.81E-04 | 2.94 |
| TGW.NC | *AX-94475621* | 2B | 594,849,586 | NC20 | 9.16E-04 | 2.39 |
| TGW.NC | *AX-109505207* | 2B | 666,474,502 | NC19 | 4.24E-04 | 3.09 |
| TGW.NC | *AX-109505207* | 2B | 666,474,502 | NC21 | 1.73E-04 | 2.79 |
| TGW.NC | *AX-109505207* | 2B | 666,474,502 | NAVE | 3.29E-05 | 3.25 |
| TGW.NC | *AX-111661669* | 2B | 666,653,511 | NC19 | 4.28E-04 | 3.09 |
| TGW.NC | *AX-111661669* | 2B | 666,653,511 | NC21 | 8.97E-05 | 3.04 |
| TGW.NC | *AX-111661669* | 2B | 666,653,511 | NAVE | 5.68E-05 | 3.05 |
| TGW.NC | *AX-111728700* | 2B | 667,415,257 | NC19 | 4.70E-04 | 3.04 |
| TGW.NC | *AX-111728700* | 2B | 667,415,257 | NC21 | 1.72E-04 | 2.79 |
| TGW.NC | *AX-111728700* | 2B | 667,415,257 | NAVE | 9.98E-05 | 2.84 |
| TGW.NC | *AX-94979731* | 2B | 667,805,353 | NC19 | 8.67E-04 | 2.76 |
| TGW.NC | *AX-94979731* | 2B | 667,805,353 | NC21 | 5.87E-05 | 3.20 |
| TGW.NC | *AX-94979731* | 2B | 667,805,353 | NAVE | 1.17E-04 | 2.79 |
| TGW.NC | *AX-95219673* | 2B | 671,741,110 | NC19 | 1.10E-04 | 3.73 |
| TGW.NC | *AX-95219673* | 2B | 671,741,110 | NC21 | 6.91E-05 | 3.14 |
| TGW.NC | *AX-95219673* | 2B | 671,741,110 | NAVE | 2.16E-05 | 3.40 |
| TGW.NC | *AX-109352432* | 2B | 731,463,570 | NC20 | 3.16E-04 | 2.83 |
| TGW.NC | *AX-109911369* | 2D | 18,201,989 | NC20 | 4.97E-04 | 2.64 |
| TGW.NC | *AX-109911369* | 2D | 18,201,989 | NAVE | 6.74E-04 | 2.16 |
| TGW.NC | *AX-86166630* | 2D | 66,759,628 | NC19 | 5.30E-04 | 2.99 |
| TGW.NC | *AX-112290193* | 2D | 83,161,369 | NC21 | 9.82E-06 | 3.89 |
| TGW.NC | *AX-89690184* | 2D | 419,865,085 | NAVE | 2.75E-04 | 2.48 |
| TGW.NC | *AX-111761440* | 2D | 609,508,490 | NC21 | 9.16E-04 | 2.17 |
| TGW.NC | *AX-108887673* | 3A | 726,511,279 | NC19 | 4.13E-04 | 3.11 |
| TGW.NC | *AX-111534973* | 3B | 41,633,676 | NC20 | 4.35E-05 | 3.66 |
| TGW.NC | *AX-111534973* | 3B | 41,633,676 | NAVE | 3.13E-04 | 2.43 |
| TGW.NC | *AX-109359639* | 3B | 123,714,815 | NC21 | 5.45E-04 | 2.36 |
| TGW.NC | *AX-89402731* | 3D | 390,036,286 | NC21 | 9.01E-04 | 2.17 |
| TGW.NC | *AX-111077580* | 4A | 25,578,658 | NC20 | 1.46E-04 | 3.15 |
| TGW.NC | *AX-110171894* | 4A | 605,753,144 | NC19 | 2.04E-04 | 3.44 |
| TGW.NC | *AX-110171894* | 4A | 605,753,144 | NC21 | 2.92E-04 | 2.59 |
| TGW.NC | *AX-109626103* | 4A | 605,764,861 | NC19 | 3.00E-04 | 3.26 |
| TGW.NC | *AX-109626103* | 4A | 605,764,861 | NC21 | 7.97E-04 | 2.22 |
| TGW.NC | *AX-108777965* | 4A | 605,767,873 | NC19 | 2.37E-04 | 3.37 |
| TGW.NC | *AX-108777965* | 4A | 605,767,873 | NC21 | 3.38E-04 | 2.54 |
| TGW.NC | *AX-110961937* | 4A | 605,785,021 | NC19 | 2.13E-04 | 3.42 |
| TGW.NC | *AX-110961937* | 4A | 605,785,021 | NC21 | 2.08E-04 | 2.72 |
| TGW.NC | *AX-110961937* | 4A | 605,785,021 | NAVE | 7.75E-04 | 2.11 |
| TGW.NC | *AX-111040045* | 4A | 632,764,370 | NAVE | 4.30E-04 | 2.32 |
| TGW.NC | *AX-109375057* | 4A | 633,308,367 | NC20 | 4.53E-04 | 2.68 |
| TGW.NC | *AX-109375057* | 4A | 633,308,367 | NC21 | 6.95E-04 | 2.27 |
| TGW.NC | *AX-109375057* | 4A | 633,308,367 | NAVE | 7.27E-05 | 2.96 |
| TGW.NC | *AX-110468999* | 4A | 634,108,610 | NC20 | 6.91E-04 | 2.50 |
| TGW.NC | *AX-110468999* | 4A | 634,108,610 | NC21 | 6.98E-04 | 2.27 |
| TGW.NC | *AX-110468999* | 4A | 634,108,610 | NAVE | 1.39E-04 | 2.72 |
| TGW.NC | *AX-111140650* | 4A | 634,368,256 | NC21 | 8.73E-04 | 2.18 |
| TGW.NC | *AX-111140650* | 4A | 634,368,256 | NAVE | 3.50E-04 | 2.39 |
| TGW.NC | *AX-111453918* | 4A | 634,833,572 | NC20 | 4.78E-04 | 2.65 |
| TGW.NC | *AX-111453918* | 4A | 634,833,572 | NC21 | 8.44E-04 | 2.20 |
| TGW.NC | *AX-111453918* | 4A | 634,833,572 | NAVE | 1.22E-04 | 2.77 |
| TGW.NC | *AX-110440161* | 4A | 635,292,292 | NC20 | 1.90E-04 | 3.04 |
| TGW.NC | *AX-110440161* | 4A | 635,292,292 | NAVE | 2.42E-04 | 2.53 |
| TGW.NC | *AX-108950546* | 4A | 635,764,214 | NC20 | 3.28E-04 | 2.81 |
| TGW.NC | *AX-108950546* | 4A | 635,764,214 | NC21 | 8.98E-04 | 2.17 |
| TGW.NC | *AX-108950546* | 4A | 635,764,214 | NAVE | 5.93E-05 | 3.03 |
| TGW.NC | *AX-94772244* | 4B | 13,079,161 | NC21 | 8.47E-04 | 2.20 |
| TGW.NC | *AX-94772244* | 4B | 13,079,161 | NAVE | 6.01E-04 | 2.20 |
| TGW.NC | *AX-95160379* | 4B | 30,589,960 | NC21 | 5.12E-05 | 3.25 |
| TGW.NC | *AX-95160379* | 4B | 30,589,960 | NAVE | 1.71E-04 | 2.65 |
| TGW.NC | *AX-110021330* | 4B | 30,863,602 | NC21 | 1.11E-04 | 2.96 |
| TGW.NC | *AX-110021330* | 4B | 30,863,602 | NAVE | 6.36E-04 | 2.18 |
| TGW.NC | *AX-109320255* | 4B | 172,526,927 | NC19 | 5.33E-04 | 2.98 |
| TGW.NC | *AX-111150060* | 4B | 660,589,159 | NC21 | 4.88E-05 | 3.27 |
| TGW.NC | *AX-110086840* | 4B | 660,666,115 | NC21 | 3.74E-04 | 2.50 |
| TGW.NC | *AX-111115088* | 5A | 536,675,666 | NC20 | 1.32E-05 | 4.16 |
| TGW.NC | *AX-110976602* | 5A | 585,742,226 | NC19 | 7.40E-05 | 3.93 |
| TGW.NC | *AX-111516865* | 5B | 693,823,642 | NAVE | 6.60E-04 | 2.17 |
| TGW.NC | *AX-89485573* | 5D | 61,460,997 | NC19 | 5.38E-04 | 2.98 |
| TGW.NC | *AX-110050695* | 5D | 63,773,557 | NC19 | 5.51E-04 | 2.97 |
| TGW.NC | *AX-110024506* | 5D | 63,783,511 | NC19 | 4.09E-04 | 3.11 |
| TGW.NC | *AX-111597176* | 5D | 70,896,874 | NC19 | 6.10E-04 | 2.92 |
| TGW.NC | *AX-109195695* | 5D | 447,900,718 | NC20 | 5.60E-04 | 2.59 |
| TGW.NC | *AX-110323751* | 5D | 485,750,527 | NC20 | 2.82E-05 | 3.84 |
| TGW.NC | *AX-108803615* | 5D | 486,092,519 | NC20 | 3.84E-05 | 3.71 |
| TGW.NC | *AX-108910713* | 5D | 486,935,936 | NC20 | 6.96E-04 | 2.50 |
| TGW.NC | *AX-94747135* | 5D | 544,279,167 | NC19 | 5.83E-04 | 2.94 |
| TGW.NC | *AX-110077933* | 6A | 496,623,764 | NC19 | 1.71E-04 | 3.52 |
| TGW.NC | *AX-108944873* | 6A | 601,258,225 | NC20 | 3.22E-04 | 2.82 |
| TGW.NC | *AX-109948773* | 6B | 571,664,657 | NC20 | 8.56E-04 | 2.42 |
| TGW.NC | *AX-111757493* | 6B | 658,833,342 | NC21 | 6.96E-04 | 2.27 |
| TGW.NC | *AX-110223575* | 6D | 202,316,296 | NC20 | 7.29E-04 | 2.48 |
| TGW.NC | *AX-108729832* | 7A | 63,056,969 | NC21 | 8.29E-04 | 2.20 |
| TGW.NC | *AX-111152764* | 7A | 81,954,432 | NC20 | 9.28E-05 | 3.34 |
| TGW.NC | *AX-109930452* | 7A | 82,305,435 | NC20 | 1.68E-04 | 3.09 |
| TGW.NC | *AX-109270297* | 7A | 201,985,415 | NC21 | 1.92E-04 | 2.75 |
| TGW.NC | *AX-111450326* | 7A | 215,177,875 | NC21 | 3.84E-04 | 2.49 |
| TGW.NC | *AX-108808481* | 7A | 218,244,507 | NC21 | 2.68E-04 | 2.63 |
| TGW.NC | *AX-110024904* | 7A | 224,492,523 | NC21 | 7.71E-04 | 2.23 |
| TGW.NC | *AX-109940210* | 7A | 644,858,583 | NC19 | 6.68E-04 | 2.88 |
| TGW.NC | *AX-109940210* | 7A | 644,858,583 | NC21 | 9.75E-04 | 2.14 |
| TGW.NC | *AX-111157953* | 7A | 646,673,200 | NC20 | 2.87E-04 | 2.87 |
| TGW.NC | *AX-111157953* | 7A | 646,673,200 | NC21 | 9.16E-04 | 2.17 |
| TGW.NC | *AX-111157953* | 7A | 646,673,200 | NAVE | 4.73E-04 | 2.29 |
| TGW.NC | *AX-108736604* | 7A | 655,403,231 | NC21 | 3.50E-04 | 2.53 |
| TGW.NC | *AX-108736604* | 7A | 655,403,231 | NAVE | 1.72E-04 | 2.65 |
| TGW.NC | *AX-109921024* | 7A | 675,593,918 | NC21 | 2.14E-04 | 2.71 |
| TGW.NC | *AX-111264354* | 7A | 677,686,885 | NC21 | 2.55E-04 | 2.64 |
| TGW.NC | *AX-109386434* | 7A | 692,683,014 | NC21 | 2.64E-04 | 2.63 |
| TGW.NC | *AX-89432708* | 7B | 701,338,884 | NC20 | 6.98E-06 | 4.44 |
| TGW.NC | *AX-89432708* | 7B | 701,338,884 | NC21 | 7.47E-04 | 2.24 |
| TGW.NC | *AX-89432708* | 7B | 701,338,884 | NAVE | 1.18E-04 | 2.78 |
| TGW.NC | *AX-111712510* | 7B | 705,737,251 | NC20 | 3.35E-04 | 2.80 |
| TGW.NC | *AX-110667549* | 7D | 91,671,945 | NC19 | 8.84E-04 | 2.75 |
| TGW.NC | *AX-94391955* | 7D | 92,570,636 | NC19 | 7.75E-05 | 3.90 |
| TGW.NC | *AX-111684409* | 7D | 94,928,642 | NC19 | 9.82E-04 | 2.70 |
| TGW.NC | *AX-109872520* | 7D | 97,305,360 | NC19 | 4.80E-04 | 3.03 |
| TGW.NC | *AX-111616426* | 7D | 112,429,226 | NC20 | 4.71E-04 | 2.66 |
| TGW.NC | *AX-108875138* | 7D | 112,949,752 | NC20 | 4.33E-04 | 2.70 |
| TGW.NC | *AX-111798949* | 7D | 545,167,822 | NC20 | 3.52E-04 | 2.78 |
| TGW.NC | *AX-111798949* | 7D | 545,167,822 | NAVE | 2.85E-04 | 2.47 |
| TGW.NC | *AX-108758043* | 7D | 547,277,450 | NC20 | 9.97E-05 | 3.31 |
| TGW.NC | *AX-108758043* | 7D | 547,277,450 | NC21 | 9.14E-04 | 2.17 |
| TGW.NC | *AX-108758043* | 7D | 547,277,450 | NAVE | 7.26E-05 | 2.96 |
| TGW.NC | *AX-110949705* | 7D | 548,055,156 | NC20 | 1.98E-04 | 3.02 |
| TGW.NC | *AX-110949705* | 7D | 548,055,156 | NAVE | 3.74E-04 | 2.37 |
| TGW.NC | *AX-108770812* | 7D | 561,926,335 | NC20 | 5.94E-04 | 2.57 |
| TGW.NC | *AX-108770812* | 7D | 561,926,335 | NC21 | 7.17E-04 | 2.26 |
| TGW.NC | *AX-108770812* | 7D | 561,926,335 | NAVE | 3.44E-05 | 3.23 |
| TGW.NC | *AX-108763519* | 7D | 572,307,546 | NC20 | 6.37E-05 | 3.49 |
| TGW.NC | *AX-109189009* | 7D | 593,701,314 | NAVE | 4.62E-04 | 2.30 |
| GL.NC | *AX-108777399* | 1A | 10,579,460 | NC19 | 3.86E-04 | 3.21 |
| GL.NC | *AX-110523831* | 1A | 15,187,341 | NC19 | 5.10E-04 | 3.07 |
| GL.NC | *AX-108966198* | 1A | 16,038,997 | NC19 | 1.92E-04 | 3.55 |
| GL.NC | *AX-110090502* | 1A | 16,407,055 | NC19 | 2.00E-04 | 3.53 |
| GL.NC | *AX-111084696* | 1A | 16,422,919 | NC19 | 3.69E-05 | 4.36 |
| GL.NC | *AX-111125144* | 1A | 18,487,694 | NC19 | 9.84E-04 | 2.76 |
| GL.NC | *AX-110525765* | 1A | 256,704,709 | NAVE | 5.51E-04 | 2.24 |
| GL.NC | *AX-109494616* | 1A | 296,216,227 | NAVE | 9.60E-04 | 2.04 |
| GL.NC | *AX-108931576* | 1A | 522,152,681 | NC20 | 7.45E-04 | 2.26 |
| GL.NC | *AX-182098040* | 1B | 42,313,047 | NC20 | 9.02E-04 | 2.19 |
| GL.NC | *AX-108774212* | 1B | 42,335,615 | NC20 | 6.05E-04 | 2.34 |
| GL.NC | *AX-108920917* | 1B | 511,050,019 | NC20 | 9.96E-04 | 2.15 |
| GL.NC | *AX-108750098* | 1B | 512,924,054 | NC21 | 3.80E-04 | 2.49 |
| GL.NC | *AX-109516797* | 1B | 556,006,557 | NC20 | 3.43E-04 | 2.55 |
| GL.NC | *AX-109903718* | 1B | 564,916,918 | NC21 | 9.68E-05 | 3.01 |
| GL.NC | *AX-109903718* | 1B | 564,916,918 | NAVE | 3.66E-04 | 2.38 |
| GL.NC | *AX-111130381* | 1B | 565,517,030 | NC20 | 4.68E-04 | 2.43 |
| GL.NC | *AX-111130381* | 1B | 565,517,030 | NC21 | 5.31E-04 | 2.37 |
| GL.NC | *AX-111130381* | 1B | 565,517,030 | NAVE | 4.60E-04 | 2.30 |
| GL.NC | *AX-94796731* | 1B | 565,672,749 | NC21 | 9.85E-04 | 2.14 |
| GL.NC | *AX-94796731* | 1B | 565,672,749 | NAVE | 7.47E-04 | 2.13 |
| GL.NC | *AX-111595814* | 1B | 568,521,078 | NAVE | 7.65E-04 | 2.12 |
| GL.NC | *AX-111011700* | 1B | 568,768,279 | NAVE | 9.67E-04 | 2.04 |
| GL.NC | *AX-111009896* | 1B | 569,506,918 | NAVE | 4.77E-04 | 2.29 |
| GL.NC | *AX-109850741* | 1B | 570,291,724 | NAVE | 7.33E-04 | 2.14 |
| GL.NC | *AX-110922932* | 1B | 570,569,224 | NC21 | 9.78E-04 | 2.14 |
| GL.NC | *AX-110922932* | 1B | 570,569,224 | NAVE | 5.77E-04 | 2.22 |
| GL.NC | *AX-109948353* | 1B | 571,073,013 | NAVE | 8.45E-04 | 2.09 |
| GL.NC | *AX-108804866* | 1B | 572,254,394 | NC21 | 7.40E-04 | 2.24 |
| GL.NC | *AX-110402488* | 1B | 580,075,805 | NC20 | 3.59E-04 | 2.53 |
| GL.NC | *AX-110402488* | 1B | 580,075,805 | NC21 | 9.32E-04 | 2.16 |
| GL.NC | *AX-110402488* | 1B | 580,075,805 | NAVE | 1.08E-04 | 2.82 |
| GL.NC | *AX-111157060* | 1B | 580,520,152 | NC20 | 7.57E-04 | 2.25 |
| GL.NC | *AX-111157060* | 1B | 580,520,152 | NAVE | 4.74E-04 | 2.29 |
| GL.NC | *AX-110359707* | 1B | 685,225,141 | NC21 | 3.81E-04 | 2.49 |
| GL.NC | *AX-110359707* | 1B | 685,225,141 | NAVE | 5.65E-04 | 2.23 |
| GL.NC | *AX-109353011* | 1B | 685,283,930 | NC20 | 4.87E-04 | 2.42 |
| GL.NC | *AX-109353011* | 1B | 685,283,930 | NC21 | 5.25E-04 | 2.37 |
| GL.NC | *AX-109353011* | 1B | 685,283,930 | NAVE | 3.31E-04 | 2.42 |
| GL.NC | *AX-108737720* | 1B | 686,751,506 | NC20 | 3.65E-04 | 2.52 |
| GL.NC | *AX-108737720* | 1B | 686,751,506 | NAVE | 8.30E-04 | 2.09 |
| GL.NC | *AX-111190944* | 1B | 686,755,725 | NC20 | 5.44E-04 | 2.38 |
| GL.NC | *AX-109847715* | 1B | 686,786,607 | NC20 | 4.18E-04 | 2.47 |
| GL.NC | *AX-109847715* | 1B | 686,786,607 | NAVE | 9.28E-04 | 2.06 |
| GL.NC | *AX-108833740* | 1B | 688,629,864 | NC19 | 9.94E-04 | 2.75 |
| GL.NC | *AX-109367382* | 1D | 206,956,737 | NAVE | 4.78E-04 | 2.29 |
| GL.NC | *AX-110530257* | 2A | 34,164,516 | NC19 | 9.57E-04 | 2.77 |
| GL.NC | *AX-108797935* | 2A | 515,308,729 | NC20 | 8.90E-04 | 2.19 |
| GL.NC | *AX-109963316* | 2A | 605,379,330 | NC19 | 5.79E-04 | 3.01 |
| GL.NC | *AX-109303155* | 2A | 605,534,683 | NC19 | 5.39E-04 | 3.05 |
| GL.NC | *AX-110451187* | 2A | 607,768,697 | NC21 | 7.55E-04 | 2.24 |
| GL.NC | *AX-109290429* | 2A | 608,865,127 | NC19 | 7.90E-05 | 3.98 |
| GL.NC | *AX-109290429* | 2A | 608,865,127 | NC21 | 1.62E-04 | 2.81 |
| GL.NC | *AX-109290429* | 2A | 608,865,127 | NAVE | 1.15E-04 | 2.80 |
| GL.NC | *AX-109425314* | 2A | 693,336,312 | NC19 | 6.38E-04 | 2.97 |
| GL.NC | *AX-110126250* | 2B | 41,440,270 | NC19 | 8.70E-04 | 2.82 |
| GL.NC | *AX-94475621* | 2B | 594,849,586 | NC20 | 8.23E-04 | 2.22 |
| GL.NC | *AX-94475621* | 2B | 594,849,586 | NAVE | 6.06E-04 | 2.21 |
| GL.NC | *AX-111491563* | 2B | 658,940,395 | NC19 | 8.69E-04 | 2.82 |
| GL.NC | *AX-110420126* | 2B | 662,317,267 | NC19 | 9.96E-04 | 2.75 |
| GL.NC | *AX-109893758* | 2B | 663,131,898 | NC19 | 6.94E-04 | 2.92 |
| GL.NC | *AX-109015706* | 2B | 664,223,432 | NC19 | 5.46E-04 | 3.04 |
| GL.NC | *AX-108980566* | 2B | 714,127,900 | NC21 | 9.68E-04 | 2.14 |
| GL.NC | *AX-94501206* | 2B | 730,998,420 | NC21 | 5.69E-04 | 2.34 |
| GL.NC | *AX-94501206* | 2B | 730,998,420 | NAVE | 2.98E-04 | 2.46 |
| GL.NC | *AX-109352432* | 2B | 731,463,570 | NC20 | 1.96E-04 | 2.76 |
| GL.NC | *AX-109352432* | 2B | 731,463,570 | NAVE | 5.92E-04 | 2.21 |
| GL.NC | *AX-111472511* | 2B | 732,339,052 | NC21 | 4.90E-04 | 2.40 |
| GL.NC | *AX-111472511* | 2B | 732,339,052 | NAVE | 2.30E-04 | 2.55 |
| GL.NC | *AX-109013500* | 2D | 63,568,198 | NC20 | 1.90E-04 | 2.77 |
| GL.NC | *AX-109013500* | 2D | 63,568,198 | NC21 | 7.59E-06 | 3.98 |
| GL.NC | *AX-109013500* | 2D | 63,568,198 | NAVE | 1.69E-05 | 3.50 |
| GL.NC | *AX-109266674* | 2D | 72,895,147 | NC21 | 2.20E-04 | 2.70 |
| GL.NC | *AX-109266674* | 2D | 72,895,147 | NAVE | 2.33E-04 | 2.55 |
| GL.NC | *AX-111559873* | 2D | 79,095,877 | NC20 | 4.71E-04 | 2.43 |
| GL.NC | *AX-111559873* | 2D | 79,095,877 | NC21 | 5.09E-05 | 3.25 |
| GL.NC | *AX-111559873* | 2D | 79,095,877 | NAVE | 2.66E-04 | 2.50 |
| GL.NC | *AX-112290158* | 2D | 79,421,800 | NC19 | 9.27E-05 | 3.90 |
| GL.NC | *AX-95631471* | 3A | 44,629,466 | NC20 | 7.85E-04 | 2.24 |
| GL.NC | *AX-95631471* | 3A | 44,629,466 | NAVE | 2.67E-04 | 2.50 |
| GL.NC | *AX-111626915* | 3A | 734,428,567 | NC20 | 7.13E-04 | 2.27 |
| GL.NC | *AX-111534973* | 3B | 41,633,676 | NC19 | 5.49E-04 | 3.04 |
| GL.NC | *AX-111534973* | 3B | 41,633,676 | NC20 | 2.76E-04 | 2.63 |
| GL.NC | *AX-111534973* | 3B | 41,633,676 | NAVE | 2.43E-04 | 2.53 |
| GL.NC | *AX-109303193* | 3B | 143,573,669 | NAVE | 9.21E-04 | 2.06 |
| GL.NC | *AX-89331046* | 3B | 781,210,302 | NC19 | 6.89E-04 | 2.93 |
| GL.NC | *AX-110418888* | 3B | 785,432,286 | NC19 | 7.06E-04 | 2.92 |
| GL.NC | *AX-110418888* | 3B | 785,432,286 | NC20 | 2.43E-04 | 2.68 |
| GL.NC | *AX-110418888* | 3B | 785,432,286 | NC21 | 5.65E-04 | 2.34 |
| GL.NC | *AX-110418888* | 3B | 785,432,286 | NAVE | 1.85E-04 | 2.63 |
| GL.NC | *AX-109881148* | 3B | 793,075,912 | NC19 | 5.23E-04 | 3.06 |
| GL.NC | *AX-109881148* | 3B | 793,075,912 | NC20 | 1.95E-04 | 2.76 |
| GL.NC | *AX-109881148* | 3B | 793,075,912 | NAVE | 2.90E-04 | 2.47 |
| GL.NC | *AX-111703658* | 3D | 32,268,623 | NAVE | 9.55E-04 | 2.05 |
| GL.NC | *AX-111103248* | 3D | 178,761,794 | NC20 | 4.47E-04 | 2.45 |
| GL.NC | *AX-111103248* | 3D | 178,761,794 | NAVE | 3.57E-04 | 2.39 |
| GL.NC | *AX-109903567* | 3D | 196,832,500 | NC20 | 9.02E-04 | 2.19 |
| GL.NC | *AX-109903567* | 3D | 196,832,500 | NAVE | 7.25E-04 | 2.14 |
| GL.NC | *AX-111778716* | 3D | 202,665,077 | NC20 | 9.27E-04 | 2.18 |
| GL.NC | *AX-111778716* | 3D | 202,665,077 | NAVE | 5.65E-04 | 2.23 |
| GL.NC | *AX-110432825* | 3D | 206,655,789 | NC20 | 3.58E-04 | 2.53 |
| GL.NC | *AX-110432825* | 3D | 206,655,789 | NAVE | 2.58E-04 | 2.51 |
| GL.NC | *AX-109341556* | 3D | 221,046,278 | NC20 | 4.47E-04 | 2.45 |
| GL.NC | *AX-109341556* | 3D | 221,046,278 | NAVE | 3.57E-04 | 2.39 |
| GL.NC | *AX-109626991* | 3D | 224,648,576 | NC20 | 8.63E-04 | 2.20 |
| GL.NC | *AX-109626991* | 3D | 224,648,576 | NAVE | 6.96E-04 | 2.16 |
| GL.NC | *AX-109392999* | 3D | 236,169,118 | NC19 | 7.81E-04 | 2.87 |
| GL.NC | *AX-109392999* | 3D | 236,169,118 | NC20 | 3.75E-04 | 2.51 |
| GL.NC | *AX-109392999* | 3D | 236,169,118 | NAVE | 2.34E-04 | 2.54 |
| GL.NC | *AX-109407590* | 3D | 246,923,598 | NC20 | 5.55E-04 | 2.37 |
| GL.NC | *AX-109407590* | 3D | 246,923,598 | NAVE | 4.26E-04 | 2.33 |
| GL.NC | *AX-110830021* | 3D | 260,816,674 | NC20 | 4.60E-04 | 2.44 |
| GL.NC | *AX-110830021* | 3D | 260,816,674 | NAVE | 3.66E-04 | 2.38 |
| GL.NC | *AX-95220409* | 3D | 467,321,045 | NC20 | 5.11E-04 | 2.40 |
| GL.NC | *AX-89666742* | 3D | 578,247,092 | NAVE | 1.73E-04 | 2.65 |
| GL.NC | *AX-111077580* | 4A | 25,578,658 | NC20 | 3.61E-05 | 3.41 |
| GL.NC | *AX-111077580* | 4A | 25,578,658 | NAVE | 4.69E-04 | 2.30 |
| GL.NC | *AX-111679727* | 4A | 97,385,587 | NC20 | 5.76E-04 | 2.35 |
| GL.NC | *AX-111587091* | 4A | 105,228,303 | NC20 | 5.05E-04 | 2.40 |
| GL.NC | *AX-110544730* | 4A | 105,593,476 | NC20 | 5.42E-04 | 2.38 |
| GL.NC | *AX-111635462* | 4A | 106,122,676 | NC20 | 8.06E-04 | 2.23 |
| GL.NC | *AX-110912744* | 4A | 107,228,194 | NC20 | 7.50E-04 | 2.25 |
| GL.NC | *AX-109581996* | 4A | 107,699,007 | NC20 | 5.06E-04 | 2.40 |
| GL.NC | *AX-109506032* | 4A | 108,192,759 | NC20 | 5.11E-04 | 2.40 |
| GL.NC | *AX-109324791* | 4A | 109,148,447 | NC20 | 7.07E-04 | 2.28 |
| GL.NC | *AX-109997716* | 4A | 109,770,546 | NC20 | 6.14E-04 | 2.33 |
| GL.NC | *AX-110552751* | 4A | 110,298,500 | NC20 | 4.64E-04 | 2.43 |
| GL.NC | *AX-109354728* | 4A | 111,268,266 | NC20 | 5.05E-04 | 2.40 |
| GL.NC | *AX-111014081* | 4A | 111,816,817 | NC20 | 5.11E-04 | 2.40 |
| GL.NC | *AX-111122406* | 4A | 112,326,134 | NC20 | 8.84E-04 | 2.19 |
| GL.NC | *AX-109330895* | 4A | 112,794,824 | NC20 | 2.54E-04 | 2.66 |
| GL.NC | *AX-109952205* | 4A | 113,320,208 | NC20 | 6.07E-04 | 2.33 |
| GL.NC | *AX-109849616* | 4A | 113,853,276 | NC20 | 5.16E-04 | 2.39 |
| GL.NC | *AX-110667940* | 4A | 114,534,224 | NC20 | 5.51E-04 | 2.37 |
| GL.NC | *AX-111067996* | 4A | 114,792,580 | NC20 | 5.29E-04 | 2.39 |
| GL.NC | *AX-111037527* | 4A | 115,569,232 | NC20 | 5.51E-04 | 2.37 |
| GL.NC | *AX-111522120* | 4A | 115,980,248 | NC20 | 7.90E-04 | 2.24 |
| GL.NC | *AX-108761059* | 4A | 117,533,026 | NC20 | 5.32E-04 | 2.38 |
| GL.NC | *AX-109559120* | 4A | 117,943,417 | NC20 | 5.08E-04 | 2.40 |
| GL.NC | *AX-111099020* | 4A | 119,067,913 | NC20 | 7.98E-04 | 2.23 |
| GL.NC | *AX-111043895* | 4A | 119,633,876 | NC20 | 5.11E-04 | 2.40 |
| GL.NC | *AX-109586259* | 4A | 120,055,385 | NC20 | 4.47E-04 | 2.45 |
| GL.NC | *AX-109012904* | 4A | 120,607,938 | NC20 | 5.11E-04 | 2.40 |
| GL.NC | *AX-108791064* | 4A | 121,149,583 | NC20 | 7.41E-04 | 2.26 |
| GL.NC | *AX-109491833* | 4A | 121,795,806 | NC20 | 5.51E-04 | 2.37 |
| GL.NC | *AX-109418813* | 4A | 122,248,852 | NC20 | 5.82E-04 | 2.35 |
| GL.NC | *AX-110039247* | 4A | 122,724,054 | NC20 | 5.51E-04 | 2.37 |
| GL.NC | *AX-109324565* | 4A | 123,277,266 | NC20 | 1.65E-04 | 2.83 |
| GL.NC | *AX-109313089* | 4A | 123,797,964 | NC20 | 7.93E-04 | 2.23 |
| GL.NC | *AX-109897504* | 4A | 124,202,338 | NC20 | 5.11E-04 | 2.40 |
| GL.NC | *AX-110448656* | 4A | 124,744,740 | NC20 | 5.46E-04 | 2.37 |
| GL.NC | *AX-108969300* | 4A | 125,356,184 | NC20 | 4.12E-04 | 2.48 |
| GL.NC | *AX-110595501* | 4A | 125,955,942 | NC20 | 6.76E-04 | 2.29 |
| GL.NC | *AX-109312810* | 4A | 126,333,228 | NC20 | 5.76E-04 | 2.35 |
| GL.NC | *AX-111034345* | 4A | 126,699,473 | NC20 | 5.46E-04 | 2.37 |
| GL.NC | *AX-110557442* | 4A | 127,182,154 | NC20 | 7.45E-04 | 2.26 |
| GL.NC | *AX-110475420* | 4A | 128,008,334 | NC20 | 4.34E-04 | 2.46 |
| GL.NC | *AX-111644366* | 4A | 128,334,402 | NC20 | 6.42E-04 | 2.31 |
| GL.NC | *AX-109928095* | 4A | 129,090,151 | NC20 | 5.51E-04 | 2.37 |
| GL.NC | *AX-109378399* | 4A | 129,442,800 | NC20 | 3.59E-04 | 2.53 |
| GL.NC | *AX-109420461* | 4A | 130,045,834 | NC20 | 5.51E-04 | 2.37 |
| GL.NC | *AX-111097741* | 4A | 130,531,560 | NC20 | 5.38E-04 | 2.38 |
| GL.NC | *AX-110189943* | 4A | 131,002,609 | NC20 | 2.93E-04 | 2.61 |
| GL.NC | *AX-109966230* | 4A | 131,483,037 | NC20 | 5.60E-04 | 2.36 |
| GL.NC | *AX-111614310* | 4A | 132,049,466 | NC20 | 4.48E-04 | 2.45 |
| GL.NC | *AX-110160936* | 4A | 132,418,119 | NC20 | 6.89E-04 | 2.29 |
| GL.NC | *AX-109912320* | 4A | 133,037,135 | NC20 | 3.31E-04 | 2.56 |
| GL.NC | *AX-111631423* | 4A | 133,490,130 | NC20 | 5.51E-04 | 2.37 |
| GL.NC | *AX-109417418* | 4A | 134,166,568 | NC20 | 4.05E-04 | 2.49 |
| GL.NC | *AX-111143353* | 4A | 135,213,716 | NC20 | 5.42E-04 | 2.38 |
| GL.NC | *AX-110955621* | 4A | 136,009,700 | NC20 | 5.51E-04 | 2.37 |
| GL.NC | *AX-111492177* | 4A | 136,791,113 | NC20 | 7.00E-04 | 2.28 |
| GL.NC | *AX-110740225* | 4A | 466,977,646 | NC20 | 2.79E-04 | 2.63 |
| GL.NC | *AX-109527362* | 4A | 467,258,017 | NC20 | 1.33E-04 | 2.91 |
| GL.NC | *AX-109527362* | 4A | 467,258,017 | NAVE | 5.24E-04 | 2.26 |
| GL.NC | *AX-111192688* | 4A | 468,154,810 | NC20 | 3.14E-04 | 2.58 |
| GL.NC | *AX-110941777* | 4A | 469,104,506 | NC20 | 2.64E-04 | 2.65 |
| GL.NC | *AX-110941777* | 4A | 469,104,506 | NAVE | 9.56E-04 | 2.05 |
| GL.NC | *AX-110457242* | 4A | 470,805,587 | NC20 | 2.70E-04 | 2.64 |
| GL.NC | *AX-109547195* | 4A | 471,936,748 | NC20 | 4.53E-04 | 2.44 |
| GL.NC | *AX-110517615* | 4A | 477,578,034 | NC20 | 3.63E-04 | 2.53 |
| GL.NC | *AX-109490376* | 4A | 482,871,379 | NC20 | 2.16E-04 | 2.72 |
| GL.NC | *AX-109490376* | 4A | 482,871,379 | NAVE | 8.40E-04 | 2.09 |
| GL.NC | *AX-109500638* | 4A | 483,900,334 | NC20 | 1.84E-04 | 2.78 |
| GL.NC | *AX-109500638* | 4A | 483,900,334 | NAVE | 8.62E-04 | 2.08 |
| GL.NC | *AX-109307896* | 4A | 484,860,570 | NC20 | 3.52E-04 | 2.54 |
| GL.NC | *AX-108898942* | 4A | 488,114,645 | NC20 | 4.43E-04 | 2.45 |
| GL.NC | *AX-110016016* | 4A | 488,553,399 | NC20 | 2.82E-04 | 2.62 |
| GL.NC | *AX-110016016* | 4A | 488,553,399 | NAVE | 8.93E-04 | 2.07 |
| GL.NC | *AX-109509506* | 4A | 488,867,412 | NC20 | 4.25E-04 | 2.47 |
| GL.NC | *AX-110468887* | 4A | 491,976,508 | NC20 | 2.34E-04 | 2.69 |
| GL.NC | *AX-110468887* | 4A | 491,976,508 | NAVE | 1.99E-04 | 2.60 |
| GL.NC | *AX-111743032* | 4A | 492,525,624 | NC20 | 2.67E-04 | 2.64 |
| GL.NC | *AX-111743032* | 4A | 492,525,624 | NAVE | 2.50E-04 | 2.52 |
| GL.NC | *AX-109563449* | 4A | 493,613,472 | NC20 | 2.05E-04 | 2.74 |
| GL.NC | *AX-109563449* | 4A | 493,613,472 | NAVE | 6.46E-04 | 2.18 |
| GL.NC | *AX-111528491* | 4A | 496,110,069 | NC20 | 3.19E-04 | 2.58 |
| GL.NC | *AX-111528491* | 4A | 496,110,069 | NAVE | 2.21E-04 | 2.56 |
| GL.NC | *AX-111451343* | 4A | 499,611,798 | NC20 | 4.01E-04 | 2.49 |
| GL.NC | *AX-111451343* | 4A | 499,611,798 | NAVE | 2.28E-04 | 2.55 |
| GL.NC | *AX-108870577* | 4A | 500,119,543 | NC20 | 2.34E-04 | 2.69 |
| GL.NC | *AX-108870577* | 4A | 500,119,543 | NAVE | 1.21E-04 | 2.78 |
| GL.NC | *AX-109398960* | 4A | 508,987,930 | NC20 | 2.91E-04 | 2.61 |
| GL.NC | *AX-109398960* | 4A | 508,987,930 | NAVE | 8.20E-04 | 2.10 |
| GL.NC | *AX-110931508* | 4A | 632,242,065 | NAVE | 1.84E-04 | 2.63 |
| GL.NC | *AX-111040045* | 4A | 632,764,370 | NAVE | 4.28E-04 | 2.33 |
| GL.NC | *AX-109375057* | 4A | 633,308,367 | NAVE | 3.35E-04 | 2.42 |
| GL.NC | *AX-110468999* | 4A | 634,108,610 | NC20 | 8.62E-04 | 2.20 |
| GL.NC | *AX-110468999* | 4A | 634,108,610 | NAVE | 3.81E-04 | 2.37 |
| GL.NC | *AX-111140650* | 4A | 634,368,256 | NAVE | 6.12E-04 | 2.20 |
| GL.NC | *AX-111453918* | 4A | 634,833,572 | NC20 | 9.35E-04 | 2.17 |
| GL.NC | *AX-111453918* | 4A | 634,833,572 | NAVE | 3.55E-04 | 2.40 |
| GL.NC | *AX-108950546* | 4A | 635,764,214 | NAVE | 3.91E-04 | 2.36 |
| GL.NC | *AX-111050965* | 4A | 703,966,317 | NC21 | 3.53E-04 | 2.52 |
| GL.NC | *AX-108747090* | 4A | 708,706,473 | NC21 | 5.60E-04 | 2.35 |
| GL.NC | *AX-111074167* | 4B | 51,188,028 | NC21 | 3.15E-04 | 2.56 |
| GL.NC | *AX-111517964* | 4B | 51,191,177 | NC21 | 3.21E-04 | 2.55 |
| GL.NC | *AX-108772683* | 4B | 53,861,811 | NC21 | 3.45E-04 | 2.53 |
| GL.NC | *AX-109580890* | 4B | 57,481,628 | NC21 | 1.41E-04 | 2.86 |
| GL.NC | *AX-110436979* | 4B | 73,152,359 | NC19 | 5.00E-04 | 3.08 |
| GL.NC | *AX-110436979* | 4B | 73,152,359 | NC21 | 1.60E-04 | 2.82 |
| GL.NC | *AX-110361956* | 4B | 75,741,547 | NC19 | 4.37E-04 | 3.15 |
| GL.NC | *AX-110361956* | 4B | 75,741,547 | NC21 | 1.89E-04 | 2.75 |
| GL.NC | *AX-110361956* | 4B | 75,741,547 | NAVE | 8.06E-04 | 2.10 |
| GL.NC | *AX-110924859* | 4B | 417,138,322 | NC20 | 4.38E-05 | 3.33 |
| GL.NC | *AX-111150060* | 4B | 660,589,159 | NC19 | 1.54E-04 | 3.65 |
| GL.NC | *AX-108765521* | 4B | 664,643,448 | NC21 | 2.90E-04 | 2.59 |
| GL.NC | *AX-95120271* | 4D | 4,132,769 | NC21 | 5.77E-04 | 2.34 |
| GL.NC | *AX-95120271* | 4D | 4,132,769 | NAVE | 5.73E-04 | 2.23 |
| GL.NC | *AX-95081842* | 4D | 109,216,418 | NC20 | 2.38E-04 | 2.69 |
| GL.NC | *AX-95081842* | 4D | 109,216,418 | NAVE | 8.82E-04 | 2.07 |
| GL.NC | *AX-108806718* | 5A | 435,245,426 | NC20 | 3.60E-04 | 2.53 |
| GL.NC | *AX-108761227* | 5A | 631,400,913 | NC19 | 4.04E-04 | 3.19 |
| GL.NC | *AX-109451992* | 5A | 663,368,927 | NC20 | 4.31E-04 | 2.46 |
| GL.NC | *AX-109451992* | 5A | 663,368,927 | NAVE | 8.17E-04 | 2.10 |
| GL.NC | *AX-110398218* | 5B | 57,493,343 | NC19 | 4.65E-04 | 3.12 |
| GL.NC | *AX-110398218* | 5B | 57,493,343 | NC21 | 6.39E-04 | 2.30 |
| GL.NC | *AX-110398218* | 5B | 57,493,343 | NAVE | 1.09E-04 | 2.82 |
| GL.NC | *AX-110538245* | 5B | 169,746,684 | NAVE | 9.84E-04 | 2.04 |
| GL.NC | *AX-110574387* | 5B | 173,399,995 | NAVE | 7.62E-04 | 2.12 |
| GL.NC | *AX-109363015* | 5B | 174,925,421 | NC19 | 2.43E-04 | 3.43 |
| GL.NC | *AX-111286497* | 5B | 178,095,746 | NAVE | 4.78E-04 | 2.29 |
| GL.NC | *AX-110195720* | 5B | 179,792,373 | NAVE | 8.79E-04 | 2.07 |
| GL.NC | *AX-109932243* | 5B | 182,802,694 | NAVE | 7.27E-04 | 2.14 |
| GL.NC | *AX-108863977* | 5B | 188,873,696 | NC19 | 3.17E-04 | 3.30 |
| GL.NC | *AX-95629151* | 5B | 206,798,948 | NAVE | 9.80E-04 | 2.04 |
| GL.NC | *AX-111551138* | 5B | 208,118,717 | NAVE | 6.25E-04 | 2.19 |
| GL.NC | *AX-108739557* | 5B | 214,182,236 | NAVE | 5.22E-04 | 2.26 |
| GL.NC | *AX-108937379* | 5B | 216,860,872 | NAVE | 7.09E-04 | 2.15 |
| GL.NC | *AX-111013279* | 5B | 219,279,347 | NAVE | 7.62E-04 | 2.12 |
| GL.NC | *AX-109293799* | 5B | 219,989,467 | NC21 | 6.53E-04 | 2.29 |
| GL.NC | *AX-109293799* | 5B | 219,989,467 | NAVE | 1.99E-04 | 2.60 |
| GL.NC | *AX-111517432* | 5B | 220,997,285 | NAVE | 7.15E-04 | 2.15 |
| GL.NC | *AX-111619112* | 5B | 226,052,844 | NAVE | 6.04E-04 | 2.21 |
| GL.NC | *AX-110735496* | 5B | 226,530,925 | NAVE | 8.96E-04 | 2.07 |
| GL.NC | *AX-108890871* | 5B | 227,344,532 | NAVE | 9.39E-04 | 2.05 |
| GL.NC | *AX-110639732* | 5B | 228,815,862 | NC19 | 3.15E-04 | 3.31 |
| GL.NC | *AX-108939406* | 5B | 229,248,808 | NAVE | 9.07E-04 | 2.06 |
| GL.NC | *AX-109101238* | 5B | 229,964,241 | NAVE | 7.97E-04 | 2.11 |
| GL.NC | *AX-111622763* | 5B | 231,352,724 | NAVE | 4.60E-04 | 2.30 |
| GL.NC | *AX-110494074* | 5B | 231,945,840 | NAVE | 8.83E-04 | 2.07 |
| GL.NC | *AX-111569451* | 5B | 233,491,515 | NC19 | 2.85E-04 | 3.36 |
| GL.NC | *AX-111533693* | 5B | 233,924,727 | NAVE | 6.34E-04 | 2.19 |
| GL.NC | *AX-111454697* | 5B | 234,774,340 | NC20 | 8.54E-04 | 2.21 |
| GL.NC | *AX-111454697* | 5B | 234,774,340 | NAVE | 3.66E-04 | 2.38 |
| GL.NC | *AX-110514822* | 5B | 235,177,818 | NAVE | 8.10E-04 | 2.10 |
| GL.NC | *AX-110688632* | 5B | 235,264,733 | NAVE | 8.14E-04 | 2.10 |
| GL.NC | *AX-108728380* | 5B | 236,197,278 | NAVE | 5.88E-04 | 2.22 |
| GL.NC | *AX-110554359* | 5B | 237,169,066 | NAVE | 7.97E-04 | 2.11 |
| GL.NC | *AX-111597396* | 5B | 237,711,410 | NAVE | 9.01E-04 | 2.07 |
| GL.NC | *AX-108851808* | 5B | 238,055,872 | NAVE | 8.27E-04 | 2.10 |
| GL.NC | *AX-108955580* | 5B | 238,709,388 | NC20 | 8.51E-04 | 2.21 |
| GL.NC | *AX-108955580* | 5B | 238,709,388 | NAVE | 3.82E-04 | 2.37 |
| GL.NC | *AX-111563063* | 5B | 239,959,069 | NC19 | 1.90E-04 | 3.55 |
| GL.NC | *AX-111667993* | 5B | 240,977,879 | NAVE | 8.06E-04 | 2.11 |
| GL.NC | *AX-109289769* | 5B | 241,602,012 | NAVE | 5.18E-04 | 2.26 |
| GL.NC | *AX-110376465* | 5B | 241,913,753 | NAVE | 7.76E-04 | 2.12 |
| GL.NC | *AX-110002541* | 5B | 242,705,963 | NC20 | 9.55E-04 | 2.16 |
| GL.NC | *AX-109321092* | 5B | 243,404,910 | NAVE | 7.87E-04 | 2.11 |
| GL.NC | *AX-111451165* | 5B | 244,757,341 | NC19 | 1.11E-04 | 3.82 |
| GL.NC | *AX-110730995* | 5B | 249,843,782 | NC19 | 3.59E-04 | 3.24 |
| GL.NC | *AX-109581524* | 5B | 250,568,458 | NC19 | 5.95E-04 | 3.00 |
| GL.NC | *AX-110020680* | 5B | 251,008,827 | NC19 | 2.64E-04 | 3.39 |
| GL.NC | *AX-111469464* | 5B | 254,974,932 | NC19 | 4.35E-04 | 3.15 |
| GL.NC | *AX-111031509* | 5B | 260,577,074 | NC19 | 2.64E-04 | 3.39 |
| GL.NC | *AX-110558483* | 5B | 262,756,054 | NC19 | 4.85E-04 | 3.10 |
| GL.NC | *AX-110624064* | 5B | 263,932,139 | NC19 | 5.31E-04 | 3.05 |
| GL.NC | *AX-110529376* | 5B | 264,839,983 | NC19 | 3.85E-04 | 3.21 |
| GL.NC | *AX-108770298* | 5B | 270,679,020 | NAVE | 9.86E-04 | 2.03 |
| GL.NC | *AX-110516200* | 5B | 277,898,057 | NC20 | 3.59E-04 | 2.53 |
| GL.NC | *AX-110516200* | 5B | 277,898,057 | NC21 | 9.51E-04 | 2.15 |
| GL.NC | *AX-110516200* | 5B | 277,898,057 | NAVE | 1.65E-04 | 2.67 |
| GL.NC | *AX-111505214* | 5B | 287,797,121 | NC21 | 8.57E-04 | 2.19 |
| GL.NC | *AX-109928742* | 5B | 394,970,067 | NC20 | 2.42E-04 | 2.68 |
| GL.NC | *AX-109928742* | 5B | 394,970,067 | NAVE | 1.55E-04 | 2.69 |
| GL.NC | *AX-109348429* | 5B | 404,478,483 | NC20 | 2.29E-04 | 2.70 |
| GL.NC | *AX-109348429* | 5B | 404,478,483 | NAVE | 2.16E-04 | 2.57 |
| GL.NC | *AX-109825450* | 5B | 404,992,049 | NC20 | 3.55E-04 | 2.54 |
| GL.NC | *AX-109825450* | 5B | 404,992,049 | NAVE | 1.83E-04 | 2.63 |
| GL.NC | *AX-111697598* | 5B | 405,520,813 | NC20 | 2.63E-04 | 2.65 |
| GL.NC | *AX-111697598* | 5B | 405,520,813 | NAVE | 1.71E-04 | 2.66 |
| GL.NC | *AX-109284684* | 5B | 418,309,355 | NAVE | 4.15E-04 | 2.34 |
| GL.NC | *AX-111504233* | 5B | 682,168,775 | NAVE | 6.40E-04 | 2.19 |
| GL.NC | *AX-109879806* | 5B | 699,999,185 | NC19 | 7.18E-04 | 2.91 |
| GL.NC | *AX-109879806* | 5B | 699,999,185 | NAVE | 8.33E-04 | 2.09 |
| GL.NC | *AX-108757760* | 5B | 701,544,672 | NC19 | 1.41E-04 | 3.70 |
| GL.NC | *AX-108757760* | 5B | 701,544,672 | NAVE | 5.60E-04 | 2.23 |
| GL.NC | *AX-108733256* | 5B | 702,228,057 | NC19 | 1.03E-04 | 3.85 |
| GL.NC | *AX-108733256* | 5B | 702,228,057 | NC20 | 9.13E-04 | 2.18 |
| GL.NC | *AX-108733256* | 5B | 702,228,057 | NAVE | 3.81E-04 | 2.37 |
| GL.NC | *AX-110946146* | 5B | 703,062,745 | NC19 | 2.84E-04 | 3.36 |
| GL.NC | *AX-110946146* | 5B | 703,062,745 | NAVE | 9.03E-04 | 2.07 |
| GL.NC | *AX-108930462* | 5D | 40,914,557 | NC20 | 3.45E-04 | 2.55 |
| GL.NC | *AX-110379095* | 5D | 193,521,361 | NC19 | 8.58E-04 | 2.82 |
| GL.NC | *AX-111739361* | 5D | 244,617,835 | NC20 | 3.50E-04 | 2.54 |
| GL.NC | *AX-111739361* | 5D | 244,617,835 | NC21 | 8.67E-04 | 2.18 |
| GL.NC | *AX-111739361* | 5D | 244,617,835 | NAVE | 2.05E-04 | 2.59 |
| GL.NC | *AX-110503408* | 5D | 379,836,128 | NC20 | 3.50E-04 | 2.54 |
| GL.NC | *AX-110503408* | 5D | 379,836,128 | NC21 | 6.97E-04 | 2.27 |
| GL.NC | *AX-110503408* | 5D | 379,836,128 | NAVE | 8.53E-05 | 2.91 |
| GL.NC | *AX-110761858* | 5D | 430,322,682 | NC20 | 3.79E-04 | 2.51 |
| GL.NC | *AX-110035093* | 5D | 464,129,291 | NAVE | 9.71E-04 | 2.04 |
| GL.NC | *AX-111499587* | 6A | 23,245,688 | NC21 | 2.74E-04 | 2.61 |
| GL.NC | *AX-111032002* | 6A | 23,867,962 | NC21 | 2.86E-04 | 2.60 |
| GL.NC | *AX-109850660* | 6A | 24,331,268 | NC21 | 2.58E-04 | 2.64 |
| GL.NC | *AX-108876438* | 6A | 24,584,124 | NC21 | 3.58E-04 | 2.51 |
| GL.NC | *AX-109302980* | 6A | 500,099,529 | NC21 | 7.95E-04 | 2.22 |
| GL.NC | *AX-110492067* | 6A | 500,363,969 | NC21 | 7.79E-04 | 2.22 |
| GL.NC | *AX-110936060* | 6A | 501,556,986 | NC21 | 5.77E-04 | 2.34 |
| GL.NC | *AX-94818186* | 6A | 501,801,411 | NC21 | 6.49E-04 | 2.29 |
| GL.NC | *AX-110451033* | 6A | 502,889,163 | NC21 | 6.78E-04 | 2.28 |
| GL.NC | *AX-108944873* | 6A | 601,258,225 | NC20 | 2.97E-04 | 2.60 |
| GL.NC | *AX-108944873* | 6A | 601,258,225 | NAVE | 3.88E-04 | 2.36 |
| GL.NC | *AX-111034352* | 6A | 603,563,082 | NC20 | 2.70E-04 | 2.64 |
| GL.NC | *AX-94788907* | 6A | 604,107,719 | NC20 | 3.01E-04 | 2.60 |
| GL.NC | *AX-110939712* | 6A | 607,955,281 | NC20 | 4.40E-04 | 2.45 |
| GL.NC | *AX-94687055* | 6B | 41,703,860 | NC21 | 1.54E-04 | 2.83 |
| GL.NC | *AX-109935603* | 6B | 153,407,026 | NC21 | 3.14E-04 | 2.56 |
| GL.NC | *AX-109935603* | 6B | 153,407,026 | NAVE | 2.78E-04 | 2.48 |
| GL.NC | *AX-109294614* | 6B | 153,635,489 | NC21 | 9.70E-04 | 2.14 |
| GL.NC | *AX-109294614* | 6B | 153,635,489 | NAVE | 8.56E-04 | 2.08 |
| GL.NC | *AX-109324368* | 6B | 681,024,809 | NC21 | 7.42E-04 | 2.24 |
| GL.NC | *AX-94875830* | 6D | 452,632,659 | NC21 | 4.18E-04 | 2.46 |
| GL.NC | *AX-108820762* | 7A | 68,319,324 | NC20 | 9.12E-04 | 2.18 |
| GL.NC | *AX-109420173* | 7A | 71,956,475 | NC20 | 9.41E-04 | 2.17 |
| GL.NC | *AX-108739742* | 7A | 72,832,150 | NC20 | 7.65E-04 | 2.25 |
| GL.NC | *AX-109360436* | 7A | 573,673,654 | NC20 | 7.92E-04 | 2.23 |
| GL.NC | *AX-110430243* | 7A | 670,500,650 | NAVE | 7.54E-04 | 2.13 |
| GL.NC | *AX-109386434* | 7A | 692,683,014 | NC21 | 2.83E-04 | 2.60 |
| GL.NC | *AX-109386434* | 7A | 692,683,014 | NAVE | 6.01E-04 | 2.21 |
| GL.NC | *AX-109320449* | 7A | 702,206,148 | NC19 | 8.41E-04 | 2.83 |
| GL.NC | *AX-108964908* | 7A | 702,619,923 | NC19 | 8.25E-04 | 2.84 |
| GL.NC | *AX-110476160* | 7A | 702,995,726 | NC19 | 4.51E-04 | 3.13 |
| GL.NC | *AX-109482315* | 7A | 703,436,711 | NC19 | 8.56E-04 | 2.82 |
| GL.NC | *AX-108952280* | 7A | 704,616,544 | NC19 | 8.56E-04 | 2.82 |
| GL.NC | *AX-109272527* | 7A | 731,240,127 | NAVE | 8.75E-04 | 2.08 |
| GL.NC | *AX-111036456* | 7B | 17,657,111 | NAVE | 7.18E-04 | 2.15 |
| GL.NC | *AX-110632991* | 7B | 483,533,955 | NC19 | 4.73E-04 | 3.11 |
| GL.NC | *AX-89432708* | 7B | 701,338,884 | NC20 | 9.50E-04 | 2.17 |
| GL.NC | *AX-109780353* | 7D | 93,701,787 | NC21 | 9.89E-04 | 2.14 |
| GL.NC | *AX-109780353* | 7D | 93,701,787 | NAVE | 1.48E-04 | 2.71 |
| GL.NC | *AX-108895252* | 7D | 122,838,225 | NC21 | 2.12E-04 | 2.71 |
| GL.NC | *AX-108895252* | 7D | 122,838,225 | NAVE | 3.95E-05 | 3.19 |
| GL.NC | *AX-108770812* | 7D | 561,926,335 | NC21 | 8.00E-04 | 2.21 |
| GL.NC | *AX-108763519* | 7D | 572,307,546 | NC20 | 7.33E-04 | 2.26 |
| GW.NC | *AX-110597141* | 1B | 368,675,675 | NC20 | 8.87E-04 | 2.21 |
| GW.NC | *AX-108845851* | 1B | 371,140,782 | NC20 | 8.15E-04 | 2.24 |
| GW.NC | *AX-111503092* | 1B | 380,647,070 | NC20 | 8.49E-04 | 2.22 |
| GW.NC | *AX-111782039* | 1B | 404,883,727 | NC20 | 9.04E-04 | 2.20 |
| GW.NC | *AX-109356137* | 1B | 409,617,898 | NC20 | 9.59E-04 | 2.18 |
| GW.NC | *AX-109457059* | 1B | 511,409,502 | NC20 | 8.48E-04 | 2.23 |
| GW.NC | *AX-110946129* | 2A | 738,776,071 | NC20 | 9.50E-04 | 2.18 |
| GW.NC | *AX-86177963* | 2B | 110,818,850 | NAVE | 2.81E-04 | 2.56 |
| GW.NC | *AX-109505207* | 2B | 666,474,502 | NC19 | 4.55E-04 | 3.18 |
| GW.NC | *AX-109505207* | 2B | 666,474,502 | NAVE | 3.62E-04 | 2.47 |
| GW.NC | *AX-111661669* | 2B | 666,653,511 | NC19 | 3.42E-04 | 3.32 |
| GW.NC | *AX-111728700* | 2B | 667,415,257 | NC19 | 5.37E-04 | 3.10 |
| GW.NC | *AX-111728700* | 2B | 667,415,257 | NAVE | 7.28E-04 | 2.21 |
| GW.NC | *AX-94979731* | 2B | 667,805,353 | NAVE | 5.06E-04 | 2.35 |
| GW.NC | *AX-95219673* | 2B | 671,741,110 | NC19 | 2.31E-04 | 3.51 |
| GW.NC | *AX-95219673* | 2B | 671,741,110 | NAVE | 2.47E-04 | 2.61 |
| GW.NC | *AX-112290193* | 2D | 83,161,369 | NC21 | 4.14E-04 | 2.55 |
| GW.NC | *AX-109822302* | 3B | 684,410,865 | NC19 | 8.71E-04 | 2.86 |
| GW.NC | *AX-109436690* | 3B | 685,018,261 | NC21 | 3.17E-04 | 2.65 |
| GW.NC | *AX-109436690* | 3B | 685,018,261 | NAVE | 6.66E-04 | 2.25 |
| GW.NC | *AX-111473006* | 3B | 707,929,828 | NC21 | 7.22E-04 | 2.34 |
| GW.NC | *AX-110589424* | 3B | 708,910,315 | NC19 | 9.34E-04 | 2.83 |
| GW.NC | *AX-111077580* | 4A | 25,578,658 | NC20 | 6.07E-04 | 2.35 |
| GW.NC | *AX-95629274* | 4A | 38,367,204 | NC19 | 2.82E-04 | 3.41 |
| GW.NC | *AX-95629274* | 4A | 38,367,204 | NC21 | 9.10E-04 | 2.25 |
| GW.NC | *AX-95629274* | 4A | 38,367,204 | NAVE | 1.08E-04 | 2.92 |
| GW.NC | *AX-89739538* | 4A | 40,443,801 | NC21 | 5.51E-04 | 2.44 |
| GW.NC | *AX-111122581* | 4A | 41,007,610 | NC21 | 2.01E-04 | 2.83 |
| GW.NC | *AX-95142576* | 4A | 43,365,322 | NC21 | 8.37E-04 | 2.28 |
| GW.NC | *AX-95142576* | 4A | 43,365,322 | NAVE | 8.07E-04 | 2.18 |
| GW.NC | *AX-108950546* | 4A | 635,764,214 | NAVE | 9.12E-04 | 2.13 |
| GW.NC | *AX-109320255* | 4B | 172,526,927 | NC19 | 3.79E-04 | 3.27 |
| GW.NC | *AX-109415660* | 4B | 278,409,046 | NC19 | 1.28E-04 | 3.81 |
| GW.NC | *AX-108847266* | 4B | 429,339,058 | NC21 | 1.83E-04 | 2.87 |
| GW.NC | *AX-108847266* | 4B | 429,339,058 | NAVE | 1.79E-04 | 2.73 |
| GW.NC | *AX-110693775* | 4B | 535,364,998 | NAVE | 9.61E-04 | 2.11 |
| GW.NC | *AX-111535446* | 4B | 536,101,581 | NAVE | 7.65E-04 | 2.20 |
| GW.NC | *AX-109382842* | 4B | 536,427,404 | NAVE | 6.68E-04 | 2.25 |
| GW.NC | *AX-111123332* | 4B | 537,537,305 | NAVE | 6.54E-04 | 2.25 |
| GW.NC | *AX-109834673* | 4B | 537,800,638 | NAVE | 4.98E-04 | 2.35 |
| GW.NC | *AX-109350990* | 4B | 538,607,209 | NAVE | 4.75E-04 | 2.37 |
| GW.NC | *AX-86174001* | 4B | 538,997,269 | NAVE | 6.03E-04 | 2.28 |
| GW.NC | *AX-110563308* | 5A | 17,300,681 | NAVE | 5.15E-04 | 2.34 |
| GW.NC | *AX-109422629* | 5A | 513,845,754 | NAVE | 8.37E-04 | 2.16 |
| GW.NC | *AX-111115088* | 5A | 536,675,666 | NC20 | 6.56E-05 | 3.20 |
| GW.NC | *AX-110976602* | 5A | 585,742,226 | NC19 | 2.86E-04 | 3.41 |
| GW.NC | *AX-109307624* | 5B | 490,992,801 | NC19 | 9.93E-04 | 2.80 |
| GW.NC | *AX-111516865* | 5B | 693,823,642 | NC19 | 6.09E-04 | 3.04 |
| GW.NC | *AX-111516865* | 5B | 693,823,642 | NAVE | 4.47E-04 | 2.39 |
| GW.NC | *AX-89485573* | 5D | 61,460,997 | NC19 | 5.90E-04 | 3.05 |
| GW.NC | *AX-110050695* | 5D | 63,773,557 | NC19 | 6.03E-04 | 3.04 |
| GW.NC | *AX-110024506* | 5D | 63,783,511 | NC19 | 4.63E-04 | 3.17 |
| GW.NC | *AX-110323751* | 5D | 485,750,527 | NC20 | 5.14E-04 | 2.41 |
| GW.NC | *AX-108803615* | 5D | 486,092,519 | NC20 | 4.56E-04 | 2.46 |
| GW.NC | *AX-109861425* | 5D | 488,693,044 | NC20 | 2.80E-04 | 2.64 |
| GW.NC | *AX-94747135* | 5D | 544,279,167 | NC19 | 5.54E-04 | 3.08 |
| GW.NC | *AX-111100875* | 5D | 560,509,338 | NAVE | 3.35E-04 | 2.50 |
| GW.NC | *AX-108836992* | 6A | 84,968,548 | NC20 | 6.24E-04 | 2.34 |
| GW.NC | *AX-109859002* | 6A | 537,250,156 | NC21 | 9.64E-04 | 2.22 |
| GW.NC | *AX-110645584* | 6A | 550,278,123 | NC21 | 9.15E-04 | 2.24 |
| GW.NC | *AX-110431275* | 6A | 571,054,819 | NC19 | 4.59E-04 | 3.17 |
| GW.NC | *AX-111152764* | 7A | 81,954,432 | NC20 | 7.67E-04 | 2.26 |
| GW.NC | *AX-109930452* | 7A | 82,305,435 | NC20 | 7.37E-04 | 2.28 |
| GW.NC | *AX-109940210* | 7A | 644,858,583 | NC19 | 4.61E-04 | 3.17 |
| GW.NC | *AX-110009621* | 7B | 716,373,353 | NC19 | 5.79E-04 | 3.06 |
| GW.NC | *AX-110006913* | 7B | 716,378,192 | NC19 | 7.43E-04 | 2.94 |
| GW.NC | *AX-108787075* | 7B | 732,644,400 | NC20 | 4.73E-04 | 2.45 |
| GW.NC | *AX-94391955* | 7D | 92,570,636 | NC19 | 1.00E-04 | 3.93 |
| GW.NC | *AX-95217260* | 7D | 618,891,392 | NC19 | 2.37E-04 | 3.50 |
| SL.NC | *AX-110581950* | 1B | 654,866,336 | NC21 | 5.23E-04 | 2.44 |
| SL.NC | *AX-110581950* | 1B | 654,866,336 | NAVE | 9.47E-04 | 2.17 |
| SL.NC | *AX-109109358* | 1B | 655,324,737 | NC21 | 2.06E-04 | 2.80 |
| SL.NC | *AX-109109358* | 1B | 655,324,737 | NAVE | 8.57E-04 | 2.21 |
| SL.NC | *AX-110602251* | 1B | 655,865,136 | NC20 | 6.84E-04 | 2.29 |
| SL.NC | *AX-110585482* | 1B | 656,500,596 | NC21 | 4.24E-04 | 2.52 |
| SL.NC | *AX-94417025* | 2A | 38,515,173 | NC19 | 6.47E-04 | 2.39 |
| SL.NC | *AX-111132810* | 2A | 42,155,602 | NC19 | 6.43E-04 | 2.40 |
| SL.NC | *AX-111132810* | 2A | 42,155,602 | NAVE | 4.61E-04 | 2.44 |
| SL.NC | *AX-108747282* | 2A | 46,071,413 | NC19 | 3.04E-04 | 2.69 |
| SL.NC | *AX-108747282* | 2A | 46,071,413 | NAVE | 8.88E-04 | 2.20 |
| SL.NC | *AX-108747720* | 2A | 58,539,285 | NC19 | 5.90E-05 | 3.34 |
| SL.NC | *AX-108747720* | 2A | 58,539,285 | NAVE | 3.74E-04 | 2.52 |
| SL.NC | *AX-110643098* | 2A | 75,645,645 | NC19 | 7.59E-04 | 2.33 |
| SL.NC | *AX-94423221* | 2A | 88,983,810 | NC20 | 8.34E-06 | 3.98 |
| SL.NC | *AX-108736564* | 2A | 89,158,728 | NC20 | 8.28E-04 | 2.22 |
| SL.NC | *AX-110522750* | 2A | 89,734,060 | NC20 | 8.34E-06 | 3.98 |
| SL.NC | *AX-110571705* | 2A | 90,218,965 | NC20 | 1.28E-05 | 3.81 |
| SL.NC | *AX-108737030* | 2A | 90,748,859 | NC20 | 2.69E-05 | 3.52 |
| SL.NC | *AX-95209305* | 2A | 92,314,368 | NC20 | 7.17E-06 | 4.04 |
| SL.NC | *AX-95209305* | 2A | 92,314,368 | NAVE | 7.47E-04 | 2.26 |
| SL.NC | *AX-108880717* | 2A | 94,337,951 | NC20 | 7.56E-06 | 4.02 |
| SL.NC | *AX-108880717* | 2A | 94,337,951 | NAVE | 8.90E-04 | 2.20 |
| SL.NC | *AX-110410792* | 2A | 503,441,606 | NC21 | 4.90E-04 | 2.46 |
| SL.NC | *AX-109452286* | 2A | 525,028,734 | NC21 | 3.02E-04 | 2.65 |
| SL.NC | *AX-109304879* | 2A | 542,733,297 | NC21 | 5.44E-04 | 2.42 |
| SL.NC | *AX-109419208* | 2A | 555,886,644 | NC21 | 9.93E-04 | 2.20 |
| SL.NC | *AX-109374842* | 2A | 557,501,278 | NC21 | 3.42E-04 | 2.60 |
| SL.NC | *AX-110370613* | 2A | 558,536,561 | NC21 | 4.88E-04 | 2.47 |
| SL.NC | *AX-111473626* | 2A | 560,214,941 | NC21 | 3.16E-04 | 2.63 |
| SL.NC | *AX-109376020* | 2A | 560,690,433 | NC21 | 9.46E-04 | 2.21 |
| SL.NC | *AX-110575088* | 2A | 561,261,264 | NC21 | 4.14E-04 | 2.53 |
| SL.NC | *AX-111546236* | 2A | 561,592,294 | NC21 | 4.86E-04 | 2.47 |
| SL.NC | *AX-111543324* | 2A | 562,416,065 | NC21 | 7.09E-04 | 2.32 |
| SL.NC | *AX-109286714* | 2A | 562,727,585 | NC21 | 3.16E-04 | 2.63 |
| SL.NC | *AX-110548395* | 2A | 563,167,478 | NC21 | 7.44E-05 | 3.19 |
| SL.NC | *AX-110548395* | 2A | 563,167,478 | NAVE | 7.49E-04 | 2.26 |
| SL.NC | *AX-86184745* | 2A | 564,502,809 | NC21 | 2.76E-04 | 2.68 |
| SL.NC | *AX-111505518* | 2A | 565,156,466 | NC21 | 1.87E-04 | 2.84 |
| SL.NC | *AX-109492818* | 2A | 566,443,098 | NC21 | 2.51E-04 | 2.72 |
| SL.NC | *AX-108771187* | 2A | 566,602,667 | NC21 | 2.24E-04 | 2.77 |
| SL.NC | *AX-109463348* | 2A | 567,768,912 | NC21 | 4.00E-04 | 2.54 |
| SL.NC | *AX-109280395* | 2A | 570,503,553 | NC21 | 2.30E-04 | 2.76 |
| SL.NC | *AX-109624083* | 2A | 571,429,016 | NC21 | 3.74E-04 | 2.57 |
| SL.NC | *AX-110928253* | 2A | 572,097,993 | NC21 | 2.47E-04 | 2.73 |
| SL.NC | *AX-94751398* | 2A | 573,368,522 | NC21 | 1.00E-04 | 3.08 |
| SL.NC | *AX-94553665* | 2A | 579,615,957 | NC21 | 8.89E-05 | 3.12 |
| SL.NC | *AX-109281522* | 2A | 580,324,391 | NC21 | 1.12E-04 | 3.04 |
| SL.NC | *AX-110510308* | 2A | 611,567,265 | NC20 | 5.43E-04 | 2.38 |
| SL.NC | *AX-86179766* | 2A | 611,946,860 | NC20 | 3.98E-04 | 2.49 |
| SL.NC | *AX-109973772* | 2B | 373,493,468 | NC20 | 6.88E-04 | 2.29 |
| SL.NC | *AX-108906314* | 2B | 550,396,678 | NAVE | 8.32E-04 | 2.22 |
| SL.NC | *AX-110647062* | 2D | 23,025,488 | NC19 | 7.67E-06 | 4.16 |
| SL.NC | *AX-110647062* | 2D | 23,025,488 | NAVE | 3.76E-05 | 3.40 |
| SL.NC | *AX-89690184* | 2D | 419,865,085 | NC19 | 6.57E-04 | 2.39 |
| SL.NC | *AX-89690184* | 2D | 419,865,085 | NAVE | 8.41E-04 | 2.22 |
| SL.NC | *AX-111708993* | 3A | 272,796,105 | NAVE | 6.61E-04 | 2.31 |
| SL.NC | *AX-110667599* | 3A | 580,598,934 | NC19 | 2.22E-04 | 2.81 |
| SL.NC | *AX-89436449* | 3A | 586,881,536 | NC20 | 9.49E-04 | 2.17 |
| SL.NC | *AX-111528962* | 3A | 633,291,342 | NC19 | 9.37E-04 | 2.25 |
| SL.NC | *AX-109984137* | 3A | 633,772,383 | NC19 | 8.17E-04 | 2.30 |
| SL.NC | *AX-108897269* | 3A | 634,086,482 | NC19 | 5.31E-04 | 2.47 |
| SL.NC | *AX-89381998* | 3A | 637,901,078 | NC19 | 1.02E-04 | 3.12 |
| SL.NC | *AX-110629944* | 3B | 30,535,199 | NC19 | 6.68E-04 | 2.38 |
| SL.NC | *AX-111089329* | 3B | 30,961,339 | NC19 | 9.66E-04 | 2.24 |
| SL.NC | *AX-109973543* | 3B | 493,634,836 | NAVE | 1.85E-04 | 2.79 |
| SL.NC | *AX-111087147* | 3B | 765,322,402 | NC19 | 2.35E-04 | 2.79 |
| SL.NC | *AX-111087147* | 3B | 765,322,402 | NAVE | 1.43E-04 | 2.89 |
| SL.NC | *AX-110122585* | 3B | 787,966,608 | NC20 | 4.35E-04 | 2.46 |
| SL.NC | *AX-110336377* | 3D | 132,645,888 | NAVE | 1.64E-04 | 2.84 |
| SL.NC | *AX-109284474* | 3D | 138,075,912 | NAVE | 3.09E-04 | 2.59 |
| SL.NC | *AX-108859112* | 3D | 139,250,658 | NAVE | 2.18E-04 | 2.73 |
| SL.NC | *AX-109662980* | 3D | 139,872,304 | NAVE | 3.84E-04 | 2.51 |
| SL.NC | *AX-110612358* | 3D | 140,733,879 | NC21 | 9.47E-04 | 2.21 |
| SL.NC | *AX-110612358* | 3D | 140,733,879 | NAVE | 5.33E-05 | 3.27 |
| SL.NC | *AX-110857044* | 3D | 141,027,488 | NAVE | 4.52E-04 | 2.45 |
| SL.NC | *AX-110826955* | 3D | 173,719,518 | NAVE | 9.43E-04 | 2.18 |
| SL.NC | *AX-109743167* | 3D | 178,403,033 | NAVE | 6.78E-04 | 2.30 |
| SL.NC | *AX-111054517* | 3D | 180,840,097 | NAVE | 9.31E-04 | 2.18 |
| SL.NC | *AX-111099273* | 3D | 182,645,267 | NAVE | 9.89E-04 | 2.16 |
| SL.NC | *AX-108895977* | 3D | 184,119,274 | NAVE | 8.52E-04 | 2.21 |
| SL.NC | *AX-111675563* | 3D | 184,510,481 | NAVE | 7.68E-04 | 2.25 |
| SL.NC | *AX-111131164* | 3D | 195,149,719 | NAVE | 5.48E-04 | 2.38 |
| SL.NC | *AX-111506806* | 3D | 198,833,762 | NAVE | 8.87E-04 | 2.20 |
| SL.NC | *AX-110434962* | 3D | 199,370,252 | NAVE | 8.43E-04 | 2.22 |
| SL.NC | *AX-109976094* | 3D | 204,178,154 | NAVE | 8.60E-04 | 2.21 |
| SL.NC | *AX-111408899* | 3D | 206,984,620 | NAVE | 7.83E-05 | 3.12 |
| SL.NC | *AX-108886572* | 3D | 212,145,323 | NAVE | 6.35E-04 | 2.32 |
| SL.NC | *AX-111504450* | 3D | 212,626,573 | NAVE | 9.86E-04 | 2.16 |
| SL.NC | *AX-110269943* | 3D | 222,364,369 | NAVE | 8.56E-04 | 2.21 |
| SL.NC | *AX-110520367* | 3D | 223,988,713 | NAVE | 9.34E-04 | 2.18 |
| SL.NC | *AX-110942803* | 3D | 228,487,539 | NAVE | 8.56E-04 | 2.21 |
| SL.NC | *AX-108756717* | 3D | 231,987,021 | NAVE | 6.77E-04 | 2.30 |
| SL.NC | *AX-109252875* | 3D | 235,244,297 | NAVE | 9.97E-04 | 2.16 |
| SL.NC | *AX-109429611* | 3D | 240,786,059 | NAVE | 8.98E-04 | 2.19 |
| SL.NC | *AX-111564705* | 3D | 241,726,550 | NAVE | 9.90E-04 | 2.16 |
| SL.NC | *AX-110944192* | 3D | 244,908,012 | NAVE | 9.60E-04 | 2.17 |
| SL.NC | *AX-109394931* | 3D | 246,015,647 | NAVE | 8.56E-04 | 2.21 |
| SL.NC | *AX-110015572* | 3D | 249,305,505 | NAVE | 8.70E-04 | 2.21 |
| SL.NC | *AX-110813891* | 3D | 249,519,280 | NAVE | 7.59E-04 | 2.26 |
| SL.NC | *AX-110890657* | 3D | 257,547,008 | NAVE | 9.16E-04 | 2.19 |
| SL.NC | *AX-111654550* | 3D | 257,929,333 | NAVE | 8.56E-04 | 2.21 |
| SL.NC | *AX-109471652* | 3D | 268,152,476 | NAVE | 9.29E-04 | 2.18 |
| SL.NC | *AX-111006048* | 3D | 270,143,417 | NAVE | 4.68E-04 | 2.44 |
| SL.NC | *AX-109399234* | 3D | 272,303,735 | NAVE | 9.53E-04 | 2.17 |
| SL.NC | *AX-110913237* | 3D | 273,001,033 | NAVE | 6.98E-04 | 2.29 |
| SL.NC | *AX-111147649* | 3D | 274,822,017 | NAVE | 8.56E-04 | 2.21 |
| SL.NC | *AX-111689571* | 3D | 274,984,684 | NAVE | 8.89E-04 | 2.20 |
| SL.NC | *AX-109505110* | 3D | 278,876,619 | NAVE | 9.52E-04 | 2.17 |
| SL.NC | *AX-109783478* | 3D | 289,604,746 | NAVE | 8.56E-04 | 2.21 |
| SL.NC | *AX-109735361* | 3D | 291,979,439 | NAVE | 8.98E-04 | 2.19 |
| SL.NC | *AX-111502769* | 3D | 295,145,732 | NAVE | 9.86E-04 | 2.16 |
| SL.NC | *AX-109954183* | 3D | 297,247,421 | NAVE | 6.55E-04 | 2.31 |
| SL.NC | *AX-110958097* | 3D | 310,718,976 | NAVE | 8.86E-04 | 2.20 |
| SL.NC | *AX-110288100* | 3D | 311,850,375 | NAVE | 8.86E-04 | 2.20 |
| SL.NC | *AX-110029250* | 3D | 312,753,243 | NAVE | 8.96E-04 | 2.20 |
| SL.NC | *AX-109355391* | 3D | 313,055,523 | NAVE | 8.47E-04 | 2.22 |
| SL.NC | *AX-108958991* | 3D | 313,586,377 | NAVE | 8.72E-04 | 2.21 |
| SL.NC | *AX-109744083* | 3D | 317,228,145 | NAVE | 7.60E-04 | 2.26 |
| SL.NC | *AX-111027124* | 3D | 400,781,396 | NC20 | 9.46E-04 | 2.17 |
| SL.NC | *AX-89699893* | 3D | 515,930,886 | NC21 | 8.33E-05 | 3.15 |
| SL.NC | *AX-89699893* | 3D | 515,930,886 | NAVE | 1.94E-04 | 2.77 |
| SL.NC | *AX-110772653* | 3D | 570,154,362 | NC20 | 5.03E-05 | 3.28 |
| SL.NC | *AX-108959104* | 4A | 617,121,150 | NC19 | 6.19E-04 | 2.41 |
| SL.NC | *AX-108959104* | 4A | 617,121,150 | NAVE | 4.74E-05 | 3.31 |
| SL.NC | *AX-111165119* | 4A | 640,174,538 | NC20 | 6.07E-04 | 2.34 |
| SL.NC | *AX-111165119* | 4A | 640,174,538 | NAVE | 2.70E-04 | 2.65 |
| SL.NC | *AX-110971025* | 4A | 641,592,792 | NC19 | 8.51E-04 | 2.29 |
| SL.NC | *AX-110971025* | 4A | 641,592,792 | NAVE | 8.47E-04 | 2.22 |
| SL.NC | *AX-108949287* | 4A | 686,173,880 | NC21 | 2.72E-04 | 2.69 |
| SL.NC | *AX-108949287* | 4A | 686,173,880 | NAVE | 3.82E-04 | 2.51 |
| SL.NC | *AX-110920884* | 4A | 686,381,407 | NC21 | 1.06E-04 | 3.06 |
| SL.NC | *AX-110920884* | 4A | 686,381,407 | NAVE | 1.82E-04 | 2.80 |
| SL.NC | *AX-111691908* | 4B | 8,560,072 | NC20 | 2.10E-04 | 2.74 |
| SL.NC | *AX-111691908* | 4B | 8,560,072 | NAVE | 6.39E-04 | 2.32 |
| SL.NC | *AX-109888584* | 4B | 533,747,853 | NAVE | 9.33E-04 | 2.18 |
| SL.NC | *AX-89577308* | 4B | 559,647,884 | NC19 | 7.57E-04 | 2.33 |
| SL.NC | *AX-111503872* | 4B | 636,757,506 | NC19 | 3.50E-04 | 2.63 |
| SL.NC | *AX-109283664* | 4B | 665,404,036 | NAVE | 8.44E-04 | 2.22 |
| SL.NC | *AX-109732646* | 4D | 96,147,593 | NC19 | 4.01E-04 | 2.58 |
| SL.NC | *AX-109732646* | 4D | 96,147,593 | NAVE | 8.06E-04 | 2.24 |
| SL.NC | *AX-108726608* | 4D | 98,066,048 | NC19 | 7.95E-04 | 2.31 |
| SL.NC | *AX-108726608* | 4D | 98,066,048 | NAVE | 6.91E-04 | 2.29 |
| SL.NC | *AX-110951951* | 4D | 133,615,550 | NC19 | 9.87E-04 | 2.23 |
| SL.NC | *AX-108968169* | 4D | 136,468,228 | NC19 | 6.36E-04 | 2.40 |
| SL.NC | *AX-108959486* | 4D | 138,804,313 | NC19 | 4.41E-04 | 2.54 |
| SL.NC | *AX-109941006* | 4D | 141,083,152 | NC19 | 9.02E-04 | 2.27 |
| SL.NC | *AX-111412197* | 4D | 142,538,143 | NC19 | 9.70E-04 | 2.24 |
| SL.NC | *AX-111142906* | 4D | 172,954,584 | NC19 | 4.95E-04 | 2.50 |
| SL.NC | *AX-110068845* | 4D | 175,585,236 | NC19 | 5.46E-04 | 2.46 |
| SL.NC | *AX-109142060* | 4D | 176,907,162 | NC19 | 8.37E-04 | 2.30 |
| SL.NC | *AX-110228302* | 4D | 181,975,287 | NC19 | 6.32E-04 | 2.40 |
| SL.NC | *AX-110568577* | 4D | 200,403,100 | NC19 | 4.26E-04 | 2.56 |
| SL.NC | *AX-109067446* | 4D | 203,168,902 | NC19 | 8.20E-04 | 2.30 |
| SL.NC | *AX-110217000* | 4D | 211,736,901 | NC19 | 3.25E-04 | 2.66 |
| SL.NC | *AX-109970514* | 4D | 226,613,847 | NC19 | 9.70E-04 | 2.24 |
| SL.NC | *AX-111367897* | 4D | 233,161,725 | NC19 | 7.65E-04 | 2.33 |
| SL.NC | *AX-109072188* | 4D | 254,073,000 | NC19 | 8.48E-04 | 2.29 |
| SL.NC | *AX-109408679* | 4D | 265,722,005 | NC19 | 9.02E-04 | 2.27 |
| SL.NC | *AX-111978711* | 4D | 283,613,809 | NC19 | 7.34E-04 | 2.35 |
| SL.NC | *AX-110040955* | 4D | 290,335,021 | NC19 | 8.85E-04 | 2.27 |
| SL.NC | *AX-111580635* | 4D | 294,021,419 | NC19 | 7.33E-04 | 2.35 |
| SL.NC | *AX-108949163* | 4D | 295,112,214 | NC19 | 3.31E-04 | 2.65 |
| SL.NC | *AX-94709746* | 4D | 369,120,548 | NAVE | 9.41E-05 | 3.05 |
| SL.NC | *AX-109439402* | 5A | 414,892,950 | NC20 | 2.47E-04 | 2.67 |
| SL.NC | *AX-110508884* | 5A | 428,416,594 | NC20 | 6.59E-04 | 2.31 |
| SL.NC | *AX-108781805* | 5A | 430,238,341 | NC20 | 2.85E-04 | 2.62 |
| SL.NC | *AX-110536448* | 5A | 536,677,135 | NC20 | 5.74E-04 | 2.36 |
| SL.NC | *AX-111530336* | 5A | 547,622,123 | NC20 | 8.32E-04 | 2.22 |
| SL.NC | *AX-109874877* | 5A | 684,629,598 | NAVE | 7.29E-04 | 2.27 |
| SL.NC | *AX-110420308* | 5B | 268,238,887 | NC19 | 7.69E-04 | 2.33 |
| SL.NC | *AX-94598757* | 5B | 316,562,253 | NC20 | 8.31E-04 | 2.22 |
| SL.NC | *AX-111759689* | 5B | 340,640,003 | NC21 | 5.76E-04 | 2.40 |
| SL.NC | *AX-111759689* | 5B | 340,640,003 | NAVE | 3.99E-04 | 2.50 |
| SL.NC | *AX-110006774* | 5B | 407,416,312 | NC20 | 3.99E-04 | 2.49 |
| SL.NC | *AX-108863692* | 5B | 410,266,474 | NC20 | 4.94E-04 | 2.41 |
| SL.NC | *AX-111013696* | 5B | 410,725,022 | NC20 | 6.51E-04 | 2.31 |
| SL.NC | *AX-109496576* | 5B | 411,742,999 | NC20 | 4.99E-04 | 2.41 |
| SL.NC | *AX-109284684* | 5B | 418,309,355 | NC20 | 1.57E-04 | 2.85 |
| SL.NC | *AX-108958865* | 5B | 418,509,439 | NC20 | 8.36E-04 | 2.22 |
| SL.NC | *AX-110576628* | 5B | 420,011,554 | NC20 | 9.78E-04 | 2.16 |
| SL.NC | *AX-108827033* | 5B | 422,218,746 | NC20 | 4.26E-04 | 2.47 |
| SL.NC | *AX-111672485* | 5B | 422,501,097 | NC20 | 3.65E-04 | 2.53 |
| SL.NC | *AX-111519225* | 5B | 422,925,740 | NC20 | 8.31E-04 | 2.22 |
| SL.NC | *AX-110950304* | 5B | 425,558,106 | NC20 | 5.48E-04 | 2.37 |
| SL.NC | *AX-111598230* | 5B | 508,062,768 | NC20 | 9.67E-04 | 2.16 |
| SL.NC | *AX-111543906* | 5D | 413,283,434 | NC20 | 9.90E-04 | 2.15 |
| SL.NC | *AX-110161029* | 5D | 417,855,064 | NC20 | 9.17E-04 | 2.18 |
| SL.NC | *AX-111355048* | 5D | 505,374,811 | NC21 | 7.24E-04 | 2.32 |
| SL.NC | *AX-109269999* | 5D | 515,042,383 | NC21 | 1.84E-04 | 2.84 |
| SL.NC | *AX-111226349* | 6A | 367,494,684 | NC21 | 6.78E-04 | 2.34 |
| SL.NC | *AX-110437185* | 6A | 401,607,947 | NC21 | 1.84E-04 | 2.84 |
| SL.NC | *AX-109826393* | 6A | 402,531,319 | NC21 | 2.40E-04 | 2.74 |
| SL.NC | *AX-108820938* | 6A | 404,451,680 | NC21 | 2.45E-04 | 2.73 |
| SL.NC | *AX-109592433* | 6A | 409,675,308 | NC21 | 2.40E-04 | 2.74 |
| SL.NC | *AX-111727298* | 6A | 411,086,966 | NC21 | 6.24E-04 | 2.37 |
| SL.NC | *AX-108914894* | 6A | 412,848,027 | NC21 | 2.40E-04 | 2.74 |
| SL.NC | *AX-111482414* | 6A | 416,377,937 | NC21 | 2.40E-04 | 2.74 |
| SL.NC | *AX-110729210* | 6A | 417,449,780 | NC21 | 2.40E-04 | 2.74 |
| SL.NC | *AX-110426364* | 6A | 418,537,094 | NC21 | 2.40E-04 | 2.74 |
| SL.NC | *AX-86167031* | 6A | 420,092,601 | NC21 | 2.40E-04 | 2.74 |
| SL.NC | *AX-110398932* | 6A | 420,820,706 | NC21 | 3.31E-04 | 2.61 |
| SL.NC | *AX-111016902* | 6A | 421,369,842 | NC21 | 1.68E-04 | 2.88 |
| SL.NC | *AX-111080243* | 6A | 423,523,213 | NC21 | 4.47E-04 | 2.50 |
| SL.NC | *AX-110988853* | 6A | 427,606,644 | NC21 | 2.40E-04 | 2.74 |
| SL.NC | *AX-109366794* | 6A | 435,252,894 | NC21 | 2.40E-04 | 2.74 |
| SL.NC | *AX-111594972* | 6A | 436,975,681 | NC21 | 3.63E-04 | 2.58 |
| SL.NC | *AX-109325937* | 6B | 57,366,180 | NC19 | 8.83E-04 | 2.27 |
| SL.NC | *AX-110961066* | 6B | 521,991,659 | NC21 | 7.04E-04 | 2.33 |
| SL.NC | *AX-110961066* | 6B | 521,991,659 | NAVE | 6.91E-04 | 2.29 |
| SL.NC | *AX-110578609* | 6B | 522,345,443 | NC21 | 3.40E-04 | 2.61 |
| SL.NC | *AX-110578609* | 6B | 522,345,443 | NAVE | 2.62E-04 | 2.66 |
| SL.NC | *AX-110977373* | 6B | 527,032,383 | NC21 | 6.82E-04 | 2.34 |
| SL.NC | *AX-110977373* | 6B | 527,032,383 | NAVE | 5.17E-04 | 2.40 |
| SL.NC | *AX-108882252* | 6B | 527,689,906 | NC21 | 4.30E-04 | 2.51 |
| SL.NC | *AX-108882252* | 6B | 527,689,906 | NAVE | 4.24E-04 | 2.48 |
| SL.NC | *AX-110587319* | 6B | 528,138,648 | NC21 | 3.20E-04 | 2.63 |
| SL.NC | *AX-110587319* | 6B | 528,138,648 | NAVE | 3.77E-04 | 2.52 |
| SL.NC | *AX-109337721* | 6B | 528,705,208 | NC20 | 8.86E-04 | 2.19 |
| SL.NC | *AX-109337721* | 6B | 528,705,208 | NC21 | 1.66E-04 | 2.88 |
| SL.NC | *AX-109337721* | 6B | 528,705,208 | NAVE | 7.17E-05 | 3.15 |
| SL.NC | *AX-110464411* | 6B | 529,217,159 | NC21 | 4.09E-04 | 2.53 |
| SL.NC | *AX-110464411* | 6B | 529,217,159 | NAVE | 2.98E-04 | 2.61 |
| SL.NC | *AX-111453972* | 6B | 529,697,367 | NC21 | 3.74E-04 | 2.57 |
| SL.NC | *AX-111453972* | 6B | 529,697,367 | NAVE | 3.11E-04 | 2.59 |
| SL.NC | *AX-111029681* | 6B | 530,743,557 | NC21 | 4.91E-04 | 2.46 |
| SL.NC | *AX-111029681* | 6B | 530,743,557 | NAVE | 3.87E-04 | 2.51 |
| SL.NC | *AX-109833239* | 6B | 644,381,391 | NC20 | 9.55E-04 | 2.17 |
| SL.NC | *AX-110936223* | 6D | 3,724,650 | NC20 | 2.37E-04 | 2.69 |
| SL.NC | *AX-110936223* | 6D | 3,724,650 | NAVE | 4.17E-04 | 2.48 |
| SL.NC | *AX-109394965* | 7A | 640,675,003 | NC19 | 2.68E-04 | 2.74 |
| SL.NC | *AX-109394965* | 7A | 640,675,003 | NAVE | 8.29E-05 | 3.10 |
| SL.NC | *AX-110398543* | 7A | 642,864,251 | NC21 | 3.24E-04 | 2.62 |
| SL.NC | *AX-110398543* | 7A | 642,864,251 | NAVE | 4.99E-04 | 2.41 |
| SL.NC | *AX-109370830* | 7A | 677,885,666 | NC20 | 8.04E-04 | 2.23 |
| SL.NC | *AX-110405886* | 7B | 45,434,802 | NC21 | 6.32E-04 | 2.37 |
| SL.NC | *AX-110405886* | 7B | 45,434,802 | NAVE | 8.15E-04 | 2.23 |
| SL.NC | *AX-111073795* | 7B | 46,342,044 | NC21 | 3.67E-04 | 2.58 |
| SL.NC | *AX-109949434* | 7B | 139,794,130 | NC20 | 2.58E-04 | 2.66 |
| SL.NC | *AX-109312499* | 7B | 140,710,979 | NC20 | 1.73E-04 | 2.81 |
| SL.NC | *AX-111200151* | 7B | 141,805,837 | NC20 | 2.21E-04 | 2.72 |
| SL.NC | *AX-111608660* | 7B | 142,291,056 | NC20 | 2.30E-04 | 2.70 |
| SL.NC | *AX-109306298* | 7B | 142,881,442 | NC20 | 3.43E-04 | 2.55 |
| SL.NC | *AX-109913831* | 7B | 143,321,152 | NC20 | 2.21E-04 | 2.72 |
| SL.NC | *AX-108818797* | 7B | 143,856,849 | NC20 | 2.06E-04 | 2.74 |
| SL.NC | *AX-110530584* | 7B | 144,269,602 | NC20 | 3.33E-04 | 2.56 |
| SL.NC | *AX-111012930* | 7B | 144,703,486 | NC20 | 5.78E-04 | 2.35 |
| SL.NC | *AX-109466205* | 7B | 152,508,240 | NC20 | 6.42E-04 | 2.31 |
| SL.NC | *AX-108803846* | 7B | 154,752,871 | NC20 | 6.40E-04 | 2.32 |
| SL.NC | *AX-109318746* | 7B | 167,173,369 | NC20 | 7.76E-04 | 2.24 |
| SL.NC | *AX-109458715* | 7B | 184,302,780 | NC20 | 2.99E-04 | 2.60 |
| SL.NC | *AX-109302618* | 7B | 185,067,482 | NC20 | 6.12E-04 | 2.33 |
| SL.NC | *AX-111098900* | 7B | 187,445,806 | NC20 | 3.33E-04 | 2.56 |
| SL.NC | *AX-108965135* | 7B | 187,487,473 | NC20 | 7.86E-05 | 3.11 |
| SL.NC | *AX-111573648* | 7B | 188,396,445 | NC20 | 2.06E-04 | 2.74 |
| SL.NC | *AX-111730499* | 7B | 327,141,110 | NC20 | 3.50E-04 | 2.54 |
| SL.NC | *AX-111514274* | 7B | 327,852,765 | NC20 | 5.61E-04 | 2.37 |
| SL.NC | *AX-110503493* | 7D | 40,499,497 | NC19 | 9.30E-04 | 2.25 |
| SL.NC | *AX-111106049* | 7D | 135,928,566 | NC21 | 1.50E-04 | 2.92 |
| SL.NC | *AX-111106049* | 7D | 135,928,566 | NAVE | 5.41E-05 | 3.26 |
| SL.NC | *AX-108818991* | 7D | 161,193,787 | NC21 | 4.22E-04 | 2.52 |
| SL.NC | *AX-111460143* | 7D | 162,456,920 | NC21 | 1.27E-05 | 3.89 |
| SL.NC | *AX-111460143* | 7D | 162,456,920 | NAVE | 2.54E-04 | 2.67 |
| SL.NC | *AX-111077878* | 7D | 164,793,391 | NC21 | 8.23E-05 | 3.15 |
| SL.NC | *AX-111507520* | 7D | 164,846,275 | NC21 | 4.83E-04 | 2.47 |
| SL.NC | *AX-109949077* | 7D | 164,892,887 | NC21 | 7.80E-05 | 3.18 |
| TGW.DT | *AX-109937019* | 1A | 525,123,787 | DT20 | 5.11E-04 | 2.33 |
| TGW.DT | *AX-110122042* | 1A | 528,633,639 | DT20 | 4.68E-04 | 2.37 |
| TGW.DT | *AX-110419096* | 1B | 290,870,245 | DT20 | 9.52E-04 | 2.11 |
| TGW.DT | *AX-109383638* | 1B | 295,437,309 | DT20 | 9.84E-04 | 2.10 |
| TGW.DT | *AX-108995348* | 1B | 357,649,901 | DT20 | 6.77E-04 | 2.23 |
| TGW.DT | *AX-110531641* | 1B | 365,441,304 | DT20 | 7.12E-04 | 2.21 |
| TGW.DT | *AX-111463788* | 1B | 365,926,056 | DT20 | 8.68E-04 | 2.14 |
| TGW.DT | *AX-111482877* | 1B | 366,492,631 | DT20 | 5.81E-04 | 2.29 |
| TGW.DT | *AX-109437757* | 1B | 366,917,565 | DT20 | 7.75E-04 | 2.18 |
| TGW.DT | *AX-111118968* | 1B | 367,588,737 | DT20 | 6.62E-04 | 2.24 |
| TGW.DT | *AX-111733823* | 1B | 367,838,063 | DT20 | 3.89E-04 | 2.43 |
| TGW.DT | *AX-110597141* | 1B | 368,675,675 | DT20 | 6.05E-04 | 2.27 |
| TGW.DT | *AX-110474956* | 1B | 369,345,717 | DT20 | 4.41E-04 | 2.39 |
| TGW.DT | *AX-110623229* | 1B | 369,515,449 | DT20 | 6.23E-04 | 2.26 |
| TGW.DT | *AX-109581046* | 1B | 370,197,585 | DT20 | 6.07E-04 | 2.27 |
| TGW.DT | *AX-108845851* | 1B | 371,140,782 | DT20 | 4.02E-04 | 2.42 |
| TGW.DT | *AX-110386580* | 1B | 372,157,887 | DT20 | 6.69E-04 | 2.24 |
| TGW.DT | *AX-109387657* | 1B | 372,217,456 | DT20 | 6.17E-04 | 2.27 |
| TGW.DT | *AX-110689665* | 1B | 372,748,785 | DT20 | 8.58E-04 | 2.15 |
| TGW.DT | *AX-110740030* | 1B | 373,856,428 | DT20 | 7.53E-04 | 2.19 |
| TGW.DT | *AX-111707424* | 1B | 374,316,467 | DT20 | 5.37E-04 | 2.32 |
| TGW.DT | *AX-109932384* | 1B | 374,913,451 | DT20 | 8.59E-04 | 2.14 |
| TGW.DT | *AX-109580210* | 1B | 375,303,546 | DT20 | 5.32E-04 | 2.32 |
| TGW.DT | *AX-110369203* | 1B | 375,821,909 | DT20 | 4.21E-04 | 2.40 |
| TGW.DT | *AX-109544098* | 1B | 377,963,460 | DT20 | 3.48E-04 | 2.47 |
| TGW.DT | *AX-109853345* | 1B | 378,461,864 | DT20 | 8.10E-04 | 2.17 |
| TGW.DT | *AX-111496509* | 1B | 378,971,833 | DT20 | 4.84E-04 | 2.35 |
| TGW.DT | *AX-109070579* | 1B | 379,583,836 | DT20 | 5.26E-04 | 2.32 |
| TGW.DT | *AX-109271826* | 1B | 380,240,446 | DT20 | 5.35E-04 | 2.32 |
| TGW.DT | *AX-111503092* | 1B | 380,647,070 | DT20 | 4.35E-04 | 2.39 |
| TGW.DT | *AX-108973265* | 1B | 381,032,751 | DT20 | 6.95E-04 | 2.22 |
| TGW.DT | *AX-94515082* | 1B | 383,002,046 | DT20 | 6.46E-04 | 2.25 |
| TGW.DT | *AX-109983759* | 1B | 383,176,594 | DT20 | 5.37E-04 | 2.32 |
| TGW.DT | *AX-109419267* | 1B | 384,249,145 | DT20 | 5.17E-04 | 2.33 |
| TGW.DT | *AX-108756721* | 1B | 384,566,103 | DT20 | 7.87E-04 | 2.18 |
| TGW.DT | *AX-111715015* | 1B | 385,300,997 | DT20 | 8.08E-04 | 2.17 |
| TGW.DT | *AX-111468437* | 1B | 386,240,704 | DT20 | 5.83E-04 | 2.29 |
| TGW.DT | *AX-109340517* | 1B | 386,689,382 | DT20 | 5.69E-04 | 2.29 |
| TGW.DT | *AX-109391593* | 1B | 387,288,895 | DT20 | 5.18E-04 | 2.33 |
| TGW.DT | *AX-110062945* | 1B | 387,783,486 | DT20 | 8.48E-04 | 2.15 |
| TGW.DT | *AX-111549107* | 1B | 388,341,838 | DT20 | 5.66E-04 | 2.30 |
| TGW.DT | *AX-110121329* | 1B | 388,895,243 | DT20 | 8.01E-04 | 2.17 |
| TGW.DT | *AX-111022842* | 1B | 389,321,645 | DT20 | 5.34E-04 | 2.32 |
| TGW.DT | *AX-109873586* | 1B | 389,879,147 | DT20 | 5.40E-04 | 2.31 |
| TGW.DT | *AX-111109396* | 1B | 390,276,179 | DT20 | 6.51E-04 | 2.25 |
| TGW.DT | *AX-110041061* | 1B | 390,870,930 | DT20 | 5.34E-04 | 2.32 |
| TGW.DT | *AX-110002681* | 1B | 391,917,068 | DT20 | 8.62E-04 | 2.14 |
| TGW.DT | *AX-108747601* | 1B | 395,959,962 | DT20 | 4.42E-04 | 2.39 |
| TGW.DT | *AX-108959675* | 1B | 398,788,024 | DT20 | 9.55E-04 | 2.11 |
| TGW.DT | *AX-110437942* | 1B | 399,329,113 | DT20 | 6.22E-04 | 2.26 |
| TGW.DT | *AX-111608582* | 1B | 400,300,433 | DT20 | 4.24E-04 | 2.40 |
| TGW.DT | *AX-111013576* | 1B | 400,746,236 | DT20 | 3.47E-04 | 2.48 |
| TGW.DT | *AX-109971835* | 1B | 401,333,244 | DT20 | 8.35E-04 | 2.16 |
| TGW.DT | *AX-109447941* | 1B | 401,821,047 | DT20 | 9.29E-04 | 2.12 |
| TGW.DT | *AX-109272373* | 1B | 404,523,501 | DT20 | 7.97E-04 | 2.17 |
| TGW.DT | *AX-111782039* | 1B | 404,883,727 | DT20 | 4.94E-04 | 2.35 |
| TGW.DT | *AX-111744000* | 1B | 405,524,154 | DT20 | 3.79E-04 | 2.44 |
| TGW.DT | *AX-109356137* | 1B | 409,617,898 | DT20 | 9.14E-04 | 2.12 |
| TGW.DT | *AX-111083420* | 1B | 418,826,302 | DT20 | 6.49E-04 | 2.25 |
| TGW.DT | *AX-111169510* | 1B | 430,155,688 | DT21 | 8.38E-04 | 2.19 |
| TGW.DT | *AX-111169510* | 1B | 430,155,688 | DAVE | 8.20E-04 | 2.10 |
| TGW.DT | *AX-108872900* | 1B | 432,495,179 | DAVE | 5.50E-04 | 2.24 |
| TGW.DT | *AX-109301822* | 1B | 433,604,952 | DAVE | 3.26E-04 | 2.43 |
| TGW.DT | *AX-111636804* | 1B | 433,952,917 | DAVE | 4.74E-04 | 2.29 |
| TGW.DT | *AX-110125845* | 1B | 434,542,934 | DAVE | 3.26E-04 | 2.43 |
| TGW.DT | *AX-111105746* | 1B | 434,753,754 | DAVE | 3.47E-04 | 2.41 |
| TGW.DT | *AX-108788663* | 1B | 441,384,338 | DAVE | 6.54E-04 | 2.18 |
| TGW.DT | *AX-110365668* | 1B | 443,330,272 | DT21 | 5.12E-04 | 2.38 |
| TGW.DT | *AX-108888595* | 1B | 443,566,852 | DAVE | 8.49E-04 | 2.09 |
| TGW.DT | *AX-110916356* | 1B | 489,129,788 | DT20 | 6.34E-04 | 2.25 |
| TGW.DT | *AX-110670988* | 1B | 489,973,360 | DT20 | 5.48E-04 | 2.31 |
| TGW.DT | *AX-110598536* | 1B | 509,300,586 | DT20 | 6.34E-04 | 2.25 |
| TGW.DT | *AX-94442756* | 1B | 509,901,004 | DT20 | 4.09E-04 | 2.41 |
| TGW.DT | *AX-111780371* | 1B | 510,356,945 | DT20 | 9.35E-05 | 2.96 |
| TGW.DT | *AX-111780371* | 1B | 510,356,945 | DAVE | 5.30E-04 | 2.26 |
| TGW.DT | *AX-108920917* | 1B | 511,050,019 | DT20 | 7.52E-05 | 3.04 |
| TGW.DT | *AX-108920917* | 1B | 511,050,019 | DAVE | 4.53E-04 | 2.31 |
| TGW.DT | *AX-109457059* | 1B | 511,409,502 | DT20 | 2.31E-05 | 3.48 |
| TGW.DT | *AX-109457059* | 1B | 511,409,502 | DAVE | 2.08E-04 | 2.59 |
| TGW.DT | *AX-109966524* | 1B | 512,107,159 | DT20 | 3.71E-05 | 3.30 |
| TGW.DT | *AX-109966524* | 1B | 512,107,159 | DAVE | 2.24E-04 | 2.56 |
| TGW.DT | *AX-94532894* | 1B | 512,458,040 | DT20 | 1.13E-04 | 2.89 |
| TGW.DT | *AX-94532894* | 1B | 512,458,040 | DAVE | 7.89E-04 | 2.11 |
| TGW.DT | *AX-108750098* | 1B | 512,924,054 | DT20 | 5.44E-05 | 3.16 |
| TGW.DT | *AX-109881451* | 1B | 643,934,430 | DT19 | 8.19E-04 | 2.71 |
| TGW.DT | *AX-112289703* | 1B | 673,176,421 | DT20 | 4.07E-04 | 2.42 |
| TGW.DT | *AX-110929436* | 1D | 201,924,443 | DT20 | 7.48E-04 | 2.19 |
| TGW.DT | *AX-95660056* | 2A | 603,524,503 | DAVE | 6.89E-04 | 2.16 |
| TGW.DT | *AX-110425132* | 2A | 705,807,076 | DT21 | 5.91E-04 | 2.32 |
| TGW.DT | *AX-109576757* | 2A | 751,912,827 | DT20 | 5.05E-04 | 2.34 |
| TGW.DT | *AX-94910470* | 2A | 763,695,272 | DT20 | 5.05E-04 | 2.34 |
| TGW.DT | *AX-94910470* | 2A | 763,695,272 | DAVE | 7.14E-04 | 2.15 |
| TGW.DT | *AX-109352432* | 2B | 731,463,570 | DT21 | 3.47E-04 | 2.52 |
| TGW.DT | *AX-111009907* | 2B | 732,889,613 | DAVE | 3.54E-04 | 2.40 |
| TGW.DT | *AX-108860141* | 3A | 713,551,836 | DT20 | 1.32E-04 | 2.83 |
| TGW.DT | *AX-108887339* | 3A | 714,020,755 | DT20 | 2.77E-05 | 3.42 |
| TGW.DT | *AX-109910758* | 3B | 59,054,885 | DAVE | 8.28E-04 | 2.10 |
| TGW.DT | *AX-109913835* | 3B | 84,414,549 | DT20 | 5.26E-04 | 2.32 |
| TGW.DT | *AX-109316602* | 3B | 84,882,515 | DT20 | 8.47E-04 | 2.15 |
| TGW.DT | *AX-110505615* | 3B | 95,857,577 | DT20 | 5.01E-04 | 2.34 |
| TGW.DT | *AX-110934652* | 3B | 96,833,319 | DT20 | 4.79E-04 | 2.36 |
| TGW.DT | *AX-111049794* | 3B | 116,062,830 | DT20 | 4.54E-04 | 2.38 |
| TGW.DT | *AX-108790822* | 3B | 141,628,197 | DAVE | 6.26E-04 | 2.20 |
| TGW.DT | *AX-110418888* | 3B | 785,432,286 | DAVE | 1.81E-04 | 2.64 |
| TGW.DT | *AX-110270468* | 3D | 75,351,635 | DT19 | 8.35E-05 | 3.77 |
| TGW.DT | *AX-110464489* | 3D | 82,139,636 | DT19 | 2.37E-04 | 3.28 |
| TGW.DT | *AX-109842327* | 3D | 82,908,502 | DT19 | 2.57E-04 | 3.25 |
| TGW.DT | *AX-110372611* | 3D | 105,100,029 | DT19 | 7.50E-04 | 2.75 |
| TGW.DT | *AX-111103248* | 3D | 178,761,794 | DT21 | 4.19E-04 | 2.45 |
| TGW.DT | *AX-111103248* | 3D | 178,761,794 | DAVE | 3.07E-04 | 2.45 |
| TGW.DT | *AX-109903567* | 3D | 196,832,500 | DT21 | 6.98E-04 | 2.26 |
| TGW.DT | *AX-109903567* | 3D | 196,832,500 | DAVE | 3.20E-04 | 2.43 |
| TGW.DT | *AX-111778716* | 3D | 202,665,077 | DAVE | 5.79E-04 | 2.22 |
| TGW.DT | *AX-110432825* | 3D | 206,655,789 | DT21 | 5.23E-04 | 2.37 |
| TGW.DT | *AX-110432825* | 3D | 206,655,789 | DAVE | 3.10E-04 | 2.45 |
| TGW.DT | *AX-109341556* | 3D | 221,046,278 | DT21 | 4.19E-04 | 2.45 |
| TGW.DT | *AX-109341556* | 3D | 221,046,278 | DAVE | 3.07E-04 | 2.45 |
| TGW.DT | *AX-109626991* | 3D | 224,648,576 | DT21 | 7.46E-04 | 2.24 |
| TGW.DT | *AX-109626991* | 3D | 224,648,576 | DAVE | 4.23E-04 | 2.34 |
| TGW.DT | *AX-109392999* | 3D | 236,169,118 | DT21 | 6.39E-04 | 2.29 |
| TGW.DT | *AX-109392999* | 3D | 236,169,118 | DAVE | 3.67E-04 | 2.39 |
| TGW.DT | *AX-109407590* | 3D | 246,923,598 | DT21 | 6.16E-04 | 2.31 |
| TGW.DT | *AX-109407590* | 3D | 246,923,598 | DAVE | 3.78E-04 | 2.38 |
| TGW.DT | *AX-110830021* | 3D | 260,816,674 | DT21 | 5.50E-04 | 2.35 |
| TGW.DT | *AX-110830021* | 3D | 260,816,674 | DAVE | 3.65E-04 | 2.39 |
| TGW.DT | *AX-109998069* | 3D | 438,515,754 | DT19 | 1.40E-04 | 3.53 |
| TGW.DT | *AX-110933776* | 3D | 441,646,439 | DT19 | 5.90E-04 | 2.86 |
| TGW.DT | *AX-109164596* | 3D | 449,334,823 | DT19 | 2.62E-04 | 3.24 |
| TGW.DT | *AX-110016016* | 4A | 488,553,399 | DT21 | 8.66E-04 | 2.18 |
| TGW.DT | *AX-109509506* | 4A | 488,867,412 | DT21 | 9.59E-04 | 2.14 |
| TGW.DT | *AX-110468887* | 4A | 491,976,508 | DT21 | 1.95E-04 | 2.74 |
| TGW.DT | *AX-111743032* | 4A | 492,525,624 | DT21 | 2.07E-04 | 2.71 |
| TGW.DT | *AX-111528491* | 4A | 496,110,069 | DT21 | 1.74E-04 | 2.78 |
| TGW.DT | *AX-111451343* | 4A | 499,611,798 | DT21 | 3.18E-04 | 2.55 |
| TGW.DT | *AX-108870577* | 4A | 500,119,543 | DT21 | 1.44E-04 | 2.85 |
| TGW.DT | *AX-110931508* | 4A | 632,242,065 | DAVE | 9.76E-04 | 2.04 |
| TGW.DT | *AX-111040045* | 4A | 632,764,370 | DT20 | 7.92E-04 | 2.17 |
| TGW.DT | *AX-111040045* | 4A | 632,764,370 | DAVE | 5.45E-04 | 2.25 |
| TGW.DT | *AX-109375057* | 4A | 633,308,367 | DT20 | 1.34E-04 | 2.83 |
| TGW.DT | *AX-109375057* | 4A | 633,308,367 | DT21 | 8.58E-04 | 2.18 |
| TGW.DT | *AX-109375057* | 4A | 633,308,367 | DAVE | 3.23E-05 | 3.26 |
| TGW.DT | *AX-110468999* | 4A | 634,108,610 | DT20 | 4.70E-04 | 2.36 |
| TGW.DT | *AX-110468999* | 4A | 634,108,610 | DAVE | 1.88E-04 | 2.62 |
| TGW.DT | *AX-111140650* | 4A | 634,368,256 | DT20 | 5.68E-04 | 2.29 |
| TGW.DT | *AX-111140650* | 4A | 634,368,256 | DAVE | 2.96E-04 | 2.46 |
| TGW.DT | *AX-111453918* | 4A | 634,833,572 | DT20 | 5.81E-04 | 2.29 |
| TGW.DT | *AX-111453918* | 4A | 634,833,572 | DAVE | 1.71E-04 | 2.66 |
| TGW.DT | *AX-110440161* | 4A | 635,292,292 | DT20 | 8.79E-04 | 2.14 |
| TGW.DT | *AX-110440161* | 4A | 635,292,292 | DAVE | 2.47E-04 | 2.53 |
| TGW.DT | *AX-108950546* | 4A | 635,764,214 | DT20 | 9.51E-05 | 2.95 |
| TGW.DT | *AX-108950546* | 4A | 635,764,214 | DAVE | 2.94E-05 | 3.30 |
| TGW.DT | *AX-108908317* | 4A | 681,180,902 | DAVE | 5.18E-04 | 2.26 |
| TGW.DT | *AX-109301937* | 4B | 19,757,454 | DT20 | 4.14E-04 | 2.41 |
| TGW.DT | *AX-108933901* | 4B | 20,228,930 | DT20 | 8.86E-04 | 2.13 |
| TGW.DT | *AX-111139813* | 4B | 20,230,356 | DT20 | 7.51E-04 | 2.19 |
| TGW.DT | *AX-111695911* | 4B | 20,282,304 | DT20 | 8.37E-04 | 2.15 |
| TGW.DT | *AX-111516589* | 4B | 20,304,554 | DT20 | 9.37E-04 | 2.11 |
| TGW.DT | *AX-95160379* | 4B | 30,589,960 | DT21 | 2.74E-10 | 8.07 |
| TGW.DT | *AX-95160379* | 4B | 30,589,960 | DAVE | 1.14E-05 | 3.64 |
| TGW.DT | *AX-110021330* | 4B | 30,863,602 | DT21 | 4.76E-10 | 7.84 |
| TGW.DT | *AX-110021330* | 4B | 30,863,602 | DAVE | 2.01E-05 | 3.44 |
| TGW.DT | *AX-110123920* | 4B | 30,865,957 | DT21 | 7.69E-05 | 3.09 |
| TGW.DT | *AX-94607619* | 4B | 32,252,401 | DT21 | 8.70E-05 | 3.04 |
| TGW.DT | *AX-109868698* | 4B | 38,456,815 | DT20 | 2.02E-04 | 2.67 |
| TGW.DT | *AX-109868698* | 4B | 38,456,815 | DAVE | 3.01E-04 | 2.46 |
| TGW.DT | *AX-95630384* | 4B | 99,064,194 | DT21 | 6.26E-04 | 2.30 |
| TGW.DT | *AX-111150060* | 4B | 660,589,159 | DT19 | 9.20E-04 | 2.66 |
| TGW.DT | *AX-110086840* | 4B | 660,666,115 | DT19 | 7.21E-04 | 2.77 |
| TGW.DT | *AX-109311273* | 4D | 239,346,092 | DT19 | 9.81E-04 | 2.63 |
| TGW.DT | *AX-108920376* | 4D | 267,200,588 | DT19 | 9.81E-04 | 2.63 |
| TGW.DT | *AX-111544209* | 4D | 338,444,992 | DT19 | 1.02E-04 | 3.68 |
| TGW.DT | *AX-111486136* | 4D | 369,262,204 | DT19 | 2.07E-04 | 3.35 |
| TGW.DT | *AX-108830474* | 4D | 413,032,782 | DT19 | 8.00E-04 | 2.72 |
| TGW.DT | *AX-109775486* | 4D | 413,584,571 | DT20 | 4.85E-04 | 2.35 |
| TGW.DT | *AX-109936458* | 5A | 46,425,005 | DT21 | 6.09E-04 | 2.31 |
| TGW.DT | *AX-109369427* | 5A | 546,521,932 | DT19 | 9.24E-05 | 3.72 |
| TGW.DT | *AX-109369427* | 5A | 546,521,932 | DT20 | 3.75E-04 | 2.45 |
| TGW.DT | *AX-110016633* | 5A | 547,333,614 | DT19 | 8.11E-04 | 2.72 |
| TGW.DT | *AX-110016633* | 5A | 547,333,614 | DT20 | 5.17E-04 | 2.33 |
| TGW.DT | *AX-110016633* | 5A | 547,333,614 | DAVE | 5.48E-04 | 2.24 |
| TGW.DT | *AX-108977165* | 5A | 644,300,111 | DT20 | 9.32E-04 | 2.12 |
| TGW.DT | *AX-111564977* | 5B | 14,177,507 | DT21 | 6.96E-05 | 3.13 |
| TGW.DT | *AX-111564977* | 5B | 14,177,507 | DAVE | 2.06E-04 | 2.59 |
| TGW.DT | *AX-108899793* | 5B | 29,124,054 | DT20 | 9.81E-05 | 2.94 |
| TGW.DT | *AX-109538613* | 5B | 29,430,400 | DT20 | 3.29E-04 | 2.50 |
| TGW.DT | *AX-110923135* | 5B | 30,025,200 | DT20 | 3.65E-04 | 2.46 |
| TGW.DT | *AX-111661980* | 5B | 30,846,525 | DT20 | 2.49E-04 | 2.60 |
| TGW.DT | *AX-111032354* | 5B | 31,142,410 | DT20 | 5.40E-04 | 2.31 |
| TGW.DT | *AX-94598757* | 5B | 316,562,253 | DT20 | 3.08E-04 | 2.52 |
| TGW.DT | *AX-108735405* | 5B | 317,779,854 | DT20 | 3.48E-04 | 2.47 |
| TGW.DT | *AX-110586527* | 5B | 317,993,039 | DT20 | 4.63E-04 | 2.37 |
| TGW.DT | *AX-108868375* | 5B | 319,333,756 | DT20 | 5.15E-04 | 2.33 |
| TGW.DT | *AX-110509091* | 5B | 321,408,342 | DT20 | 5.06E-04 | 2.34 |
| TGW.DT | *AX-111655832* | 5B | 322,335,291 | DT20 | 4.52E-04 | 2.38 |
| TGW.DT | *AX-108726877* | 5B | 324,245,758 | DT20 | 7.13E-04 | 2.21 |
| TGW.DT | *AX-108937690* | 5B | 324,414,358 | DT20 | 4.52E-04 | 2.38 |
| TGW.DT | *AX-111638173* | 5B | 325,068,931 | DT20 | 6.02E-04 | 2.27 |
| TGW.DT | *AX-108949832* | 5B | 326,508,028 | DT20 | 2.82E-04 | 2.55 |
| TGW.DT | *AX-109815720* | 5B | 326,795,120 | DT20 | 3.30E-04 | 2.49 |
| TGW.DT | *AX-110386773* | 5B | 641,060,386 | DT21 | 7.93E-04 | 2.21 |
| TGW.DT | *AX-108757760* | 5B | 701,544,672 | DT19 | 7.24E-04 | 2.77 |
| TGW.DT | *AX-109536742* | 5D | 21,013,112 | DT21 | 2.18E-04 | 2.70 |
| TGW.DT | *AX-109536742* | 5D | 21,013,112 | DAVE | 7.01E-04 | 2.16 |
| TGW.DT | *AX-110758473* | 5D | 292,945,585 | DT19 | 6.28E-04 | 2.83 |
| TGW.DT | *AX-110758473* | 5D | 292,945,585 | DT21 | 4.27E-04 | 2.44 |
| TGW.DT | *AX-108782785* | 5D | 298,445,559 | DT19 | 9.38E-04 | 2.65 |
| TGW.DT | *AX-110570148* | 5D | 407,762,345 | DT20 | 9.24E-04 | 2.12 |
| TGW.DT | *AX-110570148* | 5D | 407,762,345 | DAVE | 5.28E-04 | 2.26 |
| TGW.DT | *AX-109897023* | 5D | 408,975,320 | DT20 | 8.31E-05 | 3.00 |
| TGW.DT | *AX-109897023* | 5D | 408,975,320 | DAVE | 8.24E-04 | 2.10 |
| TGW.DT | *AX-109947280* | 5D | 409,567,524 | DT20 | 9.99E-05 | 2.94 |
| TGW.DT | *AX-109947280* | 5D | 409,567,524 | DAVE | 7.81E-04 | 2.12 |
| TGW.DT | *AX-110405892* | 5D | 410,779,455 | DT20 | 1.84E-04 | 2.71 |
| TGW.DT | *AX-111055722* | 5D | 446,113,191 | DT21 | 1.45E-04 | 2.85 |
| TGW.DT | *AX-111557248* | 5D | 539,585,015 | DAVE | 9.15E-04 | 2.06 |
| TGW.DT | *AX-109968031* | 6A | 90,030,101 | DT21 | 2.37E-04 | 2.66 |
| TGW.DT | *AX-110619257* | 6A | 105,181,270 | DT21 | 9.79E-04 | 2.14 |
| TGW.DT | *AX-109385016* | 6A | 219,624,399 | DT20 | 2.20E-05 | 3.50 |
| TGW.DT | *AX-110482082* | 6A | 237,479,423 | DT19 | 9.41E-04 | 2.65 |
| TGW.DT | *AX-110482082* | 6A | 237,479,423 | DAVE | 6.27E-04 | 2.20 |
| TGW.DT | *AX-109093066* | 6A | 237,647,623 | DAVE | 5.38E-04 | 2.25 |
| TGW.DT | *AX-109061200* | 6A | 237,728,800 | DT19 | 9.01E-04 | 2.67 |
| TGW.DT | *AX-109061200* | 6A | 237,728,800 | DAVE | 4.05E-04 | 2.35 |
| TGW.DT | *AX-111243668* | 6A | 237,805,680 | DT19 | 7.72E-04 | 2.74 |
| TGW.DT | *AX-111243668* | 6A | 237,805,680 | DAVE | 5.48E-04 | 2.24 |
| TGW.DT | *AX-109077760* | 6A | 240,361,344 | DT19 | 8.98E-04 | 2.67 |
| TGW.DT | *AX-109077760* | 6A | 240,361,344 | DAVE | 7.02E-04 | 2.16 |
| TGW.DT | *AX-110593017* | 6A | 245,781,410 | DT19 | 8.28E-04 | 2.71 |
| TGW.DT | *AX-110593017* | 6A | 245,781,410 | DAVE | 8.81E-04 | 2.08 |
| TGW.DT | *AX-111146080* | 6A | 259,315,472 | DAVE | 9.47E-04 | 2.05 |
| TGW.DT | *AX-110596423* | 6A | 262,623,476 | DAVE | 8.70E-04 | 2.08 |
| TGW.DT | *AX-109282602* | 6A | 277,282,442 | DAVE | 7.12E-04 | 2.15 |
| TGW.DT | *AX-95630469* | 6A | 286,320,473 | DT19 | 8.92E-04 | 2.67 |
| TGW.DT | *AX-95630469* | 6A | 286,320,473 | DAVE | 7.53E-04 | 2.13 |
| TGW.DT | *AX-108975281* | 6A | 292,794,082 | DAVE | 7.73E-04 | 2.12 |
| TGW.DT | *AX-111266348* | 6A | 296,128,743 | DAVE | 7.87E-04 | 2.12 |
| TGW.DT | *AX-110151642* | 6A | 296,195,705 | DAVE | 7.09E-04 | 2.15 |
| TGW.DT | *AX-111635885* | 6A | 297,411,376 | DAVE | 6.75E-04 | 2.17 |
| TGW.DT | *AX-94952400* | 6A | 299,335,643 | DAVE | 6.69E-04 | 2.17 |
| TGW.DT | *AX-109918284* | 6A | 300,755,858 | DAVE | 6.13E-04 | 2.20 |
| TGW.DT | *AX-109903000* | 6A | 302,251,872 | DAVE | 6.41E-04 | 2.19 |
| TGW.DT | *AX-108975012* | 6A | 303,534,001 | DT19 | 8.12E-04 | 2.72 |
| TGW.DT | *AX-108975012* | 6A | 303,534,001 | DAVE | 2.07E-04 | 2.59 |
| TGW.DT | *AX-108991354* | 6A | 306,048,895 | DAVE | 5.67E-04 | 2.23 |
| TGW.DT | *AX-111059292* | 6A | 308,821,559 | DAVE | 6.05E-04 | 2.21 |
| TGW.DT | *AX-111806667* | 6A | 309,291,590 | DAVE | 8.17E-04 | 2.10 |
| TGW.DT | *AX-109070240* | 6A | 310,060,628 | DAVE | 7.62E-04 | 2.13 |
| TGW.DT | *AX-111097598* | 6A | 312,077,737 | DAVE | 8.21E-04 | 2.10 |
| TGW.DT | *AX-111478362* | 6A | 318,287,443 | DAVE | 7.90E-04 | 2.11 |
| TGW.DT | *AX-110024798* | 6A | 324,812,584 | DAVE | 9.50E-04 | 2.05 |
| TGW.DT | *AX-109955975* | 6A | 326,412,162 | DT19 | 9.96E-04 | 2.62 |
| TGW.DT | *AX-111267479* | 6A | 326,823,896 | DAVE | 6.04E-04 | 2.21 |
| TGW.DT | *AX-108937778* | 6A | 327,484,309 | DAVE | 6.75E-04 | 2.17 |
| TGW.DT | *AX-109334618* | 6A | 328,130,345 | DT19 | 6.83E-04 | 2.80 |
| TGW.DT | *AX-109334618* | 6A | 328,130,345 | DT20 | 7.24E-04 | 2.21 |
| TGW.DT | *AX-109334618* | 6A | 328,130,345 | DAVE | 3.51E-05 | 3.23 |
| TGW.DT | *AX-110474160* | 6A | 328,510,329 | DAVE | 7.78E-04 | 2.12 |
| TGW.DT | *AX-110911407* | 6A | 329,608,775 | DAVE | 8.32E-04 | 2.10 |
| TGW.DT | *AX-110916005* | 6A | 330,873,938 | DAVE | 8.02E-04 | 2.11 |
| TGW.DT | *AX-108800286* | 6A | 335,807,066 | DAVE | 5.98E-04 | 2.21 |
| TGW.DT | *AX-111278973* | 6A | 336,253,032 | DAVE | 6.49E-04 | 2.18 |
| TGW.DT | *AX-108869501* | 6A | 338,237,015 | DAVE | 7.80E-04 | 2.12 |
| TGW.DT | *AX-111273273* | 6A | 340,704,113 | DAVE | 6.08E-04 | 2.21 |
| TGW.DT | *AX-108817301* | 6A | 341,957,483 | DAVE | 4.95E-04 | 2.28 |
| TGW.DT | *AX-109448914* | 6A | 349,053,789 | DAVE | 7.69E-04 | 2.12 |
| TGW.DT | *AX-111835367* | 6A | 349,835,322 | DAVE | 5.38E-04 | 2.25 |
| TGW.DT | *AX-109043962* | 6A | 352,377,413 | DAVE | 9.63E-04 | 2.04 |
| TGW.DT | *AX-109080612* | 6A | 352,542,166 | DAVE | 9.26E-04 | 2.06 |
| TGW.DT | *AX-111695805* | 6A | 367,236,934 | DAVE | 9.78E-04 | 2.04 |
| TGW.DT | *AX-111226349* | 6A | 367,494,684 | DAVE | 9.85E-04 | 2.04 |
| TGW.DT | *AX-109084122* | 6A | 374,826,615 | DT19 | 6.13E-04 | 2.85 |
| TGW.DT | *AX-109084122* | 6A | 374,826,615 | DAVE | 4.66E-04 | 2.30 |
| TGW.DT | *AX-111466636* | 6A | 377,363,579 | DAVE | 8.70E-04 | 2.08 |
| TGW.DT | *AX-111801320* | 6A | 378,218,123 | DAVE | 7.72E-04 | 2.12 |
| TGW.DT | *AX-111072559* | 6A | 398,796,577 | DAVE | 9.19E-04 | 2.06 |
| TGW.DT | *AX-109063873* | 6A | 433,569,417 | DAVE | 2.54E-04 | 2.52 |
| TGW.DT | *AX-109353146* | 6A | 442,400,023 | DAVE | 2.26E-04 | 2.56 |
| TGW.DT | *AX-108944873* | 6A | 601,258,225 | DT21 | 9.67E-05 | 3.00 |
| TGW.DT | *AX-110362828* | 6B | 137,091,867 | DT21 | 5.22E-04 | 2.37 |
| TGW.DT | *AX-109601574* | 6B | 141,174,195 | DT21 | 1.37E-04 | 2.87 |
| TGW.DT | *AX-109601574* | 6B | 141,174,195 | DAVE | 7.20E-04 | 2.15 |
| TGW.DT | *AX-109602187* | 6B | 141,925,565 | DT21 | 2.82E-04 | 2.60 |
| TGW.DT | *AX-110956033* | 6B | 142,886,717 | DT21 | 1.79E-04 | 2.77 |
| TGW.DT | *AX-110956033* | 6B | 142,886,717 | DAVE | 4.56E-04 | 2.31 |
| TGW.DT | *AX-109936817* | 6B | 143,411,297 | DT21 | 5.02E-05 | 3.25 |
| TGW.DT | *AX-109936817* | 6B | 143,411,297 | DAVE | 2.26E-04 | 2.56 |
| TGW.DT | *AX-110987417* | 6B | 144,979,597 | DT21 | 1.23E-05 | 3.79 |
| TGW.DT | *AX-110987417* | 6B | 144,979,597 | DAVE | 2.37E-04 | 2.54 |
| TGW.DT | *AX-110942372* | 6B | 146,135,281 | DT21 | 4.03E-04 | 2.47 |
| TGW.DT | *AX-110442023* | 6B | 148,004,139 | DT21 | 3.68E-04 | 2.50 |
| TGW.DT | *AX-111799187* | 6B | 148,755,317 | DT21 | 4.85E-05 | 3.26 |
| TGW.DT | *AX-111799187* | 6B | 148,755,317 | DAVE | 4.17E-04 | 2.34 |
| TGW.DT | *AX-111230620* | 6B | 149,006,455 | DT21 | 3.15E-05 | 3.43 |
| TGW.DT | *AX-111230620* | 6B | 149,006,455 | DAVE | 3.52E-04 | 2.40 |
| TGW.DT | *AX-109580213* | 6B | 162,617,855 | DT21 | 9.12E-04 | 2.16 |
| TGW.DT | *AX-109986783* | 6B | 163,153,220 | DT21 | 4.16E-04 | 2.45 |
| TGW.DT | *AX-108777810* | 6B | 163,652,142 | DT21 | 5.29E-04 | 2.36 |
| TGW.DT | *AX-109854760* | 6B | 181,402,955 | DT21 | 4.57E-04 | 2.42 |
| TGW.DT | *AX-108868224* | 6B | 181,833,115 | DT21 | 2.65E-04 | 2.62 |
| TGW.DT | *AX-108868224* | 6B | 181,833,115 | DAVE | 5.14E-04 | 2.27 |
| TGW.DT | *AX-111556078* | 6B | 184,176,020 | DT21 | 3.04E-04 | 2.57 |
| TGW.DT | *AX-108885091* | 6B | 184,366,833 | DT21 | 8.55E-04 | 2.19 |
| TGW.DT | *AX-110536146* | 6B | 184,757,682 | DT21 | 4.08E-04 | 2.46 |
| TGW.DT | *AX-108838540* | 6B | 189,185,798 | DT21 | 2.48E-04 | 2.65 |
| TGW.DT | *AX-109466155* | 6B | 189,451,426 | DT21 | 6.79E-04 | 2.27 |
| TGW.DT | *AX-109382255* | 6B | 497,267,595 | DT20 | 6.10E-04 | 2.27 |
| TGW.DT | *AX-109327918* | 6B | 502,090,933 | DT20 | 8.78E-04 | 2.14 |
| TGW.DT | *AX-111473384* | 6B | 568,573,782 | DT19 | 6.32E-04 | 2.83 |
| TGW.DT | *AX-110566220* | 6B | 569,374,210 | DT19 | 4.82E-04 | 2.96 |
| TGW.DT | *AX-111189818* | 6B | 570,159,601 | DT19 | 9.76E-04 | 2.63 |
| TGW.DT | *AX-109050147* | 6B | 570,707,566 | DT19 | 9.00E-04 | 2.67 |
| TGW.DT | *AX-108965440* | 6B | 571,046,876 | DT19 | 2.81E-04 | 3.20 |
| TGW.DT | *AX-109948773* | 6B | 571,664,657 | DT19 | 5.31E-04 | 2.91 |
| TGW.DT | *AX-110569331* | 6B | 572,188,603 | DT19 | 5.01E-04 | 2.94 |
| TGW.DT | *AX-108837662* | 6B | 572,770,512 | DT19 | 9.00E-04 | 2.67 |
| TGW.DT | *AX-111047526* | 6B | 573,257,318 | DT19 | 4.91E-04 | 2.95 |
| TGW.DT | *AX-109893981* | 6B | 574,460,621 | DT19 | 5.37E-04 | 2.91 |
| TGW.DT | *AX-108732920* | 6B | 576,825,870 | DT19 | 5.01E-04 | 2.94 |
| TGW.DT | *AX-111652073* | 6B | 610,363,648 | DT19 | 8.72E-04 | 2.68 |
| TGW.DT | *AX-108921085* | 6B | 610,981,799 | DT19 | 4.07E-04 | 3.03 |
| TGW.DT | *AX-111566434* | 6B | 612,059,447 | DT19 | 7.70E-04 | 2.74 |
| TGW.DT | *AX-111457109* | 6B | 649,150,521 | DT20 | 4.02E-04 | 2.42 |
| TGW.DT | *AX-111457109* | 6B | 649,150,521 | DAVE | 6.73E-04 | 2.17 |
| TGW.DT | *AX-109487902* | 6B | 650,042,822 | DT20 | 8.28E-04 | 2.16 |
| TGW.DT | *AX-110489742* | 6B | 650,637,938 | DT20 | 8.27E-04 | 2.16 |
| TGW.DT | *AX-109416493* | 6B | 651,023,070 | DT20 | 7.94E-04 | 2.17 |
| TGW.DT | *AX-108768271* | 6B | 651,887,533 | DT20 | 7.68E-04 | 2.19 |
| TGW.DT | *AX-110058991* | 6B | 653,089,227 | DT20 | 6.62E-04 | 2.24 |
| TGW.DT | *AX-110422139* | 6B | 687,186,077 | DAVE | 2.27E-04 | 2.56 |
| TGW.DT | *AX-94488517* | 6B | 696,151,765 | DT20 | 2.25E-04 | 2.63 |
| TGW.DT | *AX-109830719* | 6B | 696,291,862 | DT20 | 7.47E-04 | 2.20 |
| TGW.DT | *AX-111569912* | 6D | 71,008,312 | DT21 | 2.58E-05 | 3.51 |
| TGW.DT | *AX-111569912* | 6D | 71,008,312 | DAVE | 3.07E-04 | 2.45 |
| TGW.DT | *AX-111000883* | 6D | 71,027,180 | DT21 | 1.71E-05 | 3.66 |
| TGW.DT | *AX-111000883* | 6D | 71,027,180 | DAVE | 2.91E-04 | 2.47 |
| TGW.DT | *AX-109447460* | 6D | 89,262,091 | DT20 | 5.03E-08 | 5.85 |
| TGW.DT | *AX-109447460* | 6D | 89,262,091 | DAVE | 4.33E-06 | 4.00 |
| TGW.DT | *AX-110423754* | 6D | 322,413,086 | DT20 | 8.60E-04 | 2.14 |
| TGW.DT | *AX-111285003* | 6D | 335,923,113 | DT20 | 9.28E-04 | 2.12 |
| TGW.DT | *AX-111707460* | 6D | 380,560,901 | DT19 | 9.51E-04 | 2.64 |
| TGW.DT | *AX-108729832* | 7A | 63,056,969 | DT19 | 8.41E-04 | 2.70 |
| TGW.DT | *AX-108808481* | 7A | 218,244,507 | DT21 | 9.47E-04 | 2.15 |
| TGW.DT | *AX-111543573* | 7A | 608,141,697 | DT21 | 6.07E-04 | 2.31 |
| TGW.DT | *AX-111460219* | 7A | 619,291,317 | DT21 | 3.71E-04 | 2.50 |
| TGW.DT | *AX-108866878* | 7A | 619,879,562 | DT21 | 4.82E-04 | 2.40 |
| TGW.DT | *AX-110501928* | 7A | 620,936,613 | DT21 | 3.39E-04 | 2.53 |
| TGW.DT | *AX-109386434* | 7A | 692,683,014 | DT21 | 2.95E-04 | 2.58 |
| TGW.DT | *AX-109386434* | 7A | 692,683,014 | DAVE | 8.06E-04 | 2.11 |
| TGW.DT | *AX-108743244* | 7B | 636,934,739 | DT19 | 7.89E-04 | 2.73 |
| TGW.DT | *AX-110648188* | 7B | 637,227,520 | DT19 | 9.98E-04 | 2.62 |
| TGW.DT | *AX-109817033* | 7B | 711,066,385 | DT21 | 4.29E-04 | 2.44 |
| TGW.DT | *AX-111000584* | 7B | 715,289,272 | DT21 | 8.86E-05 | 3.03 |
| TGW.DT | *AX-89658728* | 7B | 721,217,470 | DT21 | 5.49E-04 | 2.35 |
| TGW.DT | *AX-108920250* | 7D | 54,995,342 | DAVE | 4.81E-04 | 2.29 |
| TGW.DT | *AX-110231089* | 7D | 109,822,213 | DT21 | 3.74E-04 | 2.49 |
| TGW.DT | *AX-110231089* | 7D | 109,822,213 | DAVE | 8.15E-04 | 2.10 |
| TGW.DT | *AX-110949705* | 7D | 548,055,156 | DT21 | 8.58E-04 | 2.18 |
| TGW.DT | *AX-110949705* | 7D | 548,055,156 | DAVE | 7.93E-04 | 2.11 |
| TGW.DT | *AX-108770812* | 7D | 561,926,335 | DT19 | 5.02E-04 | 2.94 |
| TGW.DT | *AX-108770812* | 7D | 561,926,335 | DT21 | 6.60E-04 | 2.28 |
| TGW.DT | *AX-108770812* | 7D | 561,926,335 | DAVE | 1.55E-04 | 2.69 |
| GL.DT | *AX-111084696* | 1A | 16,422,919 | DT19 | 4.59E-04 | 3.09 |
| GL.DT | *AX-111084696* | 1A | 16,422,919 | DAVE | 4.11E-04 | 2.31 |
| GL.DT | *AX-110046566* | 1A | 503,449,799 | DT19 | 5.00E-04 | 3.05 |
| GL.DT | *AX-110046566* | 1A | 503,449,799 | DT20 | 7.80E-04 | 2.13 |
| GL.DT | *AX-110046566* | 1A | 503,449,799 | DT21 | 7.05E-04 | 2.20 |
| GL.DT | *AX-110046566* | 1A | 503,449,799 | DAVE | 2.95E-04 | 2.43 |
| GL.DT | *AX-111802924* | 1A | 504,003,811 | DT19 | 7.34E-04 | 2.86 |
| GL.DT | *AX-111802924* | 1A | 504,003,811 | DT20 | 9.44E-04 | 2.06 |
| GL.DT | *AX-111802924* | 1A | 504,003,811 | DT21 | 8.55E-04 | 2.13 |
| GL.DT | *AX-111802924* | 1A | 504,003,811 | DAVE | 4.70E-04 | 2.26 |
| GL.DT | *AX-111827505* | 1A | 507,223,542 | DT19 | 9.07E-04 | 2.76 |
| GL.DT | *AX-109326813* | 1A | 513,859,613 | DT21 | 4.60E-04 | 2.35 |
| GL.DT | *AX-109326813* | 1A | 513,859,613 | DAVE | 2.62E-04 | 2.47 |
| GL.DT | *AX-109904229* | 1A | 516,450,384 | DT21 | 4.69E-04 | 2.35 |
| GL.DT | *AX-109904229* | 1A | 516,450,384 | DAVE | 4.83E-04 | 2.25 |
| GL.DT | *AX-95213134* | 1B | 16,464,540 | DT20 | 8.11E-04 | 2.12 |
| GL.DT | *AX-94484295* | 1B | 32,776,200 | DT20 | 2.65E-04 | 2.52 |
| GL.DT | *AX-110501384* | 1B | 33,947,872 | DT20 | 8.04E-04 | 2.12 |
| GL.DT | *AX-95108394* | 1B | 227,792,006 | DT20 | 9.76E-04 | 2.05 |
| GL.DT | *AX-108848761* | 1B | 459,191,489 | DT21 | 3.65E-04 | 2.44 |
| GL.DT | *AX-108848761* | 1B | 459,191,489 | DAVE | 9.71E-04 | 2.01 |
| GL.DT | *AX-110124276* | 1B | 464,840,844 | DT21 | 5.53E-04 | 2.29 |
| GL.DT | *AX-109903718* | 1B | 564,916,918 | DT21 | 1.67E-04 | 2.72 |
| GL.DT | *AX-111130381* | 1B | 565,517,030 | DT21 | 2.49E-04 | 2.58 |
| GL.DT | *AX-94796731* | 1B | 565,672,749 | DT21 | 7.73E-04 | 2.17 |
| GL.DT | *AX-108846907* | 1B | 566,532,044 | DT21 | 5.94E-04 | 2.26 |
| GL.DT | *AX-108846907* | 1B | 566,532,044 | DAVE | 4.63E-04 | 2.27 |
| GL.DT | *AX-110395364* | 1B | 566,996,573 | DT21 | 5.04E-04 | 2.32 |
| GL.DT | *AX-111595814* | 1B | 568,521,078 | DT21 | 1.95E-04 | 2.67 |
| GL.DT | *AX-111595814* | 1B | 568,521,078 | DAVE | 5.61E-04 | 2.20 |
| GL.DT | *AX-111011700* | 1B | 568,768,279 | DT21 | 3.10E-04 | 2.50 |
| GL.DT | *AX-111011700* | 1B | 568,768,279 | DAVE | 7.23E-04 | 2.11 |
| GL.DT | *AX-111009896* | 1B | 569,506,918 | DT21 | 1.67E-04 | 2.72 |
| GL.DT | *AX-111009896* | 1B | 569,506,918 | DAVE | 4.11E-04 | 2.31 |
| GL.DT | *AX-109850741* | 1B | 570,291,724 | DT21 | 1.57E-04 | 2.74 |
| GL.DT | *AX-109850741* | 1B | 570,291,724 | DAVE | 6.65E-04 | 2.14 |
| GL.DT | *AX-110922932* | 1B | 570,569,224 | DT21 | 1.33E-04 | 2.81 |
| GL.DT | *AX-109948353* | 1B | 571,073,013 | DT21 | 9.89E-05 | 2.92 |
| GL.DT | *AX-109948353* | 1B | 571,073,013 | DAVE | 6.13E-04 | 2.17 |
| GL.DT | *AX-108804866* | 1B | 572,254,394 | DT21 | 8.57E-05 | 2.97 |
| GL.DT | *AX-108804866* | 1B | 572,254,394 | DAVE | 6.85E-04 | 2.13 |
| GL.DT | *AX-109439401* | 1B | 575,503,316 | DT21 | 6.74E-04 | 2.22 |
| GL.DT | *AX-109916275* | 1B | 575,828,993 | DT21 | 6.28E-04 | 2.24 |
| GL.DT | *AX-108904194* | 1B | 577,031,581 | DT21 | 4.64E-04 | 2.35 |
| GL.DT | *AX-110091358* | 1B | 577,924,275 | DT21 | 6.41E-04 | 2.23 |
| GL.DT | *AX-110402488* | 1B | 580,075,805 | DT21 | 6.88E-05 | 3.05 |
| GL.DT | *AX-110402488* | 1B | 580,075,805 | DAVE | 2.66E-04 | 2.46 |
| GL.DT | *AX-111157060* | 1B | 580,520,152 | DT21 | 4.46E-04 | 2.36 |
| GL.DT | *AX-111157060* | 1B | 580,520,152 | DAVE | 9.92E-04 | 2.00 |
| GL.DT | *AX-111478905* | 1B | 584,167,426 | DT21 | 4.82E-05 | 3.18 |
| GL.DT | *AX-109041231* | 1B | 584,761,170 | DT21 | 2.14E-04 | 2.63 |
| GL.DT | *AX-109273019* | 1B | 670,429,611 | DT21 | 9.94E-05 | 2.91 |
| GL.DT | *AX-109273019* | 1B | 670,429,611 | DAVE | 9.67E-05 | 2.82 |
| GL.DT | *AX-112289703* | 1B | 673,176,421 | DT20 | 4.38E-04 | 2.34 |
| GL.DT | *AX-109353011* | 1B | 685,283,930 | DT20 | 9.63E-04 | 2.06 |
| GL.DT | *AX-109353011* | 1B | 685,283,930 | DAVE | 8.58E-04 | 2.05 |
| GL.DT | *AX-108737720* | 1B | 686,751,506 | DT19 | 6.57E-04 | 2.92 |
| GL.DT | *AX-108737720* | 1B | 686,751,506 | DT20 | 5.49E-04 | 2.26 |
| GL.DT | *AX-108737720* | 1B | 686,751,506 | DAVE | 2.85E-04 | 2.44 |
| GL.DT | *AX-111190944* | 1B | 686,755,725 | DT19 | 6.54E-04 | 2.92 |
| GL.DT | *AX-111190944* | 1B | 686,755,725 | DT20 | 8.66E-04 | 2.09 |
| GL.DT | *AX-111190944* | 1B | 686,755,725 | DAVE | 5.12E-04 | 2.23 |
| GL.DT | *AX-109847715* | 1B | 686,786,607 | DT19 | 7.68E-04 | 2.84 |
| GL.DT | *AX-109847715* | 1B | 686,786,607 | DT20 | 7.33E-04 | 2.15 |
| GL.DT | *AX-109847715* | 1B | 686,786,607 | DAVE | 3.36E-04 | 2.38 |
| GL.DT | *AX-109007674* | 1D | 8,714,404 | DT20 | 3.40E-04 | 2.43 |
| GL.DT | *AX-111542671* | 1D | 317,076,819 | DT19 | 4.01E-04 | 3.15 |
| GL.DT | *AX-110595695* | 1D | 320,173,505 | DT19 | 5.38E-04 | 3.01 |
| GL.DT | *AX-94442698* | 1D | 342,069,818 | DT21 | 2.24E-04 | 2.62 |
| GL.DT | *AX-94442698* | 1D | 342,069,818 | DAVE | 6.19E-04 | 2.17 |
| GL.DT | *AX-110530257* | 2A | 34,164,516 | DT19 | 6.25E-04 | 2.94 |
| GL.DT | *AX-95660056* | 2A | 603,524,503 | DT20 | 9.13E-04 | 2.08 |
| GL.DT | *AX-110407291* | 2A | 603,732,041 | DT19 | 6.01E-04 | 2.96 |
| GL.DT | *AX-110407291* | 2A | 603,732,041 | DAVE | 9.41E-04 | 2.02 |
| GL.DT | *AX-110168387* | 2A | 604,129,562 | DT19 | 8.65E-04 | 2.79 |
| GL.DT | *AX-109280291* | 2A | 604,861,056 | DT19 | 8.65E-04 | 2.79 |
| GL.DT | *AX-109963316* | 2A | 605,379,330 | DT19 | 3.11E-04 | 3.27 |
| GL.DT | *AX-109303155* | 2A | 605,534,683 | DT19 | 4.88E-04 | 3.06 |
| GL.DT | *AX-110451187* | 2A | 607,768,697 | DT19 | 3.73E-04 | 3.19 |
| GL.DT | *AX-110451187* | 2A | 607,768,697 | DT21 | 2.52E-05 | 3.42 |
| GL.DT | *AX-110451187* | 2A | 607,768,697 | DAVE | 1.99E-04 | 2.57 |
| GL.DT | *AX-109290429* | 2A | 608,865,127 | DT19 | 6.78E-05 | 4.01 |
| GL.DT | *AX-109290429* | 2A | 608,865,127 | DT21 | 1.29E-05 | 3.67 |
| GL.DT | *AX-109290429* | 2A | 608,865,127 | DAVE | 8.27E-05 | 2.88 |
| GL.DT | *AX-109425314* | 2A | 693,336,312 | DT19 | 2.91E-04 | 3.31 |
| GL.DT | *AX-109425314* | 2A | 693,336,312 | DT21 | 9.77E-04 | 2.08 |
| GL.DT | *AX-109576757* | 2A | 751,912,827 | DT20 | 5.64E-05 | 3.08 |
| GL.DT | *AX-110416654* | 2A | 756,836,682 | DT20 | 1.61E-04 | 2.70 |
| GL.DT | *AX-109316607* | 2A | 771,326,282 | DT21 | 6.70E-04 | 2.22 |
| GL.DT | *AX-110126250* | 2B | 41,440,270 | DT19 | 8.69E-05 | 3.89 |
| GL.DT | *AX-94501206* | 2B | 730,998,420 | DT20 | 6.13E-04 | 2.22 |
| GL.DT | *AX-111472511* | 2B | 732,339,052 | DT20 | 5.08E-04 | 2.28 |
| GL.DT | *AX-109909946* | 2B | 735,520,883 | DT20 | 7.52E-04 | 2.14 |
| GL.DT | *AX-109578675* | 2B | 740,730,962 | DT20 | 6.55E-04 | 2.19 |
| GL.DT | *AX-109013500* | 2D | 63,568,198 | DT20 | 5.60E-04 | 2.25 |
| GL.DT | *AX-109013500* | 2D | 63,568,198 | DT21 | 4.85E-04 | 2.33 |
| GL.DT | *AX-109877450* | 2D | 582,013,112 | DT21 | 6.65E-05 | 3.06 |
| GL.DT | *AX-109877450* | 2D | 582,013,112 | DAVE | 5.60E-04 | 2.20 |
| GL.DT | *AX-94669346* | 2D | 609,234,499 | DT20 | 9.10E-04 | 2.08 |
| GL.DT | *AX-95631471* | 3A | 44,629,466 | DT21 | 9.15E-05 | 2.94 |
| GL.DT | *AX-95631471* | 3A | 44,629,466 | DAVE | 9.53E-05 | 2.83 |
| GL.DT | *AX-110707258* | 3A | 733,432,801 | DT20 | 1.47E-04 | 2.73 |
| GL.DT | *AX-110707258* | 3A | 733,432,801 | DT21 | 9.53E-04 | 2.09 |
| GL.DT | *AX-110707258* | 3A | 733,432,801 | DAVE | 9.39E-04 | 2.02 |
| GL.DT | *AX-111626915* | 3A | 734,428,567 | DT20 | 3.28E-05 | 3.28 |
| GL.DT | *AX-111626915* | 3A | 734,428,567 | DAVE | 3.54E-04 | 2.36 |
| GL.DT | *AX-109907412* | 3A | 743,704,414 | DT19 | 2.33E-04 | 3.41 |
| GL.DT | *AX-109907412* | 3A | 743,704,414 | DAVE | 5.71E-04 | 2.20 |
| GL.DT | *AX-109281734* | 3A | 744,244,068 | DT19 | 5.23E-05 | 4.14 |
| GL.DT | *AX-109281734* | 3A | 744,244,068 | DT20 | 4.11E-04 | 2.36 |
| GL.DT | *AX-109281734* | 3A | 744,244,068 | DAVE | 1.48E-04 | 2.67 |
| GL.DT | *AX-110371960* | 3B | 2,335,309 | DT19 | 9.87E-05 | 3.83 |
| GL.DT | *AX-110935747* | 3B | 54,865,590 | DT21 | 2.38E-04 | 2.59 |
| GL.DT | *AX-110935747* | 3B | 54,865,590 | DAVE | 3.09E-04 | 2.41 |
| GL.DT | *AX-111523002* | 3B | 55,369,721 | DT21 | 2.00E-04 | 2.66 |
| GL.DT | *AX-111523002* | 3B | 55,369,721 | DAVE | 2.28E-04 | 2.52 |
| GL.DT | *AX-109910758* | 3B | 59,054,885 | DT20 | 6.00E-05 | 3.05 |
| GL.DT | *AX-109910758* | 3B | 59,054,885 | DAVE | 4.45E-04 | 2.28 |
| GL.DT | *AX-111121613* | 3B | 116,973,899 | DT21 | 8.08E-04 | 2.15 |
| GL.DT | *AX-109334330* | 3B | 118,193,311 | DT21 | 4.57E-04 | 2.36 |
| GL.DT | *AX-111460388* | 3B | 118,758,942 | DT21 | 4.31E-04 | 2.38 |
| GL.DT | *AX-110506853* | 3B | 119,222,104 | DT21 | 8.24E-04 | 2.14 |
| GL.DT | *AX-108830826* | 3B | 120,271,219 | DT21 | 4.65E-04 | 2.35 |
| GL.DT | *AX-110434278* | 3B | 121,249,574 | DT21 | 3.59E-04 | 2.44 |
| GL.DT | *AX-111143672* | 3B | 121,851,856 | DT21 | 3.09E-04 | 2.50 |
| GL.DT | *AX-109359639* | 3B | 123,714,815 | DT21 | 8.02E-04 | 2.15 |
| GL.DT | *AX-109898213* | 3B | 125,550,726 | DT21 | 2.64E-04 | 2.55 |
| GL.DT | *AX-109859165* | 3B | 126,482,604 | DT21 | 2.85E-04 | 2.53 |
| GL.DT | *AX-109338627* | 3B | 126,845,393 | DT21 | 1.81E-04 | 2.69 |
| GL.DT | *AX-109506024* | 3B | 127,815,155 | DT21 | 1.95E-04 | 2.67 |
| GL.DT | *AX-110401384* | 3B | 127,853,486 | DT21 | 1.88E-04 | 2.68 |
| GL.DT | *AX-109395116* | 3B | 128,705,070 | DT21 | 3.15E-04 | 2.49 |
| GL.DT | *AX-110007345* | 3B | 129,442,308 | DT21 | 2.94E-04 | 2.52 |
| GL.DT | *AX-109422148* | 3B | 130,152,525 | DT21 | 1.25E-04 | 2.83 |
| GL.DT | *AX-111047357* | 3B | 131,328,597 | DT21 | 2.36E-04 | 2.60 |
| GL.DT | *AX-111490441* | 3B | 131,508,802 | DT21 | 2.11E-04 | 2.64 |
| GL.DT | *AX-108746747* | 3B | 133,188,397 | DT21 | 2.80E-04 | 2.53 |
| GL.DT | *AX-109394157* | 3B | 133,803,932 | DT21 | 7.63E-04 | 2.17 |
| GL.DT | *AX-108790822* | 3B | 141,628,197 | DT21 | 1.89E-04 | 2.68 |
| GL.DT | *AX-108790822* | 3B | 141,628,197 | DAVE | 1.83E-04 | 2.60 |
| GL.DT | *AX-109420075* | 3B | 142,841,868 | DT21 | 4.14E-04 | 2.39 |
| GL.DT | *AX-109420075* | 3B | 142,841,868 | DAVE | 3.72E-04 | 2.35 |
| GL.DT | *AX-109303193* | 3B | 143,573,669 | DT21 | 2.67E-04 | 2.55 |
| GL.DT | *AX-109303193* | 3B | 143,573,669 | DAVE | 2.96E-04 | 2.43 |
| GL.DT | *AX-111148430* | 3B | 144,573,595 | DT21 | 2.16E-04 | 2.63 |
| GL.DT | *AX-109995421* | 3B | 144,775,288 | DT21 | 3.80E-04 | 2.42 |
| GL.DT | *AX-110397799* | 3B | 144,903,259 | DT21 | 2.17E-04 | 2.63 |
| GL.DT | *AX-110534176* | 3B | 145,696,681 | DT21 | 2.07E-04 | 2.64 |
| GL.DT | *AX-110402477* | 3B | 146,283,576 | DT21 | 1.89E-04 | 2.68 |
| GL.DT | *AX-108901344* | 3B | 146,828,510 | DT21 | 3.04E-04 | 2.50 |
| GL.DT | *AX-109908727* | 3B | 148,064,322 | DT21 | 3.91E-04 | 2.41 |
| GL.DT | *AX-110492055* | 3B | 152,129,909 | DT21 | 5.80E-04 | 2.27 |
| GL.DT | *AX-110985096* | 3B | 719,824,246 | DT19 | 1.11E-04 | 3.77 |
| GL.DT | *AX-109890936* | 3B | 721,920,517 | DT19 | 1.21E-04 | 3.73 |
| GL.DT | *AX-94851468* | 3B | 723,032,017 | DT19 | 2.44E-04 | 3.39 |
| GL.DT | *AX-110418888* | 3B | 785,432,286 | DT19 | 8.19E-05 | 3.92 |
| GL.DT | *AX-110418888* | 3B | 785,432,286 | DT20 | 4.41E-05 | 3.17 |
| GL.DT | *AX-110418888* | 3B | 785,432,286 | DT21 | 7.11E-06 | 3.90 |
| GL.DT | *AX-110418888* | 3B | 785,432,286 | DAVE | 6.71E-06 | 3.78 |
| GL.DT | *AX-109881148* | 3B | 793,075,912 | DAVE | 9.56E-04 | 2.02 |
| GL.DT | *AX-111703658* | 3D | 32,268,623 | DT21 | 2.31E-04 | 2.60 |
| GL.DT | *AX-111703658* | 3D | 32,268,623 | DAVE | 3.29E-04 | 2.39 |
| GL.DT | *AX-109377485* | 3D | 71,517,175 | DT21 | 6.77E-04 | 2.21 |
| GL.DT | *AX-94604542* | 3D | 75,357,976 | DT21 | 2.13E-04 | 2.63 |
| GL.DT | *AX-109506193* | 3D | 94,859,581 | DT21 | 1.53E-04 | 2.75 |
| GL.DT | *AX-109819016* | 3D | 97,116,754 | DT21 | 4.62E-04 | 2.35 |
| GL.DT | *AX-111103248* | 3D | 178,761,794 | DT20 | 6.12E-04 | 2.22 |
| GL.DT | *AX-111103248* | 3D | 178,761,794 | DAVE | 5.44E-04 | 2.21 |
| GL.DT | *AX-109903567* | 3D | 196,832,500 | DT20 | 8.18E-04 | 2.11 |
| GL.DT | *AX-109903567* | 3D | 196,832,500 | DAVE | 7.98E-04 | 2.08 |
| GL.DT | *AX-111778716* | 3D | 202,665,077 | DT20 | 8.95E-04 | 2.08 |
| GL.DT | *AX-111778716* | 3D | 202,665,077 | DAVE | 8.08E-04 | 2.07 |
| GL.DT | *AX-110432825* | 3D | 206,655,789 | DT20 | 5.95E-04 | 2.23 |
| GL.DT | *AX-110432825* | 3D | 206,655,789 | DAVE | 4.86E-04 | 2.25 |
| GL.DT | *AX-109341556* | 3D | 221,046,278 | DT20 | 6.12E-04 | 2.22 |
| GL.DT | *AX-109341556* | 3D | 221,046,278 | DAVE | 5.44E-04 | 2.21 |
| GL.DT | *AX-109626991* | 3D | 224,648,576 | DAVE | 9.04E-04 | 2.04 |
| GL.DT | *AX-109392999* | 3D | 236,169,118 | DT20 | 4.45E-04 | 2.33 |
| GL.DT | *AX-109392999* | 3D | 236,169,118 | DAVE | 3.22E-04 | 2.40 |
| GL.DT | *AX-109407590* | 3D | 246,923,598 | DT20 | 6.58E-04 | 2.19 |
| GL.DT | *AX-109407590* | 3D | 246,923,598 | DAVE | 5.69E-04 | 2.20 |
| GL.DT | *AX-110830021* | 3D | 260,816,674 | DT20 | 7.48E-04 | 2.15 |
| GL.DT | *AX-110830021* | 3D | 260,816,674 | DAVE | 5.82E-04 | 2.19 |
| GL.DT | *AX-108806099* | 3D | 549,623,348 | DT19 | 6.46E-04 | 2.92 |
| GL.DT | *AX-109500294* | 3D | 599,688,383 | DT19 | 4.04E-04 | 3.15 |
| GL.DT | *AX-109500294* | 3D | 599,688,383 | DT21 | 1.25E-04 | 2.83 |
| GL.DT | *AX-109500294* | 3D | 599,688,383 | DAVE | 2.21E-04 | 2.53 |
| GL.DT | *AX-110740225* | 4A | 466,977,646 | DT21 | 3.61E-04 | 2.44 |
| GL.DT | *AX-109527362* | 4A | 467,258,017 | DT21 | 1.99E-04 | 2.66 |
| GL.DT | *AX-109527362* | 4A | 467,258,017 | DAVE | 8.93E-04 | 2.04 |
| GL.DT | *AX-111192688* | 4A | 468,154,810 | DT21 | 4.65E-04 | 2.35 |
| GL.DT | *AX-110941777* | 4A | 469,104,506 | DT21 | 3.75E-04 | 2.43 |
| GL.DT | *AX-110457242* | 4A | 470,805,587 | DT21 | 5.90E-04 | 2.26 |
| GL.DT | *AX-108972249* | 4A | 470,982,625 | DT21 | 5.71E-04 | 2.27 |
| GL.DT | *AX-109547195* | 4A | 471,936,748 | DT21 | 4.24E-04 | 2.38 |
| GL.DT | *AX-110517615* | 4A | 477,578,034 | DT21 | 5.02E-04 | 2.32 |
| GL.DT | *AX-109490376* | 4A | 482,871,379 | DT21 | 2.84E-04 | 2.53 |
| GL.DT | *AX-109500638* | 4A | 483,900,334 | DT21 | 4.00E-04 | 2.40 |
| GL.DT | *AX-109307896* | 4A | 484,860,570 | DT21 | 3.30E-04 | 2.47 |
| GL.DT | *AX-108898942* | 4A | 488,114,645 | DT21 | 5.23E-04 | 2.31 |
| GL.DT | *AX-110016016* | 4A | 488,553,399 | DT21 | 3.58E-04 | 2.44 |
| GL.DT | *AX-109509506* | 4A | 488,867,412 | DT21 | 4.52E-04 | 2.36 |
| GL.DT | *AX-110468887* | 4A | 491,976,508 | DT21 | 4.45E-05 | 3.21 |
| GL.DT | *AX-111743032* | 4A | 492,525,624 | DT21 | 3.37E-05 | 3.31 |
| GL.DT | *AX-111743032* | 4A | 492,525,624 | DAVE | 7.83E-04 | 2.09 |
| GL.DT | *AX-109563449* | 4A | 493,613,472 | DT21 | 5.90E-04 | 2.26 |
| GL.DT | *AX-111528491* | 4A | 496,110,069 | DT21 | 5.00E-05 | 3.17 |
| GL.DT | *AX-111451343* | 4A | 499,611,798 | DT21 | 2.81E-04 | 2.53 |
| GL.DT | *AX-108870577* | 4A | 500,119,543 | DT21 | 1.07E-04 | 2.89 |
| GL.DT | *AX-111624503* | 4A | 532,224,647 | DT21 | 2.13E-04 | 2.63 |
| GL.DT | *AX-110557238* | 4A | 603,105,510 | DT19 | 2.13E-04 | 3.45 |
| GL.DT | *AX-111537186* | 4A | 613,525,981 | DT19 | 6.18E-05 | 4.06 |
| GL.DT | *AX-111040045* | 4A | 632,764,370 | DAVE | 8.82E-04 | 2.04 |
| GL.DT | *AX-109375057* | 4A | 633,308,367 | DT20 | 3.76E-04 | 2.39 |
| GL.DT | *AX-109375057* | 4A | 633,308,367 | DAVE | 1.52E-04 | 2.66 |
| GL.DT | *AX-110468999* | 4A | 634,108,610 | DT20 | 4.93E-04 | 2.29 |
| GL.DT | *AX-110468999* | 4A | 634,108,610 | DAVE | 2.23E-04 | 2.52 |
| GL.DT | *AX-111140650* | 4A | 634,368,256 | DT20 | 7.53E-04 | 2.14 |
| GL.DT | *AX-111140650* | 4A | 634,368,256 | DAVE | 3.42E-04 | 2.37 |
| GL.DT | *AX-111453918* | 4A | 634,833,572 | DT20 | 6.13E-04 | 2.22 |
| GL.DT | *AX-111453918* | 4A | 634,833,572 | DAVE | 4.05E-04 | 2.32 |
| GL.DT | *AX-110440161* | 4A | 635,292,292 | DAVE | 7.72E-04 | 2.09 |
| GL.DT | *AX-108950546* | 4A | 635,764,214 | DT20 | 3.13E-04 | 2.46 |
| GL.DT | *AX-108950546* | 4A | 635,764,214 | DAVE | 2.10E-04 | 2.55 |
| GL.DT | *AX-109416582* | 4A | 668,471,682 | DT20 | 5.80E-04 | 2.24 |
| GL.DT | *AX-111074167* | 4B | 51,188,028 | DT21 | 6.52E-04 | 2.23 |
| GL.DT | *AX-111517964* | 4B | 51,191,177 | DT21 | 7.58E-04 | 2.17 |
| GL.DT | *AX-108772683* | 4B | 53,861,811 | DT21 | 7.25E-04 | 2.19 |
| GL.DT | *AX-109580890* | 4B | 57,481,628 | DT21 | 3.66E-04 | 2.44 |
| GL.DT | *AX-110436979* | 4B | 73,152,359 | DT21 | 3.51E-04 | 2.45 |
| GL.DT | *AX-110361956* | 4B | 75,741,547 | DT21 | 5.67E-04 | 2.28 |
| GL.DT | *AX-111057902* | 4B | 652,373,508 | DT21 | 1.32E-04 | 2.81 |
| GL.DT | *AX-111057902* | 4B | 652,373,508 | DAVE | 8.36E-04 | 2.06 |
| GL.DT | *AX-89380210* | 4B | 652,854,834 | DT21 | 6.39E-04 | 2.23 |
| GL.DT | *AX-111150060* | 4B | 660,589,159 | DT19 | 7.45E-04 | 2.86 |
| GL.DT | *AX-95120271* | 4D | 4,132,769 | DT21 | 8.33E-04 | 2.14 |
| GL.DT | *AX-95081842* | 4D | 109,216,418 | DT21 | 4.08E-04 | 2.40 |
| GL.DT | *AX-86162928* | 5A | 417,895,322 | DT20 | 6.82E-04 | 2.18 |
| GL.DT | *AX-109308752* | 5A | 419,203,455 | DT20 | 8.65E-04 | 2.09 |
| GL.DT | *AX-108806718* | 5A | 435,245,426 | DT20 | 8.65E-04 | 2.09 |
| GL.DT | *AX-110921140* | 5A | 623,381,440 | DT20 | 6.77E-04 | 2.18 |
| GL.DT | *AX-110921140* | 5A | 623,381,440 | DAVE | 5.41E-04 | 2.21 |
| GL.DT | *AX-108738342* | 5A | 627,985,757 | DT21 | 4.02E-04 | 2.40 |
| GL.DT | *AX-108761227* | 5A | 631,400,913 | DT21 | 4.71E-05 | 3.19 |
| GL.DT | *AX-109496894* | 5B | 7,736,591 | DT19 | 4.71E-04 | 3.07 |
| GL.DT | *AX-110561585* | 5B | 35,291,342 | DT19 | 8.78E-04 | 2.78 |
| GL.DT | *AX-110561585* | 5B | 35,291,342 | DAVE | 6.06E-04 | 2.17 |
| GL.DT | *AX-110398218* | 5B | 57,493,343 | DT19 | 1.79E-04 | 3.54 |
| GL.DT | *AX-110398218* | 5B | 57,493,343 | DT21 | 8.25E-04 | 2.14 |
| GL.DT | *AX-110398218* | 5B | 57,493,343 | DAVE | 1.19E-04 | 2.75 |
| GL.DT | *AX-110639732* | 5B | 228,815,862 | DT19 | 5.13E-04 | 3.03 |
| GL.DT | *AX-111569451* | 5B | 233,491,515 | DT19 | 5.86E-04 | 2.97 |
| GL.DT | *AX-111563063* | 5B | 239,959,069 | DT19 | 4.96E-04 | 3.05 |
| GL.DT | *AX-111451165* | 5B | 244,757,341 | DT19 | 7.66E-04 | 2.84 |
| GL.DT | *AX-110730995* | 5B | 249,843,782 | DT19 | 9.48E-04 | 2.74 |
| GL.DT | *AX-109581524* | 5B | 250,568,458 | DT19 | 6.28E-04 | 2.94 |
| GL.DT | *AX-110020680* | 5B | 251,008,827 | DT19 | 7.02E-04 | 2.88 |
| GL.DT | *AX-111031509* | 5B | 260,577,074 | DT19 | 7.02E-04 | 2.88 |
| GL.DT | *AX-110558483* | 5B | 262,756,054 | DT19 | 6.68E-04 | 2.91 |
| GL.DT | *AX-110529376* | 5B | 264,839,983 | DT19 | 7.14E-04 | 2.88 |
| GL.DT | *AX-110516200* | 5B | 277,898,057 | DT19 | 5.59E-04 | 2.99 |
| GL.DT | *AX-110516200* | 5B | 277,898,057 | DAVE | 8.44E-04 | 2.06 |
| GL.DT | *AX-109928742* | 5B | 394,970,067 | DAVE | 7.39E-04 | 2.11 |
| GL.DT | *AX-109348429* | 5B | 404,478,483 | DT19 | 4.35E-04 | 3.11 |
| GL.DT | *AX-109348429* | 5B | 404,478,483 | DAVE | 2.16E-04 | 2.54 |
| GL.DT | *AX-109825450* | 5B | 404,992,049 | DT19 | 6.54E-04 | 2.92 |
| GL.DT | *AX-109825450* | 5B | 404,992,049 | DAVE | 2.63E-04 | 2.47 |
| GL.DT | *AX-111697598* | 5B | 405,520,813 | DT19 | 6.00E-04 | 2.96 |
| GL.DT | *AX-111697598* | 5B | 405,520,813 | DAVE | 2.09E-04 | 2.55 |
| GL.DT | *AX-108729070* | 5B | 406,190,132 | DT19 | 3.70E-04 | 3.19 |
| GL.DT | *AX-110054717* | 5B | 406,534,932 | DT19 | 3.27E-04 | 3.25 |
| GL.DT | *AX-109329070* | 5B | 447,561,698 | DT21 | 9.23E-04 | 2.10 |
| GL.DT | *AX-110397972* | 5B | 688,971,057 | DT20 | 9.15E-04 | 2.07 |
| GL.DT | *AX-108733827* | 5B | 697,378,921 | DT20 | 3.31E-04 | 2.44 |
| GL.DT | *AX-109879806* | 5B | 699,999,185 | DT20 | 8.02E-04 | 2.12 |
| GL.DT | *AX-109879806* | 5B | 699,999,185 | DT21 | 2.08E-04 | 2.64 |
| GL.DT | *AX-109879806* | 5B | 699,999,185 | DAVE | 3.51E-04 | 2.37 |
| GL.DT | *AX-108757760* | 5B | 701,544,672 | DT19 | 6.91E-04 | 2.89 |
| GL.DT | *AX-108757760* | 5B | 701,544,672 | DT20 | 2.56E-04 | 2.53 |
| GL.DT | *AX-108757760* | 5B | 701,544,672 | DAVE | 4.32E-04 | 2.29 |
| GL.DT | *AX-108733256* | 5B | 702,228,057 | DT19 | 3.86E-04 | 3.17 |
| GL.DT | *AX-108733256* | 5B | 702,228,057 | DT20 | 5.92E-04 | 2.23 |
| GL.DT | *AX-108733256* | 5B | 702,228,057 | DAVE | 5.11E-04 | 2.23 |
| GL.DT | *AX-110946146* | 5B | 703,062,745 | DT20 | 7.85E-04 | 2.13 |
| GL.DT | *AX-109587839* | 5B | 703,529,204 | DT20 | 2.20E-04 | 2.58 |
| GL.DT | *AX-110521341* | 5D | 30,854,436 | DT19 | 7.43E-05 | 3.97 |
| GL.DT | *AX-110521341* | 5D | 30,854,436 | DT21 | 5.13E-04 | 2.31 |
| GL.DT | *AX-110521341* | 5D | 30,854,436 | DAVE | 1.29E-04 | 2.72 |
| GL.DT | *AX-111917292* | 5D | 38,931,819 | DAVE | 6.42E-04 | 2.15 |
| GL.DT | *AX-108930462* | 5D | 40,914,557 | DT21 | 3.83E-04 | 2.42 |
| GL.DT | *AX-108930462* | 5D | 40,914,557 | DAVE | 1.02E-04 | 2.80 |
| GL.DT | *AX-110503408* | 5D | 379,836,128 | DT19 | 9.23E-04 | 2.76 |
| GL.DT | *AX-110503408* | 5D | 379,836,128 | DT21 | 5.02E-05 | 3.17 |
| GL.DT | *AX-110503408* | 5D | 379,836,128 | DAVE | 4.30E-05 | 3.11 |
| GL.DT | *AX-110570148* | 5D | 407,762,345 | DT20 | 8.22E-04 | 2.11 |
| GL.DT | *AX-110570148* | 5D | 407,762,345 | DAVE | 9.01E-04 | 2.04 |
| GL.DT | *AX-111055722* | 5D | 446,113,191 | DT21 | 2.95E-04 | 2.52 |
| GL.DT | *AX-110593017* | 6A | 245,781,410 | DT19 | 5.13E-04 | 3.03 |
| GL.DT | *AX-111146080* | 6A | 259,315,472 | DT19 | 9.51E-04 | 2.74 |
| GL.DT | *AX-108944873* | 6A | 601,258,225 | DT21 | 5.79E-04 | 2.27 |
| GL.DT | *AX-111034352* | 6A | 603,563,082 | DT19 | 2.69E-05 | 4.46 |
| GL.DT | *AX-111034352* | 6A | 603,563,082 | DT21 | 1.75E-04 | 2.70 |
| GL.DT | *AX-111034352* | 6A | 603,563,082 | DAVE | 2.01E-05 | 3.38 |
| GL.DT | *AX-94788907* | 6A | 604,107,719 | DT19 | 1.97E-04 | 3.49 |
| GL.DT | *AX-94788907* | 6A | 604,107,719 | DT21 | 4.77E-04 | 2.34 |
| GL.DT | *AX-94788907* | 6A | 604,107,719 | DAVE | 5.52E-05 | 3.02 |
| GL.DT | *AX-109534275* | 6A | 604,573,611 | DT19 | 2.56E-04 | 3.37 |
| GL.DT | *AX-109534275* | 6A | 604,573,611 | DAVE | 1.01E-04 | 2.81 |
| GL.DT | *AX-109935603* | 6B | 153,407,026 | DAVE | 7.07E-04 | 2.12 |
| GL.DT | *AX-109034932* | 6B | 660,710,281 | DT20 | 9.26E-04 | 2.07 |
| GL.DT | *AX-109324368* | 6B | 681,024,809 | DT21 | 3.75E-04 | 2.43 |
| GL.DT | *AX-108729832* | 7A | 63,056,969 | DT19 | 1.62E-04 | 3.59 |
| GL.DT | *AX-110430243* | 7A | 670,500,650 | DT20 | 6.76E-06 | 3.86 |
| GL.DT | *AX-110430243* | 7A | 670,500,650 | DAVE | 5.71E-04 | 2.20 |
| GL.DT | *AX-111024497* | 7A | 670,917,387 | DT20 | 4.10E-05 | 3.19 |
| GL.DT | *AX-111024497* | 7A | 670,917,387 | DAVE | 3.19E-04 | 2.40 |
| GL.DT | *AX-109386434* | 7A | 692,683,014 | DT21 | 4.60E-04 | 2.35 |
| GL.DT | *AX-108748484* | 7A | 730,508,297 | DT20 | 9.05E-05 | 2.91 |
| GL.DT | *AX-108748484* | 7A | 730,508,297 | DT21 | 7.07E-04 | 2.20 |
| GL.DT | *AX-108748484* | 7A | 730,508,297 | DAVE | 1.12E-04 | 2.77 |
| GL.DT | *AX-110007752* | 7A | 730,855,963 | DT20 | 1.28E-04 | 2.78 |
| GL.DT | *AX-110007752* | 7A | 730,855,963 | DT21 | 6.54E-04 | 2.23 |
| GL.DT | *AX-110007752* | 7A | 730,855,963 | DAVE | 1.18E-04 | 2.75 |
| GL.DT | *AX-109272527* | 7A | 731,240,127 | DT20 | 1.20E-04 | 2.80 |
| GL.DT | *AX-109272527* | 7A | 731,240,127 | DT21 | 4.60E-04 | 2.35 |
| GL.DT | *AX-109272527* | 7A | 731,240,127 | DAVE | 9.80E-05 | 2.82 |
| GL.DT | *AX-158544357* | 7B | 3,853,541 | DT20 | 6.82E-04 | 2.18 |
| GL.DT | *AX-111190466* | 7B | 14,874,652 | DT21 | 8.72E-04 | 2.12 |
| GL.DT | *AX-108783702* | 7B | 49,533,903 | DT21 | 6.13E-04 | 2.25 |
| GL.DT | *AX-111032340* | 7B | 53,244,819 | DT21 | 6.13E-04 | 2.25 |
| GL.DT | *AX-110395066* | 7B | 53,760,546 | DT21 | 6.78E-04 | 2.21 |
| GL.DT | *AX-109375143* | 7B | 54,779,941 | DT21 | 9.34E-04 | 2.10 |
| GL.DT | *AX-110632991* | 7B | 483,533,955 | DT19 | 8.71E-04 | 2.78 |
| GL.DT | *AX-111013927* | 7B | 682,199,428 | DT21 | 5.89E-05 | 3.11 |
| GL.DT | *AX-111013927* | 7B | 682,199,428 | DAVE | 8.57E-04 | 2.05 |
| GL.DT | *AX-108895252* | 7D | 122,838,225 | DAVE | 6.80E-04 | 2.13 |
| GL.DT | *AX-108770812* | 7D | 561,926,335 | DT20 | 6.40E-04 | 2.20 |
| GL.DT | *AX-108815963* | 7D | 577,348,898 | DT19 | 3.37E-06 | 5.50 |
| GL.DT | *AX-108815963* | 7D | 577,348,898 | DT21 | 1.95E-04 | 2.67 |
| GL.DT | *AX-108815963* | 7D | 577,348,898 | DAVE | 3.23E-05 | 3.21 |
| GL.DT | *AX-109867095* | 7D | 577,937,328 | DT19 | 3.36E-06 | 5.50 |
| GL.DT | *AX-109867095* | 7D | 577,937,328 | DT21 | 2.08E-04 | 2.64 |
| GL.DT | *AX-109867095* | 7D | 577,937,328 | DAVE | 3.38E-05 | 3.20 |
| GL.DT | *AX-110983154* | 7D | 590,121,412 | DT20 | 7.23E-04 | 2.16 |
| GW.DT | *AX-94615199* | 1A | 21,624,343 | DT19 | 8.85E-04 | 2.79 |
| GW.DT | *AX-110023564* | 1A | 528,973,058 | DT20 | 9.74E-04 | 1.99 |
| GW.DT | *AX-109271243* | 1D | 9,424,088 | DAVE | 8.79E-04 | 2.11 |
| GW.DT | *AX-108727343* | 1D | 26,410,363 | DT21 | 9.34E-04 | 2.23 |
| GW.DT | *AX-94910470* | 2A | 763,695,272 | DT20 | 4.44E-04 | 2.26 |
| GW.DT | *AX-94910470* | 2A | 763,695,272 | DAVE | 7.43E-04 | 2.17 |
| GW.DT | *AX-86177963* | 2B | 110,818,850 | DAVE | 8.85E-04 | 2.11 |
| GW.DT | *AX-110417260* | 3A | 566,328,400 | DT20 | 7.86E-04 | 2.06 |
| GW.DT | *AX-111634399* | 3A | 589,373,358 | DAVE | 8.76E-04 | 2.11 |
| GW.DT | *AX-108860141* | 3A | 713,551,836 | DT20 | 7.13E-04 | 2.10 |
| GW.DT | *AX-108887339* | 3A | 714,020,755 | DT20 | 4.82E-04 | 2.23 |
| GW.DT | *AX-108794764* | 3D | 72,674,445 | DT19 | 3.09E-04 | 3.30 |
| GW.DT | *AX-109516184* | 3D | 74,463,281 | DT19 | 5.16E-04 | 3.05 |
| GW.DT | *AX-110270468* | 3D | 75,351,635 | DT19 | 2.76E-04 | 3.35 |
| GW.DT | *AX-110464489* | 3D | 82,139,636 | DT19 | 5.91E-04 | 2.98 |
| GW.DT | *AX-109842327* | 3D | 82,908,502 | DT19 | 5.47E-04 | 3.02 |
| GW.DT | *AX-109879761* | 3D | 104,565,286 | DT19 | 5.37E-04 | 3.03 |
| GW.DT | *AX-111045512* | 3D | 504,958,825 | DT19 | 9.51E-04 | 2.76 |
| GW.DT | *AX-95629274* | 4A | 38,367,204 | DAVE | 7.42E-04 | 2.17 |
| GW.DT | *AX-109375057* | 4A | 633,308,367 | DT20 | 2.54E-04 | 2.45 |
| GW.DT | *AX-109375057* | 4A | 633,308,367 | DAVE | 2.84E-04 | 2.51 |
| GW.DT | *AX-110440161* | 4A | 635,292,292 | DT20 | 4.09E-04 | 2.29 |
| GW.DT | *AX-110440161* | 4A | 635,292,292 | DAVE | 4.91E-04 | 2.32 |
| GW.DT | *AX-108950546* | 4A | 635,764,214 | DT20 | 2.21E-04 | 2.50 |
| GW.DT | *AX-108950546* | 4A | 635,764,214 | DAVE | 2.64E-04 | 2.54 |
| GW.DT | *AX-95160379* | 4B | 30,589,960 | DT21 | 3.70E-06 | 4.40 |
| GW.DT | *AX-95160379* | 4B | 30,589,960 | DAVE | 8.62E-04 | 2.11 |
| GW.DT | *AX-110021330* | 4B | 30,863,602 | DT21 | 6.19E-06 | 4.19 |
| GW.DT | *AX-110021330* | 4B | 30,863,602 | DAVE | 9.90E-04 | 2.07 |
| GW.DT | *AX-111585045* | 4B | 40,755,024 | DT19 | 4.97E-04 | 3.07 |
| GW.DT | *AX-108847266* | 4B | 429,339,058 | DT21 | 4.22E-04 | 2.53 |
| GW.DT | *AX-110013985* | 4B | 604,048,394 | DT21 | 9.07E-04 | 2.24 |
| GW.DT | *AX-111224356* | 5A | 88,918,881 | DT20 | 7.90E-04 | 2.06 |
| GW.DT | *AX-109422629* | 5A | 513,845,754 | DT19 | 5.72E-04 | 3.00 |
| GW.DT | *AX-109422629* | 5A | 513,845,754 | DAVE | 9.20E-04 | 2.09 |
| GW.DT | *AX-110016633* | 5A | 547,333,614 | DT19 | 5.19E-04 | 3.05 |
| GW.DT | *AX-109900224* | 5A | 573,095,667 | DT19 | 6.90E-04 | 2.91 |
| GW.DT | *AX-111564977* | 5B | 14,177,507 | DT21 | 2.30E-04 | 2.77 |
| GW.DT | *AX-111564977* | 5B | 14,177,507 | DAVE | 4.93E-04 | 2.32 |
| GW.DT | *AX-108899793* | 5B | 29,124,054 | DT20 | 6.50E-04 | 2.13 |
| GW.DT | *AX-108956314* | 5B | 544,673,479 | DT20 | 4.77E-04 | 2.23 |
| GW.DT | *AX-109536742* | 5D | 21,013,112 | DT21 | 4.20E-04 | 2.53 |
| GW.DT | *AX-109536742* | 5D | 21,013,112 | DAVE | 9.22E-04 | 2.09 |
| GW.DT | *AX-89655686* | 5D | 370,827,067 | DT20 | 8.02E-04 | 2.06 |
| GW.DT | *AX-111557248* | 5D | 539,585,015 | DAVE | 2.71E-04 | 2.53 |
| GW.DT | *AX-111100875* | 5D | 560,509,338 | DT20 | 9.47E-05 | 2.80 |
| GW.DT | *AX-111100875* | 5D | 560,509,338 | DAVE | 6.02E-05 | 3.08 |
| GW.DT | *AX-111085580* | 5D | 561,114,815 | DAVE | 7.85E-05 | 2.98 |
| GW.DT | *AX-109946146* | 6A | 63,057,834 | DT21 | 2.09E-05 | 3.71 |
| GW.DT | *AX-109946146* | 6A | 63,057,834 | DAVE | 9.46E-05 | 2.92 |
| GW.DT | *AX-95012251* | 6A | 65,845,842 | DT21 | 1.89E-04 | 2.84 |
| GW.DT | *AX-109933415* | 6A | 66,564,615 | DT21 | 1.97E-04 | 2.83 |
| GW.DT | *AX-109933415* | 6A | 66,564,615 | DAVE | 8.33E-04 | 2.13 |
| GW.DT | *AX-109520642* | 6A | 67,347,005 | DT21 | 3.25E-04 | 2.63 |
| GW.DT | *AX-111490517* | 6A | 67,464,465 | DT21 | 3.11E-04 | 2.65 |
| GW.DT | *AX-110445351* | 6A | 68,423,592 | DT21 | 3.11E-04 | 2.65 |
| GW.DT | *AX-108988699* | 6A | 68,626,300 | DT21 | 3.03E-04 | 2.66 |
| GW.DT | *AX-109563646* | 6A | 70,569,837 | DT21 | 3.35E-04 | 2.62 |
| GW.DT | *AX-108987721* | 6A | 70,571,197 | DT21 | 3.95E-04 | 2.56 |
| GW.DT | *AX-109469598* | 6A | 71,053,426 | DT21 | 7.41E-04 | 2.31 |
| GW.DT | *AX-109886669* | 6A | 72,845,334 | DT21 | 4.13E-04 | 2.54 |
| GW.DT | *AX-108852271* | 6A | 73,095,270 | DT21 | 3.64E-04 | 2.59 |
| GW.DT | *AX-108815103* | 6A | 73,652,625 | DT21 | 5.74E-04 | 2.41 |
| GW.DT | *AX-110622605* | 6A | 74,321,816 | DT21 | 1.54E-04 | 2.92 |
| GW.DT | *AX-109605929* | 6A | 74,974,399 | DT21 | 1.23E-04 | 3.01 |
| GW.DT | *AX-110944922* | 6A | 75,164,240 | DT21 | 1.14E-04 | 3.04 |
| GW.DT | *AX-110944922* | 6A | 75,164,240 | DAVE | 9.61E-04 | 2.08 |
| GW.DT | *AX-108758959* | 6A | 75,570,466 | DT21 | 2.06E-04 | 2.81 |
| GW.DT | *AX-109424753* | 6A | 76,708,283 | DT21 | 7.17E-05 | 3.22 |
| GW.DT | *AX-109532511* | 6A | 79,404,271 | DT21 | 7.91E-05 | 3.18 |
| GW.DT | *AX-110422588* | 6A | 80,091,310 | DT21 | 1.43E-04 | 2.95 |
| GW.DT | *AX-109382841* | 6A | 80,475,039 | DT21 | 7.08E-05 | 3.23 |
| GW.DT | *AX-109382841* | 6A | 80,475,039 | DAVE | 9.65E-04 | 2.07 |
| GW.DT | *AX-109971505* | 6A | 80,813,538 | DT21 | 8.94E-05 | 3.13 |
| GW.DT | *AX-109351164* | 6A | 83,437,991 | DT21 | 1.15E-04 | 3.03 |
| GW.DT | *AX-110619257* | 6A | 105,181,270 | DAVE | 1.56E-04 | 2.73 |
| GW.DT | *AX-110640576* | 6A | 124,268,452 | DT19 | 9.15E-04 | 2.78 |
| GW.DT | *AX-110640576* | 6A | 124,268,452 | DAVE | 4.22E-04 | 2.37 |
| GW.DT | *AX-111041695* | 6A | 148,524,505 | DT19 | 8.63E-05 | 3.91 |
| GW.DT | *AX-111041695* | 6A | 148,524,505 | DT21 | 6.75E-04 | 2.35 |
| GW.DT | *AX-111041695* | 6A | 148,524,505 | DAVE | 8.24E-06 | 3.82 |
| GW.DT | *AX-110936625* | 6A | 158,068,188 | DT19 | 2.70E-04 | 3.36 |
| GW.DT | *AX-110936625* | 6A | 158,068,188 | DT21 | 5.67E-04 | 2.42 |
| GW.DT | *AX-110936625* | 6A | 158,068,188 | DAVE | 1.14E-05 | 3.70 |
| GW.DT | *AX-109974589* | 6A | 182,604,296 | DT19 | 6.87E-04 | 2.91 |
| GW.DT | *AX-109974589* | 6A | 182,604,296 | DAVE | 2.16E-04 | 2.61 |
| GW.DT | *AX-110931567* | 6A | 196,819,052 | DT19 | 2.31E-04 | 3.44 |
| GW.DT | *AX-110931567* | 6A | 196,819,052 | DAVE | 1.99E-05 | 3.49 |
| GW.DT | *AX-109385016* | 6A | 219,624,399 | DT20 | 4.50E-04 | 2.25 |
| GW.DT | *AX-110482082* | 6A | 237,479,423 | DAVE | 5.94E-04 | 2.25 |
| GW.DT | *AX-109093066* | 6A | 237,647,623 | DAVE | 5.12E-04 | 2.30 |
| GW.DT | *AX-109061200* | 6A | 237,728,800 | DAVE | 7.64E-04 | 2.16 |
| GW.DT | *AX-111243668* | 6A | 237,805,680 | DAVE | 9.01E-04 | 2.10 |
| GW.DT | *AX-111146080* | 6A | 259,315,472 | DAVE | 9.75E-04 | 2.07 |
| GW.DT | *AX-108975012* | 6A | 303,534,001 | DT20 | 4.86E-04 | 2.23 |
| GW.DT | *AX-108975012* | 6A | 303,534,001 | DAVE | 2.28E-04 | 2.59 |
| GW.DT | *AX-109334618* | 6A | 328,130,345 | DT20 | 2.41E-04 | 2.47 |
| GW.DT | *AX-109334618* | 6A | 328,130,345 | DAVE | 6.77E-05 | 3.04 |
| GW.DT | *AX-109084122* | 6A | 374,826,615 | DAVE | 2.63E-04 | 2.54 |
| GW.DT | *AX-111072559* | 6A | 398,796,577 | DAVE | 9.10E-04 | 2.10 |
| GW.DT | *AX-110942969* | 6A | 428,454,560 | DT19 | 2.80E-04 | 3.34 |
| GW.DT | *AX-110942969* | 6A | 428,454,560 | DT21 | 9.72E-04 | 2.21 |
| GW.DT | *AX-110942969* | 6A | 428,454,560 | DAVE | 1.12E-05 | 3.71 |
| GW.DT | *AX-109063873* | 6A | 433,569,417 | DAVE | 4.96E-04 | 2.31 |
| GW.DT | *AX-109353146* | 6A | 442,400,023 | DAVE | 4.59E-04 | 2.34 |
| GW.DT | *AX-109382255* | 6B | 497,267,595 | DT20 | 8.31E-04 | 2.04 |
| GW.DT | *AX-94444496* | 6B | 676,152,344 | DT21 | 7.53E-04 | 2.31 |
| GW.DT | *AX-109447460* | 6D | 89,262,091 | DT20 | 1.57E-05 | 3.44 |
| GW.DT | *AX-109868635* | 7A | 36,485,440 | DT21 | 2.35E-04 | 2.76 |
| GW.DT | *AX-109868635* | 7A | 36,485,440 | DAVE | 4.58E-04 | 2.34 |
| GW.DT | *AX-95080418* | 7A | 108,811,168 | DT21 | 6.19E-04 | 2.38 |
| GW.DT | *AX-95080418* | 7A | 108,811,168 | DAVE | 9.06E-04 | 2.10 |
| GW.DT | *AX-109356871* | 7B | 123,482,032 | DT20 | 3.30E-04 | 2.36 |
| GW.DT | *AX-111574714* | 7B | 124,544,385 | DT20 | 5.73E-04 | 2.17 |
| GW.DT | *AX-110032392* | 7B | 124,740,578 | DT20 | 8.37E-04 | 2.04 |
| GW.DT | *AX-108755073* | 7B | 480,203,425 | DT19 | 3.75E-05 | 4.32 |
| GW.DT | *AX-108755073* | 7B | 480,203,425 | DAVE | 4.27E-05 | 3.21 |
| GW.DT | *AX-109499481* | 7B | 480,250,839 | DT19 | 3.61E-05 | 4.34 |
| GW.DT | *AX-109499481* | 7B | 480,250,839 | DAVE | 3.09E-05 | 3.33 |
| GW.DT | *AX-110632991* | 7B | 483,533,955 | DT19 | 6.11E-04 | 2.97 |
| GW.DT | *AX-110611417* | 7D | 102,965,992 | DT21 | 7.27E-04 | 2.32 |
| GW.DT | *AX-110910179* | 7D | 103,178,967 | DT21 | 4.09E-04 | 2.54 |
| GW.DT | *AX-110910179* | 7D | 103,178,967 | DAVE | 9.99E-04 | 2.06 |
| GW.DT | *AX-109270431* | 7D | 104,730,268 | DT21 | 4.76E-04 | 2.48 |
| GW.DT | *AX-108917680* | 7D | 106,856,353 | DT21 | 9.81E-04 | 2.21 |
| GW.DT | *AX-109728672* | 7D | 109,469,429 | DT21 | 2.35E-04 | 2.76 |
| GW.DT | *AX-109728672* | 7D | 109,469,429 | DAVE | 9.80E-04 | 2.07 |
| GW.DT | *AX-95217260* | 7D | 618,891,392 | DT21 | 2.58E-04 | 2.72 |
| SL.DT | *AX-108733288* | 1A | 31,891,661 | DT20 | 3.09E-05 | 3.41 |
| SL.DT | *AX-110603923* | 1B | 530,597,303 | DT19 | 7.48E-04 | 2.18 |
| SL.DT | *AX-110603923* | 1B | 530,597,303 | DAVE | 6.15E-04 | 2.28 |
| SL.DT | *AX-109436738* | 1B | 645,531,213 | DT19 | 8.63E-04 | 2.13 |
| SL.DT | *AX-109042683* | 1D | 47,014,297 | DAVE | 6.65E-04 | 2.25 |
| SL.DT | *AX-109367382* | 1D | 206,956,737 | DT20 | 3.68E-04 | 2.48 |
| SL.DT | *AX-111404590* | 1D | 417,570,479 | DT19 | 5.82E-04 | 2.28 |
| SL.DT | *AX-111258525* | 1D | 417,972,340 | DT19 | 9.39E-04 | 2.10 |
| SL.DT | *AX-108807500* | 2A | 36,908,023 | DAVE | 4.89E-04 | 2.36 |
| SL.DT | *AX-111132810* | 2A | 42,155,602 | DAVE | 9.01E-04 | 2.14 |
| SL.DT | *AX-108747282* | 2A | 46,071,413 | DT20 | 7.71E-04 | 2.21 |
| SL.DT | *AX-89641927* | 2A | 55,176,566 | DT19 | 1.59E-06 | 4.48 |
| SL.DT | *AX-89641927* | 2A | 55,176,566 | DAVE | 7.43E-07 | 4.82 |
| SL.DT | *AX-108747720* | 2A | 58,539,285 | DT19 | 2.08E-04 | 2.65 |
| SL.DT | *AX-108747720* | 2A | 58,539,285 | DT20 | 1.17E-05 | 3.78 |
| SL.DT | *AX-108747720* | 2A | 58,539,285 | DAVE | 6.08E-06 | 4.01 |
| SL.DT | *AX-108741112* | 2A | 63,924,247 | DAVE | 8.85E-04 | 2.14 |
| SL.DT | *AX-95209305* | 2A | 92,314,368 | DT19 | 7.97E-04 | 2.16 |
| SL.DT | *AX-95209305* | 2A | 92,314,368 | DAVE | 7.28E-04 | 2.21 |
| SL.DT | *AX-108880717* | 2A | 94,337,951 | DT19 | 5.85E-04 | 2.27 |
| SL.DT | *AX-108880717* | 2A | 94,337,951 | DAVE | 5.17E-04 | 2.34 |
| SL.DT | *AX-109852228* | 2A | 709,820,814 | DT20 | 4.03E-04 | 2.45 |
| SL.DT | *AX-110452729* | 2B | 12,195,773 | DT19 | 7.91E-04 | 2.16 |
| SL.DT | *AX-108906314* | 2B | 550,396,678 | DT19 | 3.72E-04 | 2.44 |
| SL.DT | *AX-94509892* | 2B | 777,301,801 | DT19 | 7.44E-04 | 2.19 |
| SL.DT | *AX-108988107* | 2D | 19,624,470 | DT19 | 7.51E-05 | 3.03 |
| SL.DT | *AX-110647062* | 2D | 23,025,488 | DT19 | 1.45E-08 | 6.31 |
| SL.DT | *AX-110647062* | 2D | 23,025,488 | DT20 | 5.52E-04 | 2.33 |
| SL.DT | *AX-110647062* | 2D | 23,025,488 | DAVE | 4.59E-08 | 5.91 |
| SL.DT | *AX-109973606* | 2D | 24,879,710 | DT19 | 1.45E-04 | 2.78 |
| SL.DT | *AX-111708993* | 3A | 272,796,105 | DT19 | 5.18E-04 | 2.32 |
| SL.DT | *AX-110667599* | 3A | 580,598,934 | DT19 | 8.91E-04 | 2.12 |
| SL.DT | *AX-111078409* | 3A | 583,347,793 | DT19 | 7.69E-04 | 2.17 |
| SL.DT | *AX-111528962* | 3A | 633,291,342 | DAVE | 3.05E-04 | 2.53 |
| SL.DT | *AX-109984137* | 3A | 633,772,383 | DAVE | 4.23E-04 | 2.41 |
| SL.DT | *AX-108897269* | 3A | 634,086,482 | DAVE | 5.04E-04 | 2.35 |
| SL.DT | *AX-89381998* | 3A | 637,901,078 | DT20 | 5.63E-04 | 2.32 |
| SL.DT | *AX-89381998* | 3A | 637,901,078 | DAVE | 1.39E-04 | 2.82 |
| SL.DT | *AX-110518818* | 3A | 649,438,322 | DT19 | 2.00E-04 | 2.66 |
| SL.DT | *AX-110496623* | 3A | 697,019,421 | DT19 | 7.58E-04 | 2.18 |
| SL.DT | *AX-111092736* | 3B | 457,755,811 | DT19 | 5.74E-04 | 2.28 |
| SL.DT | *AX-94595457* | 3B | 552,928,260 | DT20 | 7.08E-04 | 2.24 |
| SL.DT | *AX-110558618* | 3B | 765,318,016 | DT19 | 9.65E-04 | 2.09 |
| SL.DT | *AX-111087147* | 3B | 765,322,402 | DT19 | 2.65E-04 | 2.56 |
| SL.DT | *AX-109287041* | 3B | 766,022,155 | DT19 | 9.41E-04 | 2.10 |
| SL.DT | *AX-110122585* | 3B | 787,966,608 | DAVE | 9.99E-04 | 2.10 |
| SL.DT | *AX-110237963* | 3D | 102,119,028 | DT19 | 9.45E-04 | 2.10 |
| SL.DT | *AX-109284474* | 3D | 138,075,912 | DT19 | 7.03E-04 | 2.21 |
| SL.DT | *AX-108859112* | 3D | 139,250,658 | DT19 | 2.65E-04 | 2.56 |
| SL.DT | *AX-109662980* | 3D | 139,872,304 | DT19 | 8.79E-04 | 2.13 |
| SL.DT | *AX-110363017* | 3D | 165,411,038 | DT19 | 4.73E-04 | 2.35 |
| SL.DT | *AX-109383254* | 3D | 165,740,593 | DT19 | 5.25E-04 | 2.31 |
| SL.DT | *AX-108954855* | 3D | 168,170,961 | DT19 | 6.16E-04 | 2.25 |
| SL.DT | *AX-108874896* | 3D | 168,363,074 | DT19 | 5.26E-04 | 2.31 |
| SL.DT | *AX-108898785* | 3D | 170,935,503 | DT19 | 4.49E-04 | 2.37 |
| SL.DT | *AX-110826955* | 3D | 173,719,518 | DT19 | 4.77E-04 | 2.35 |
| SL.DT | *AX-109743167* | 3D | 178,403,033 | DT19 | 2.61E-04 | 2.57 |
| SL.DT | *AX-111362517* | 3D | 179,182,860 | DT19 | 5.77E-04 | 2.28 |
| SL.DT | *AX-111054517* | 3D | 180,840,097 | DT19 | 7.71E-04 | 2.17 |
| SL.DT | *AX-111099273* | 3D | 182,645,267 | DT19 | 6.19E-04 | 2.25 |
| SL.DT | *AX-108895977* | 3D | 184,119,274 | DT19 | 4.27E-04 | 2.39 |
| SL.DT | *AX-111675563* | 3D | 184,510,481 | DT19 | 4.53E-04 | 2.37 |
| SL.DT | *AX-108895854* | 3D | 184,816,308 | DT19 | 6.65E-04 | 2.23 |
| SL.DT | *AX-111131164* | 3D | 195,149,719 | DT19 | 5.30E-04 | 2.31 |
| SL.DT | *AX-111506806* | 3D | 198,833,762 | DT19 | 4.96E-04 | 2.33 |
| SL.DT | *AX-110434962* | 3D | 199,370,252 | DT19 | 8.10E-04 | 2.16 |
| SL.DT | *AX-109976094* | 3D | 204,178,154 | DT19 | 6.05E-04 | 2.26 |
| SL.DT | *AX-111408899* | 3D | 206,984,620 | DT19 | 9.95E-04 | 2.08 |
| SL.DT | *AX-109517575* | 3D | 208,559,092 | DT19 | 5.14E-04 | 2.32 |
| SL.DT | *AX-108886572* | 3D | 212,145,323 | DT19 | 3.05E-04 | 2.51 |
| SL.DT | *AX-111504450* | 3D | 212,626,573 | DT19 | 5.14E-04 | 2.32 |
| SL.DT | *AX-111322575* | 3D | 213,342,483 | DT19 | 5.03E-04 | 2.33 |
| SL.DT | *AX-109477772* | 3D | 220,375,362 | DT19 | 7.92E-04 | 2.16 |
| SL.DT | *AX-110269943* | 3D | 222,364,369 | DT19 | 4.70E-04 | 2.35 |
| SL.DT | *AX-110520367* | 3D | 223,988,713 | DT19 | 3.51E-04 | 2.46 |
| SL.DT | *AX-110942803* | 3D | 228,487,539 | DT19 | 4.70E-04 | 2.35 |
| SL.DT | *AX-108756717* | 3D | 231,987,021 | DT19 | 4.03E-04 | 2.41 |
| SL.DT | *AX-109889579* | 3D | 234,508,367 | DT19 | 3.69E-04 | 2.44 |
| SL.DT | *AX-109429611* | 3D | 240,786,059 | DT19 | 4.22E-04 | 2.39 |
| SL.DT | *AX-111564705* | 3D | 241,726,550 | DT19 | 3.48E-04 | 2.46 |
| SL.DT | *AX-110944192* | 3D | 244,908,012 | DT19 | 4.60E-04 | 2.36 |
| SL.DT | *AX-109394931* | 3D | 246,015,647 | DT19 | 4.70E-04 | 2.35 |
| SL.DT | *AX-110015572* | 3D | 249,305,505 | DT19 | 5.57E-04 | 2.29 |
| SL.DT | *AX-110813891* | 3D | 249,519,280 | DT19 | 3.87E-04 | 2.42 |
| SL.DT | *AX-110890657* | 3D | 257,547,008 | DT19 | 4.87E-04 | 2.34 |
| SL.DT | *AX-111654550* | 3D | 257,929,333 | DT19 | 4.70E-04 | 2.35 |
| SL.DT | *AX-109471652* | 3D | 268,152,476 | DT19 | 4.15E-04 | 2.40 |
| SL.DT | *AX-108877790* | 3D | 268,234,466 | DT19 | 8.01E-04 | 2.16 |
| SL.DT | *AX-111006048* | 3D | 270,143,417 | DT19 | 3.98E-04 | 2.41 |
| SL.DT | *AX-109399234* | 3D | 272,303,735 | DT19 | 4.00E-04 | 2.41 |
| SL.DT | *AX-110913237* | 3D | 273,001,033 | DT19 | 5.91E-04 | 2.27 |
| SL.DT | *AX-111147649* | 3D | 274,822,017 | DT19 | 4.70E-04 | 2.35 |
| SL.DT | *AX-111689571* | 3D | 274,984,684 | DT19 | 4.18E-04 | 2.40 |
| SL.DT | *AX-109505110* | 3D | 278,876,619 | DT19 | 5.22E-04 | 2.31 |
| SL.DT | *AX-110915537* | 3D | 281,172,931 | DT19 | 4.64E-04 | 2.36 |
| SL.DT | *AX-110282059* | 3D | 285,279,239 | DT19 | 4.62E-04 | 2.36 |
| SL.DT | *AX-111504843* | 3D | 288,669,293 | DT19 | 5.74E-04 | 2.28 |
| SL.DT | *AX-109783478* | 3D | 289,604,746 | DT19 | 4.70E-04 | 2.35 |
| SL.DT | *AX-109920728* | 3D | 290,681,086 | DT19 | 7.16E-04 | 2.20 |
| SL.DT | *AX-109735361* | 3D | 291,979,439 | DT19 | 3.60E-04 | 2.45 |
| SL.DT | *AX-110381932* | 3D | 294,604,232 | DT19 | 5.32E-04 | 2.31 |
| SL.DT | *AX-111502769* | 3D | 295,145,732 | DT19 | 5.74E-04 | 2.28 |
| SL.DT | *AX-109954183* | 3D | 297,247,421 | DT19 | 4.96E-04 | 2.33 |
| SL.DT | *AX-110958097* | 3D | 310,718,976 | DT19 | 5.18E-04 | 2.32 |
| SL.DT | *AX-110288100* | 3D | 311,850,375 | DT19 | 5.18E-04 | 2.32 |
| SL.DT | *AX-110029250* | 3D | 312,753,243 | DT19 | 8.65E-04 | 2.13 |
| SL.DT | *AX-109355391* | 3D | 313,055,523 | DT19 | 5.29E-04 | 2.31 |
| SL.DT | *AX-108958991* | 3D | 313,586,377 | DT19 | 5.06E-04 | 2.33 |
| SL.DT | *AX-109744083* | 3D | 317,228,145 | DT19 | 4.93E-04 | 2.34 |
| SL.DT | *AX-109914330* | 3D | 320,537,077 | DT19 | 3.51E-04 | 2.46 |
| SL.DT | *AX-111027124* | 3D | 400,781,396 | DT19 | 1.79E-04 | 2.70 |
| SL.DT | *AX-111027124* | 3D | 400,781,396 | DT20 | 7.17E-04 | 2.23 |
| SL.DT | *AX-111027124* | 3D | 400,781,396 | DAVE | 4.67E-04 | 2.38 |
| SL.DT | *AX-109366390* | 3D | 605,325,583 | DT19 | 8.66E-05 | 2.97 |
| SL.DT | *AX-111068370* | 3D | 606,079,108 | DT19 | 6.69E-05 | 3.07 |
| SL.DT | *AX-111481627* | 3D | 606,612,639 | DT19 | 1.49E-07 | 5.39 |
| SL.DT | *AX-111481627* | 3D | 606,612,639 | DAVE | 3.29E-05 | 3.37 |
| SL.DT | *AX-110975570* | 4A | 613,734,621 | DT19 | 5.73E-05 | 3.13 |
| SL.DT | *AX-110971025* | 4A | 641,592,792 | DT19 | 2.22E-04 | 2.63 |
| SL.DT | *AX-108949287* | 4A | 686,173,880 | DT19 | 6.22E-04 | 2.25 |
| SL.DT | *AX-110920884* | 4A | 686,381,407 | DT19 | 4.98E-04 | 2.33 |
| SL.DT | *AX-110920884* | 4A | 686,381,407 | DAVE | 8.85E-04 | 2.14 |
| SL.DT | *AX-109302588* | 4A | 689,834,479 | DT21 | 9.10E-04 | 2.23 |
| SL.DT | *AX-95630628* | 4B | 10,437,508 | DAVE | 3.95E-04 | 2.44 |
| SL.DT | *AX-109732646* | 4D | 96,147,593 | DT19 | 8.50E-04 | 2.14 |
| SL.DT | *AX-108726608* | 4D | 98,066,048 | DT19 | 7.61E-04 | 2.18 |
| SL.DT | *AX-108726608* | 4D | 98,066,048 | DAVE | 3.41E-04 | 2.49 |
| SL.DT | *AX-94940465* | 4D | 489,187,576 | DT19 | 8.81E-04 | 2.13 |
| SL.DT | *AX-94940465* | 4D | 489,187,576 | DAVE | 6.78E-04 | 2.24 |
| SL.DT | *AX-111509179* | 5A | 31,163,746 | DT19 | 6.06E-04 | 2.26 |
| SL.DT | *AX-110537939* | 5A | 37,389,245 | DT19 | 5.20E-04 | 2.32 |
| SL.DT | *AX-111530336* | 5A | 547,622,123 | DT20 | 9.20E-04 | 2.14 |
| SL.DT | *AX-109483736* | 5A | 603,380,801 | DT20 | 2.59E-04 | 2.61 |
| SL.DT | *AX-110396006* | 5B | 28,213,204 | DT21 | 2.74E-04 | 2.69 |
| SL.DT | *AX-110396006* | 5B | 28,213,204 | DAVE | 4.58E-04 | 2.38 |
| SL.DT | *AX-110538245* | 5B | 169,746,684 | DT20 | 4.54E-04 | 2.40 |
| SL.DT | *AX-109969417* | 5B | 171,215,533 | DT20 | 4.79E-04 | 2.38 |
| SL.DT | *AX-110574387* | 5B | 173,399,995 | DT20 | 2.99E-04 | 2.56 |
| SL.DT | *AX-111286497* | 5B | 178,095,746 | DT20 | 7.89E-04 | 2.20 |
| SL.DT | *AX-110195720* | 5B | 179,792,373 | DT20 | 6.38E-04 | 2.28 |
| SL.DT | *AX-109932243* | 5B | 182,802,694 | DT20 | 4.72E-04 | 2.39 |
| SL.DT | *AX-109390686* | 5B | 189,196,757 | DT20 | 1.95E-04 | 2.72 |
| SL.DT | *AX-95629151* | 5B | 206,798,948 | DT20 | 2.20E-04 | 2.67 |
| SL.DT | *AX-110609591* | 5B | 206,888,469 | DT20 | 3.91E-04 | 2.46 |
| SL.DT | *AX-111551138* | 5B | 208,118,717 | DT20 | 3.38E-04 | 2.51 |
| SL.DT | *AX-108739557* | 5B | 214,182,236 | DT20 | 2.49E-04 | 2.63 |
| SL.DT | *AX-108937379* | 5B | 216,860,872 | DT20 | 3.11E-04 | 2.54 |
| SL.DT | *AX-111013279* | 5B | 219,279,347 | DT20 | 2.99E-04 | 2.56 |
| SL.DT | *AX-109293799* | 5B | 219,989,467 | DT20 | 2.24E-04 | 2.66 |
| SL.DT | *AX-111517432* | 5B | 220,997,285 | DT20 | 3.19E-04 | 2.53 |
| SL.DT | *AX-111073870* | 5B | 222,111,171 | DT20 | 2.74E-04 | 2.59 |
| SL.DT | *AX-111680963* | 5B | 222,694,338 | DT20 | 4.32E-04 | 2.42 |
| SL.DT | *AX-110978478* | 5B | 223,092,073 | DT20 | 9.76E-04 | 2.12 |
| SL.DT | *AX-111575871* | 5B | 223,627,702 | DT20 | 6.37E-04 | 2.28 |
| SL.DT | *AX-108856729* | 5B | 224,625,276 | DT20 | 4.00E-04 | 2.45 |
| SL.DT | *AX-110362762* | 5B | 225,108,504 | DT20 | 4.43E-04 | 2.41 |
| SL.DT | *AX-108754217* | 5B | 225,706,839 | DT20 | 5.41E-04 | 2.34 |
| SL.DT | *AX-111619112* | 5B | 226,052,844 | DT20 | 4.68E-04 | 2.39 |
| SL.DT | *AX-110735496* | 5B | 226,530,925 | DT20 | 6.52E-04 | 2.27 |
| SL.DT | *AX-108890871* | 5B | 227,344,532 | DT20 | 4.26E-04 | 2.43 |
| SL.DT | *AX-108939406* | 5B | 229,248,808 | DT20 | 6.83E-04 | 2.25 |
| SL.DT | *AX-109101238* | 5B | 229,964,241 | DT20 | 4.48E-04 | 2.41 |
| SL.DT | *AX-111622763* | 5B | 231,352,724 | DT20 | 8.15E-04 | 2.19 |
| SL.DT | *AX-110494074* | 5B | 231,945,840 | DT20 | 6.72E-04 | 2.26 |
| SL.DT | *AX-111533693* | 5B | 233,924,727 | DT20 | 4.98E-04 | 2.37 |
| SL.DT | *AX-111454697* | 5B | 234,774,340 | DT20 | 5.27E-04 | 2.35 |
| SL.DT | *AX-110514822* | 5B | 235,177,818 | DT20 | 6.15E-04 | 2.29 |
| SL.DT | *AX-110688632* | 5B | 235,264,733 | DT20 | 5.84E-04 | 2.31 |
| SL.DT | *AX-108728380* | 5B | 236,197,278 | DT20 | 5.35E-04 | 2.34 |
| SL.DT | *AX-110554359* | 5B | 237,169,066 | DT20 | 4.48E-04 | 2.41 |
| SL.DT | *AX-108851808* | 5B | 238,055,872 | DT20 | 7.36E-04 | 2.22 |
| SL.DT | *AX-108955580* | 5B | 238,709,388 | DT20 | 7.03E-04 | 2.24 |
| SL.DT | *AX-111667993* | 5B | 240,977,879 | DT20 | 4.45E-04 | 2.41 |
| SL.DT | *AX-110376465* | 5B | 241,913,753 | DT20 | 5.72E-04 | 2.32 |
| SL.DT | *AX-111501562* | 5B | 242,356,046 | DT20 | 8.83E-04 | 2.16 |
| SL.DT | *AX-109321092* | 5B | 243,404,910 | DT20 | 4.67E-04 | 2.39 |
| SL.DT | *AX-110563039* | 5B | 269,294,177 | DT20 | 4.37E-04 | 2.42 |
| SL.DT | *AX-108770298* | 5B | 270,679,020 | DT20 | 5.03E-04 | 2.36 |
| SL.DT | *AX-110516200* | 5B | 277,898,057 | DT20 | 1.19E-04 | 2.90 |
| SL.DT | *AX-94496563* | 5B | 279,097,462 | DT20 | 6.34E-04 | 2.28 |
| SL.DT | *AX-86173022* | 5B | 279,097,660 | DT20 | 7.07E-04 | 2.24 |
| SL.DT | *AX-110467281* | 5B | 279,597,853 | DT20 | 9.29E-04 | 2.14 |
| SL.DT | *AX-109516452* | 5B | 280,943,711 | DT20 | 8.14E-04 | 2.19 |
| SL.DT | *AX-111505214* | 5B | 287,797,121 | DT20 | 8.14E-04 | 2.19 |
| SL.DT | *AX-94598757* | 5B | 316,562,253 | DT20 | 3.79E-05 | 3.33 |
| SL.DT | *AX-108735405* | 5B | 317,779,854 | DT20 | 3.97E-05 | 3.31 |
| SL.DT | *AX-110586527* | 5B | 317,993,039 | DT20 | 4.90E-05 | 3.24 |
| SL.DT | *AX-108868375* | 5B | 319,333,756 | DT20 | 2.36E-05 | 3.51 |
| SL.DT | *AX-110509091* | 5B | 321,408,342 | DT20 | 6.29E-05 | 3.14 |
| SL.DT | *AX-111759689* | 5B | 340,640,003 | DT20 | 8.72E-04 | 2.16 |
| SL.DT | *AX-109441188* | 5B | 387,765,973 | DT20 | 3.24E-04 | 2.53 |
| SL.DT | *AX-111274399* | 5B | 388,846,440 | DT20 | 2.76E-04 | 2.59 |
| SL.DT | *AX-109418455* | 5B | 390,400,782 | DT20 | 2.86E-04 | 2.57 |
| SL.DT | *AX-111054273* | 5B | 391,050,631 | DT20 | 2.36E-04 | 2.65 |
| SL.DT | *AX-108748929* | 5B | 391,446,098 | DT20 | 1.25E-04 | 2.88 |
| SL.DT | *AX-110506922* | 5B | 392,898,707 | DT20 | 2.01E-04 | 2.71 |
| SL.DT | *AX-108775541* | 5B | 394,025,627 | DT20 | 2.26E-04 | 2.66 |
| SL.DT | *AX-110968967* | 5B | 394,537,477 | DT20 | 2.14E-04 | 2.68 |
| SL.DT | *AX-109928742* | 5B | 394,970,067 | DT20 | 2.46E-04 | 2.63 |
| SL.DT | *AX-109095411* | 5B | 397,234,517 | DT20 | 4.40E-04 | 2.41 |
| SL.DT | *AX-109837402* | 5B | 400,770,851 | DT20 | 4.75E-04 | 2.39 |
| SL.DT | *AX-111039513* | 5B | 402,128,748 | DT20 | 6.64E-04 | 2.26 |
| SL.DT | *AX-109348429* | 5B | 404,478,483 | DT20 | 1.76E-04 | 2.75 |
| SL.DT | *AX-109825450* | 5B | 404,992,049 | DT20 | 1.36E-04 | 2.85 |
| SL.DT | *AX-111697598* | 5B | 405,520,813 | DT20 | 9.23E-05 | 3.00 |
| SL.DT | *AX-110006774* | 5B | 407,416,312 | DT20 | 1.78E-05 | 3.62 |
| SL.DT | *AX-110006774* | 5B | 407,416,312 | DAVE | 3.00E-04 | 2.54 |
| SL.DT | *AX-86184335* | 5B | 408,230,235 | DT20 | 5.74E-04 | 2.32 |
| SL.DT | *AX-86184335* | 5B | 408,230,235 | DAVE | 7.51E-04 | 2.20 |
| SL.DT | *AX-108863692* | 5B | 410,266,474 | DT20 | 3.55E-05 | 3.36 |
| SL.DT | *AX-108863692* | 5B | 410,266,474 | DAVE | 3.81E-04 | 2.45 |
| SL.DT | *AX-111013696* | 5B | 410,725,022 | DT20 | 4.73E-05 | 3.25 |
| SL.DT | *AX-111013696* | 5B | 410,725,022 | DAVE | 5.44E-04 | 2.32 |
| SL.DT | *AX-109496576* | 5B | 411,742,999 | DT20 | 2.83E-05 | 3.44 |
| SL.DT | *AX-109496576* | 5B | 411,742,999 | DAVE | 4.62E-04 | 2.38 |
| SL.DT | *AX-110934820* | 5B | 412,682,516 | DT20 | 2.34E-04 | 2.65 |
| SL.DT | *AX-110934820* | 5B | 412,682,516 | DAVE | 1.52E-04 | 2.79 |
| SL.DT | *AX-108885876* | 5B | 414,442,926 | DT20 | 5.30E-04 | 2.35 |
| SL.DT | *AX-108885876* | 5B | 414,442,926 | DAVE | 3.22E-04 | 2.51 |
| SL.DT | *AX-109964143* | 5B | 415,526,589 | DT20 | 1.41E-04 | 2.84 |
| SL.DT | *AX-109964143* | 5B | 415,526,589 | DAVE | 1.84E-04 | 2.72 |
| SL.DT | *AX-110703046* | 5B | 415,738,957 | DT20 | 2.69E-04 | 2.60 |
| SL.DT | *AX-110703046* | 5B | 415,738,957 | DAVE | 1.12E-04 | 2.90 |
| SL.DT | *AX-109936301* | 5B | 416,436,584 | DT20 | 2.84E-04 | 2.58 |
| SL.DT | *AX-109936301* | 5B | 416,436,584 | DAVE | 2.03E-04 | 2.68 |
| SL.DT | *AX-110542749* | 5B | 416,971,022 | DT20 | 3.44E-04 | 2.50 |
| SL.DT | *AX-110542749* | 5B | 416,971,022 | DAVE | 2.19E-04 | 2.66 |
| SL.DT | *AX-109284684* | 5B | 418,309,355 | DT20 | 1.69E-05 | 3.64 |
| SL.DT | *AX-109284684* | 5B | 418,309,355 | DAVE | 2.15E-04 | 2.66 |
| SL.DT | *AX-108958865* | 5B | 418,509,439 | DT20 | 1.70E-04 | 2.77 |
| SL.DT | *AX-108958865* | 5B | 418,509,439 | DAVE | 4.63E-04 | 2.38 |
| SL.DT | *AX-110576628* | 5B | 420,011,554 | DT20 | 1.33E-04 | 2.86 |
| SL.DT | *AX-110576628* | 5B | 420,011,554 | DAVE | 3.23E-04 | 2.51 |
| SL.DT | *AX-108827033* | 5B | 422,218,746 | DT20 | 8.09E-05 | 3.05 |
| SL.DT | *AX-111672485* | 5B | 422,501,097 | DT20 | 7.20E-05 | 3.09 |
| SL.DT | *AX-109581281* | 5B | 437,250,895 | DT20 | 3.03E-04 | 2.55 |
| SL.DT | *AX-111124661* | 5B | 437,564,786 | DT19 | 9.26E-04 | 2.11 |
| SL.DT | *AX-111124661* | 5B | 437,564,786 | DT20 | 2.44E-04 | 2.63 |
| SL.DT | *AX-109847779* | 5B | 438,162,503 | DAVE | 5.57E-04 | 2.31 |
| SL.DT | *AX-94524014* | 5B | 440,179,428 | DT20 | 3.60E-04 | 2.49 |
| SL.DT | *AX-110608136* | 5B | 454,650,013 | DT19 | 3.20E-04 | 2.49 |
| SL.DT | *AX-111470663* | 5B | 455,533,462 | DT19 | 4.45E-05 | 3.22 |
| SL.DT | *AX-86185288* | 5B | 455,738,909 | DT19 | 1.14E-04 | 2.87 |
| SL.DT | *AX-108961268* | 5B | 496,987,522 | DT20 | 7.95E-04 | 2.20 |
| SL.DT | *AX-109500107* | 5B | 497,375,926 | DT20 | 8.61E-04 | 2.17 |
| SL.DT | *AX-111574616* | 5B | 499,375,416 | DT20 | 9.86E-04 | 2.12 |
| SL.DT | *AX-95087248* | 5B | 499,384,472 | DT20 | 9.83E-04 | 2.12 |
| SL.DT | *AX-109549994* | 5B | 501,954,912 | DT20 | 9.04E-04 | 2.15 |
| SL.DT | *AX-110428884* | 5B | 503,225,188 | DT20 | 9.86E-04 | 2.12 |
| SL.DT | *AX-111458448* | 5B | 508,681,557 | DT20 | 6.88E-04 | 2.25 |
| SL.DT | *AX-111548112* | 5B | 508,796,063 | DT20 | 6.29E-04 | 2.28 |
| SL.DT | *AX-109411601* | 5B | 511,852,027 | DT20 | 7.97E-04 | 2.20 |
| SL.DT | *AX-109556435* | 5B | 512,246,353 | DT20 | 8.17E-04 | 2.19 |
| SL.DT | *AX-110958685* | 5B | 512,839,278 | DT20 | 8.02E-04 | 2.19 |
| SL.DT | *AX-108766482* | 5B | 513,590,972 | DT20 | 7.60E-04 | 2.21 |
| SL.DT | *AX-111709936* | 5B | 515,183,465 | DT20 | 5.21E-04 | 2.35 |
| SL.DT | *AX-108943772* | 5B | 516,366,829 | DT20 | 8.38E-04 | 2.18 |
| SL.DT | *AX-108745488* | 5B | 516,561,605 | DT20 | 8.80E-04 | 2.16 |
| SL.DT | *AX-109286283* | 5B | 518,078,921 | DT20 | 4.42E-04 | 2.41 |
| SL.DT | *AX-109820610* | 5B | 518,570,389 | DT20 | 8.29E-04 | 2.18 |
| SL.DT | *AX-110912471* | 5B | 520,555,009 | DT20 | 7.44E-04 | 2.22 |
| SL.DT | *AX-110439426* | 5B | 521,014,241 | DT20 | 6.64E-04 | 2.26 |
| SL.DT | *AX-111739361* | 5D | 244,617,835 | DT20 | 5.33E-04 | 2.34 |
| SL.DT | *AX-108848982* | 5D | 332,040,170 | DT20 | 2.99E-04 | 2.56 |
| SL.DT | *AX-111730067* | 5D | 369,299,875 | DAVE | 3.67E-04 | 2.47 |
| SL.DT | *AX-111451774* | 5D | 414,097,105 | DT20 | 8.93E-04 | 2.15 |
| SL.DT | *AX-108865212* | 5D | 415,746,981 | DT20 | 8.73E-04 | 2.16 |
| SL.DT | *AX-110161029* | 5D | 417,855,064 | DT20 | 9.94E-04 | 2.11 |
| SL.DT | *AX-109527199* | 5D | 426,296,784 | DT20 | 4.89E-04 | 2.38 |
| SL.DT | *AX-94504234* | 5D | 428,496,395 | DT20 | 8.47E-04 | 2.17 |
| SL.DT | *AX-111035187* | 5D | 429,360,696 | DT20 | 1.99E-04 | 2.71 |
| SL.DT | *AX-111512411* | 5D | 432,132,545 | DT20 | 7.89E-04 | 2.20 |
| SL.DT | *AX-110616233* | 5D | 434,403,364 | DT20 | 9.36E-04 | 2.14 |
| SL.DT | *AX-109207441* | 5D | 534,992,827 | DAVE | 3.26E-04 | 2.51 |
| SL.DT | *AX-109376346* | 6A | 61,748,025 | DAVE | 7.73E-04 | 2.19 |
| SL.DT | *AX-110385344* | 6A | 107,691,933 | DAVE | 4.51E-04 | 2.39 |
| SL.DT | *AX-109830450* | 6A | 109,699,408 | DAVE | 8.15E-04 | 2.17 |
| SL.DT | *AX-94510892* | 6A | 112,585,030 | DAVE | 4.23E-04 | 2.41 |
| SL.DT | *AX-109272810* | 6A | 520,671,232 | DAVE | 3.69E-04 | 2.46 |
| SL.DT | *AX-110087250* | 6A | 525,761,014 | DAVE | 2.11E-04 | 2.67 |
| SL.DT | *AX-109894066* | 6A | 525,763,348 | DAVE | 2.20E-04 | 2.65 |
| SL.DT | *AX-109527686* | 6A | 582,640,221 | DT19 | 6.15E-04 | 2.25 |
| SL.DT | *AX-109527686* | 6A | 582,640,221 | DAVE | 9.67E-05 | 2.96 |
| SL.DT | *AX-111077590* | 6A | 582,941,584 | DAVE | 2.86E-04 | 2.56 |
| SL.DT | *AX-109337721* | 6B | 528,705,208 | DT20 | 5.97E-04 | 2.30 |
| SL.DT | *AX-109337721* | 6B | 528,705,208 | DAVE | 8.17E-04 | 2.17 |
| SL.DT | *AX-110464411* | 6B | 529,217,159 | DT20 | 5.61E-04 | 2.32 |
| SL.DT | *AX-110464411* | 6B | 529,217,159 | DAVE | 9.59E-04 | 2.11 |
| SL.DT | *AX-111453972* | 6B | 529,697,367 | DT20 | 7.50E-04 | 2.22 |
| SL.DT | *AX-108871977* | 6D | 294,634,977 | DAVE | 6.66E-04 | 2.25 |
| SL.DT | *AX-111917346* | 6D | 301,654,754 | DAVE | 3.74E-04 | 2.46 |
| SL.DT | *AX-111619425* | 7A | 79,654,213 | DT21 | 2.45E-04 | 2.73 |
| SL.DT | *AX-111638232* | 7A | 80,313,737 | DT21 | 5.11E-04 | 2.45 |
| SL.DT | *AX-111587421* | 7A | 80,839,980 | DT21 | 1.22E-04 | 3.00 |
| SL.DT | *AX-109952999* | 7A | 81,303,838 | DT21 | 4.95E-04 | 2.46 |
| SL.DT | *AX-109848988* | 7A | 86,820,252 | DAVE | 6.78E-04 | 2.24 |
| SL.DT | *AX-111544318* | 7A | 638,519,956 | DT19 | 2.04E-04 | 2.66 |
| SL.DT | *AX-109394965* | 7A | 640,675,003 | DT19 | 2.74E-05 | 3.40 |
| SL.DT | *AX-109394965* | 7A | 640,675,003 | DAVE | 2.61E-04 | 2.59 |
| SL.DT | *AX-110398543* | 7A | 642,864,251 | DT19 | 1.03E-04 | 2.91 |
| SL.DT | *AX-94778315* | 7B | 587,914,953 | DT21 | 7.80E-04 | 2.29 |
| SL.DT | *AX-110057611* | 7B | 739,450,212 | DT19 | 3.50E-04 | 2.46 |
| SL.DT | *AX-108951559* | 7D | 181,350,701 | DT19 | 1.65E-04 | 2.74 |
| SL.DT | *AX-109541707* | 7D | 538,214,362 | DAVE | 6.74E-04 | 2.24 |
| SL.DT | *AX-110452592* | 7D | 637,094,588 | DT19 | 2.55E-04 | 2.58 |

^a^ Physical position of the SNP in the reference genome (IWGSC RefSeq v1.0); ^b^ Phenotypic variation explained by the identified SNP.

**Supplementary Table 4.** The significant SNPs identified by GWAS for stress tolerance index.

| Trait | SNP | Chr | Position (bp)^a^ | *P* value | PVE (%)^b^ |
| --- | --- | --- | --- | --- | --- |
| STIA | *AX-111503092* | 1B | 380,647,070 | 8.16E-04 | 2.08 |
| STIA | *AX-111169510* | 1B | 430,155,688 | 2.80E-04 | 2.45 |
| STIA | *AX-110365668* | 1B | 443,330,272 | 9.76E-04 | 2.02 |
| STIA | *AX-110916356* | 1B | 489,129,788 | 5.34E-04 | 2.23 |
| STIA | *AX-110670988* | 1B | 489,973,360 | 3.65E-04 | 2.36 |
| STIA | *AX-111780371* | 1B | 510,356,945 | 6.71E-04 | 2.15 |
| STIA | *AX-108920917* | 1B | 511,050,019 | 4.20E-04 | 2.31 |
| STIA | *AX-109457059* | 1B | 511,409,502 | 2.90E-04 | 2.44 |
| STIA | *AX-109966524* | 1B | 512,107,159 | 3.56E-04 | 2.37 |
| STIA | *AX-109271243* | 1D | 9,424,088 | 9.97E-04 | 2.01 |
| STIA | *AX-109290429* | 2A | 608,865,127 | 6.80E-04 | 2.14 |
| STIA | *AX-110425132* | 2A | 705,807,076 | 8.96E-04 | 2.05 |
| STIA | *AX-95219673* | 2B | 671,741,110 | 9.06E-04 | 2.04 |
| STIA | *AX-109352432* | 2B | 731,463,570 | 2.58E-04 | 2.48 |
| STIA | *AX-111534973* | 3B | 41,633,676 | 6.33E-04 | 2.17 |
| STIA | *AX-111103248* | 3D | 178,761,794 | 5.63E-04 | 2.21 |
| STIA | *AX-109903567* | 3D | 196,832,500 | 7.44E-04 | 2.11 |
| STIA | *AX-110432825* | 3D | 206,655,789 | 5.23E-04 | 2.23 |
| STIA | *AX-109341556* | 3D | 221,046,278 | 5.63E-04 | 2.21 |
| STIA | *AX-109626991* | 3D | 224,648,576 | 7.69E-04 | 2.10 |
| STIA | *AX-109392999* | 3D | 236,169,118 | 6.75E-04 | 2.14 |
| STIA | *AX-109407590* | 3D | 246,923,598 | 7.50E-04 | 2.11 |
| STIA | *AX-110830021* | 3D | 260,816,674 | 6.14E-04 | 2.18 |
| STIA | *AX-110931508* | 4A | 632,242,065 | 4.52E-04 | 2.28 |
| STIA | *AX-111040045* | 4A | 632,764,370 | 1.61E-04 | 2.65 |
| STIA | *AX-109375057* | 4A | 633,308,367 | 1.23E-05 | 3.58 |
| STIA | *AX-110468999* | 4A | 634,108,610 | 4.49E-05 | 3.11 |
| STIA | *AX-111140650* | 4A | 634,368,256 | 9.97E-05 | 2.82 |
| STIA | *AX-111453918* | 4A | 634,833,572 | 3.93E-05 | 3.15 |
| STIA | *AX-110440161* | 4A | 635,292,292 | 7.85E-05 | 2.91 |
| STIA | *AX-108950546* | 4A | 635,764,214 | 1.01E-05 | 3.65 |
| STIA | *AX-95160379* | 4B | 30,589,960 | 4.75E-06 | 3.92 |
| STIA | *AX-110021330* | 4B | 30,863,602 | 1.66E-05 | 3.47 |
| STIA | *AX-108784886* | 5A | 46,574,485 | 7.18E-04 | 2.12 |
| STIA | *AX-108813905* | 5A | 47,440,953 | 9.18E-04 | 2.04 |
| STIA | *AX-110482082* | 6A | 237,479,423 | 7.55E-04 | 2.11 |
| STIA | *AX-109093066* | 6A | 237,647,623 | 7.24E-04 | 2.12 |
| STIA | *AX-109061200* | 6A | 237,728,800 | 5.53E-04 | 2.21 |
| STIA | *AX-111243668* | 6A | 237,805,680 | 7.16E-04 | 2.12 |
| STIA | *AX-109077760* | 6A | 240,361,344 | 9.77E-04 | 2.02 |
| STIA | *AX-110593017* | 6A | 245,781,410 | 9.14E-04 | 2.04 |
| STIA | *AX-108975281* | 6A | 292,794,082 | 8.60E-04 | 2.06 |
| STIA | *AX-111266348* | 6A | 296,128,743 | 8.55E-04 | 2.06 |
| STIA | *AX-110151642* | 6A | 296,195,705 | 9.54E-04 | 2.02 |
| STIA | *AX-111635885* | 6A | 297,411,376 | 8.42E-04 | 2.07 |
| STIA | *AX-94952400* | 6A | 299,335,643 | 6.33E-04 | 2.17 |
| STIA | *AX-109918284* | 6A | 300,755,858 | 6.94E-04 | 2.13 |
| STIA | *AX-109903000* | 6A | 302,251,872 | 6.93E-04 | 2.14 |
| STIA | *AX-108975012* | 6A | 303,534,001 | 2.43E-04 | 2.50 |
| STIA | *AX-108991354* | 6A | 306,048,895 | 6.95E-04 | 2.13 |
| STIA | *AX-111059292* | 6A | 308,821,559 | 5.90E-04 | 2.19 |
| STIA | *AX-111806667* | 6A | 309,291,590 | 7.97E-04 | 2.09 |
| STIA | *AX-109070240* | 6A | 310,060,628 | 6.95E-04 | 2.13 |
| STIA | *AX-111097598* | 6A | 312,077,737 | 8.05E-04 | 2.08 |
| STIA | *AX-111267479* | 6A | 326,823,896 | 8.05E-04 | 2.08 |
| STIA | *AX-108937778* | 6A | 327,484,309 | 8.42E-04 | 2.07 |
| STIA | *AX-109334618* | 6A | 328,130,345 | 7.48E-05 | 2.92 |
| STIA | *AX-110474160* | 6A | 328,510,329 | 7.03E-04 | 2.13 |
| STIA | *AX-110911407* | 6A | 329,608,775 | 9.65E-04 | 2.02 |
| STIA | *AX-110916005* | 6A | 330,873,938 | 7.89E-04 | 2.09 |
| STIA | *AX-108800286* | 6A | 335,807,066 | 7.99E-04 | 2.09 |
| STIA | *AX-111278973* | 6A | 336,253,032 | 6.90E-04 | 2.14 |
| STIA | *AX-108869501* | 6A | 338,237,015 | 8.42E-04 | 2.07 |
| STIA | *AX-111273273* | 6A | 340,704,113 | 8.05E-04 | 2.08 |
| STIA | *AX-108817301* | 6A | 341,957,483 | 4.84E-04 | 2.26 |
| STIA | *AX-109448914* | 6A | 349,053,789 | 8.87E-04 | 2.05 |
| STIA | *AX-111835367* | 6A | 349,835,322 | 8.62E-04 | 2.06 |
| STIA | *AX-109043962* | 6A | 352,377,413 | 9.65E-04 | 2.02 |
| STIA | *AX-109080612* | 6A | 352,542,166 | 9.30E-04 | 2.03 |
| STIA | *AX-111695805* | 6A | 367,236,934 | 7.23E-04 | 2.12 |
| STIA | *AX-109084122* | 6A | 374,826,615 | 4.60E-04 | 2.28 |
| STIA | *AX-111466636* | 6A | 377,363,579 | 7.27E-04 | 2.12 |
| STIA | *AX-111801320* | 6A | 378,218,123 | 8.51E-04 | 2.06 |
| STIA | *AX-111072559* | 6A | 398,796,577 | 6.41E-04 | 2.16 |
| STIA | *AX-109063873* | 6A | 433,569,417 | 3.31E-04 | 2.39 |
| STIA | *AX-109353146* | 6A | 442,400,023 | 3.12E-04 | 2.41 |
| STIA | *AX-111189818* | 6B | 570,159,601 | 8.89E-04 | 2.05 |
| STIA | *AX-109447460* | 6D | 89,262,091 | 6.34E-04 | 2.17 |
| STIA | *AX-108920250* | 7D | 54,995,342 | 6.01E-04 | 2.18 |
| STIA | *AX-111898961* | 7D | 107,933,478 | 9.09E-04 | 2.04 |
| STIA | *AX-111798949* | 7D | 545,167,822 | 7.36E-04 | 2.11 |
| STIA | *AX-108758043* | 7D | 547,277,450 | 2.52E-04 | 2.49 |
| STIA | *AX-110949705* | 7D | 548,055,156 | 3.02E-04 | 2.43 |
| STIA | *AX-108770812* | 7D | 561,926,335 | 3.57E-05 | 3.19 |
| STI19 | *AX-111084696* | 1A | 16,422,919 | 5.23E-05 | 4.05 |
| STI19 | *AX-109290429* | 2A | 608,865,127 | 7.86E-04 | 2.77 |
| STI19 | *AX-109425314* | 2A | 693,336,312 | 2.74E-05 | 4.36 |
| STI19 | *AX-94547450* | 2A | 694,333,888 | 2.35E-04 | 3.34 |
| STI19 | *AX-95219673* | 2B | 671,741,110 | 5.98E-04 | 2.90 |
| STI19 | *AX-108887673* | 3A | 726,511,279 | 6.61E-04 | 2.85 |
| STI19 | *AX-109246690* | 3D | 372,330,014 | 6.72E-04 | 2.85 |
| STI19 | *AX-110171894* | 4A | 605,753,144 | 2.20E-04 | 3.37 |
| STI19 | *AX-109626103* | 4A | 605,764,861 | 3.90E-04 | 3.10 |
| STI19 | *AX-108777965* | 4A | 605,767,873 | 2.56E-04 | 3.30 |
| STI19 | *AX-110961937* | 4A | 605,785,021 | 2.13E-04 | 3.38 |
| STI19 | *AX-109320255* | 4B | 172,526,927 | 7.68E-04 | 2.78 |
| STI19 | *AX-111150060* | 4B | 660,589,159 | 5.86E-04 | 2.91 |
| STI19 | *AX-108830474* | 4D | 413,032,782 | 6.21E-04 | 2.88 |
| STI19 | *AX-109422629* | 5A | 513,845,754 | 4.21E-04 | 3.06 |
| STI19 | *AX-109369427* | 5A | 546,521,932 | 1.85E-04 | 3.45 |
| STI19 | *AX-110976602* | 5A | 585,742,226 | 4.03E-04 | 3.08 |
| STI19 | *AX-111516865* | 5B | 693,823,642 | 2.83E-05 | 4.35 |
| STI19 | *AX-108757760* | 5B | 701,544,672 | 1.07E-04 | 3.71 |
| STI19 | *AX-108733256* | 5B | 702,228,057 | 2.12E-04 | 3.39 |
| STI19 | *AX-110946146* | 5B | 703,062,745 | 5.36E-04 | 2.95 |
| STI19 | *AX-110758473* | 5D | 292,945,585 | 4.02E-04 | 3.09 |
| STI19 | *AX-108782785* | 5D | 298,445,559 | 2.07E-04 | 3.40 |
| STI19 | *AX-111616614* | 5D | 393,799,703 | 8.38E-04 | 2.74 |
| STI19 | *AX-110482082* | 6A | 237,479,423 | 2.68E-04 | 3.27 |
| STI19 | *AX-109093066* | 6A | 237,647,623 | 2.76E-04 | 3.26 |
| STI19 | *AX-109061200* | 6A | 237,728,800 | 2.26E-04 | 3.36 |
| STI19 | *AX-111243668* | 6A | 237,805,680 | 2.59E-04 | 3.29 |
| STI19 | *AX-109077760* | 6A | 240,361,344 | 4.12E-04 | 3.07 |
| STI19 | *AX-110593017* | 6A | 245,781,410 | 2.83E-04 | 3.25 |
| STI19 | *AX-111146080* | 6A | 259,315,472 | 5.97E-04 | 2.90 |
| STI19 | *AX-110596423* | 6A | 262,623,476 | 4.41E-04 | 3.04 |
| STI19 | *AX-109282602* | 6A | 277,282,442 | 3.20E-04 | 3.19 |
| STI19 | *AX-95630469* | 6A | 286,320,473 | 2.36E-04 | 3.33 |
| STI19 | *AX-108975281* | 6A | 292,794,082 | 4.21E-04 | 3.06 |
| STI19 | *AX-111493035* | 6A | 293,145,390 | 4.16E-04 | 3.07 |
| STI19 | *AX-111266348* | 6A | 296,128,743 | 3.51E-04 | 3.15 |
| STI19 | *AX-110151642* | 6A | 296,195,705 | 3.58E-04 | 3.14 |
| STI19 | *AX-111635885* | 6A | 297,411,376 | 3.75E-04 | 3.12 |
| STI19 | *AX-94952400* | 6A | 299,335,643 | 3.22E-04 | 3.19 |
| STI19 | *AX-109918284* | 6A | 300,755,858 | 3.79E-04 | 3.11 |
| STI19 | *AX-109903000* | 6A | 302,251,872 | 3.72E-04 | 3.12 |
| STI19 | *AX-108975012* | 6A | 303,534,001 | 3.95E-04 | 3.09 |
| STI19 | *AX-108991354* | 6A | 306,048,895 | 3.75E-04 | 3.12 |
| STI19 | *AX-110129241* | 6A | 306,943,674 | 6.30E-04 | 2.88 |
| STI19 | *AX-111059292* | 6A | 308,821,559 | 2.69E-04 | 3.27 |
| STI19 | *AX-111806667* | 6A | 309,291,590 | 4.17E-04 | 3.07 |
| STI19 | *AX-109070240* | 6A | 310,060,628 | 3.75E-04 | 3.12 |
| STI19 | *AX-111214686* | 6A | 310,507,515 | 4.54E-04 | 3.03 |
| STI19 | *AX-111097598* | 6A | 312,077,737 | 3.61E-04 | 3.14 |
| STI19 | *AX-109863766* | 6A | 316,056,716 | 6.10E-04 | 2.89 |
| STI19 | *AX-109967817* | 6A | 316,147,992 | 6.95E-04 | 2.83 |
| STI19 | *AX-111818250* | 6A | 316,545,919 | 4.42E-04 | 3.04 |
| STI19 | *AX-111478362* | 6A | 318,287,443 | 3.27E-04 | 3.18 |
| STI19 | *AX-110024798* | 6A | 324,812,584 | 2.36E-04 | 3.34 |
| STI19 | *AX-111267479* | 6A | 326,823,896 | 4.27E-04 | 3.06 |
| STI19 | *AX-108937778* | 6A | 327,484,309 | 3.75E-04 | 3.12 |
| STI19 | *AX-109334618* | 6A | 328,130,345 | 6.73E-04 | 2.85 |
| STI19 | *AX-110474160* | 6A | 328,510,329 | 4.37E-04 | 3.05 |
| STI19 | *AX-110911407* | 6A | 329,608,775 | 4.17E-04 | 3.07 |
| STI19 | *AX-110916005* | 6A | 330,873,938 | 3.58E-04 | 3.14 |
| STI19 | *AX-108911940* | 6A | 332,602,380 | 4.17E-04 | 3.07 |
| STI19 | *AX-108800286* | 6A | 335,807,066 | 6.96E-04 | 2.83 |
| STI19 | *AX-111278973* | 6A | 336,253,032 | 3.89E-04 | 3.10 |
| STI19 | *AX-108869501* | 6A | 338,237,015 | 3.75E-04 | 3.12 |
| STI19 | *AX-111273273* | 6A | 340,704,113 | 3.61E-04 | 3.14 |
| STI19 | *AX-108817301* | 6A | 341,957,483 | 3.09E-04 | 3.21 |
| STI19 | *AX-110428139* | 6A | 344,829,509 | 8.04E-04 | 2.76 |
| STI19 | *AX-109448914* | 6A | 349,053,789 | 3.75E-04 | 3.12 |
| STI19 | *AX-111835367* | 6A | 349,835,322 | 4.67E-04 | 3.02 |
| STI19 | *AX-109043962* | 6A | 352,377,413 | 4.17E-04 | 3.07 |
| STI19 | *AX-109080612* | 6A | 352,542,166 | 4.27E-04 | 3.06 |
| STI19 | *AX-111685841* | 6A | 358,591,651 | 4.83E-04 | 3.00 |
| STI19 | *AX-111695805* | 6A | 367,236,934 | 3.72E-04 | 3.12 |
| STI19 | *AX-109084122* | 6A | 374,826,615 | 3.67E-04 | 3.13 |
| STI19 | *AX-111466636* | 6A | 377,363,579 | 4.32E-04 | 3.05 |
| STI19 | *AX-111801320* | 6A | 378,218,123 | 4.31E-04 | 3.05 |
| STI19 | *AX-109063873* | 6A | 433,569,417 | 4.37E-04 | 3.05 |
| STI19 | *AX-109353146* | 6A | 442,400,023 | 3.61E-04 | 3.14 |
| STI19 | *AX-111652073* | 6B | 610,363,648 | 4.82E-04 | 3.00 |
| STI19 | *AX-108921085* | 6B | 610,981,799 | 2.48E-04 | 3.31 |
| STI19 | *AX-111566434* | 6B | 612,059,447 | 3.91E-04 | 3.10 |
| STI19 | *AX-111605648* | 6B | 612,849,771 | 7.94E-04 | 2.77 |
| STI19 | *AX-110500742* | 6B | 630,949,717 | 8.07E-04 | 2.76 |
| STI19 | *AX-109940210* | 7A | 644,858,583 | 4.85E-04 | 3.00 |
| STI19 | *AX-110925530* | 7A | 725,129,384 | 9.89E-04 | 2.67 |
| STI19 | *AX-110534723* | 7B | 521,592,642 | 7.31E-04 | 2.81 |
| STI19 | *AX-108934838* | 7B | 523,606,124 | 9.17E-04 | 2.70 |
| STI19 | *AX-110949705* | 7D | 548,055,156 | 7.94E-04 | 2.77 |
| STI19 | *AX-108770812* | 7D | 561,926,335 | 3.65E-04 | 3.13 |
| STI20 | *AX-109425297* | 1A | 523,030,643 | 8.08E-04 | 2.40 |
| STI20 | *AX-109937019* | 1A | 525,123,787 | 5.63E-04 | 2.54 |
| STI20 | *AX-110122042* | 1A | 528,633,639 | 6.88E-04 | 2.46 |
| STI20 | *AX-109308096* | 1B | 289,529,400 | 5.64E-04 | 2.54 |
| STI20 | *AX-108741139* | 1B | 290,203,821 | 6.93E-04 | 2.46 |
| STI20 | *AX-110419096* | 1B | 290,870,245 | 1.11E-04 | 3.21 |
| STI20 | *AX-109037969* | 1B | 292,197,988 | 6.31E-04 | 2.50 |
| STI20 | *AX-110552541* | 1B | 293,996,773 | 6.05E-04 | 2.51 |
| STI20 | *AX-109383638* | 1B | 295,437,309 | 4.51E-04 | 2.63 |
| STI20 | *AX-111217038* | 1B | 295,451,495 | 7.22E-04 | 2.44 |
| STI20 | *AX-109857682* | 1B | 296,491,871 | 5.65E-04 | 2.54 |
| STI20 | *AX-110468810* | 1B | 302,543,411 | 7.75E-04 | 2.41 |
| STI20 | *AX-108960559* | 1B | 303,863,408 | 5.97E-04 | 2.52 |
| STI20 | *AX-109356154* | 1B | 310,932,331 | 7.69E-04 | 2.42 |
| STI20 | *AX-110368320* | 1B | 311,736,328 | 9.34E-04 | 2.34 |
| STI20 | *AX-110601772* | 1B | 313,527,562 | 8.92E-04 | 2.36 |
| STI20 | *AX-95176576* | 1B | 314,569,349 | 8.95E-04 | 2.36 |
| STI20 | *AX-94715187* | 1B | 317,013,429 | 6.96E-04 | 2.46 |
| STI20 | *AX-110524472* | 1B | 340,381,039 | 4.57E-04 | 2.63 |
| STI20 | *AX-109050847* | 1B | 340,423,903 | 3.02E-04 | 2.80 |
| STI20 | *AX-108838990* | 1B | 342,122,438 | 3.07E-04 | 2.79 |
| STI20 | *AX-110924487* | 1B | 343,245,050 | 2.62E-04 | 2.85 |
| STI20 | *AX-111597240* | 1B | 343,953,543 | 1.98E-04 | 2.97 |
| STI20 | *AX-111610602* | 1B | 354,492,000 | 8.20E-04 | 2.39 |
| STI20 | *AX-108909153* | 1B | 356,984,891 | 7.99E-04 | 2.40 |
| STI20 | *AX-108995348* | 1B | 357,649,901 | 6.63E-04 | 2.48 |
| STI20 | *AX-111524617* | 1B | 364,328,609 | 3.82E-04 | 2.70 |
| STI20 | *AX-110531641* | 1B | 365,441,304 | 2.19E-04 | 2.93 |
| STI20 | *AX-111463788* | 1B | 365,926,056 | 2.34E-04 | 2.90 |
| STI20 | *AX-111482877* | 1B | 366,492,631 | 1.51E-04 | 3.08 |
| STI20 | *AX-109437757* | 1B | 366,917,565 | 1.68E-04 | 3.03 |
| STI20 | *AX-111118968* | 1B | 367,588,737 | 1.86E-04 | 2.99 |
| STI20 | *AX-111733823* | 1B | 367,838,063 | 1.15E-04 | 3.19 |
| STI20 | *AX-110597141* | 1B | 368,675,675 | 1.32E-04 | 3.13 |
| STI20 | *AX-110474956* | 1B | 369,345,717 | 1.06E-04 | 3.22 |
| STI20 | *AX-110623229* | 1B | 369,515,449 | 1.51E-04 | 3.08 |
| STI20 | *AX-109581046* | 1B | 370,197,585 | 1.68E-04 | 3.04 |
| STI20 | *AX-108845851* | 1B | 371,140,782 | 7.29E-05 | 3.38 |
| STI20 | *AX-110386580* | 1B | 372,157,887 | 2.05E-04 | 2.95 |
| STI20 | *AX-109387657* | 1B | 372,217,456 | 1.95E-04 | 2.97 |
| STI20 | *AX-110689665* | 1B | 372,748,785 | 2.92E-04 | 2.81 |
| STI20 | *AX-110740030* | 1B | 373,856,428 | 2.02E-04 | 2.96 |
| STI20 | *AX-111707424* | 1B | 374,316,467 | 1.51E-04 | 3.08 |
| STI20 | *AX-109932384* | 1B | 374,913,451 | 1.84E-04 | 3.00 |
| STI20 | *AX-109580210* | 1B | 375,303,546 | 1.16E-04 | 3.19 |
| STI20 | *AX-110369203* | 1B | 375,821,909 | 9.44E-05 | 3.27 |
| STI20 | *AX-111121879* | 1B | 376,791,311 | 1.41E-04 | 3.11 |
| STI20 | *AX-109544098* | 1B | 377,963,460 | 6.63E-05 | 3.42 |
| STI20 | *AX-109853345* | 1B | 378,461,864 | 2.12E-04 | 2.94 |
| STI20 | *AX-111496509* | 1B | 378,971,833 | 1.26E-04 | 3.15 |
| STI20 | *AX-109070579* | 1B | 379,583,836 | 1.12E-04 | 3.20 |
| STI20 | *AX-109271826* | 1B | 380,240,446 | 1.44E-04 | 3.10 |
| STI20 | *AX-111503092* | 1B | 380,647,070 | 5.72E-05 | 3.48 |
| STI20 | *AX-108973265* | 1B | 381,032,751 | 1.31E-04 | 3.14 |
| STI20 | *AX-111041532* | 1B | 381,315,130 | 4.39E-04 | 2.64 |
| STI20 | *AX-94515082* | 1B | 383,002,046 | 2.45E-04 | 2.88 |
| STI20 | *AX-109983759* | 1B | 383,176,594 | 1.05E-04 | 3.23 |
| STI20 | *AX-109419267* | 1B | 384,249,145 | 1.25E-04 | 3.16 |
| STI20 | *AX-108756721* | 1B | 384,566,103 | 7.96E-05 | 3.34 |
| STI20 | *AX-182144578* | 1B | 385,157,636 | 4.51E-04 | 2.63 |
| STI20 | *AX-111715015* | 1B | 385,300,997 | 1.79E-04 | 3.01 |
| STI20 | *AX-111468437* | 1B | 386,240,704 | 1.23E-04 | 3.16 |
| STI20 | *AX-109340517* | 1B | 386,689,382 | 1.49E-04 | 3.08 |
| STI20 | *AX-109391593* | 1B | 387,288,895 | 1.25E-04 | 3.16 |
| STI20 | *AX-110062945* | 1B | 387,783,486 | 6.81E-04 | 2.47 |
| STI20 | *AX-111549107* | 1B | 388,341,838 | 1.24E-04 | 3.16 |
| STI20 | *AX-110121329* | 1B | 388,895,243 | 8.27E-04 | 2.39 |
| STI20 | *AX-111022842* | 1B | 389,321,645 | 1.07E-04 | 3.22 |
| STI20 | *AX-109873586* | 1B | 389,879,147 | 7.27E-05 | 3.38 |
| STI20 | *AX-111109396* | 1B | 390,276,179 | 1.24E-04 | 3.16 |
| STI20 | *AX-110041061* | 1B | 390,870,930 | 2.42E-04 | 2.88 |
| STI20 | *AX-111759816* | 1B | 391,416,920 | 3.25E-04 | 2.77 |
| STI20 | *AX-110002681* | 1B | 391,917,068 | 2.52E-04 | 2.87 |
| STI20 | *AX-111540229* | 1B | 392,457,146 | 3.94E-04 | 2.69 |
| STI20 | *AX-110449412* | 1B | 395,727,013 | 4.22E-04 | 2.66 |
| STI20 | *AX-108747601* | 1B | 395,959,962 | 2.67E-04 | 2.85 |
| STI20 | *AX-111119209* | 1B | 397,999,065 | 6.35E-04 | 2.49 |
| STI20 | *AX-110437942* | 1B | 399,329,113 | 6.13E-04 | 2.51 |
| STI20 | *AX-111608582* | 1B | 400,300,433 | 2.20E-04 | 2.92 |
| STI20 | *AX-111013576* | 1B | 400,746,236 | 7.02E-04 | 2.45 |
| STI20 | *AX-109971835* | 1B | 401,333,244 | 6.96E-04 | 2.46 |
| STI20 | *AX-109447941* | 1B | 401,821,047 | 7.22E-04 | 2.44 |
| STI20 | *AX-108877371* | 1B | 402,337,255 | 7.96E-04 | 2.40 |
| STI20 | *AX-109272373* | 1B | 404,523,501 | 5.64E-04 | 2.54 |
| STI20 | *AX-111782039* | 1B | 404,883,727 | 3.10E-04 | 2.78 |
| STI20 | *AX-111744000* | 1B | 405,524,154 | 3.77E-04 | 2.71 |
| STI20 | *AX-109920004* | 1B | 405,985,965 | 1.52E-04 | 3.08 |
| STI20 | *AX-111701712* | 1B | 407,510,156 | 6.88E-04 | 2.46 |
| STI20 | *AX-110057926* | 1B | 407,869,097 | 2.08E-04 | 2.95 |
| STI20 | *AX-109356137* | 1B | 409,617,898 | 1.82E-04 | 3.00 |
| STI20 | *AX-111007632* | 1B | 410,299,329 | 2.23E-04 | 2.92 |
| STI20 | *AX-111569795* | 1B | 410,787,194 | 3.06E-04 | 2.79 |
| STI20 | *AX-108738808* | 1B | 411,177,595 | 2.47E-04 | 2.88 |
| STI20 | *AX-109835720* | 1B | 411,823,029 | 2.33E-04 | 2.90 |
| STI20 | *AX-110676933* | 1B | 412,236,309 | 2.79E-04 | 2.83 |
| STI20 | *AX-108888480* | 1B | 412,794,523 | 2.29E-04 | 2.91 |
| STI20 | *AX-110737060* | 1B | 413,387,645 | 2.29E-04 | 2.91 |
| STI20 | *AX-109888316* | 1B | 413,748,062 | 9.31E-04 | 2.34 |
| STI20 | *AX-110513953* | 1B | 414,157,717 | 2.81E-04 | 2.83 |
| STI20 | *AX-111161220* | 1B | 414,806,318 | 1.28E-04 | 3.15 |
| STI20 | *AX-111077672* | 1B | 415,335,518 | 2.66E-04 | 2.85 |
| STI20 | *AX-109404795* | 1B | 415,614,587 | 1.74E-04 | 3.02 |
| STI20 | *AX-110491218* | 1B | 416,171,678 | 1.62E-04 | 3.05 |
| STI20 | *AX-111649750* | 1B | 416,825,691 | 2.47E-04 | 2.88 |
| STI20 | *AX-111726835* | 1B | 417,400,703 | 3.41E-04 | 2.75 |
| STI20 | *AX-110419189* | 1B | 418,160,680 | 6.25E-04 | 2.50 |
| STI20 | *AX-110169030* | 1B | 418,617,796 | 2.45E-04 | 2.88 |
| STI20 | *AX-111083420* | 1B | 418,826,302 | 5.17E-05 | 3.52 |
| STI20 | *AX-108808608* | 1B | 419,407,395 | 1.69E-04 | 3.03 |
| STI20 | *AX-110368439* | 1B | 419,950,198 | 2.89E-04 | 2.81 |
| STI20 | *AX-108831096* | 1B | 420,568,994 | 5.61E-04 | 2.54 |
| STI20 | *AX-111672727* | 1B | 420,711,917 | 5.51E-04 | 2.55 |
| STI20 | *AX-111169510* | 1B | 430,155,688 | 2.56E-04 | 2.86 |
| STI20 | *AX-109421320* | 1B | 431,469,851 | 5.49E-04 | 2.55 |
| STI20 | *AX-110670988* | 1B | 489,973,360 | 8.29E-04 | 2.39 |
| STI20 | *AX-109541457* | 1B | 492,300,623 | 7.17E-04 | 2.45 |
| STI20 | *AX-111746919* | 1B | 503,663,047 | 6.74E-04 | 2.47 |
| STI20 | *AX-110598536* | 1B | 509,300,586 | 6.83E-04 | 2.46 |
| STI20 | *AX-94442756* | 1B | 509,901,004 | 3.54E-04 | 2.73 |
| STI20 | *AX-111780371* | 1B | 510,356,945 | 6.29E-05 | 3.44 |
| STI20 | *AX-108920917* | 1B | 511,050,019 | 3.04E-05 | 3.74 |
| STI20 | *AX-109457059* | 1B | 511,409,502 | 3.50E-05 | 3.68 |
| STI20 | *AX-109966524* | 1B | 512,107,159 | 6.88E-05 | 3.40 |
| STI20 | *AX-94532894* | 1B | 512,458,040 | 1.47E-04 | 3.09 |
| STI20 | *AX-108750098* | 1B | 512,924,054 | 4.31E-04 | 2.65 |
| STI20 | *AX-109516797* | 1B | 556,006,557 | 8.62E-04 | 2.37 |
| STI20 | *AX-89390302* | 1B | 662,208,958 | 8.37E-04 | 2.38 |
| STI20 | *AX-109273019* | 1B | 670,429,611 | 4.65E-04 | 2.62 |
| STI20 | *AX-110929436* | 1D | 201,924,443 | 6.47E-04 | 2.49 |
| STI20 | *AX-108842099* | 1D | 214,185,496 | 7.64E-04 | 2.42 |
| STI20 | *AX-94820546* | 1D | 318,497,325 | 6.14E-04 | 2.51 |
| STI20 | *AX-109306875* | 2A | 728,810,807 | 3.79E-04 | 2.70 |
| STI20 | *AX-110022500* | 2A | 734,503,082 | 3.99E-04 | 2.68 |
| STI20 | *AX-108757311* | 3B | 22,056,205 | 9.79E-04 | 2.32 |
| STI20 | *AX-111534973* | 3B | 41,633,676 | 1.77E-04 | 3.01 |
| STI20 | *AX-111040045* | 4A | 632,764,370 | 1.14E-04 | 3.19 |
| STI20 | *AX-109375057* | 4A | 633,308,367 | 9.76E-06 | 4.22 |
| STI20 | *AX-110468999* | 4A | 634,108,610 | 2.77E-05 | 3.78 |
| STI20 | *AX-111140650* | 4A | 634,368,256 | 4.82E-05 | 3.55 |
| STI20 | *AX-111453918* | 4A | 634,833,572 | 2.19E-05 | 3.88 |
| STI20 | *AX-110440161* | 4A | 635,292,292 | 2.20E-05 | 3.88 |
| STI20 | *AX-108950546* | 4A | 635,764,214 | 6.19E-06 | 4.41 |
| STI20 | *AX-109775486* | 4D | 413,584,571 | 5.22E-04 | 2.57 |
| STI20 | *AX-110561585* | 5B | 35,291,342 | 4.20E-04 | 2.66 |
| STI20 | *AX-108949832* | 5B | 326,508,028 | 7.72E-04 | 2.42 |
| STI20 | *AX-109815720* | 5B | 326,795,120 | 8.82E-04 | 2.36 |
| STI20 | *AX-111917292* | 5D | 38,931,819 | 1.80E-04 | 3.01 |
| STI20 | *AX-108930462* | 5D | 40,914,557 | 5.95E-04 | 2.52 |
| STI20 | *AX-110761858* | 5D | 430,322,682 | 9.29E-04 | 2.34 |
| STI20 | *AX-110323751* | 5D | 485,750,527 | 8.09E-05 | 3.34 |
| STI20 | *AX-108803615* | 5D | 486,092,519 | 7.67E-05 | 3.36 |
| STI20 | *AX-108910713* | 5D | 486,935,936 | 6.83E-04 | 2.46 |
| STI20 | *AX-109861425* | 5D | 488,693,044 | 9.47E-04 | 2.33 |
| STI20 | *AX-110939712* | 6A | 607,955,281 | 8.57E-04 | 2.37 |
| STI20 | *AX-109382255* | 6B | 497,267,595 | 9.61E-04 | 2.33 |
| STI20 | *AX-109929885* | 6B | 622,021,029 | 6.17E-04 | 2.51 |
| STI20 | *AX-110058991* | 6B | 653,089,227 | 6.27E-04 | 2.50 |
| STI20 | *AX-95173501* | 6B | 696,468,301 | 9.09E-04 | 2.35 |
| STI20 | *AX-109967269* | 6D | 22,183,383 | 2.66E-04 | 2.85 |
| STI20 | *AX-109447460* | 6D | 89,262,091 | 2.28E-05 | 3.86 |
| STI20 | *AX-110223575* | 6D | 202,316,296 | 3.09E-04 | 2.79 |
| STI20 | *AX-89432708* | 7B | 701,338,884 | 4.04E-04 | 2.68 |
| STI20 | *AX-111898961* | 7D | 107,933,478 | 8.83E-04 | 2.36 |
| STI20 | *AX-111798949* | 7D | 545,167,822 | 2.27E-04 | 2.91 |
| STI20 | *AX-108758043* | 7D | 547,277,450 | 1.52E-04 | 3.08 |
| STI20 | *AX-110949705* | 7D | 548,055,156 | 1.62E-04 | 3.05 |
| STI21 | *AX-111503092* | 1B | 380,647,070 | 7.60E-04 | 2.24 |
| STI21 | *AX-110041061* | 1B | 390,870,930 | 9.42E-04 | 2.16 |
| STI21 | *AX-111525116* | 1B | 428,802,849 | 4.99E-04 | 2.40 |
| STI21 | *AX-111169510* | 1B | 430,155,688 | 1.50E-04 | 2.85 |
| STI21 | *AX-110365668* | 1B | 443,330,272 | 3.07E-04 | 2.58 |
| STI21 | *AX-111599494* | 1B | 444,807,500 | 8.58E-04 | 2.19 |
| STI21 | *AX-110687238* | 1B | 448,131,731 | 4.99E-04 | 2.40 |
| STI21 | *AX-111594013* | 1B | 449,703,084 | 7.04E-04 | 2.27 |
| STI21 | *AX-108964423* | 1B | 450,603,208 | 5.21E-04 | 2.38 |
| STI21 | *AX-108725476* | 1B | 451,575,800 | 4.07E-04 | 2.47 |
| STI21 | *AX-108892781* | 1B | 657,274,040 | 6.61E-04 | 2.29 |
| STI21 | *AX-109290429* | 2A | 608,865,127 | 5.11E-04 | 2.39 |
| STI21 | *AX-110425132* | 2A | 705,807,076 | 6.30E-04 | 2.31 |
| STI21 | *AX-94495090* | 2A | 747,147,132 | 2.31E-04 | 2.68 |
| STI21 | *AX-94497666* | 2A | 748,441,942 | 6.21E-04 | 2.31 |
| STI21 | *AX-109352432* | 2B | 731,463,570 | 7.04E-04 | 2.27 |
| STI21 | *AX-112290193* | 2D | 83,161,369 | 6.77E-05 | 3.15 |
| STI21 | *AX-109359639* | 3B | 123,714,815 | 5.02E-04 | 2.39 |
| STI21 | *AX-111103248* | 3D | 178,761,794 | 5.86E-04 | 2.34 |
| STI21 | *AX-110432825* | 3D | 206,655,789 | 6.90E-04 | 2.28 |
| STI21 | *AX-109341556* | 3D | 221,046,278 | 5.86E-04 | 2.34 |
| STI21 | *AX-109626991* | 3D | 224,648,576 | 9.26E-04 | 2.17 |
| STI21 | *AX-109392999* | 3D | 236,169,118 | 8.15E-04 | 2.21 |
| STI21 | *AX-109407590* | 3D | 246,923,598 | 8.80E-04 | 2.18 |
| STI21 | *AX-110830021* | 3D | 260,816,674 | 7.03E-04 | 2.27 |
| STI21 | *AX-110468887* | 4A | 491,976,508 | 9.75E-04 | 2.15 |
| STI21 | *AX-111528491* | 4A | 496,110,069 | 9.18E-04 | 2.17 |
| STI21 | *AX-108870577* | 4A | 500,119,543 | 5.17E-04 | 2.38 |
| STI21 | *AX-110171894* | 4A | 605,753,144 | 4.42E-04 | 2.44 |
| STI21 | *AX-108777965* | 4A | 605,767,873 | 6.40E-04 | 2.30 |
| STI21 | *AX-110961937* | 4A | 605,785,021 | 3.35E-04 | 2.54 |
| STI21 | *AX-109375057* | 4A | 633,308,367 | 2.56E-04 | 2.65 |
| STI21 | *AX-110468999* | 4A | 634,108,610 | 4.61E-04 | 2.43 |
| STI21 | *AX-111140650* | 4A | 634,368,256 | 6.04E-04 | 2.32 |
| STI21 | *AX-111453918* | 4A | 634,833,572 | 3.69E-04 | 2.51 |
| STI21 | *AX-110440161* | 4A | 635,292,292 | 8.12E-04 | 2.21 |
| STI21 | *AX-108950546* | 4A | 635,764,214 | 3.19E-04 | 2.56 |
| STI21 | *AX-95160379* | 4B | 30,589,960 | 3.87E-10 | 7.97 |
| STI21 | *AX-110021330* | 4B | 30,863,602 | 1.03E-09 | 7.56 |
| STI21 | *AX-110123920* | 4B | 30,865,957 | 5.41E-05 | 3.24 |
| STI21 | *AX-94607619* | 4B | 32,252,401 | 4.47E-04 | 2.44 |
| STI21 | *AX-95630384* | 4B | 99,064,194 | 2.98E-04 | 2.59 |
| STI21 | *AX-111150060* | 4B | 660,589,159 | 7.36E-04 | 2.25 |
| STI21 | *AX-109936458* | 5A | 46,425,005 | 9.47E-04 | 2.16 |
| STI21 | *AX-109374431* | 5A | 595,601,337 | 5.28E-04 | 2.37 |
| STI21 | *AX-111055722* | 5D | 446,113,191 | 9.88E-04 | 2.14 |
| STI21 | *AX-109334618* | 6A | 328,130,345 | 8.43E-04 | 2.20 |
| STI21 | *AX-108944873* | 6A | 601,258,225 | 3.98E-04 | 2.48 |
| STI21 | *AX-111757493* | 6B | 658,833,342 | 5.83E-04 | 2.34 |
| STI21 | *AX-108948208* | 6B | 659,920,570 | 3.36E-04 | 2.54 |
| STI21 | *AX-110422139* | 6B | 687,186,077 | 9.70E-04 | 2.15 |
| STI21 | *AX-109270297* | 7A | 201,985,415 | 4.96E-04 | 2.40 |
| STI21 | *AX-111450326* | 7A | 215,177,875 | 3.22E-04 | 2.56 |
| STI21 | *AX-108808481* | 7A | 218,244,507 | 1.93E-04 | 2.75 |
| STI21 | *AX-110024904* | 7A | 224,492,523 | 7.94E-04 | 2.22 |
| STI21 | *AX-108736604* | 7A | 655,403,231 | 4.84E-04 | 2.41 |
| STI21 | *AX-111264354* | 7A | 677,686,885 | 9.21E-04 | 2.17 |
| STI21 | *AX-108807321* | 7A | 691,640,086 | 2.57E-04 | 2.64 |
| STI21 | *AX-109386434* | 7A | 692,683,014 | 6.71E-05 | 3.15 |
| STI21 | *AX-109817033* | 7B | 711,066,385 | 2.65E-04 | 2.63 |
| STI21 | *AX-111000584* | 7B | 715,289,272 | 2.03E-04 | 2.73 |
| STI21 | *AX-89658728* | 7B | 721,217,470 | 4.00E-04 | 2.48 |
| STI21 | *AX-108758043* | 7D | 547,277,450 | 8.92E-04 | 2.18 |
| STI21 | *AX-110949705* | 7D | 548,055,156 | 7.52E-04 | 2.24 |
| STI21 | *AX-108770812* | 7D | 561,926,335 | 8.94E-04 | 2.18 |

^a^ Physical position of the SNP in the reference genome (IWGSC RefSeq v1.0); ^b^ Phenotypic variation explained by the identified SNP; STI19, STI20, STI21 and STIA represented 2018 – 2019, 2019 – 2020, 2020 – 2021 and the mean value of three crop seasons for STI, respectively.
